# Copper-catalyzed synthesis of β-boryl cyclopropanes *via* 1,2-borocyclopropanation of aryl olefins with CO as the C1 source†

**DOI:** 10.1039/d3sc01090b

**Published:** 2023-05-05

**Authors:** Hui-Qing Geng, Xiao-Feng Wu

**Affiliations:** a Leibniz-Institut für Katalyse e.V. Albert-Einstein-Straße 29a 18059 Rostock Germany xiao-feng.wu@catalysis.de; b Dalian National Laboratory for Clean Energy, Dalian Institute of Chemical Physics, Chinese Academy of Sciences 116023 Dalian Liaoning China xwu2020@dicp.ac.cn

## Abstract

Cyclopropane represents one of the most critical rings and has been found present in various bioactive compounds, especially in clinical medicines. It can be synthesized by the reaction of olefins with diazo-derived carbenoids which are potentially hazardous. Carbonylation is a powerful tool for synthesizing carbonylated or carbon-extended compounds. In this communication, we describe a straightforward approach for synthesizing β-boryl cyclopropane derivatives catalyzed by an inexpensive copper catalyst with CO as the C1 source. This reaction was mediated by an *in situ* generated carbene intermediate and afforded a wide range of cyclopropane-containing organoboron compounds in moderate to good yields.

## Abstract 2

A one-pot 1,2-borocyclopropanation of aryl olefins for synthesizing β-boryl cyclopropanes was developed, catalyzed by the inexpensive copper catalyst. Various β-boryl cyclopropanes were produced in moderate to good yields.

## Graphical abstract

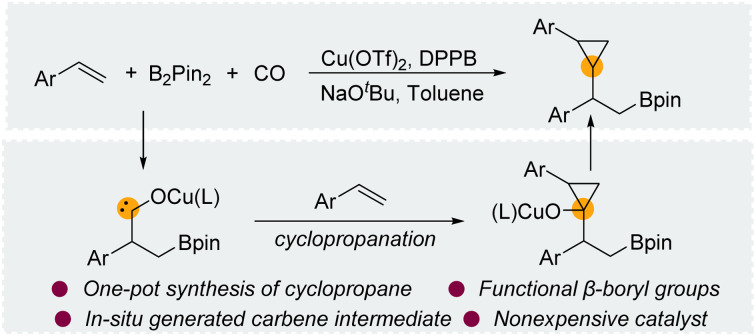

The cyclopropyl motif is an important structure found in various natural products, insecticides, and medicines (Fig. 1a).^1,2^ In small molecule medicines, cyclopropane was the sixth most common ring till 2022.^3^Cyclopropane can enhance the pharmacological properties of medicines, such as metabolic stability and lipolytic properties.^4^ Paxlovid, which was approved by the FDA for the treatment of SARS-COVID-19, contains a cyclopropane ring. In cyclopropane, the three coplanar and highly strained C–C bonds with similar reactivity to C=C double bonds and their C–H bonds are shorter and stronger than the C–H bond in other alkanes.^5–8^ The unusual chemical properties and structure of cyclopropane make it a fascinating and outstanding synthetic intermediate and also a valuable building block for many biologically active compounds.^9^

**Fig. 1:**
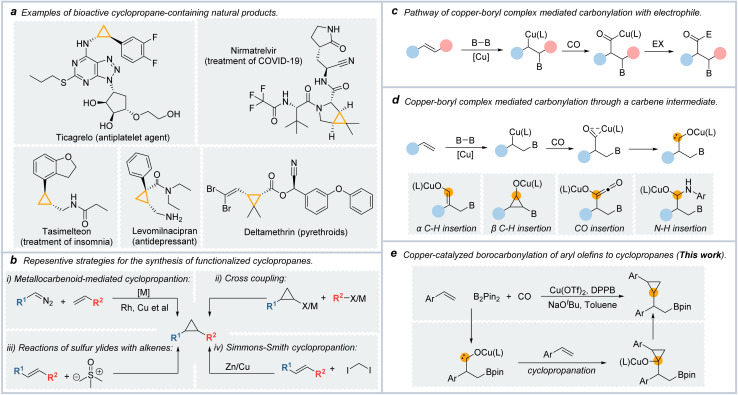
(a) Representative examples of cyclopropane-containing natural products. (b) Current methods for synthesizing derivatives of cyclopropanes. (c) Pathway for copper-catalyzed carbonylative borylation of alkenes. (d) Pathway for copper-catalyzed carbonylative borylation of alkenes through a carbene intermediate. (e) This work.

Chemists have achieved many efficient methods for synthesizing cyclopropane derivatives (Fig. 1b).^10–16^ Examples include the reaction of sulfur ylides with electron-poor olefins (the Corey–Chaykovsky reaction),^17^ and transition metal-catalyzed cross-coupling reaction of cyclopropane organometallic or cyclopropyl halides.^18,19^

Nowadays, the most popular and dependable strategies for preparing functionalized cyclopropyl motifs are [2 + 1]-type reactions with alkenes such as the Simmons–Smith cyclopropanation.^20,21^ Another important example is the transition metal-catalyzed cyclopropanation of alkenes with carbenoid species, which were generated from the composition of diazo compounds.^22,23^ However, this cyclopropanation method was limited in that the carbene species were generated from the decomposition of diazo compounds which are potentially hazardous.^24^

The carbonylation reactions represent one of the essential industrial methods for synthesizing carbonyl-containing compounds using CO as an inexpensive C1 source.^25,26^ Furthermore, the carbonylation reactions can be unitized in the carbon extension of their parent compounds. Noble metal catalyzed carbonylation reactions have been well developed, such as carbonylative cross-coupling reactions catalyzed by palladium.^26^ Considering the high price of these noble metals, more attention has focused on developing remarkable, inexpensive, and non-toxic catalyst systems for carbonylative reactions.

As a representation of base metals, copper is attractive due to its low price, low toxicity, and ready availability. Copper catalysts were highly active for many synthetic transformations, including C–C, C–B, C–N, and C–Si bond formations.^27^ Over the past few years, many synthetic methods have been reported by utilizing copper-boryl complexes as the reactive intermediates.^28^ Among them, Cu-catalyzed borofunctionalization was approved as a powerfully synthetic tool in that the borylation reagents obtained from this reaction could be used in further transformations such as C–C coupling reactions.^29^

In borocarbonylation, high-value β-boryl acyl compounds can be obtained to undergo a similar reaction mechanism. As shown in Fig. 1c, in the reaction of copper-boryl complexes with alkenes, the β-borylalkylcopper intermediate could be formed by the addition of a copper-boryl complex into the C=C bond. After the coordination and insertion of CO, acyl copper species were formed. In the presence of an electrophile, the final carbonylated products could be formed by the oxidative addition of acyl-copper species with the electrophile and then reductive elimination steps.^30,31^ In our recent studies on copper-boryl complex-mediated carbonylation reactions, we found that the acyl-copper intermediate could isomerize to carbene species in the absence of electrophilic reagents, which was different from the reported achievements in the borocarbonylation of alkenes (Fig. 1d). We could obtain a range of different products through the transformations of the *in situ* generated carbene intermediates including α-C–H insertion,^32^ β-C–H insertion,^33,34^ CO insertion,^35^ and N–H/O–H insertion of carbene.^36^

With our continued interest in copper-catalyzed borocarbonylation of olefins, we speculate that the carbene intermediates can be captured by C=C double bonds to generate cyclopropanes. In this communication, we describe a straightforward approach to synthesis β-boryl cyclopropanes *via* copper-catalyzed 1,2-borocyclopropanation of aryl olefins with CO as the C1 source (Fig. 1e).

We began our investigation by using styrene and bis(pinacolato)diboron (B_2_pin_2_) as the model substrates (for details, see the ESI†). Throughout the optimization process (Table 1), the hydroboration reaction provided the major by-product 3a. We could also detect trace amounts of 4a and 5a which may indicate the pathway of this reaction. To establish suitable reaction conditions, we initially screened different ligands (Table 1, entries 1–4, for details, see the ESI†). We found that only bidentate phosphines could give access to the β-boryl cyclopropane product. Among these ligands, the reaction with DPPB was influential in obtaining the target product (Table 1, entry 4). Moreover, no significant hydroboration by-product was observed. Then various parameters of this copper-catalyzed carbonylation, such as bases and solvent, were explored (for details, see the ESI†). In our testing of copper pre-catalysts, the results showed no significant difference between Cu(ii) and Cu(i) precursors (Table 1, entries 4–7, for details, see the ESI†). A higher loading of catalysts and slightly lower temperature increased both the yield of 2a and the dr value (Table 1, entries 8 and 9). When we used the combination of the Cu(OTf)_2_/DPPB/NaO^*t*^Bu system in toluene, 57% yield of 2a was obtained (Table 1, entry 9). When NaO^*t*^Bu or B_2_pin_2_ was decreased to 1 equivalent, only a trace amount of 2a was yielded (Table 1, entries 10 and 11). Increasing the amount of NaO^*t*^Bu and B_2_pin_2_ and meanwhile prolonging the reaction time to 20 hours can gave 77% yield of the desired 2a (Table 1, entry 12).

**Table 1:** Optimization of reaction conditions^a^

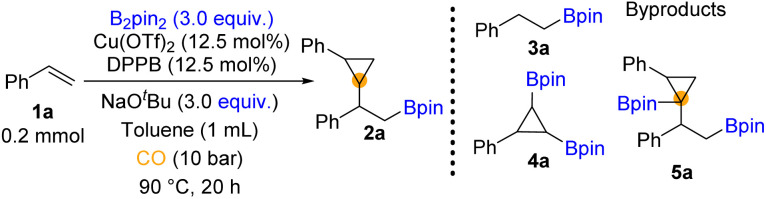				
Entry	Ligand (*x* mol%)	[Cu] (*y* mol%)	Dr^f^	Yield^g^ (%)
1	DPPF (10)	CuCl (10)	7 : 1	29
2	DPPPe (10)	CuCl (10)	7 : 1	13
3	DPEphos (10)	CuCl (10)	8 : 1	19
4	DPPB (10)	CuCl (10)	8 : 1	42
5	DPPB (10)	CuSO_4_ (10)	5 : 1	43
6	DPPB (10)	CuCl_2_ (10)	8 : 1	39
7	DPPB (10)	Cu(OTf)_2_ (10)	12 : 1	48
8	DPPB (12.5)	Cu(OTf)_2_ (12.5)	13 : 1	53
9^b^	DPPB (12.5)	Cu(OTf)_2_ (12.5)	13 : 1	57
10^c^	DPPB (12.5)	Cu(OTf)_2_ (12.5)	18 : 1	8
11^d^	DPPB (12.5)	Cu(OTf)_2_ (12.5)	—	Trace
12^e^	DPPB (12.5)	Cu(OTf)_2_ (12.5)	14 : 1	77

a Reaction conditions: 1a (0.2 mmol), [Cu] (*y* mol%), ligand (*x* mol%), NaO^*t*^Bu (2.5 equiv.), B_2_Pin_2_ (2.5 equiv.), toluene (1 mL), CO (10 bar), 100 °C, and 16 h.

b 90 °C, 16 h.

c NaO^*t*^Bu (1.0 equiv.), 90 °C, 16 h.

d B_2_Pin_2_ (1.0 equiv.), 90 °C, 16 h.

e NaO^*t*^Bu (3.0 equiv.), B_2_Pin_2_ (3.0 equiv.), 90 °C, and 20 h (standard conditions).

f The value of dr determined by GC.

g Yields are determined by GC with *n*-hexadecane as an internal standard.

With the optimized reaction conditions in hand, we turned to examine the scope of aryl olefins for this copper-catalyzed 1,2-borocyclopropanation reaction. As shown in Fig. 2, a broad spectrum of aryl olefins underwent the desired reaction and were transformed into the corresponding β-boryl cyclopropanes in moderate to good yields (2a–y). Aryl olefins bearing either electron-donating groups (*e.g.*, –Me, –MeO, –MeS, and –PhO) or electron-withdrawing (*e.g.*, –OCF_3_ and –SCF_3_) at the *para* position of the phenyl ring were all well reacted and provided the corresponding β-boryl cyclopropane products in moderate to good yields. Notably, 39% yield of product (2h) was produced from Bpin-substituted styrene. The reaction was not sensitive to the substitution pattern of the benzene ring. There was no significant difference in the reactivity of vinyl benzene substituted with fluorine atoms at the *para*, *meta*, or *ortho* positions, respectively (2i–k). The electron-withdrawing group (SCF_3_) and the electron-donating group were also applied at the *meta*-position of vinyl benzene, and good yields of the corresponding products were obtained (2l and 2m). Styrene decorated with sulfonyl (2n) and trifluoromethyl (2o) was also suitable for this carbonylative reaction to deliver the desired products in moderate yields. The adamantyl group substituted styrene provided the desired product 2p in 36% yield. Considering that heterocycles play an essential role in drug design due to their widespread presence in biologically active structures, we prepared cyclopropane products with various heterocycles. Heterocycles such as indole (2q), pyrrole (2r), furan (2s), morpholine (2t), and thiophene (2u) on the phenyl rings of styrenes could be successfully applied in the reaction and deliver the corresponding cyclopropane-containing products. Meanwhile, heterocyclic substituents containing two heteroatoms provided 2v and 2w in 47% and 53% yields, respectively. In addition, valid substrates containing complex-molecule-derived substrates, such as diacetone fructose- and citronellol-derived aryl olefins, can also be converted into products 2x and 2y in 44% and 43% yields, correspondingly.

**Fig. 2:**
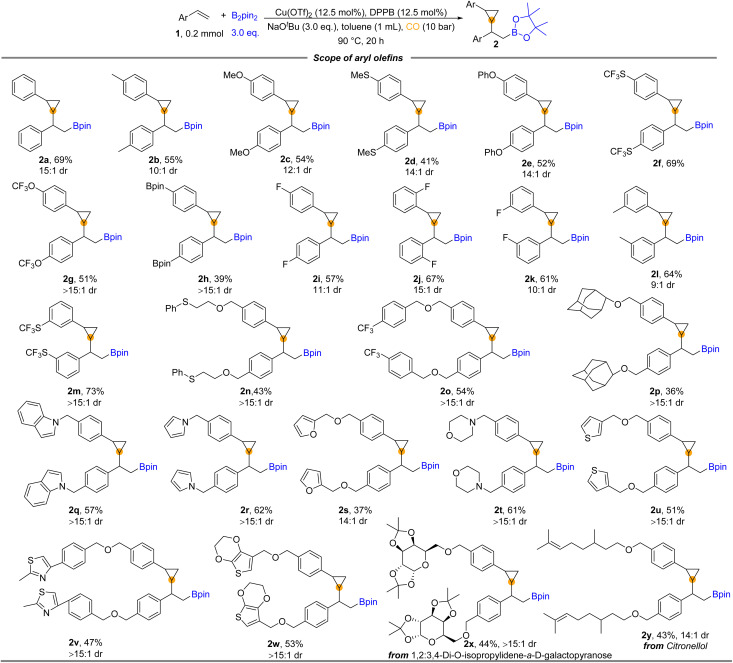
Substrate scope for the borocarbonylative reaction for synthesizing β-boryl cyclopropanes. Reaction conditions: 1 (0.2 mmol), Cu(OTf)_2_ (12.5 mol%), DPPB (12.5 mol%), NaO^*t*^Bu (3.0 equiv.), B_2_Pin_2_ (3.0 equiv.), toluene (1 mL), CO (10 bar), stirring at 90 °C for 20 h, and isolated yields. The value of dr determined by GC and ^1^H NMR.

We also depicted a possible mechanism for this carbonylation process (Fig. 3). In this transformation, the initiated copper catalyst reacted with the ligand, NaO^*t*^Bu, and B_2_pin_2_ to produce the (L)CuBpin complex. Then, after the addition of (L)CuBpin to styrene 1a and then the subsequent insertion of CO, the acyl-copper species II was delivered.^28^ Afterwards, the acyl-copper II species will isomerize to the carbene intermediate III. Next, the complex III will be captured by unsaturated C–C double bonds to give the cyclopropane-containing structure IV. In the presence of another equivalent of B_2_pin_2_, the complex IV afforded -OBpin substituted compound V.^37^ The OBpin group acted as a leaving group that activates the C–O bond of compound V. In the presence of an adequate amount of B_2_pin_2_ and base, compound V will be converted into compound VI, which could be detected by GC-MS during the optimization process. Finally, the final β-boryl substituted cyclopropanes could be eliminated after protodeborylation with a trace amount of water from reagents as the proton source.^38^ To support our mechanism, we applied ^13^CO in the reaction to obtain the ^13^C-labelled cyclopropane 2ae which demonstrates that CO was the source of one of the carbons in cyclopropane (Fig. 3B). In our performed reaction with α-deuterated styrene under the standard conditions 2af was obtained in 56% yield (Fig. 3C). The deuterium position in 2af indicated that there was no intramolecular hydrogen transfer.

**Fig. 3:**
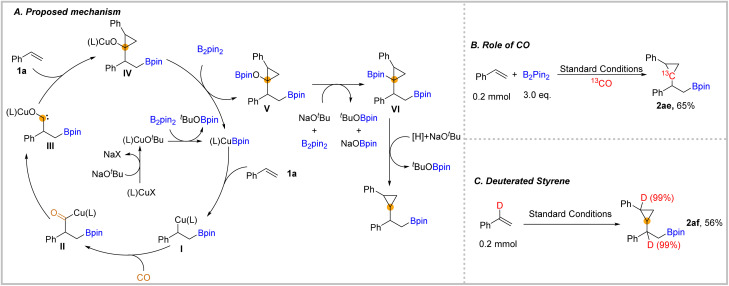
Isotope labelling experiments and proposed mechanism.

To demonstrate the utility of this 1,2-borocyclopropanation of aryl olefins, we conducted several diversification reactions of 2a (Fig. 4). As we expected, 2a can be oxidized by NaBO_3_·H_2_O to alcohol 2aa in 65% yield with a *cis* configuration.^39^ Zweifel-Olefination converts β-boryl substituted cyclopropane 2a to 2ab when the Grignard reagent is employed. Moreover, transformation to the trifluoroborate salt 2ac occurred in 77% yield. The further Suzuki–Miyaura reaction of 2ac can be successfully applied to deliver the desired product 2ad in 70% yield.

**Fig. 4:**
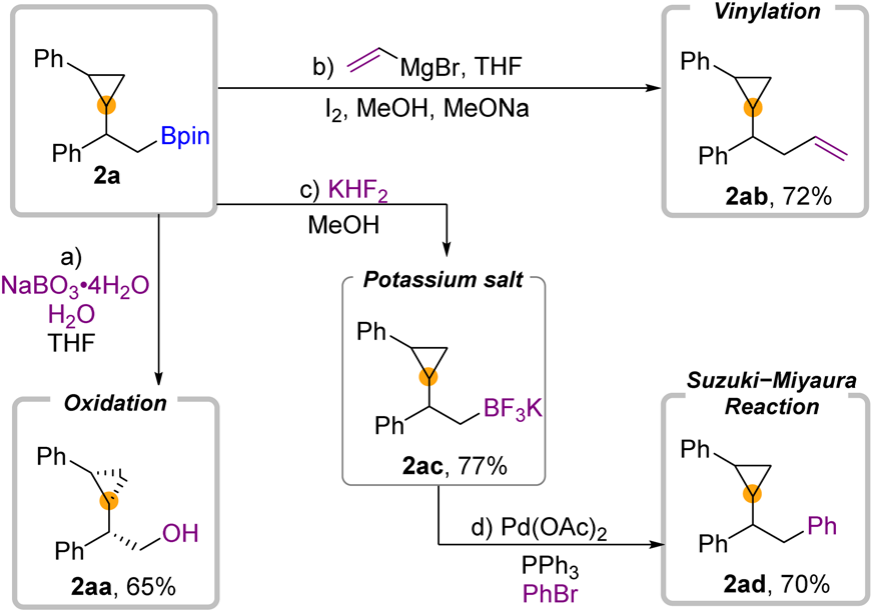
Synthetic application.

In conclusion, we have discovered a copper-catalyzed straightforward method for synthesizing cyclopropane-containing organoboron compounds. Taking advantage of the *in situ* carbene intermediate generation from the isomerization of the acyl-copper species, we can access the final β-boryl substituted cyclopropane structures by capturing the carbene intermediate with C=C bonds in another equivalent of aryl olefins. Various β-boryl cyclopropane derivatives were produced in moderate to good yields. Further synthetic applications of the formed β-boryl cyclopropane were successfully performed as well to demonstrate the value of this borocarbonylation process.

## Data availability

All data supporting the findings of this study are available within the article and its ESI file.†

## Author contributions

X.-F. W. conceived this project. H.-Q. G. performed all the experiments and prepared the ESI.† X.-F. W. and H.-Q. G. wrote and revised the manuscript.

## Conflicts of interest

The authors declare no competing financial interest.

## Supplementary Material

SC-014-D3SC01090B-s001
