# Supplementary material for: Copper-catalyzed synthesis of β-boryl cyclopropanes via 1,2-borocyclopropanation of aryl olefins with CO as the C1 source
Source: Chem Sci. 2023 May 5;14(21):5638–42. doi: 10.1039/d3sc01090b (PMC10231323; doi:10.1039/d3sc01090b)
Supplement: SC-014-D3SC01090B-s001 [file SC-014-D3SC01090B-s001.pdf]

Electronic Supplementary Material (ESI) for Chemical Science.
This journal is © The Royal Society of Chemistry 2023

Supporting Information

Copper-catalyzed synthesis of *β*-boryl cyclopropanes via 1,2-borocyclopropanation of aryl olefins with CO as the C1 source

Hui-Qing Geng, [a] and Xiao-Feng Wu* [a,b]

[a] Leibniz-Institut für Katalyse e.V., Albert-Einstein-Straße 29a, 18059 Rostock (Germany).
[b] Dalian National Laboratory for Clean Energy, Dalian Institute of Chemical Physics, Chinese Academy of Sciences, 116023, Dalian, Liaoning, China, E-mail: xwu2020@dicp.ac.cn

Table of Contents

1. General information S2
2. Optimization of reaction conditions S2
3. General procedures for synthesis of **2**. S8
4. Characterization Data. S9
5. Derivatization of **2a**. S18
   5.1 Oxidation of **2a.** S18
   5.2 Vinylation of **2a.** S18
   5.3 BF_3K preparation**.** S18
   5.4 Suzuki−Miyaura Reaction of **2ac.** S19
6. Mechanism studies. S20
   6.1 ^{13}C labeling experiment. S20
   6.2 Deuterated Styrene as starting material. S20
   6.3 Deuterated solvent. S20
   6.4 Additional deuterium water. S22
7. References. S23
8. Spectra Copies. S24

1. General information

Unless otherwise stated, all experiments were performed under carbon monoxide or nitrogen atmosphere. Aryl olefins were prepared according to previous references. All chemical reagents were purchased Sigma-Aldrich, TCI, ABCR, and Acros of the highest purity grade and used without purification. All solvents were dried by standard techniques. Silica gel column chromatography was carried out using silica gel (200–300 mesh) and *n*-pentane and ethyl acetate as eluent. Analytical thin layer chromatography (TLC) was performed on silica gel (silica gel 60 DC-Platten ALUGRAM® Xtra SIL G / UV_{254}). TLC plates were visualized with UV light, and/or submersion in $KMnO_4$ solution). All NMR spectra were recorded with Bruker Avance III HD 300 NMR (^1H, 300 MHz; $^{13}C\{^1H\}$, 75 MHz; ^{11}B, 96 MHz), Bruker ARX 400 NMR spectrometers (^1H, 400 MHz; $^{13}C\{^1H\}$, 101 MHz). Chemical shifts are reported as δ values relative to internal chloroform (δ 7.26 for ^1H NMR) and TMS (δ 0.00 for ^1H NMR) chloroform (δ 77.16 for $^{13}C\{^1H\}$ NMR) in parts per million (ppm). The following abbreviations are used for the NMR spectra' multiplicities: s: singlet, d: doublet, dd: doublet of doublet, t: triplet, q: quartet, m: multiplet, and br: broad signal for proton spectra. All ^{13}C NMR spectra were broad-band ^1H decoupled. However, it is hard to observe the signals for the carbon attached to boron in the $^{13}C\{^1H\}$ NMR spectra. Gas chromatography (GC) analyses were performed on an Agilent HP-7890A instrument with an FID detector and HP-5 capillary column (polydimethylsiloxane with 5% phenyl groups, 30 m, 0.32 mm i.d. 0.25 μm film thickness) using argon as carrier gas. High-resolution mass spectra were recorded on an Agilent 6210 system. All the reactions which used CO were performed in an autoclave. The laboratory should be well-equipped with a CO detector and alarm system.

2. Optimization of reaction conditions.

***Table S1*. Screening of ligand**[a]

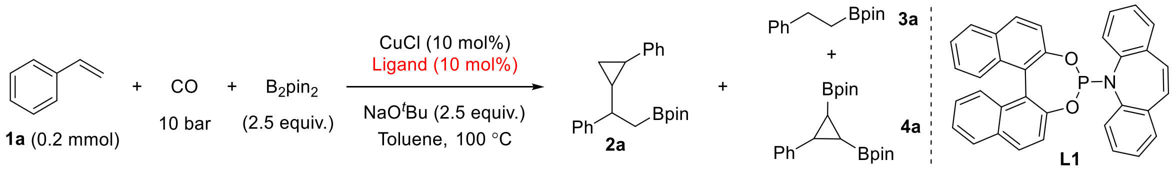

Entry	Ligand	**2a** (%)	**dr**	**3a** (%)	**4a** (%)
1	DPPP	-	-	Trace	16
2	DPPE	-	-	Trace	10
3	DPPF	29	7:1	Trace	-
4	Xantphos	-	-	Major[c]	-
5	BINAP	-	-	Major[c]	-
6	DPEphos	19	8:1	Trace	11
7[b]	PPh_3	-	-	Major[c]	-
8	IPr	-	-	-	-
9	IMes	-	-	-	-
10	DPPM	6	-	Major[c]	-
11	DPPB	42	8:1	Trace	-
12	DPPPe	13	7:1	Trace	6
13	DPPBz	-	-	Trace	-
14	BPY	-	-	-	-

15	**L1**	-	-	Trace	-

[a] Reaction conditions: **1a** (0.2 mmol), B_2Pin_2 (2.5 equiv.), CuCl (10 mol%), ligand (10 mol%), NaO*t*Bu (2.5 equiv.), Toluene (1 mL), CO (10 Bar), 100 °C, 16 h. Yields are determined by GC with hexadecane as an internal standard. The yield of **2a** is calculated based on 0.1 mmol styrene. Because two equivalents of **1a** are required to produce one equivalent of **2a**. The value of dr determined by GC. [b] PPh_3 (20 mol%). [c] **3a** is the major product.

***Table S2*. Screening of base**[a]

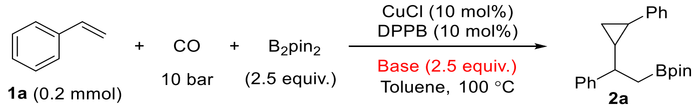

Entry	Base	**2a (%)**	**dr**
1	LiO*t*Bu	Trace	-
2	LiOMe	Trace	-
3	NaOEt	Trace	-
4	KO*t*Bu	7	4:1
5	KOMe	Trace	-
6	NaO*t*Bu	42	8:1

[a] Reaction Conditions: **1a** (0.2 mmol), CuCl (10 mol%), DPPB (10 mol%), Base (2.5 equiv.), B_2Pin_2 (2.5 equiv.), Toluene (1 mL), CO (10 bar), 100 °C, 16 h. Yields are determined by GC with hexadecane as an internal standard. The yield of **2a** is calculated based on 0.1 mmol styrene. Because two equivalents of **1a** are required to produce one equivalent of **2a**. The value of dr determined by GC.

***Table S3*. Screening of solvent**[a]

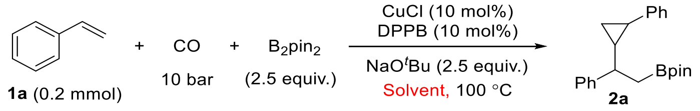

Entry	Solvent	**2a (%)**	**dr**
1	DMAc	4	8:1
2	1,4-dioxane	2	-
3	DCE	18	8:1
4	MeCN	Trace	-
5	DMF	6	-
6	Et_2O	10	-
7	DMSO	4	-
8	Cyalohexane	Trace	-
9	PhF	36	11:1

10	PhCl	34	5:1
11	$PhCF_3$	35	7:1
12	PhEt	39	9:1
13	Toluene	42	8:1

[a] Reaction Conditions: **1a** (0.2 mmol), CuCl (10 mol%), DPPB (10 mol%), NaO^{t}Bu (2.5 equiv.), B_2Pin_2 (2.5 equiv.), solvent (1 mL), CO (10 bar), 100 °C, 16 h. Yields are determined by GC with hexadecane as an internal standard. The yield of **2a** is calculated based on 0.1 mmol styrene. Because two equivalents of **1a** are required to produce one equivalent of **2a**. The value of dr determined by GC.

***Table S4*. Screening of copper source**[a]

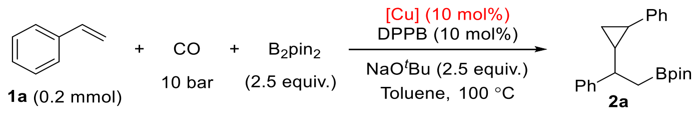

Entry	[Cu]	**2a** (%)	**dr**
1	$CuCl_2$	39	8:1
2	CuI	26	8:1
3	CuBr	25	13:1
4	$Cu(OAc)_2$	14	9:1
5	$Cu(OTf)_2$	48	12:1
6	CuCN	4	-
7	$Cu(acac)_2$	47	8:1
8	$CuCl_2 + AgBF_4$	37	11:1
9	$CuCl_2 + AgNO_3$	n.d.	-
10	$Cu(PPh_3)_2NO_3$	10	5:1
11	$Cu(NO_3)_2$•$3H_2O$	29	6:1
12	$Cu(TFA)_2$•xH_2O	31	6:1
13	$CuSO_4$	43	5:1

[a] Reaction Conditions: **1a** (0.2 mmol), [Cu] (10 mol%), DPPB (10 mol%), NaO^{t}Bu (2.5 equiv.), B_2Pin_2 (2.5 equiv.), Toluene (1 mL), CO (10 bar), 100 °C, 16 h. Yields are determined by GC with hexadecane as an internal standard. The yield of **2a** is calculated based on 0.1 mmol styrene. Because two equivalents of **1a** are required to produce one equivalent of **2a**. The value of dr determined by GC.

***Table S5*. Screening of amount of $Cu(OTf)_2$ and DPPB[a]**

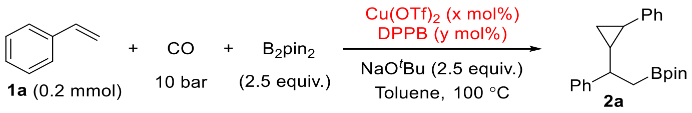

Entry	$Cu(OTf)_2$	DPPB	**2a** (%)	**dr**
1	2.5 mol%	2.5 mol%	15	13:1
2	5 mol%	5 mol%	37	12:1
3	7.5 mol%	7.5 mol%	47	13:1
4	10 mol%	10 mol%	48	13:1
5	12.5 mol%	12.5 mol%	53	13:1
6	15 mol%	15 mol%	38	13:1
7	10 mol%	12.5 mol%	28	12:1
8	10 mol%	15 mol%	35	13:1
9	10 mol%	20 mol%	20	12:1

[a] Reaction Conditions: **1a** (0.2 mmol), $Cu(OTf)_2$ (x mol%), DPPB (y mol%), NaO^tBu (2.5 equiv.), B_2Pin_2 (2.5 equiv.), Toluene (1 mL), CO (10 bar), 100 °C, 16 h. Yields are determined by GC with hexadecane as an internal standard. The yield of **2a** is calculated based on 0.1 mmol styrene. Because two equivalents of **1a** are required to produce one equivalent of **2a**. The value of dr determined by GC. The value of dr determined by GC.

***Table S6*. Screening of temperature[a]**

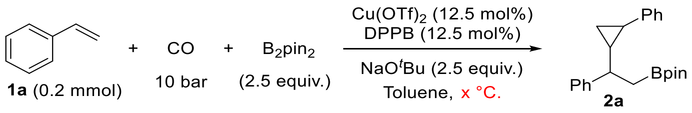

Entry	Temperature	**2a** (%)	**dr**
1	80 °C	43	11:1
2	90 °C	57	10:1
3	100 °C	53	10:1
4	110 °C	33	10:1
5	120 °C	54	10:1
6	130 °C	52	10:1
7	140 °C	38	10:1

[a] Reaction Conditions: **1a** (0.2 mmol), $Cu(OTf)_2$ (12.5 mol%), DPPB (12.5 mol%), NaO^tBu (2.5 equiv.), B_2Pin_2 (2.5 equiv.), Toluene (1 mL), CO (10 bar), x °C, 16 h. Yields are determined by GC with hexadecane as an internal standard. The yield of **2a** is calculated based on 0.1 mmol styrene. Because two equivalents of **1a** are required to produce one equivalent of **2a**. The value of dr determined by GC.

***Table S7*. Screening of the amount of NaO^{t}Bu**[a]

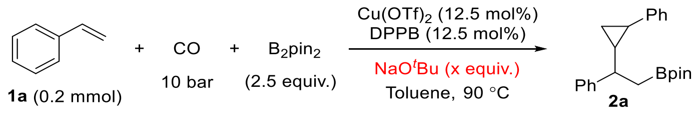

Entry	The amount of NaO^{t}Bu	**2a** (%)	**dr**
1	w/o	n.d.	-
2	12.5 mol%	n.d.	-
3	1 equiv.	8	18:1
4	1.25 equiv.	31	13:1
5	1.75 equiv.	20	11:1
6	2 equiv.	29	11:1
7	2.25 equiv.	45	11:1
8	2.5 equiv.	57	12:1
9	2.75 equiv.	61	12:1
10	3.0 equiv.	63	12:1
11	3.5 equiv.	56	11:1
12	3.75 equiv.	48	10:1

[a] Reaction Conditions: **1a** (0.2 mmol), $Cu(OTf)_2$ (12.5 mol%), DPPB (12.5 mol%), NaO^{t}Bu (x equiv.), B_2Pin_2 (2.5 equiv.), Toluene (1 mL), CO (10 bar), 90 °C, 16 h. Yields are determined by GC with hexadecane as an internal standard. The yield of **2a** is calculated based on 0.1 mmol styrene. Because two equivalents of **1a** are required to produce one equivalent of **2a**. The value of dr determined by GC.

***Table S8*. Screening of the amount of toluene**[a]

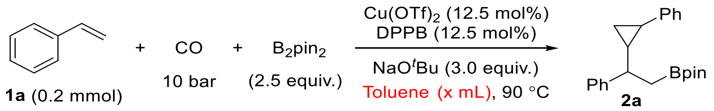

Entry	The amount of toluene	**2a** (%)	**dr**
1	0.5 mL	37	11:1
2	1.0 mL	63	12:1
3	2 mL	46	12:1
4	2.5 mL	24	12:1

[a] Reaction Conditions: **1a** (0.2 mmol), $Cu(OTf)_2$ (12.5 mol%), DPPB (12.5 mol%), NaO^{t}Bu (3.0 equiv.), B_2Pin_2 (2.5 equiv.), Toluene (x mL), CO (10 bar), 90 °C, 16 h. Yields are determined by GC with hexadecane as an internal standard. The yield of **2a** is calculated based on 0.1 mmol styrene. Because two equivalents of **1a** are required to produce one equivalent of **2a**. The value of dr determined by GC.

***Table S9*. Screening of the amount of B_2pin_2[a]**

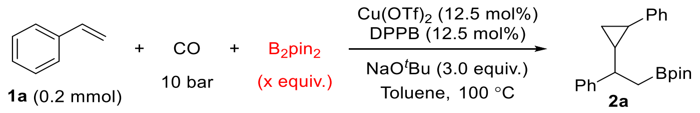

Entry	The amount of B_2pin_2	**2a** (%)	**dr**
1	1 equiv.	trace	-
2	1.5 equiv.	24	12:1
3	1.75 equiv.	62	12:1
4	2 equiv.	64	13:1
5	2.5 equiv.	63	12:1
6	3.0 equiv.	74	12:1
7	3.5 equiv.	53	13:1

[a] Reaction Conditions: **1a** (0.2 mmol), $Cu(OTf)_2$ (12.5 mol%), DPPB (12.5 mol%), NaO^tBu (3.0 equiv.), B_2Pin_2 (x equiv.), Toluene (1 mL), CO (10 bar), 90 °C, 16 h. Yields are determined by GC with hexadecane as an internal standard. The yield of **2a** is calculated based on 0.1 mmol styrene. Because two equivalents of **1a** are required to produce one equivalent of **2a**. The value of dr determined by GC.

***Table S10*. Screening of the reaction time[a]**

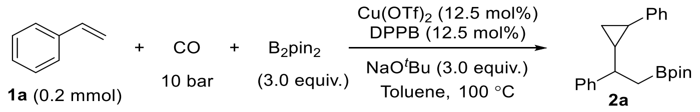

Entry	Reaction time	**2a** (%)	**dr**
1	16 h	74	12:1
2	20 h	77 (69[c])	14:1
3[b]	20 h	62	14:1

[a] Reaction Conditions: **1a** (0.2 mmol), $Cu(OTf)_2$ (12.5 mol%), DPPB (12.5 mol%), NaO^tBu (3.0 equiv.), B_2Pin_2 (3.0 equiv.), Toluene (1 mL), CO (10 bar), 90 °C, x h. Yields are determined by GC with hexadecane as an internal standard. The yield of **2a** is calculated based on 0.1 mmol styrene. Because two equivalents of **1a** are required to produce one equivalent of **2a**. The value of dr determined by GC. [b] 4Å MS (30 mg). [c] Isolated yield.

3. General procedures for synthesis of 2.

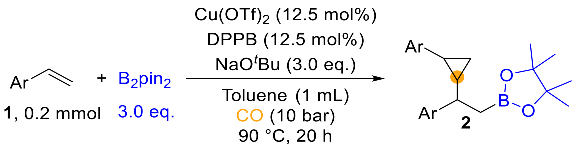

A dried vial (4 mL) was charged with $Cu(OTf)_2$ (12.5 mol%), DPPB (12.5 mol%), B_2pin_2 (3.0 equiv.), NaO^tBu (3.0 equiv.), and a stirring bar. The vial was sealed by PTFE/white rubber septum (Wheaton 13 mm Septa) and phenolic cap and connected with atmosphere with a needle. The vial was evacuated under vacuum and recharged with argon for three times. Then, aryl olefins (0.2 mmol) and toluene (1.0 mL) were added under argon by using a syringe. The vial (or several vials) was placed in an alloy plate and transferred into a 300 mL autoclave of the 4560 series from Parr Instruments. The autoclave was charged with CO (10 bar) after flushing two times with N_2 and two times with CO. The autoclave was then placed into an aluminum block on a magnetic stirrer. The reaction was performed for 20 h at 90 °C. After the reaction was complete, the autoclave was cooled down with ice water to room temperature and the pressure was released carefully. After that the residue was directly purified by column chromatography to afford the corresponding products.

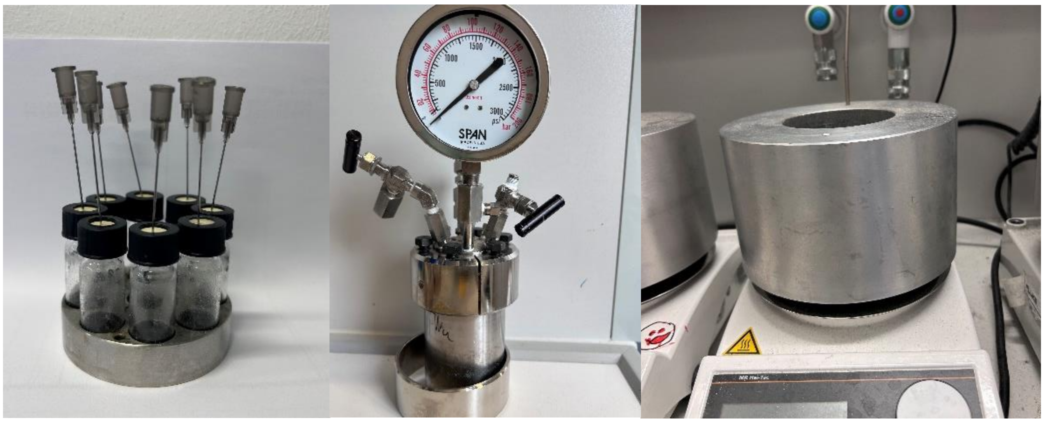

4. Characterization Data.

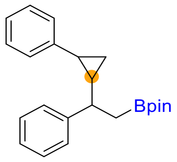

2a, 69%

4,4,5,5-Tetramethyl-2-(2-phenyl-2-(2-phenylcyclopropyl)ethyl)-1,3,2-dioxaborolane (2a)

The title compound was prepared from styrene (0.2 mmol), according to general procedure. The crude residue was purified by flash chromatography (*n*-pentane/EA = 100:1, Rf = 0.2) to give the product as a colorless oil (24 mg, 69%).

^{1}H NMR (300 MHz, $CDCl_3$) δ 7.29 – 7.26 (m, 2H), 7.25 – 7.05 (m, 8H), 2.39 (td, *J* = 9.3, 6.3 Hz, 1H), 1.92 – 1.81 (m, 1H), 1.40 – 1.32 (m, 3H), 1.06 (s, 6H), 1.02 (s, 6H), 0.87 – 0.77 (m, 2H).

^{13}C NMR (75 MHz, $CDCl_3$) δ 146.4, 143.6, 128.2, 128.1, 127.4, 126.0, 126.0, 125.2, 83.0, 46.1, 31.8, 24.7, 24.5, 23.8, 15.8.

^{11}B NMR (96 MHz, $CDCl_3$) δ 33.0.

HRMS (**EI**): calcd for $[M]^+$ $C_{23}H_{29}BO_2$ 348.2255, found: 348.2259.

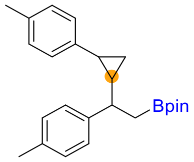

2b, 55%

4,4,5,5-Tetramethyl-2-(2-(*p*-tolyl)-2-(2-(*p*-tolyl)cyclopropyl)ethyl)-1,3,2-dioxaborolane (2b)

The title compound was prepared from 1-methyl-4-vinylbenzene (0.2 mmol), according to general procedure. The crude residue was purified by flash chromatography (*n*-pentane/EA = 100:1, Rf = 0.2) to give the product as a colorless oil (21 mg, 55%).

^{1}H NMR (300 MHz, $CDCl_3$) δ 7.18 – 7.14 (m, 2H), 7.11 – 7.03 (m, 4H), 6.98 (d, *J* = 8.2 Hz, 2H), 2.37 – 2.28 (m, 7H), 1.85 (dd, *J* = 9.0, 4.5 Hz, 1H), 1.31 (dt, *J* = 13.2, 3.4 Hz, 3H), 1.07 (d, *J* = 8.5 Hz, 12H), 0.85 – 0.73 (m, 2H).

^{13}C NMR (75 MHz, $CDCl_3$) δ 143.4, 140.6, 135.3, 134.7, 128.8, 128.8, 127.2, 125.9, 82.9, 45.6, 31.6, 24.7, 24.6, 23.4, 21.0, 20.9, 15.5.

^{11}B NMR (96 MHz, $CDCl_3$) δ 33.2.

HRMS (**EI**): calcd for $[M]^+$ $C_{25}H_{33}BO_2$ 376.2568, found: 376.2574.

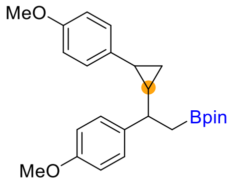

2c, 54%

2-(2-(4-Methoxyphenyl)-2-(2-(4-methoxyphenyl)cyclopropyl)ethyl)-4,4,5,5-tetramethyl-1,3,2-dioxaborolane (2c)

The title compound was prepared from 1-methoxy-4-vinylbenzene (0.2 mmol), according to general procedure. The crude residue was purified by flash chromatography (*n*-pentane/EA = 20:1, Rf = 0.2) to give the product as a colorless oil (22 mg, 54%).

^{1}H NMR (300 MHz, $CDCl_3$) δ 7.22 – 7.14 (m, 2H), 7.11 – 6.94 (m, 2H), 6.85 – 6.76 (m, 4H), 3.79 (s, 3H), 3.77 (s, 3H), 2.34 (td, *J* = 9.3, 6.1 Hz, 1H), 1.81 (dt, *J* = 9.1, 5.0 Hz, 1H), 1.30 – 1.25 (m, 3H), 1.08 (s, 6H), 1.05 (s, 6H), 0.82 – 0.67 (m, 2H).

^{13}C NMR (75 MHz, $CDCl_3$) δ 157.8, 157.5, 138.7, 135.6, 128.3, 127.1, 113.6, 113.5, 83.0, 55.3, 55.2, 45.2, 31.4, 24.8, 24.6, 22.9, 15.1.

^{11}B NMR (96 MHz, $CDCl_3$) δ 33.1.

HRMS (**EI**): calcd for $[M]^+$ $C_{25}H_{33}BO_4$ 408.2466, found: 408.2474.

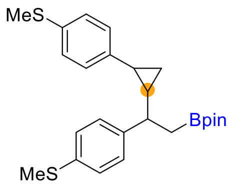

2d, 41%

4,4,5,5-Tetramethyl-2-(2-(4-(methylthio)phenyl)-2-(2-(4-(methylthio)phenyl)cyclopropyl)ethyl)-1,3,2-dioxaborolane (2d)

The title compound was prepared from methyl(4-vinylphenyl)sulfane (0.2 mmol), according to general procedure. The crude residue was purified by flash chromatography (*n*-pentane/EA = 30:1, Rf = 0.3) to give the product as a colorless oil (18 mg, 41%).

^{1}H NMR (300 MHz, $CDCl_3$) δ 7.21 – 7.15 (m, 6H), 7.01 (d, *J* = 8.2 Hz, 2H), 2.46 (s, 3H), 2.45 (s, 3H), 2.40 – 2.32 (m, 1H), 1.87 – 1.77 (m, 1H), 1.35 – 1.26 (m, 3H), 1.08 (s, 6H), 1.05 (s, 6H), 0.84 – 0.74 (m, 2H).

^{13}C NMR (75 MHz, $CDCl_3$) δ 143.6, 140.9, 128.0, 127.4, 127.1, 126.6, 83.1, 45.5, 31.6, 24.8, 24.6, 23.3, 16.6, 16.4, 15.6.

^{11}B NMR (96 MHz, $CDCl_3$) δ 33.1.

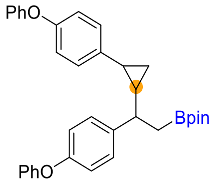

2e, 52%

4,4,5,5-Tetramethyl-2-(2-(4-phenoxyphenyl)-2-(2-(4-phenoxyphenyl)cyclopropyl)ethyl)-1,3,2-dioxaborolane (2e)

The title compound was prepared from 1-phenoxy-4-vinylbenzene (0.2 mmol), according to general procedure. The crude residue was purified by flash chromatography (*n*-pentane/EA = 20:1, Rf = 0.2) to give the product as a colorless oil (28 mg, 52%).

^{1}H NMR (300 MHz, $CDCl_3$) δ 7.40 – 7.29 (m, 4H), 7.28 (s, 2H), 7.14 – 7.05 (m, 4H), 7.04 – 6.85 (m, 8H), 2.42 (td, *J* = 9.3, 6.1 Hz, 1H), 1.95 – 1.85 (m, 1H), 1.43 – 1.30 (m, 3H), 1.12 (d, *J* = 9.7 Hz, 12H), 0.87 – 0.79 (m, 2H).

^{13}C NMR (75 MHz, $CDCl_3$) δ 157.8, 129.6, 128.6, 127.3, 122.8, 122.8, 119.1, 119.0, 118.4, 118.3, 83.1, 45.4, 31.6, 24.8, 24.5, 23.2, 15.6.

^{11}B NMR (96 MHz, $CDCl_3$) δ 33.54.

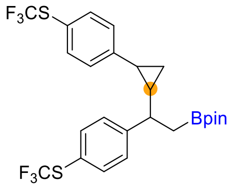

2f, 69%

4,4,5,5-Tetramethyl-2-(2-(4-((trifluoromethyl)thio)phenyl)-2-(2-(4-((trifluoromethyl)thio)phenyl)cyclopropyl)ethyl)-1,3,2-dioxaborolane (2f)

The title compound was prepared from (trifluoromethyl)(4-vinylphenyl)sulfane (0.2 mmol), according to general procedure. The crude residue was purified by flash chromatography (*n*-pentane/EA = 30:1, Rf = 0.2) to give the product as a colorless oil (38 mg, 69%).

^{1}H NMR (300 MHz, $CDCl_3$) δ 7.60 – 7.50 (m, 4H), 7.35 – 7.31 (m, 2H), 7.12 – 7.09 (m, 2H), 2.43 (td, *J* = 9.4, 6.1 Hz, 1H), 1.91 (dt, *J* = 8.8, 5.0 Hz, 1H), 1.43 – 1.37 (m, 3H), 1.05 (s, 6H), 1.00 (s, 6H), 0.89 – 0.83 (m, 2H).

^{13}C NMR (75 MHz, $CDCl_3$) δ 149.4, 146.9, 136.4, 131.1 (q, *J* = 309.5 Hz), 129.60 (q, *J* = 307.7 Hz), 128.6, 126.9, 83.2, 45.9, 29.7, 24.6, 24.4, 23.8, 16.5.

^{11}B NMR (96 MHz, $CDCl_3$) δ 32.9.

^{19}F NMR (282 MHz, $CDCl_3$) δ -43.13, -43.22.

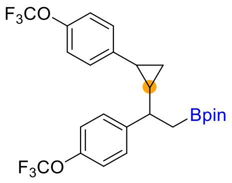

2g, 51%

4,4,5,5-Tetramethyl-2-(2-(4-(trifluoromethoxy)phenyl)-2-(2-(4-(trifluoromethoxy)phenyl)cyclopropyl)ethyl)-1,3,2-dioxaborolane (2g)

The title compound was prepared from 1-(trifluoromethoxy)-4-vinylbenzene (0.2 mmol), according to general procedure. The crude residue was purified by flash chromatography (*n*-pentane/EA = 30:1, Rf = 0.2) to give the product as a colorless oil (26 mg, 51%).

^{1}H NMR (300 MHz, $CDCl_3$) δ 7.36 – 7.27 (m, 2H), 7.13 (ddd, *J* = 8.6, 3.4, 1.7 Hz, 2H), 7.09 (s, 4H), 2.40 (td, *J* = 9.4, 6.1 Hz, 1H), 1.88 (dt, *J* = 8.6, 5.2 Hz, 1H), 1.37 (t, *J* = 3.0 Hz, 3H), 1.06 (s, 6H), 1.02 (s, 6H), 0.87 – 0.84 (m, 2H).

^{13}C NMR (75 MHz, $CDCl_3$) δ 145.0, 142.1, 128.6, 127.2, 120.9, 120.8, 120.5 (q, *J* = 256.6 Hz), 83.2, 45.5, 31.5, 24.7, 24.5, 23.3, 15.9.

^{11}B NMR (96 MHz, $CDCl_3$) δ 33.6.

^{19}F NMR (282 MHz, $CDCl_3$) δ -58.0, -58.0.

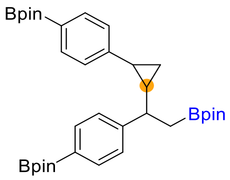

2h, 39%

4,4,5,5-Tetramethyl-2-(4-(2-(4,4,5,5-tetramethyl-1,3,2-dioxaborolan-2-yl)-1-(2-(4-(4,4,5,5-tetramethyl-1,3,2-dioxaborolan-2-yl)phenyl)cyclopropyl)ethyl)phenyl)-1,3,2-dioxaborolane (2h)

The title compound was prepared from 4,4,5,5-tetramethyl-2-(4-vinylphenyl)-1,3,2-dioxaborolane (0.2 mmol), according to general procedure. The crude residue was purified by flash chromatography (*n*-pentane/EA = 50:1, Rf = 0.2) to give the product as a colorless oil (23 mg, 39%).

^{1}H NMR (300 MHz, $CDCl_3$) δ 7.73 (dd, *J* = 16.9, 8.1 Hz, 4H), 7.30 – 7.24 (m, 2H), 7.09 (d, *J* = 8.1 Hz, 2H), 2.44 (td, *J* = 9.1, 6.5 Hz, 1H), 2.00 – 1.83 (m, 1H), 1.35 (dd, *J* = 2.9, 1.8 Hz, 27H), 1.09 (d, *J* = 11.4 Hz, 12H), 0.91 – 0.82 (m, 2H).

^{13}C NMR (75 MHz, $CDCl_3$) δ 149.8, 147.2, 134.8, 126.9, 125.3, 83.6, 83.1, 46.2, 32.0, 24.9, 24.9, 24.6, 24.2, 16.2.

^{11}B NMR (96 MHz, $CDCl_3$) δ 30.0.

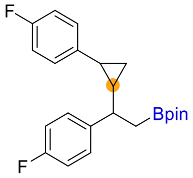

2i, 57%

2-(2-(4-Fluorophenyl)-2-(2-(4-fluorophenyl)cyclopropyl)ethyl)-4,4,5,5-tetramethyl-1,3,2-dioxaborolane (2i)

The title compound was prepared from 1-fluoro-4-vinylbenzene (0.2 mmol), according to general procedure. The crude residue was purified by flash chromatography (*n*-pentane/EA = 100:1, Rf = 0.2) to give the product as a colorless oil (22 mg, 39%).

^{1}H NMR (300 MHz, $CDCl_3$) δ 7.25 – 7.17 (m, 2H), 7.07 – 6.89 (m, 6H), 2.38 (td, *J* = 9.3, 6.1 Hz, 1H), 1.92 – 1.74 (m, 1H), 1.28 (d, *J* = 4.8 Hz, 3H), 1.08 (s, 6H), 1.05 (s, 6H), 0.83 – 0.74 (m, 2H).

^{13}C NMR (75 MHz, $CDCl_3$) δ 161.3 (d, *J* = 243.1 Hz), 161.0 (d, *J* = 243.1 Hz), 142.0 (d, *J* = 3.1 Hz), 138.9 (d, *J* = 3.0 Hz), 128.7 (d, *J* = 7.8 Hz), 127.5 (d, *J* = 7.8 Hz), 114.9 (d, *J* = 21.1 Hz), 114.8 (d, *J* = 21.1 Hz), 83.1, 45.2, 31.5, 24.7, 24.5, 23.1, 15.4.

^{11}B NMR (96 MHz, $CDCl_3$) δ 32.8.

^{19}F NMR (282 MHz, $CDCl_3$) δ -117.5, -118.2.

HRMS (EI): calcd for $[M]^+$ $C_{23}H_{27}BF_2O_2$ 384.2067, found: 384.2070.

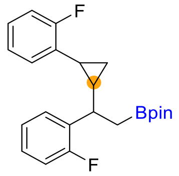

2j, 67%

2-(2-(2-Fluorophenyl)-2-(2-(2-fluorophenyl)cyclopropyl)ethyl)-4,4,5,5-tetramethyl-1,3,2-dioxaborolane (2j)

The title compound was prepared from 1-fluoro-2-vinylbenzene (0.2 mmol), according to general procedure. The crude residue was purified by flash chromatography (*n*-pentane/EA = 100:1, Rf = 0.2) to give the product as a colorless oil (26 mg, 67%).

^{1}H NMR (300 MHz, $CDCl_3$) δ 7.35 – 7.29 (m, 1H), 7.19 – 7.08 (m, 3H), 6.99 (ddt, *J* = 7.9, 6.7, 2.1 Hz, 3H), 6.95 – 6.89 (m, 1H), 2.76 (td, *J* = 9.5, 6.3 Hz, 1H), 2.11 – 2.08 (m, 1H), 1.48 – 1.34 (m, 3H), 1.06 (s, 6H), 1.02 (s, 6H), 0.93 – 0.81 (m, 2H).

^{13}C NMR (75 MHz, $CDCl_3$) δ 161.7 (d, *J* = 245.3 Hz), 160.6 (d, *J* = 244.6 Hz), 133.1 (d, *J* = 15.1 Hz), 130.2 (d, *J* = 14.5 Hz), 128.8 (d, *J* = 5.3 Hz), 127.4 (d, *J* = 8.1 Hz), 126.6 (d, *J* = 7.5 Hz), 126.5 (d, *J* = 4.5 Hz), 123.8 (d, *J* = 3.8 Hz), 123.8 (d, *J* = 3.7 Hz), 115.2 (d, *J* = 23.1 Hz), 114.9 (d, *J* = 22.2 Hz), 83.0, 39.3, 29.5, 24.7, 24.4, 17.1 (d, *J* = 5.0 Hz).14.2.

^{11}B NMR (96 MHz, $CDCl_3$) δ 33.0.

^{19}F NMR (282 MHz, $CDCl_3$) δ -117.74 (dt, *J* = 11.3, 6.5 Hz), -119.11 (ddd, *J* = 10.3, 7.4, 5.2 Hz).

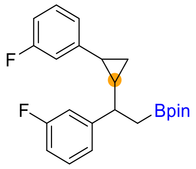

2k, 61%

2-(2-(3-Fluorophenyl)-2-(2-(3-fluorophenyl)cyclopropyl)ethyl)-4,4,5,5-tetramethyl-1,3,2-dioxaborolane (2k)

The title compound was prepared from 1-fluoro-3-vinylbenzene (0.2 mmol), according to general procedure. The crude residue was purified by flash chromatography (*n*-pentane/EA = 100:1, Rf = 0.2) to give the product as a colorless oil (23 mg, 61%).

^{1}H NMR (300 MHz, $CDCl_3$) δ 7.26 – 7.11 (m, 2H), 7.06 – 6.96 (m, 2H), 6.92 – 6.73 (m, 4H), 2.39 (td, *J* = 9.2, 6.1 Hz, 1H), 1.96 – 1.82 (m, 1H), 1.38 – 1.28 (m, 3H), 1.10 (s, 6H), 1.06 (s, 6H), 0.89 – 0.81 (m, 2H).

^{13}C NMR (75 MHz, $CDCl_3$) δ 163.0 (d, *J* = 244.6 Hz), 162.8 (d, *J* = 245.3 Hz), 149.0 (d, *J* = 6.6 Hz), 146.2 (d, *J* = 7.4 Hz), 129.6 (d, *J* = 7.8 Hz), 129.6 (d, *J* = 8.3 Hz), 123.0 (d, *J* = 2.8 Hz), 121.7 (d, *J* = 2.7 Hz), 114.3 (d, *J* = 21.0 Hz), 112.9 (d, *J* = 21.1 Hz), 112.7 (d, *J* = 21.9 Hz), 112.2 (d, *J* = 21.1 Hz), 83.2, 45.8, 31.6, 24.8, 24.6, 23.7, 16.0.

^{11}B NMR (96 MHz, $CDCl_3$) δ 32.9.

^{19}F NMR (282 MHz, $CDCl_3$) δ -113.80 – -113.86 (m), -113.99 – -114.13 (m).

HRMS (**EI**): calcd for $[M]^+$ $C_{23}H_{27}BF_2O_2$ 384.2067, found: 384.2076.

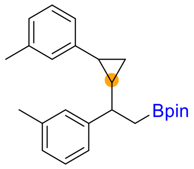

2l, 64%

4,4,5,5-Tetramethyl-2-(2-(*m*-tolyl)-2-(2-(*m*-tolyl)cyclopropyl)ethyl)-1,3,2-dioxaborolane (2l)

The title compound was prepared from 1-fluoro-3-vinylbenzene (0.2 mmol), according to general procedure. The crude residue was purified by flash chromatography (*n*-pentane/EA = 100:1, Rf = 0.2) to give the product as a colorless oil (24 mg, 64%).

^{1}H NMR (300 MHz, $CDCl_3$) δ 7.20 – 7.05 (m, 4H), 7.02 – 6.86 (m, 4H), 2.37 – 2.26 (m, 1H), 2.32 (d, *J* = 7.9 Hz, 6H), 1.84 (dt, *J* = 8.6, 5.0 Hz, 1H), 1.37 – 1.26 (m, 3H), 1.08 (s, 6H), 1.05 (s, 6H), 0.87 – 0.78 (m, 2H).

^{13}C NMR (75 MHz, $CDCl_3$) δ 146.4, 143.6, 137.7, 137.5, 128.2, 128.1, 128.0, 126.8, 126.7, 126.0, 124.4, 123.0, 82.9, 46.2, 31.7, 24.7, 24.5, 23.8, 21.5, 21.4, 15.8.

^{11}B NMR (96 MHz, $CDCl_3$) δ 33.3.

HRMS (**EI**): calcd for $[M]^+$ $C_{25}H_{33}BO_2$ 376.2568, found: 376.2573.

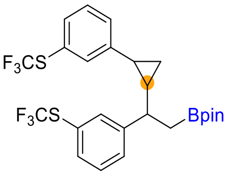

2m, 73%

4,4,5,5-Tetramethyl-2-(2-(3-((trifluoromethyl)thio)phenyl)-2-(2-(3-((trifluoromethyl)thio)phenyl)cyclopropyl)ethyl)-1,3,2-dioxaborolane (2m)

The title compound was prepared from (trifluoromethyl)(3-vinylphenyl)sulfane (0.2 mmol), according to general procedure. The crude residue was purified by flash chromatography (*n*-pentane/EA = 30:1, Rf = 0.2) to give the product as a colorless oil (40 mg, 73%).

^{1}H NMR (300 MHz, $CDCl_3$) δ 7.56 – 7.29 (m, 7H), 7.19 – 7.09 (m, 1H), 2.45 (td, *J* = 9.0, 6.3 Hz, 1H), 1.95 – 1.87 (m, 1H), 1.48 – 1.38 (m, 3H), 1.05 (d, *J* = 12.7 Hz, 12H), 0.89 – 0.85 (m, 2H).

^{13}C NMR (75 MHz, $CDCl_3$) δ 147.8, 145.0, 135.5, 134.0, 133.9, 133.3, 129.9, 129.3, 128.4, 128.5 (q, *J* = 309.4 Hz), 128.3 (q, *J* = 309.5 Hz), 124.4, 83.2, 45.6, 31.5, 24.7, 24.5, 23.5, 15.9.

^{11}B NMR (96 MHz, $CDCl_3$) δ 33.2.

^{19}F NMR (282 MHz, $CDCl_3$) δ -42.59, -42.61.

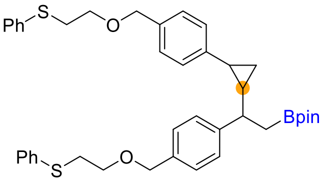

2n,43%

4,4,5,5-Tetramethyl-2-(2-(4-((2-(phenylthio)ethoxy)methyl)phenyl)-2-(2-(4-((2-(phenylthio)ethoxy)methyl)phenyl)cyclopropyl)ethyl)-1,3,2-dioxaborolane (2n)

The title compound was prepared from phenyl(2-((4-vinylbenzyl)oxy)ethyl)sulfane (0.2 mmol), according to general procedure. The crude residue was purified by flash chromatography (*n*-pentane/EA = 7:1, Rf = 0.2) to give the product as a colorless oil (29 mg, 43%).

^{1}H NMR (300 MHz, $CDCl_3$) δ 7.32 – 7.24 (m, 4H), 7.22 – 7.15 (m, 8H), 7.14 – 7.09 (m, 4H), 6.97 (d, *J* = 8.1 Hz, 2H), 4.41 (d, *J* = 8.8 Hz, 4H), 3.56 (td, *J* = 6.9, 3.1 Hz, 4H), 3.07 (s, 4H), 2.32 (td, *J* = 9.2, 6.3 Hz, 1H), 1.79 (dt, *J* = 8.7, 4.9 Hz, 1H), 1.32 – 1.21 (m, 3H), 0.97 (d, *J* = 10.0 Hz, 12H), 0.79 – 0.70 (m, 2H).

^{13}C NMR (75 MHz, $CDCl_3$) δ 146.0, 143.2, 135.6, 134.9, 129.4, 128.9, 127.9, 127.7, 127.5, 126.1, 126.1, 83.5, 83.0, 72.9, 45.8, 33.3, 31.8, 24.8, 24.6, 23.6, 15.9.

^{11}B NMR (96 MHz, $CDCl_3$) δ 30.1.

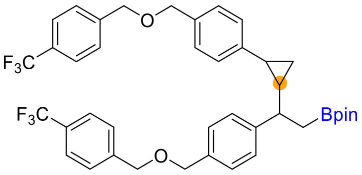

2o, 54%

4,4,5,5-Tetramethyl-2-(2-(4-(((4-(trifluoromethyl)benzyl)oxy)methyl)phenyl)-2-(2-(4-(((4-(trifluoromethyl)benzyl)oxy)methyl)phenyl)cyclopropyl)ethyl)-1,3,2-dioxaborolane (2o)

The title compound was prepared from 1-(trifluoromethyl)-4-(((4-vinylbenzyl)oxy)methyl)benzene (0.2 mmol), according to general procedure. The crude residue was purified by flash chromatography (*n*-pentane/EA = 20:1, Rf = 0.3) to give the product as a colorless oil (39 mg, 54%).

^{1}H NMR (300 MHz, $CDCl_3$) δ 7.64 (d, *J* = 7.1 Hz, 4H), 7.50 (d, *J* = 7.6 Hz, 4H), 7.35 – 7.24 (m, 6H), 7.12 (d, *J* = 8.1 Hz, 2H), 4.64 – 4.54 (m, 8H), 2.46 (td, *J* = 9.1, 6.2 Hz, 1H), 1.93 (dt, *J* = 9.0, 4.9 Hz, 1H), 1.46 – 1.34 (m, 3H), 1.09 (d, *J* = 9.6 Hz, 12H), 0.93 – 0.80 (m, 2H).

^{13}C NMR (75 MHz, $CDCl_3$) δ 146.2, 143.4, 142.6, 135.5, 134.8, 129.7 (q, *J* = 30.2 Hz), 128.0, 127.9, 127.6, 127.6, 127.6, 126.1, 125.28 (q, *J* = 3.7 Hz), 124.2 (q, *J* = 272.0 Hz), 83.1, 72.4, 72.4, 71.0, 45.8, 31.9, 24.8, 24.6, 23.6, 15.9.

^{11}B NMR (96 MHz, $CDCl_3$) δ 31.5.

^{19}F NMR (282 MHz, $CDCl_3$) δ -62.46.

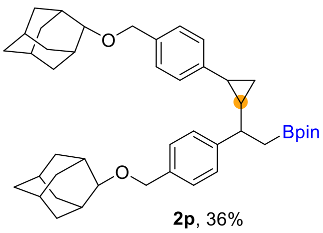

2p, 36%

2-(2-(4-((((1*r*,3*r*,5*r*,7*r*)-Adamantan-2-yl)oxy)methyl)phenyl)-2-(2-(4-((((1*r*,3*r*,5*r*,7*r*)-adamantan-2-yl)oxy)methyl)phenyl)cyclopropyl)ethyl)-4,4,5,5-tetramethyl-1,3,2-dioxaborolane (2p)

The title compound was prepared from (1*r*,3*r*,5*r*,7*r*)-2-((4-vinylbenzyl)oxy)adamantane (0.2 mmol), according to general procedure. The crude residue was purified by flash chromatography (*n*-pentane/EA = 20:1, Rf = 0.3) to give the product as a colorless oil (24 mg, 36%).

^{1}H NMR (300 MHz, $CDCl_3$) δ 7.24 – 7.13 (m, 6H), 6.97 (d, *J* = 8.1 Hz, 2H), 4.43 (d, *J* = 9.2 Hz, 4H), 3.45 (t, *J* = 2.9 Hz, 2H), 2.31 (td, *J* = 9.2, 6.3 Hz, 1H), 2.20 – 1.84 (m, 9H), 1.81 – 1.51 (m, 19H), 1.30 – 1.22 (m, 3H), 0.99 (d, *J* = 9.6 Hz, 12H), 0.79 – 0.69 (m, 2H).
^{13}C NMR (75 MHz, $CDCl_3$) δ 145.4, 142.6, 137.1, 136.5, 127.4, 127.3, 125.9, 83.0, 80.9, 80.8, 69.1, 45.8, 37.6, 36.6, 31.9, 31.7, 31.7, 27.5, 24.8, 24.6, 23.6, 15.8.

^{11}B NMR (96 MHz, $CDCl_3$) δ 33.6.

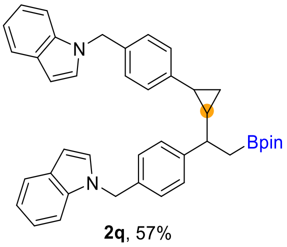

2q, 57%

1-(4-(2-(1-(4-((1*H*-Indol-1-yl)methyl)phenyl)-2-(4,4,5,5-tetramethyl-1,3,2-dioxaborolan-2-yl)ethyl)cyclopropyl)benzyl)-1*H*-indole (2q)

The title compound was prepared from 1-(4-vinylbenzyl)-1*H*-indole (0.2 mmol), according to general procedure. The crude residue was purified by flash chromatography (*n*-pentane/EA = 20:1, Rf = 0.2) to give the product as a colorless oil (35 mg, 57%).

^{1}H NMR (300 MHz, $CDCl_3$) δ 7.61 – 7.46 (m, 2H), 7.20 – 6.95 (m, 12H), 6.89 (s, 4H), 6.44 (d, *J* = 3.1 Hz, 2H), 5.17 (d, *J* = 6.4 Hz, 4H), 2.25 (td, *J* = 9.3, 6.2 Hz, 1H), 1.73 (dt, *J* = 9.2, 4.9 Hz, 1H), 1.22 – 1.16 (m, 3H), 0.91 (d, *J* = 11.3 Hz, 12H), 0.74 – 0.62 (m, 2H).

^{13}C NMR (75 MHz, $CDCl_3$) δ 145.8, 142.9, 136.2, 135.1, 134.4, 128.6, 128.1, 127.7, 126.7, 126.2, 121.5, 121.5, 120.9, 120.8, 119.4, 119.4, 109.7, 109.6, 101.5, 101.4, 82.9, 49.9, 49.8, 45.7, 31.8, 24.7, 24.5, 23.4, 15.8.

^{11}B NMR (96 MHz, $CDCl_3$) δ 31.6.

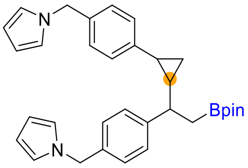

2r, 62%

1-(4-(2-(1-(4-((1*H*-Pyrrol-1-yl)methyl)phenyl)-2-(4,4,5,5-tetramethyl-1,3,2-dioxaborolan-2-yl)ethyl)cyclopropyl)benzyl)-1*H*-pyrrole (2r)

The title compound was prepared from 1-(4-vinylbenzyl)-1*H*-pyrrole (0.2 mmol), according to general procedure. The crude residue was purified by flash chromatography (*n*-pentane/EA = 20:1, Rf = 0.2) to give the product as a colorless oil (31 mg, 62%).

^{1}H NMR (400 MHz, $CDCl_3$) δ 7.21 (d, *J* = 8.1 Hz, 2H), 7.06 (d, *J* = 8.3 Hz, 2H), 7.04 – 6.95 (m, 4H), 6.67 (t, *J* = 2.1 Hz, 4H), 6.17 (t, *J* = 2.1 Hz, 4H), 5.03 (s, 2H), 5.01 (s, 2H), 2.36 (td, *J* = 9.2, 6.1 Hz, 1H), 1.85 (dt, *J* = 9.0, 4.9 Hz, 1H), 1.36 – 1.29 (m, 3H), 1.04 (d, *J* = 13.3 Hz, 12H), 0.85 – 0.75 (m, 2H).

^{13}C NMR (75 MHz, $CDCl_3$) δ 135.7, 135.0, 127.7, 127.0, 126.2, 121.0, 108.3, 108.2, 83.0, 53.1, 53.0, 45.7, 31.8, 25.0, 24.7, 23.4, 15.8.

^{11}B NMR (96 MHz, $CDCl_3$) δ 30.2.

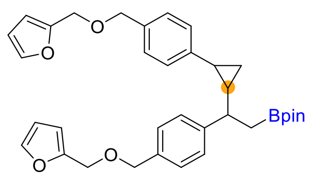

2s, 37%

2-(2-(4-((Furan-2-ylmethoxy)methyl)phenyl)-2-(2-(4-((furan-2-ylmethoxy)methyl)phenyl)cyclopropyl)ethyl)-4,4,5,5-tetramethyl-1,3,2-dioxaborolane (2s)

The title compound was prepared from 2-(((4-vinylbenzyl)oxy)methyl)furan (0.2 mmol), according to general procedure. The crude residue was purified by flash chromatography (*n*-pentane/EA = 20:1, Rf = 0.3) to give the product as a colorless oil (21 mg, 37%).

^{1}H NMR (300 MHz, $CDCl_3$) δ 7.44 (td, *J* = 1.7, 0.9 Hz, 2H), 7.29 – 7.23 (m, 6H), 7.14 – 7.01 (m, 2H), 6.39 – 6.31 (m, 4H), 4.55 (s, 2H), 4.52 (s, 2H), 4.48 (d, *J* = 1.8 Hz, 4H), 2.43 (td, *J* = 9.2, 6.2 Hz, 1H), 1.90 (dt, *J* = 8.9, 5.0 Hz, 1H), 1.40 – 1.30 (m, 3H), 1.08 (d, *J* = 9.9 Hz, 12H), 0.87 – 0.77 (m, 2H).

^{13}C NMR (75 MHz, $CDCl_3$) δ 151.8, 146.0, 143.2, 142.7, 135.4, 134.8, 128.0, 127.9, 127.5, 126.0, 110.2, 109.3, 83.0, 71.8, 71.7, 63.5, 45.7, 31.8, 24.7, 24.6, 23.6, 15.8.

^{11}B NMR (96 MHz, $CDCl_3$) δ 33.3.

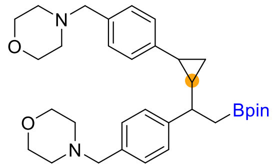

2t, 61%

4-(4-(2-(1-(4-(Morpholinomethyl)phenyl)-2-(4,4,5,5-tetramethyl-1,3,2-dioxaborolan-2-yl)ethyl)cyclopropyl)benzyl)morpholine (2t)

The title compound was prepared from 4-(4-vinylbenzyl)morpholine (0.2 mmol), according to general procedure. The crude residue was purified by flash chromatography (EA/MeOH = 20:1, Rf = 0.2) to give the product as a colorless oil (33 mg, 61%).

^{1}H NMR (400 MHz, $CDCl_3$) δ 7.24 – 7.17 (m, 6H), 7.02 (d, *J* = 8.0 Hz, 2H), 3.72 – 3.68 (m, 8H), 3.50 (d, *J* = 8.0 Hz, 4H), 2.46 (s, 8H), 2.37 (dd, *J* = 9.3, 3.4 Hz, 1H), 1.85 (dt, *J* = 8.9, 4.9 Hz, 1H), 1.40 – 1.28 (m, 3H), 1.03 (d, *J* = 12.5 Hz, 12H), 0.87 – 0.80 (m, 2H).

^{13}C NMR (101 MHz, $CDCl_3$) δ 145.5, 142.8, 129.3, 129.3, 127.4, 125.9, 83.0, 66.8, 63.1, 53.4, 45.8, 31.9, 24.7, 24.6, 23.6, 15.9.

^{11}B NMR (128 MHz, $CDCl_3$) δ 33.3.

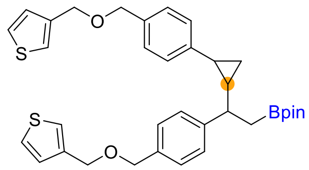

2u, 51%

4,4,5,5-Tetramethyl-2-(2-(4-((thiophen-3-ylmethoxy)methyl)phenyl)-2-(2-(4-((thiophen-3-ylmethoxy)methyl)phenyl)cyclopropyl)ethyl)-1,3,2-dioxaborolane (2u)

The title compound was prepared from 3-(((4-vinylbenzyl)oxy)methyl)thiophene (0.2 mmol), according to general procedure. The crude residue was purified by flash chromatography (*n*-pentane/EA = 30:1, Rf = 0.3) to give the product as a colorless oil (31 mg, 51%).

^{1}H NMR (300 MHz, $CDCl_3$) δ 7.23 – 7.13 (m, 10H), 7.03 – 6.97 (m, 4H), 4.46 – 4.41 (m, 8H), 2.33 (td, *J* = 9.2, 6.3 Hz, 1H), 1.80 (dt, *J* = 8.9, 5.0 Hz, 1H), 1.26 – 1.15 (m, 3H), 0.98 (d, *J* = 9.6 Hz, 12H), 0.79 – 0.70 (m, 2H).
^{13}C NMR (75 MHz, $CDCl_3$) δ 145.9, 143.1, 139.5, 135.7, 135.0, 127.9, 127.8, 127.4, 127.4, 126.0, 125.9, 125.9, 122.7, 83.0, 71.9, 71.8, 67.0, 45.7, 31.8, 24.7, 24.6, 23.6, 15.8.
^{11}B NMR (96 MHz, $CDCl_3$) δ 33.3.

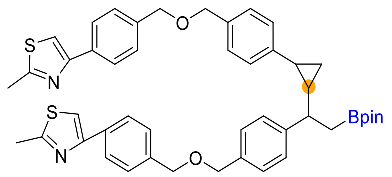

2v, 47%

2-methyl-4-(4-(((4-(2-(1-(4-(((4-(2-methylthiazol-4-yl)benzyl)oxy)methyl)phenyl)-2-(4,4,5,5-tetramethyl-1,3,2-dioxaborolan-2-yl)ethyl)cyclopropyl)benzyl)oxy)methyl)phenyl)thiazole (2v)

The title compound was prepared from 2-methyl-4-(4-(((4-vinylbenzyl)oxy)methyl)phenyl)thiazole (0.2 mmol), according to general procedure. The crude residue was purified by flash chromatography (*n*-pentane/EA = 4:1, Rf = 0.2) to give the product as a colorless oil (37 mg, 47%).

^{1}H NMR (300 MHz, $CDCl_3$) δ 7.89 (dd, *J* = 8.2, 1.7 Hz, 4H), 7.42 (d, *J* = 7.5 Hz, 4H), 7.29 (ddd, *J* = 14.2, 5.2, 2.8 Hz, 8H), 7.10 (d, *J* = 8.1 Hz, 2H), 4.66 – 4.49 (m, 8H), 2.80 (s, 6H), 2.44 (td, *J* = 9.2, 6.2 Hz, 1H), 1.91 (dq, *J* = 8.9, 4.4 Hz, 1H), 1.45 – 1.32 (m, 3H), 1.08 (d, *J* = 9.6 Hz, 12H), 0.90 – 0.79(m, 2H).

^{13}C NMR (75 MHz, $CDCl_3$) δ 166.0, 154.9, 146.0, 143.2, 138.2, 135.8, 135.1, 133.8, 128.2, 128.0, 127.9, 127.5, 126.4, 126.1, 112.2, 83.0, 75.0, 72.0, 71.9, 71.6, 45.8, 31.9, 24.9, 24.8, 24.6, 23.6, 19.3, 15.9.

^{11}B NMR (96 MHz, $CDCl_3$) δ 22.6.

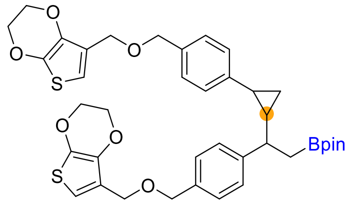

2w, 53%

2-(2-(4-(((2,3-Dihydrothieno[2,3-*b*][1,4]dioxin-7-yl)methoxy)methyl)phenyl)-2-(2-(4-(((2,3-dihydrothieno[2,3-*b*][1,4]dioxin-7-yl)methoxy)methyl)phenyl)cyclopropyl)ethyl)-4,4,5,5-tetramethyl-1,3,2-dioxaborolane (2w)

The title compound was prepared from 7-(((4-vinylbenzyl)oxy)methyl)-2,3-dihydrothieno[2,3-*b*][1,4]dioxine (0.2 mmol), according to general procedure. The crude residue was purified by flash chromatography (*n*-pentane/EA = 5:1, Rf = 0.2) to give the product as a colorless oil (38 mg, 53%).

^{1}H NMR (300 MHz, $CDCl_3$) δ 7.29 – 7.06 (m, 8H), 6.98 (d, *J* = 8.1 Hz, 2H), 6.23 (dd, *J* = 4.6, 1.3 Hz, 2H), 4.46 – 4.42 (m, 8H), 4.12 (qq, *J* = 2.8, 1.3 Hz, 8H), 2.37 – 2.28 (m, 1H), 1.79 (dt, *J* = 8.9, 5.0 Hz, 1H), 1.32 – 1.26 (m, 3H), 0.98 (d, *J* = 9.9 Hz, 12H), 0.77 – 0.70 (m, 2H).

^{13}C NMR (75 MHz, $CDCl_3$) δ 145.9, 143.1, 141.3, 139.7, 135.7, 135.0, 128.0, 127.9, 127.4, 126.0, 113.7, 113.2, 98.9, 83.0, 75.0, 71.5, 71.5, 64.7, 64.6, 62.2, 62.2, 45.8, 31.8, 24.9, 24.8, 24.6, 15.8.
^{11}B NMR (96 MHz, $CDCl_3$) δ 22.6.

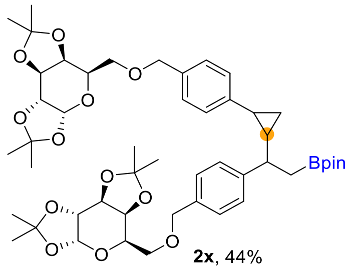

2x, 44%

4,4,5,5-Tetramethyl-2-(2-(4-((((3a*R*,5*R*,5a*S*,8a*S*,8b*R*)-2,2,7,7-tetramethyltetrahydro-5*H*-bis([1,3]dioxolo)[4,5-b:4',5'-*d*]pyran-5-yl)methoxy)methyl)phenyl)-2-(2-(4-((((3a*R*,5*R*,5a*S*,8a*S*,8b*R*)-2,2,7,7-tetramethyltetrahydro-5*H*-bis([1,3]dioxolo)[4,5-b:4',5'-*d*]pyran-5-yl)methoxy)methyl)phenyl)cyclopropyl)ethyl)-1,3,2-dioxaborolane (2x)

The title compound was prepared from (3a*R*,5*R*,5a*S*,8a*S*,8b*R*)-2,2,7,7-tetramethyl-5-(((4-vinylbenzyl)oxy)methyl)tetrahydro-5*H*-bis([1,3]dioxolo)[4,5-b:4',5'-*d*]pyran (0.2 mmol), according to general procedure. The crude residue was purified by flash chromatography (*n*-pentane/EA = 1:1, Rf = 0.2) to give the product as a colorless oil (39 mg, 44%).

^{1}**H NMR** (300 MHz, $CDCl_3$) δ 7.23 – 7.07 (m, 6H), 6.96 (d, *J* = 8.2 Hz, 2H), 4.59 – 4.41 (m, 6H), 4.39 – 4.34 (m, 2H), 4.19 – 4.13 (m, 2H), 3.89 – 3.83 (m, 1H), 3.82 (d, *J* = 2.0 Hz, 1H), 3.66 (ddd, *J* = 13.0, 1.9, 0.7 Hz, 2H), 3.57 – 3.41 (m, 4H), 2.31 (td, *J* = 9.2, 6.3 Hz, 1H), 1.78 (dt, *J* = 9.0, 5.0 Hz, 1H), 1.37 (d, *J* = 4.5 Hz, 3H), 1.18 (d, *J* = 6.4 Hz, 24H), 0.97 (d, *J* = 11.2 Hz, 12H), 0.78 – 0.63 (m, 2H).
^{13}**C NMR** (75 MHz, $CDCl_3$) δ 145.6, 142.9, 135.7, 135.0, 127.6, 127.4, 127.3, 125.8, 108.9, 108.5, 102.7, 102.7, 82.9, 74.9, 73.6, 73.5, 71.4, 71.4, 71.3, 71.0, 70.2, 70.2, 70.1, 60.9, 45.7, 31.7, 26.5, 25.8, 25.8, 25.4, 24.8, 24.7, 24.6, 24.5, 24.5, 24.0, 23.6, 15.8.

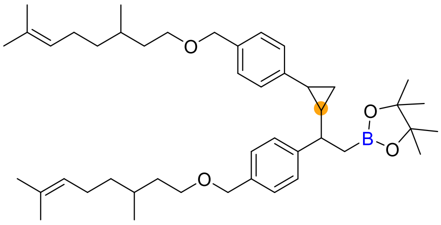

2y, 43%

2-(2-(4-(((3,7-Dimethyloct-6-en-1-yl)oxy)methyl)phenyl)-2-(2-(4-(((3,7-dimethyloct-6-en-1-yl)oxy)methyl)phenyl)cyclopropyl)ethyl)-4,4,5,5-tetramethyl-1,3,2-dioxaborolane (2y)

The title compound was prepared from 1-(((3,7-dimethyloct-6-en-1-yl)oxy)methyl)-4-vinylbenzene (0.2 mmol), according to general procedure. The crude residue was purified by flash chromatography (*n*-pentane/EA = 30:1, Rf = 0.4) to give the product as a colorless oil (29 mg, 43%).

^{1}**H NMR** (300 MHz, $CDCl_3$) δ 7.25 (s, 4H), 7.22 – 7.19 (m, 2H), 7.05 (d, *J* = 8.1 Hz, 2H), 5.09 (dddd, *J* = 7.1, 5.8, 2.9, 1.4 Hz, 2H), 4.46 (d, *J* = 8.4 Hz, 4H), 3.47 (ddq, *J* = 6.9, 4.7, 2.4 Hz, 4H), 2.39 (td, *J* = 9.2, 6.2 Hz, 1H), 2.01 – 1.84 (m, 5H), 1.68 (s, 6H), 1.60 (s, 6H), 1.49 – 1.20 (m, 11H), 1.14 (t, *J* = 2.3 Hz, 2H), 1.06 (d, *J* = 9.8 Hz, 12H), 0.88 (dd, *J* = 6.6, 1.2 Hz, 6H), 0.84 – 0.74 (m, 2H).
^{13}**C NMR** (75 MHz, $CDCl_3$) δ 145.7, 142.9, 136.3, 135.6, 131.1, 127.7, 127.6, 127.4, 125.9, 124.8, 83.0, 72.8, 68.5, 45.7, 37.2, 36.7, 31.8, 29.6, 29.6, 25.7, 25.5, 24.7, 24.6, 23.6, 19.5, 17.6, 15.8.

^{11}**B NMR** (96 MHz, $CDCl_3$) δ 34.8.

5. Derivatization of 2a.

5.1 Oxidation of **2a.**

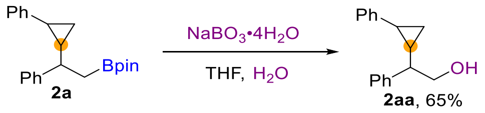

The title compound **2aa** was synthesized according to the following literature[1]: $NaBO_3$•$4H_2O$ (5 equiv.) was added to a solution of boration product **2a** (0.1 mmol) in THF/H_2O (2.5 mL/2.5 mL). The resulting mixture was stirred vigorously for 2 h at room temperature. The reaction mixture was quenched with water and then extracted with ethyl acetate (5 mL). The combined organic layers were washed with brine (15 mL), dried over Na_2SO_4 and concentrated. The crude product was purified by column chromatography on silica gel to afford the corresponding product **2aa** as a colorless oil.

2-Phenyl-2-(2-phenylcyclopropyl)ethan-1-ol (2aa)

^{1}H NMR (400 MHz, $CDCl_3$) δ 7.44 – 7.37 (m, 2H), 7.34 – 7.28 (m, 5H), 7.22 – 7.17 (m, 1H), 7.16 – 7.09 (m, 2H), 4.03 – 3.89 (m, 2H), 2.36 (ddd, *J* = 9.7, 7.5, 6.2 Hz, 1H), 1.95 (dt, *J* = 9.1, 4.9 Hz, 1H), 1.41 – 1.31 (m, 2H), 0.97 (dt, *J* = 8.6, 5.2 Hz, 1H), 0.86 (dt, *J* = 8.7, 5.4 Hz, 1H).

^{13}C NMR (101 MHz, $CDCl_3$) δ 142.8, 141.6, 128.7, 128.4, 128.0, 126.9, 125.7, 125.6, 67.3, 53.2, 25.8, 23.8, 14.1.

5.2 Vinylation of **2a.**

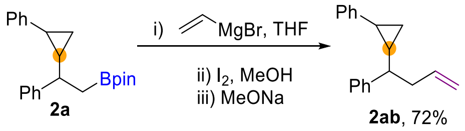

The title compound **2ab** was synthesized according to the following literature[2]: a solution of **2a** (0.1 mmol) in THF (2 mL) was added to an oven-dried round bottom flask containing a stirring bar. Vinylmagnesium bromide (1 M, 0.4 mL, 4.0 equiv.) was added dropwise to the mixture. The mixture was stirred at room temperature for 0.5 h. Then the flask was cooled to -78 ℃. I_2 (2.0 equiv.) in methanol (3.0 mL) was added dropwise. The reaction mixture was allowed to stir 0.5 h at the same temperature, followed by the dropwise addition of a solution of NaOMe (8.0 equiv.) in methanol (3 mL). Then the reaction mixture was warmed to room temperature and stirred for another 1.5 h, diluted with EtOAc (10 mL), and washed sequentially with 10% aqueous solution of $Na_2S_2O_3$ (5 mL). Then, the mixture was extracted with EtOAc (10 mL), dried over Na_2SO_4, filtered, and concentrated under reduced pressure. The crude product was purified by column chromatography on silica gel to afford the corresponding product **2ab** as a colorless oil.

(2-(1-Phenylbut-3-en-1-yl)cyclopropyl)benzene (2ab)

^{1}H NMR (300 MHz, $CDCl_3$) δ 7.28 – 7.01 (m, 10H), 5.66 (ddt, *J* = 17.2, 10.1, 7.1 Hz, 1H), 4.97 – 4.81 (m, 2H), 2.56 – 2.42 (m, 2H), 2.17 – 2.03 (m, 1H), 1.75 (dt, *J* = 9.1, 4.9 Hz, 1H), 1.26 (d, *J* = 3.5 Hz, 1H), 0.85 – 0.71 (m, 2H).
^{13}C NMR (75 MHz, $CDCl_3$) δ 144.5, 143.3, 136.8, 128.3, 128.3, 127.6, 126.2, 125.7, 125.4, 116.0, 51.0, 41.0, 29.5, 24.3, 14.9.

5.3 BF_3K preparation.

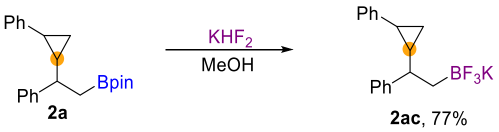

The title compound **2ac** was prepared according to the following literature[3]: a solution of **2a** (0.1 mmol) and KHF_2 (7 equiv.) in MeOH (2 mL) was added to an oven-dried round bottom flask containing a stirring bar. The reaction mixture was stirred vigorously for 3 h at room temperature. The resulting slurry was stirred concentrated, then placed under high vacuum. The dried solids were triturated with hot acetone and filtered to remove inorganic salts. The resulting filtrate was concentrated to a minimal volume and wash with *n*-pentane (5 x 2 mL) was added to afford **2ac** as a white solid. (There is still some **2a** that has not reacted completely, and acetone cannot wash it off completely. Therefore, there is still about 23% of the **2a**)

Trifluoro(2-phenyl-2-(2-phenylcyclopropyl)ethyl)-λ^4-borane, potassium salt (2ac)

^{1}H NMR (400 MHz, $CDCl_3$) δ 7.25 – 7.22 (m, 6H), 7.12 – 7.08 (m, 4H), 2.38 (td, *J* = 9.3, 6.1 Hz, 1H), 2.20 – 2.09 (m, 2H), 1.92 – 1.78 (m, 2H), 0.82 – 0.80 (m, 2H).

^{13}C NMR (101 MHz, $CDCl_3$) δ 146.4, 143.6, 128.4, 128.2, 128.2, 127.5, 126.0, 125.7, 46.2, 31.8, 24.8, 24.6, 23.9, 15.8.

5.4 Suzuki–Miyaura Reaction of **2ad.**

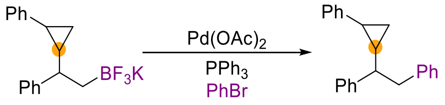

The title compound **2ad** was prepared according to a modified methods[3]: A 4 mL screw-cap vial with a magnetic stir bar was charged was charged with $Pd(OAc)_2$ (1 mol %), RuPhos (1 mol %), K_2CO_3 (3 equiv.), and **2ac** (0.12 mmol). The vial was closed with a Teflon septum and cap and connected to the atmosphere via a needle. Toluene (0.5 mL) was added, followed by H_2O (50 μL), and bromobenzene (1 equiv.). The vial was quickly exchanged for a Teflon-lined screw cap and the reaction was stirred at 80 ℃ for 24 h. At this time, the reaction was allowed to cool to room temperature and the reaction was then quenched upon addition of water (5 mL) and the mixture was extracted with EtOAc (3 mL). The combined organic was dried using Na2SO4 and then concentrated in vacuo. The crude product was purified by column chromatography on silica gel (*n*-pentane/EA = 100/1) to afford the corresponding product **2ad** as a colorless oil.

(1-(2-Phenylcyclopropyl)ethane-1,2-diyl)dibenzene (2ad)

^{1}H NMR (300 MHz, $CDCl_3$) δ 7.28 – 7.02 (m, 12H), 6.91 – 6.88 (m, 1H), 6.86 – 6.77 (m, 2H), 3.07 – 2.90 (m, 2H), 2.28 (dt, *J* = 9.4, 7.3 Hz, 1H), 1.50 (dt, *J* = 9.0, 4.9 Hz, 1H), 1.29 – 1.24 (m, 1H), 0.72 (ddt, *J* = 29.6, 8.6, 5.2 Hz, 2H).

^{13}C NMR (75 MHz, $CDCl_3$) δ 144.7, 143.2, 140.3, 129.3, 128.3, 128.1, 128.1, 127.7, 126.3, 125.8, 125.7, 125.3, 53.1, 43.5, 29.5, 24.6, 15.0.

6. Mechanism studies.

6.1 ^{13}C labeling experiment.

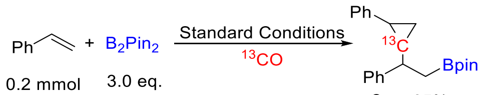

A dried vial (4 mL) was charged with $Cu(OTf)_2$ (12.5 mol%), DPPB (12.5 mol%), B_2pin_2 (3.0 equiv.), NaO^{t}Bu (3.0 equiv.), and a stirring bar. The vial was sealed by PTFE/white rubber septum (Wheaton 13 mm Septa) and phenolic cap and connected with atmosphere with a needle. The vial was evacuated under vacuum and recharged with argon for three times. Then, styrene (0.2 mmol) and toluene (1.0 mL) were added under argon by using a syringe. The vial (or several vials) was placed in an alloy plate and transferred into a 300 mL autoclave of the 4560 series from Parr Instruments. The autoclave was charged with ^{13}CO (1 bar) and CO (9 bar) after flushing two times with N_2 and two times with CO. The autoclave was then placed into an aluminum block on a magnetic stirrer. The reaction was performed for 20 h at 90 °C. After the reaction was complete, the autoclave was cooled down with ice water to room temperature and the pressure was released carefully. After that the residue was directly purified by column chromatography to afford the corresponding products **2ae**.

^{1}H NMR (300 MHz, $CDCl_3$) δ 7.20 – 6.99 (m, 10H), 2.31 (ddt, *J* = 12.6, 6.2, 3.2 Hz, 1H), 1.81 (dt, *J* = 8.8, 5.0 Hz, 1H), 1.34 – 1.25 (m, 3H), 0.98 (d, *J* = 10.1 Hz, 12H), 0.79 – 0.71 (m, 2H).
^{13}C NMR (75 MHz, $CDCl_3$) δ 146.4, 143.6, 128.2, 128.1, 127.4, 126.0, 126.0, 125.3, 83.0, 46.1 (t, *J* = 21.13 Hz), 31.8, 24.7, 24.5, 23.8 (t, *J* = 6.0 Hz), 15.8 (t, *J* = 6.8 Hz), 15.7.
^{11}B NMR (96 MHz, $CDCl_3$) δ 33.6.
(By the result of this reaction, we confirm that the carbon at the β-position of the benzene ring comes from this carbon monoxide.)

6.2 Deuterated Styrene as starting material.

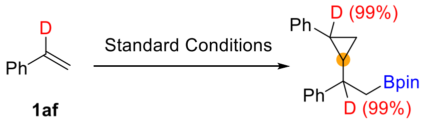

A dried vial (4 mL) was charged with $Cu(OTf)_2$ (12.5 mol%), DPPB (12.5 mol%), B_2pin_2 (3.0 equiv.), NaO^{t}Bu (3.0 equiv.), and a stirring bar. The vial was sealed by PTFE/white rubber septum (Wheaton 13 mm Septa) and phenolic cap and connected with atmosphere with a needle. The vial was evacuated under vacuum and recharged with argon for three times. Then, **1af** (0.2 mmol) and toluene (1.0 mL) were added under argon by using a syringe. The vial (or several vials) was placed in an alloy plate and transferred into a 300 mL autoclave of the 4560 series from Parr Instruments. The autoclave was charged with CO (10 bar) after flushing two times with N_2 and two times with CO. The autoclave was then placed into an aluminum block on a magnetic stirrer. The reaction was performed for 20 h at 90 °C. After the reaction was complete, the autoclave was cooled down with ice water to room temperature and the pressure was released carefully. After that the residue was directly purified by column chromatography to afford the corresponding products **2af**.

^{1}H NMR (300 MHz, $CDCl_3$) δ 7.28 – 7.23 (m, 5H), 7.21 – 7.03 (m, 5H), 1.36 (dd, *J* = 6.8, 2.3 Hz, 3H), 1.06 (s, 6H), 1.02 (s, 6H), 0.81 (dd, *J* = 9.0, 7.1 Hz, 2H).

^{11}B NMR (96 MHz, $CDCl_3$) δ 32.9.

(Compared with the 1H NMR of standard product **2a**, the hydrogen in the α-position of styrene does not undergo intramolecular transfer.)

6.3 Deuterated solvent.

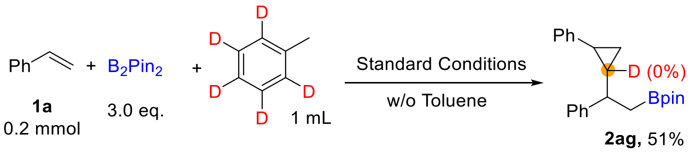

A dried vial (4 mL) was charged with $Cu(OTf)_2$ (12.5 mol%), DPPB (12.5 mol%), B_2pin_2 (3.0 equiv.), NaO^{t}Bu (3.0 equiv.), and a stirring bar. The vial was sealed by PTFE/white rubber septum (Wheaton 13 mm Septa) and phenolic cap and connected with atmosphere with a needle. The vial was evacuated under vacuum and recharged with argon for three times. Then, **1a** (0.2 mmol) and toluene-D_5 (1.0 mL) were added under argon by using a syringe. The vial (or several vials) was placed in an alloy plate and transferred into a 300 mL autoclave of the 4560 series from Parr Instruments. The autoclave was charged with CO (10 bar) after flushing two times

with N_2 and two times with CO. The autoclave was then placed into an aluminum block on a magnetic stirrer. The reaction was performed for 20 h at 90 °C. After the reaction was complete, the autoclave was cooled down with ice water to room temperature and the pressure was released carefully. After that the residue was directly purified by column chromatography to afford the corresponding products **2ag**.

^{1}H NMR (300 MHz, $CDCl_3$) δ 7.22 – 7.16 (m, 5H), 7.16 – 6.99 (m, 5H), 2.32 (td, *J* = 9.3, 6.2 Hz, 1H), 1.97 – 1.72 (m, 1H), 1.33 – 1.20 (m, 3H), 0.98 (d, *J* = 10.1 Hz, 12H), 0.81 – 0.71 (m, 2H).
^{13}C NMR (75 MHz, $CDCl_3$) δ 146.4, 143.6, 128.2, 128.1, 127.4, 126.0, 126.0, 125.2, 83.0, 46.1, 31.8, 24.7, 24.5, 23.8, 15.8.
^{11}B NMR (96 MHz, $CDCl_3$) δ 33.2.
(No peaks of deuterium atoms were found in ^{2}H NMR spectra of **2ag**, proving that the hydrogen at the β-position of the benzene ring does not come from toluene.)

221201.397.11.fid — Huiqing Geng, GHQ-L-230 // 2H chloroform/no lock — 46.07MHz

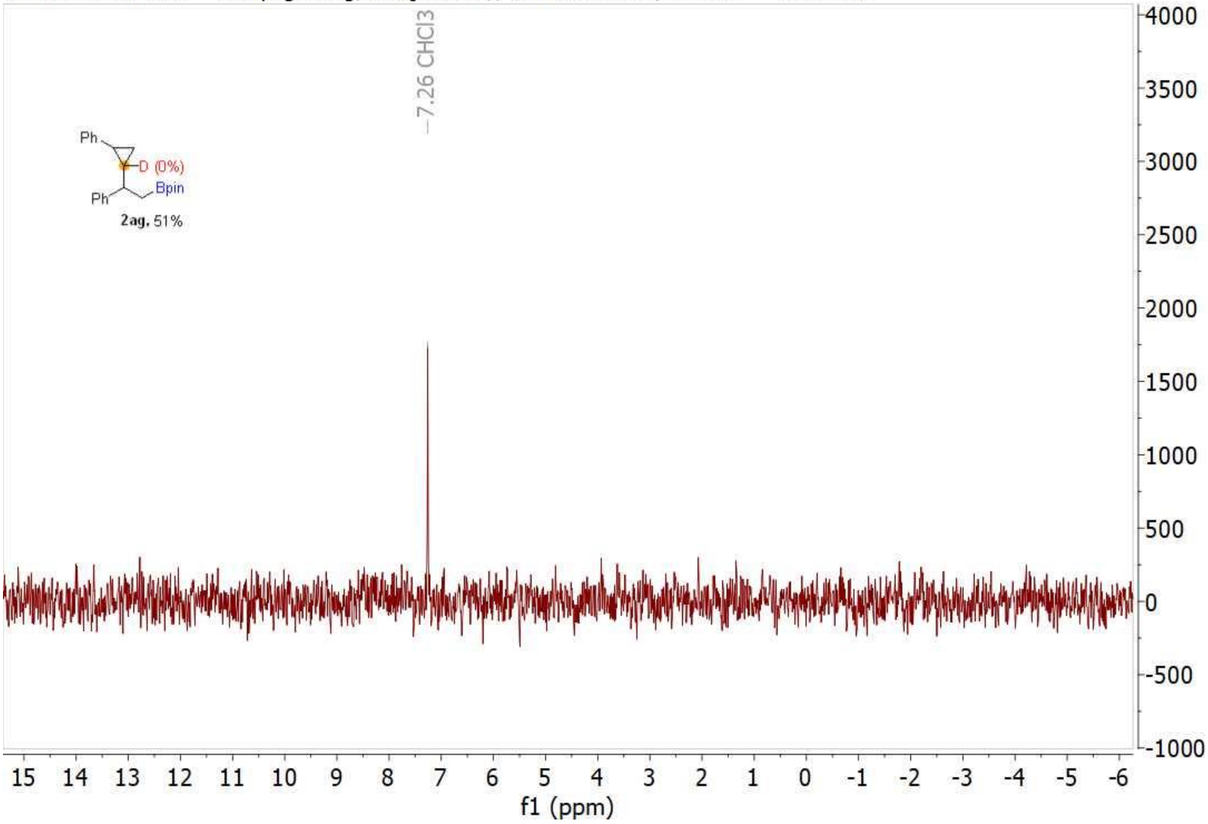

6.4 Additional deuterium water.

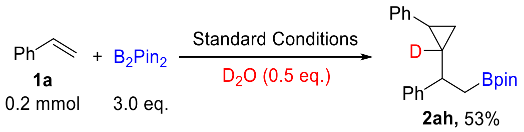

A dried vial (4 mL) was charged with $Cu(OTf)_2$ (12.5 mol%), DPPB (12.5 mol%), B_2pin_2 (3.0 equiv.), NaO^tBu (3.0 equiv.), and a stirring bar. The vial was sealed by PTFE/white rubber septum (Wheaton 13 mm Septa) and phenolic cap and connected with atmosphere with a needle. The vial was evacuated under vacuum and recharged with argon for three times. Then, **1a** (0.2 mmol) and toluene (1.0 mL) and D_2O (1.8 μL, 0.5 equiv.) were added under argon by using a syringe. The vial (or several vials) was placed in an alloy plate and transferred into a 300 mL autoclave of the 4560 series from Parr Instruments. The autoclave was charged with CO (10 bar) after flushing two times with N_2 and two times with CO. The autoclave was then placed into an aluminum block on a magnetic stirrer. The reaction was performed for 20 h at 90 °C. After the reaction was complete, the autoclave was cooled down with ice water to room temperature and the pressure was released carefully. After that the residue was directly purified by column chromatography to afford the corresponding products **2ah**.

(The hydrogen at *β*-position overlaps with other alkyl region hydrogens in the ^1H NMR spectra, so the specific deuteration content cannot be seen qualitatively. In this control reaction, we added about Volumetric ratio (500:1) of solvent to D_2O and obtain 53% of the product **2ah**. In the ^2H NMR spectra, we can see that the deuterium peak. This result allowed us to qualitatively determine that the hydrogen at the *β*-position of the benzene ring comes from the trace amount of water present in the solvent.)

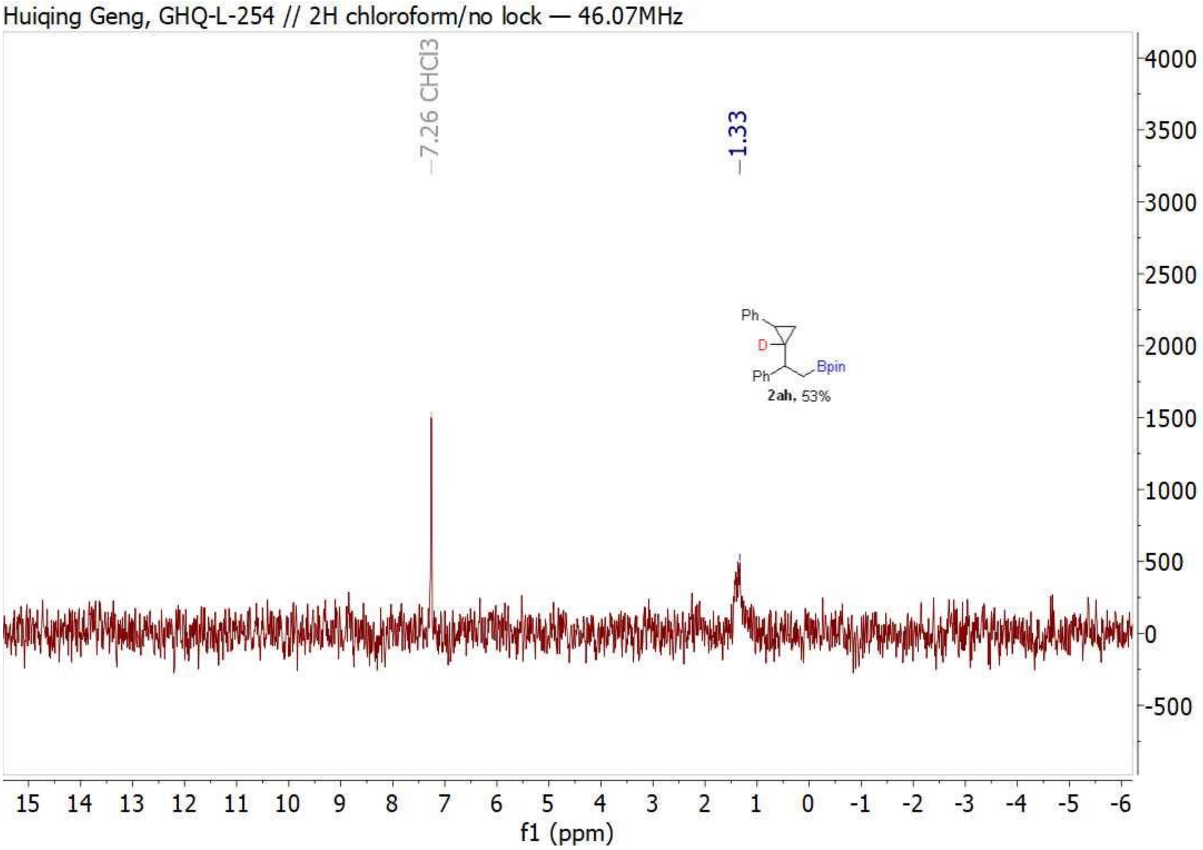

7. References.

(1) Lee, J.-E.; Yun, J. Catalytic Asymmetric Boration of Acyclic α, β-Unsaturated Esters and Nitriles. *Angew. Chem. Int. Ed*. **2008**, *47*, 145-147.
(2) Sun S.-Z.; Martin R. Nickel-Catalyzed Umpolung Arylation of Ambiphilic α-Bromoalkyl Boronic Esters. *Angew. Chem. Int. Ed.* **2018**, *57*, 3684-3687
(3) Fang, G.-H.; Yan, Z.-J.; Deng, M.-Z. Palladium-Catalyzed Cross-Coupling of Stereospecific Potassium Cyclopropyl Trifluoroborates with Aryl Bromides. *Org. Lett*. **2004**, *6*, 357–360.
(4) Dreher, S. D.; Lim, S.-E.; Sandrock, D. L.; Molander, G. A. Suzuki–Miyaura Cross-Coupling Reactions of Primary Alkyltrifluoroborates with Aryl Chlorides. *J. Org. Chem*. **2009**, *74*, 3626–3631.

8. Spectra Copies.

220609.f309.10.fid — Geng/ GHQ-L — PROTON CDCl3 {C:\Bruker\TopSpin3.6.2} 2206 9 — 300.20MHz

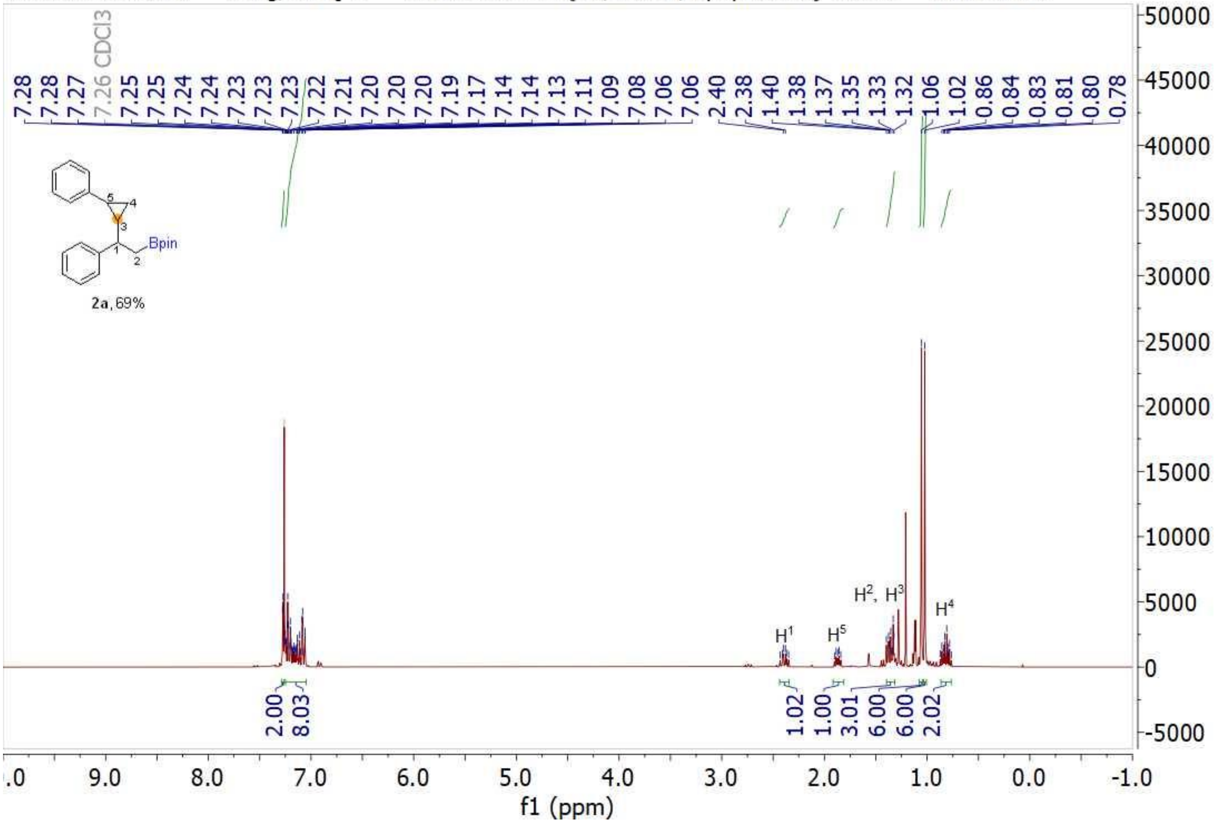

220609.f309.11.fid — Geng/ GHQ-L — C13CPD CDCl3 {C:\Bruker\TopSpin3.6.2} 2206 9 — 75.49MHz

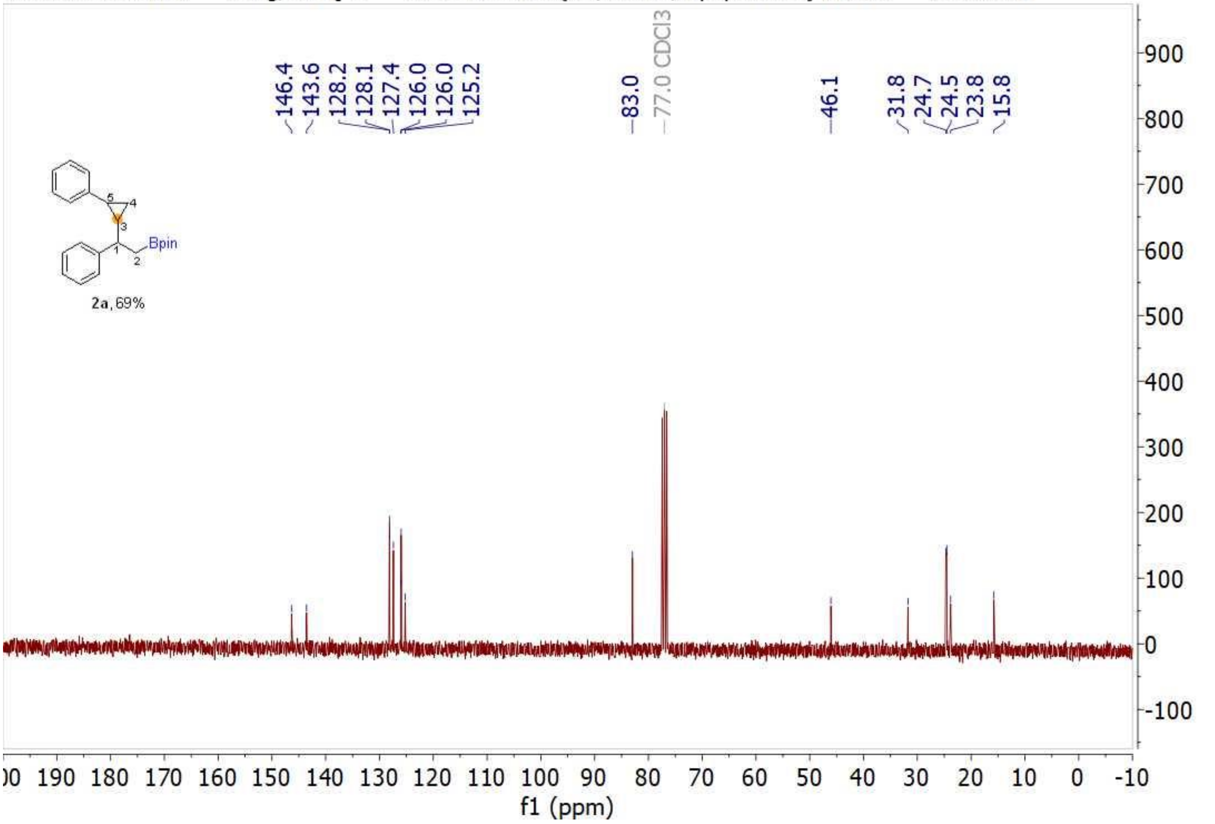

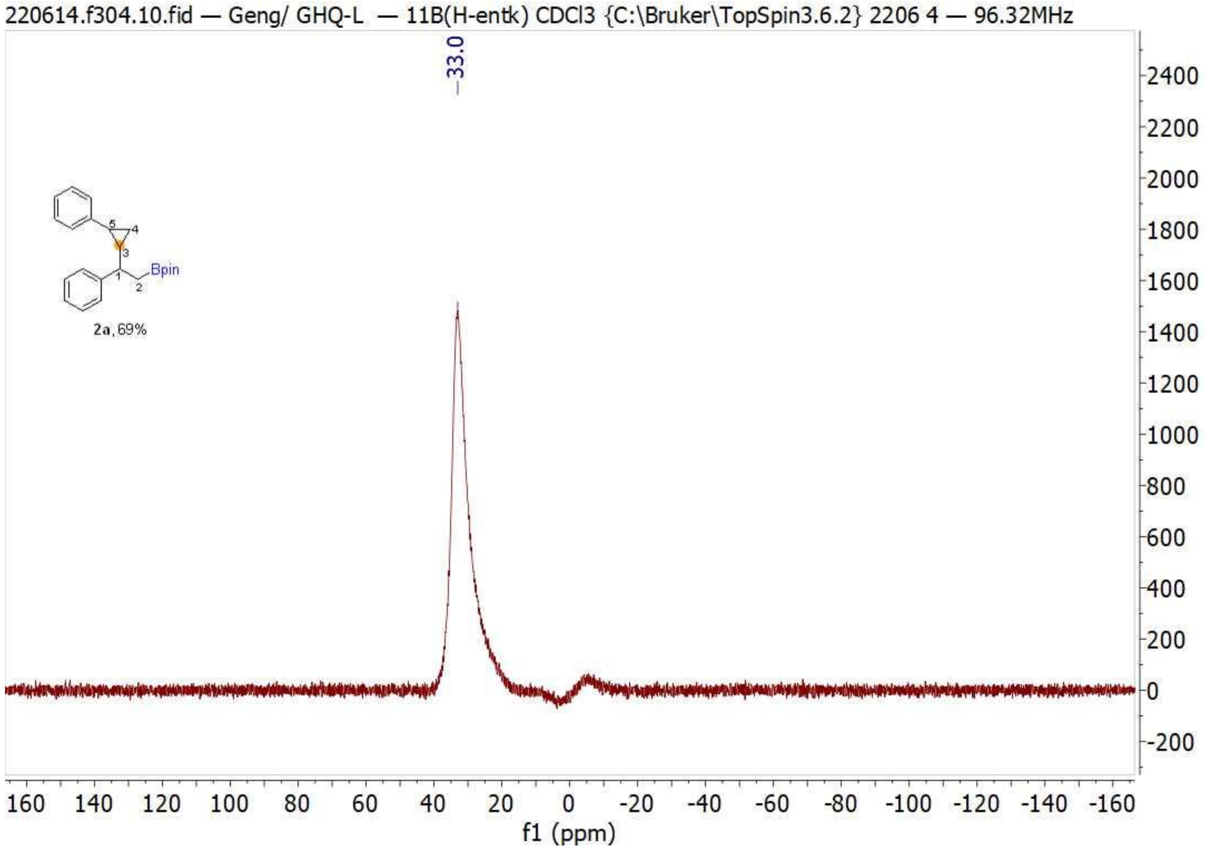
220614.f304.10.fid — Geng/ GHQ-L — 11B(H-entk) CDCl3 {C:\Bruker\TopSpin3.6.2} 2206 4 — 96.32MHz
33.0
Bpin
2a, 69%
f1 (ppm)

220617.f302.10.fid — Geng/ GHQ-L-140 — PROTON CDCl3 {C:\Bruker\TopSpin3.6.2} 2206 2 — 300.20MHz

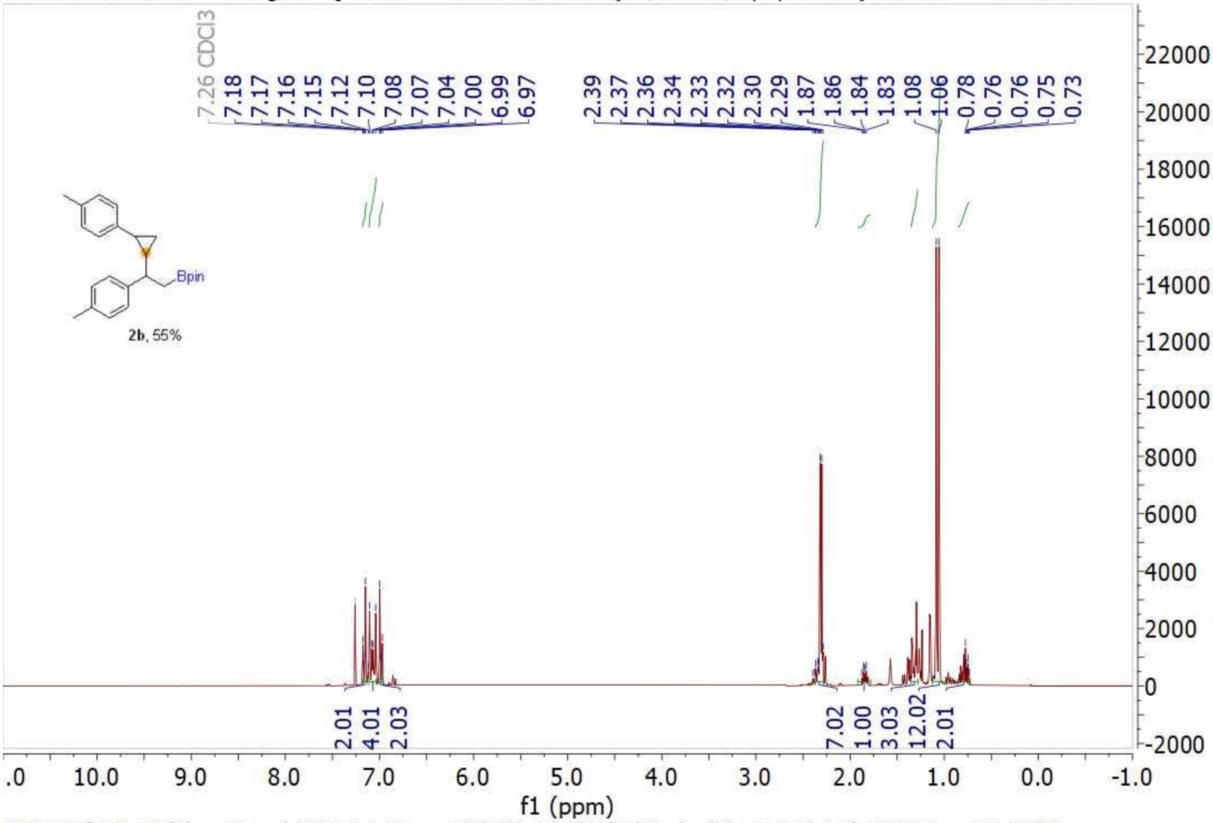

220617.f368.11.fid — Geng/ GHQ-L-140 — C13CPD CDCl3 {C:\Bruker\TopSpin3.6.2} 2206 8 — 75.49MHz

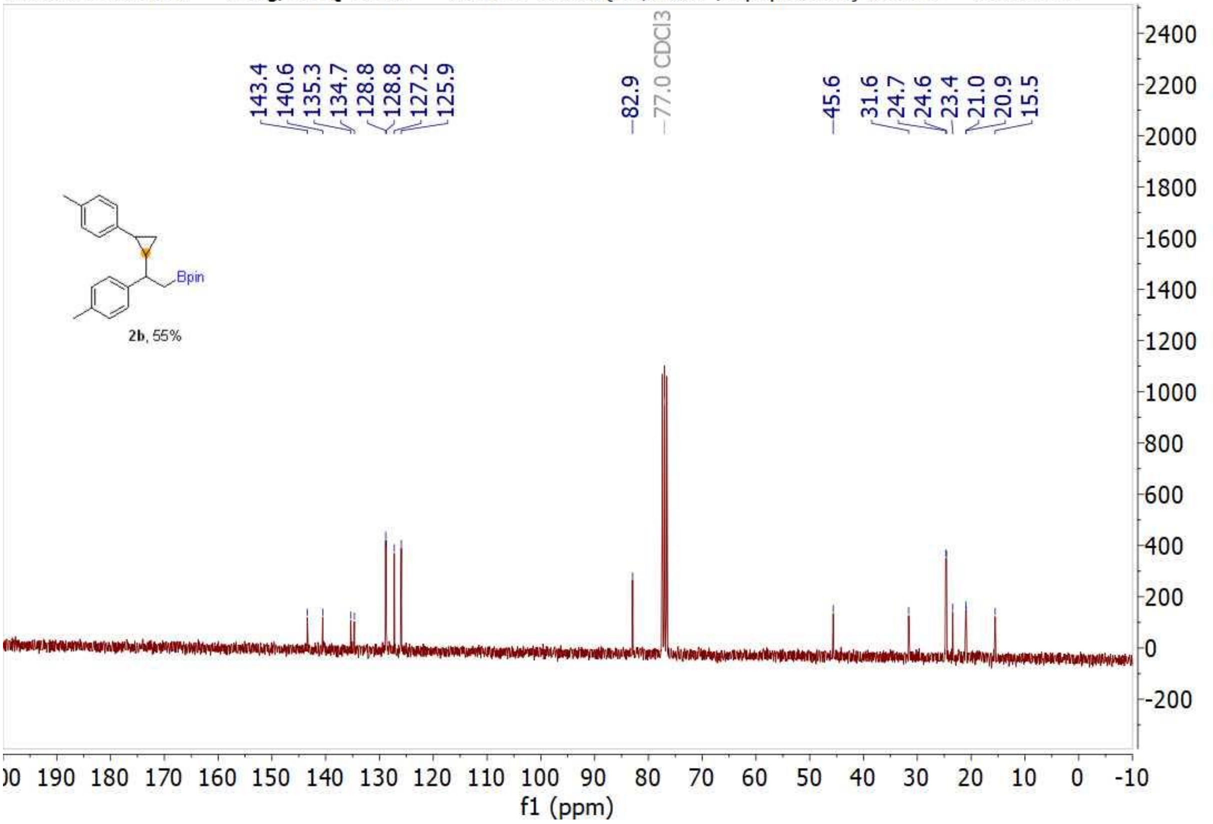

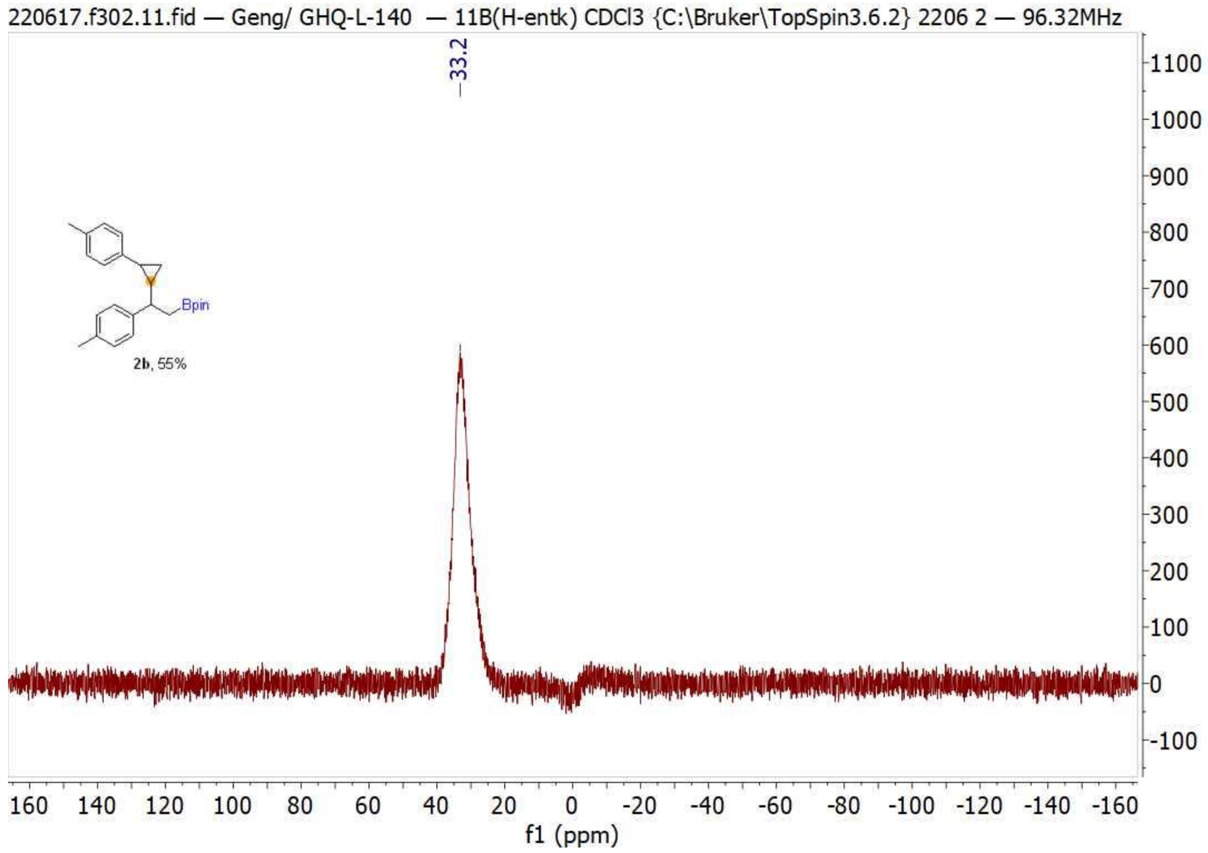

220617.f302.11.fid — Geng/ GHQ-L-140 — 11B(H-entk) CDCl3 {C:\Bruker\TopSpin3.6.2} 2206 2 — 96.32MHz
33.2
Bpin
2b, 55%
f1 (ppm)

220622.f318.10.fid — Huiqing Geng GHQ-L-142 — PROTON CDCl3 {C:\Bruker\TopSpin3.6.2} 2206 18 — 300.20MHz

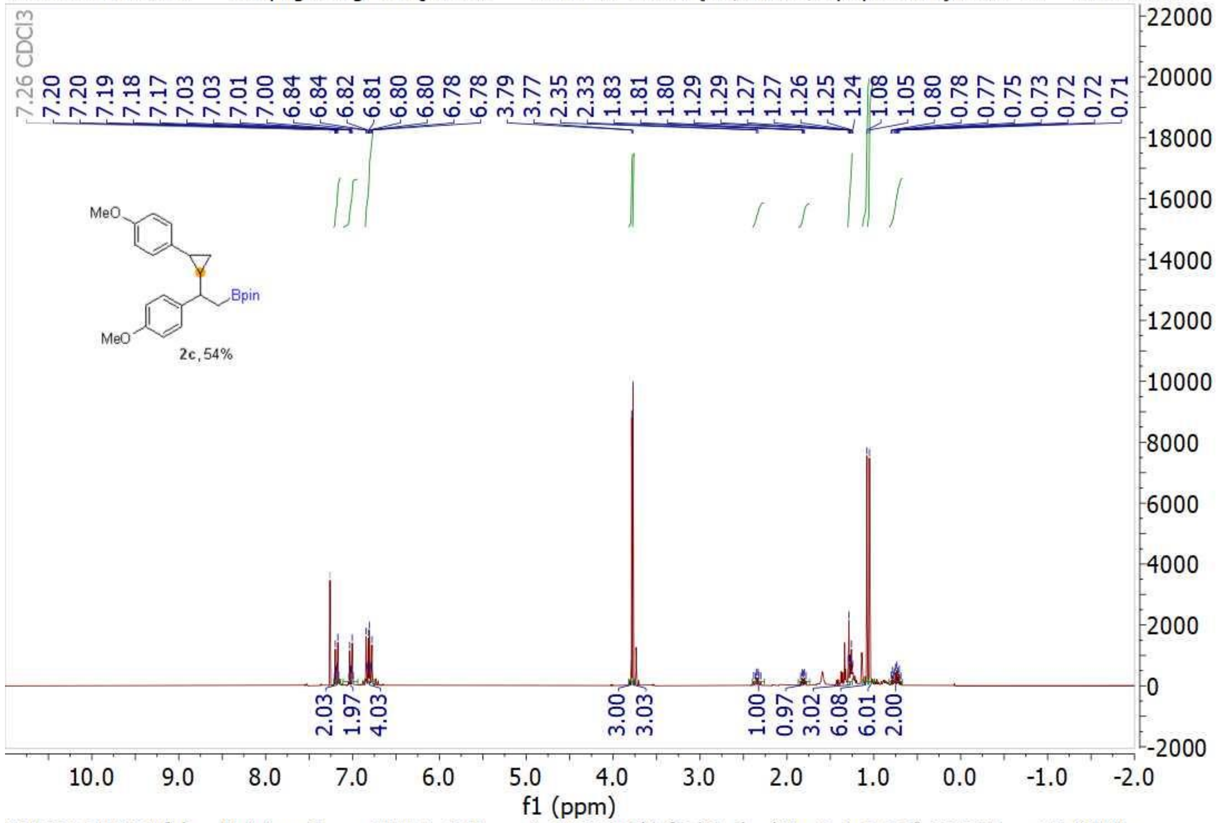

220623.305.11.fid — Huiqing Geng GHQ-L-142 — Au13C CDCl3 {C:\Bruker\TopSpin3.6.2} 2206 5 — 75.48MHz

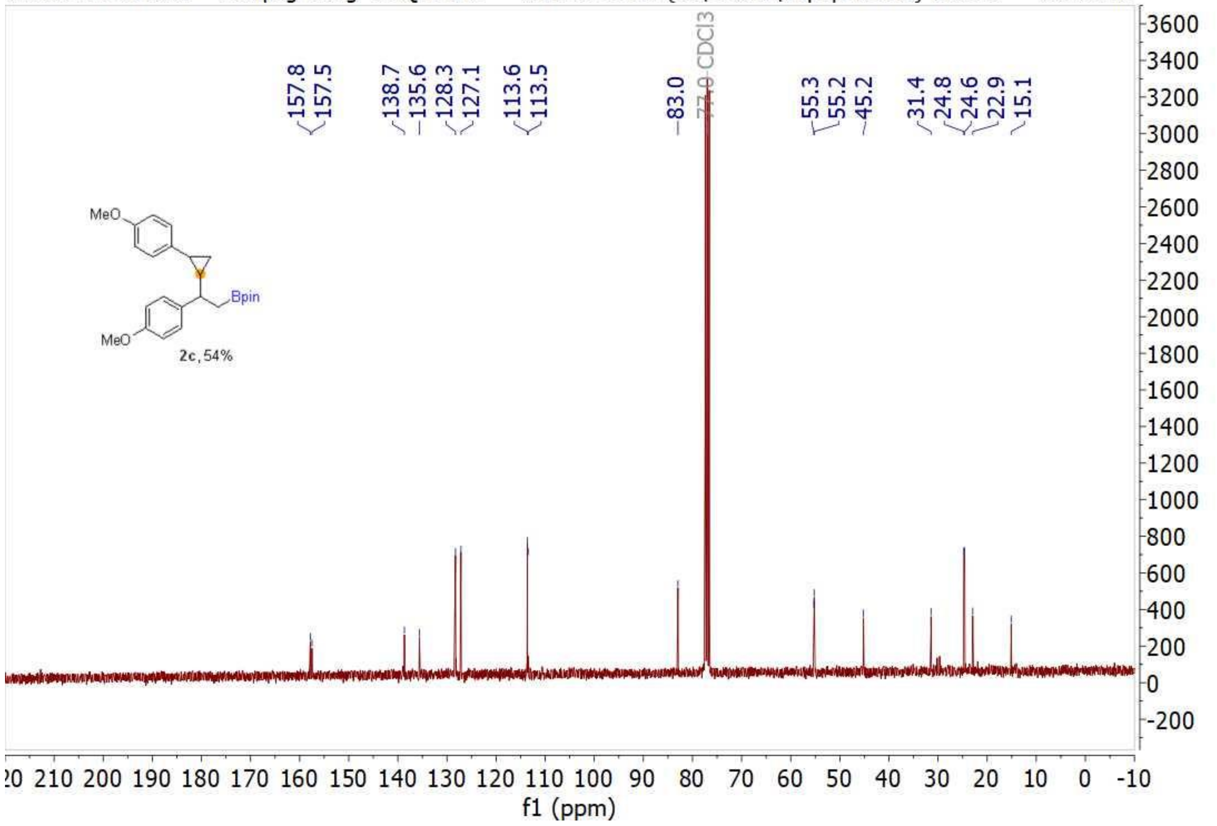

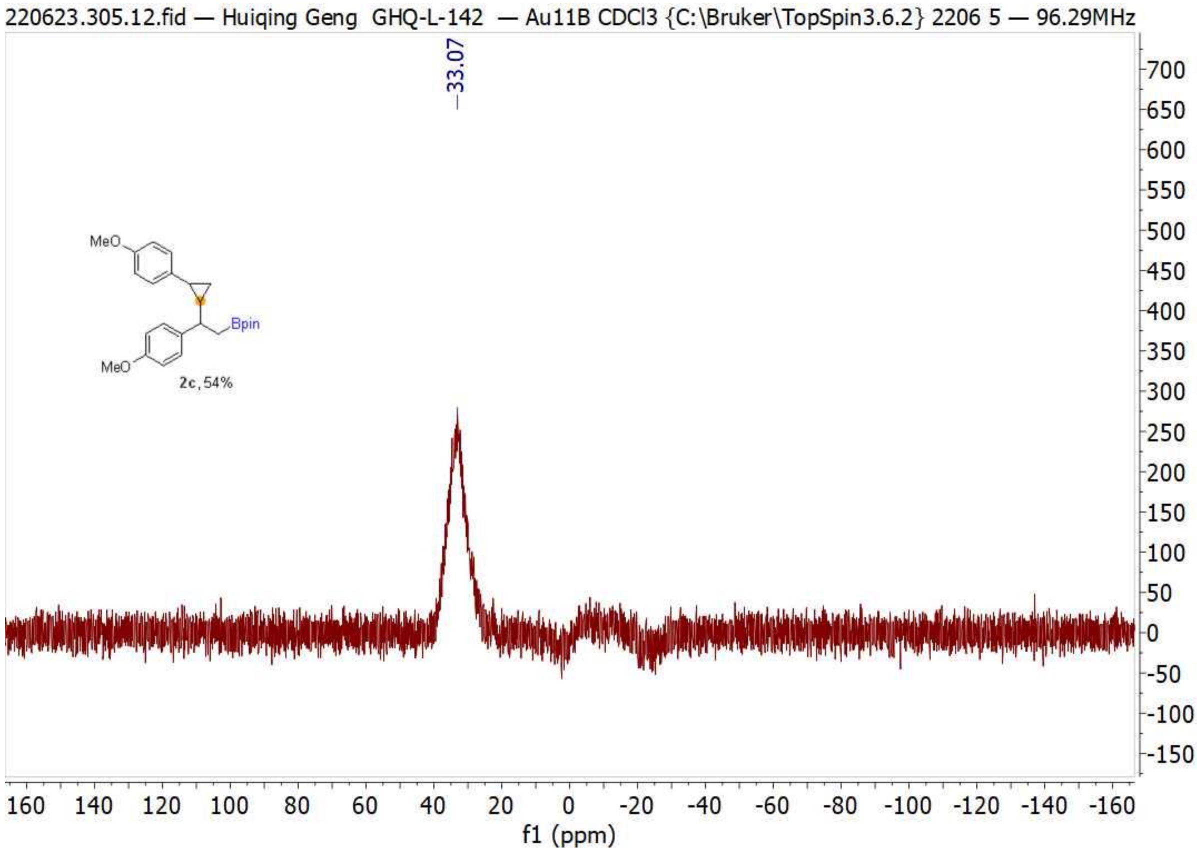

220623.305.12.fid — Huiqing Geng GHQ-L-142 — Au11B CDCl3 {C:\Bruker\TopSpin3.6.2} 2206 5 — 96.29MHz
33.07
MeO
Bpin
MeO
2c, 54%
160 140 120 100 80 60 40 20 0 -20 -40 -60 -80 -100 -120 -140 -160
f1 (ppm)
700 650 600 550 500 450 400 350 300 250 200 150 100 50 0 -50 -100 -150

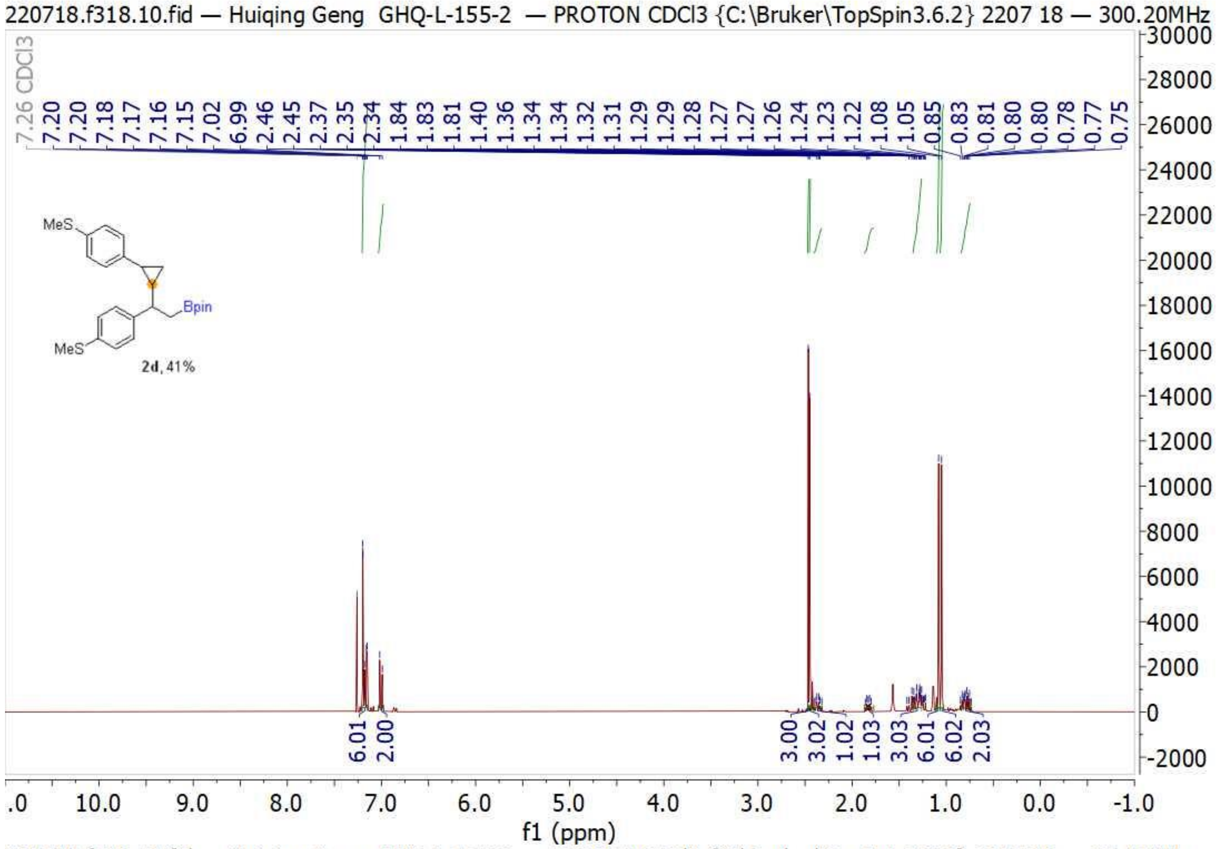
220718.f318.10.fid — Huiqing Geng GHQ-L-155-2 — PROTON CDCl3 {C:\Bruker\TopSpin3.6.2} 2207 18 — 300.20MHz
7.26 CDCl3
7.20 7.20 7.18 7.17 7.16 7.15 7.02 6.99 2.46 2.45 2.37 2.35 2.34 1.84 1.83 1.81 1.40 1.36 1.34 1.34 1.32 1.31 1.29 1.29 1.28 1.27 1.27 1.26 1.24 1.23 1.22 1.08 1.05 0.85 0.83 0.81 0.80 0.80 0.78 0.77 0.75
MeS
Bpin
MeS
2d, 41%
6.01 2.00 3.00 3.02 1.02 1.03 3.03 6.01 6.02 2.03
f1 (ppm)
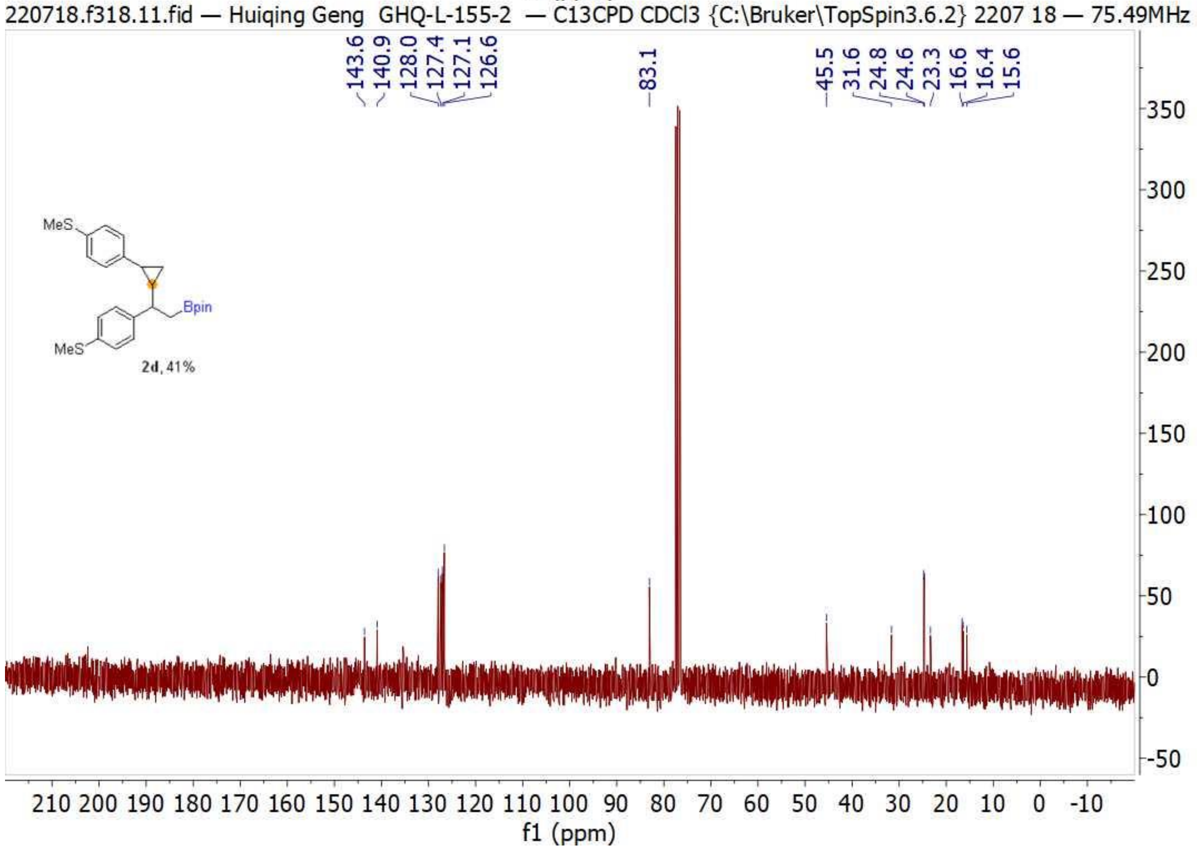
220718.f318.11.fid — Huiqing Geng GHQ-L-155-2 — C13CPD CDCl3 {C:\Bruker\TopSpin3.6.2} 2207 18 — 75.49MHz
143.6 140.9 128.0 127.4 127.1 126.6 83.1 45.5 31.6 24.8 24.6 23.3 16.6 16.4 15.6
MeS
Bpin
MeS
2d, 41%
f1 (ppm)

220718.f318.12.fid — Huiqing Geng GHQ-L-155-2 — 11B CDCl3 {C:\Bruker\TopSpin3.6.2} 2207 18 — 96.32MHz

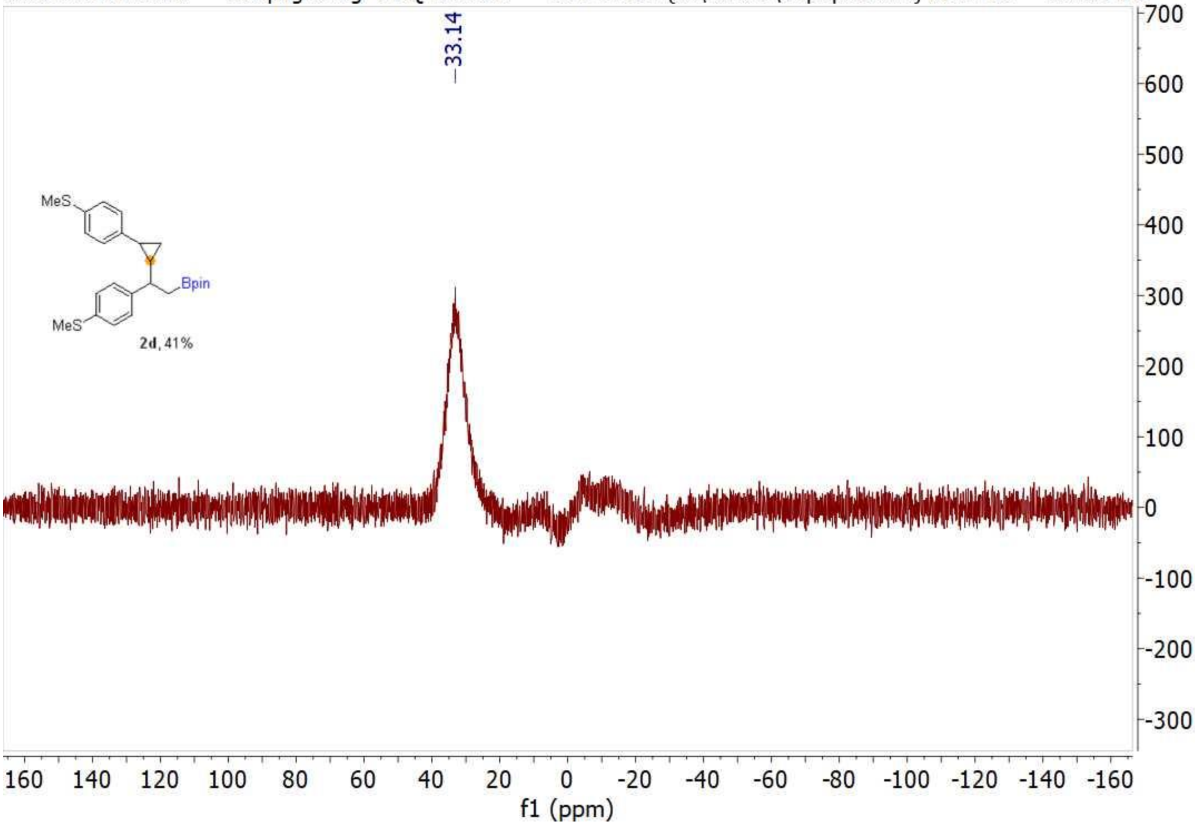

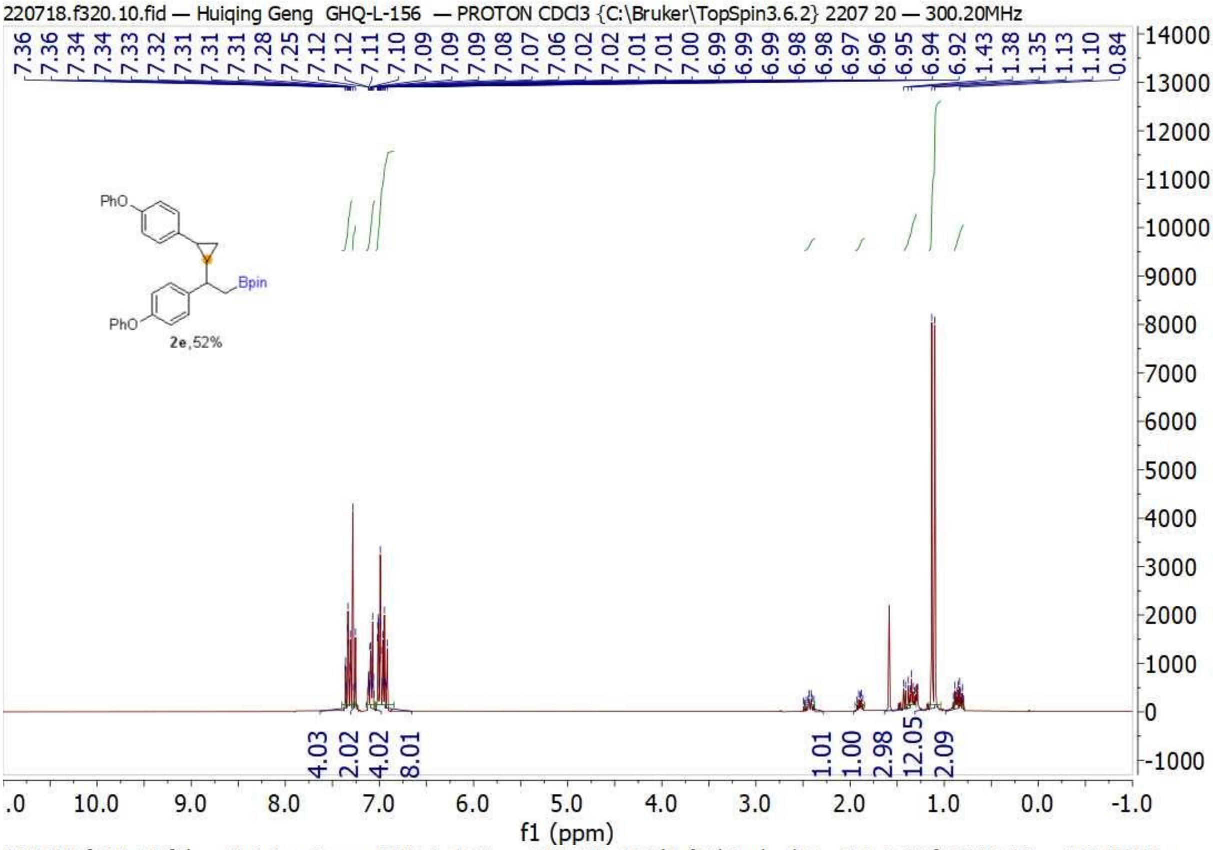
220718.f320.10.fid — Huiqing Geng GHQ-L-156 — PROTON CDCl3 {C:\Bruker\TopSpin3.6.2} 2207 20 — 300.20MHz
PhO
Bpin
PhO
2e, 52%
f1 (ppm)

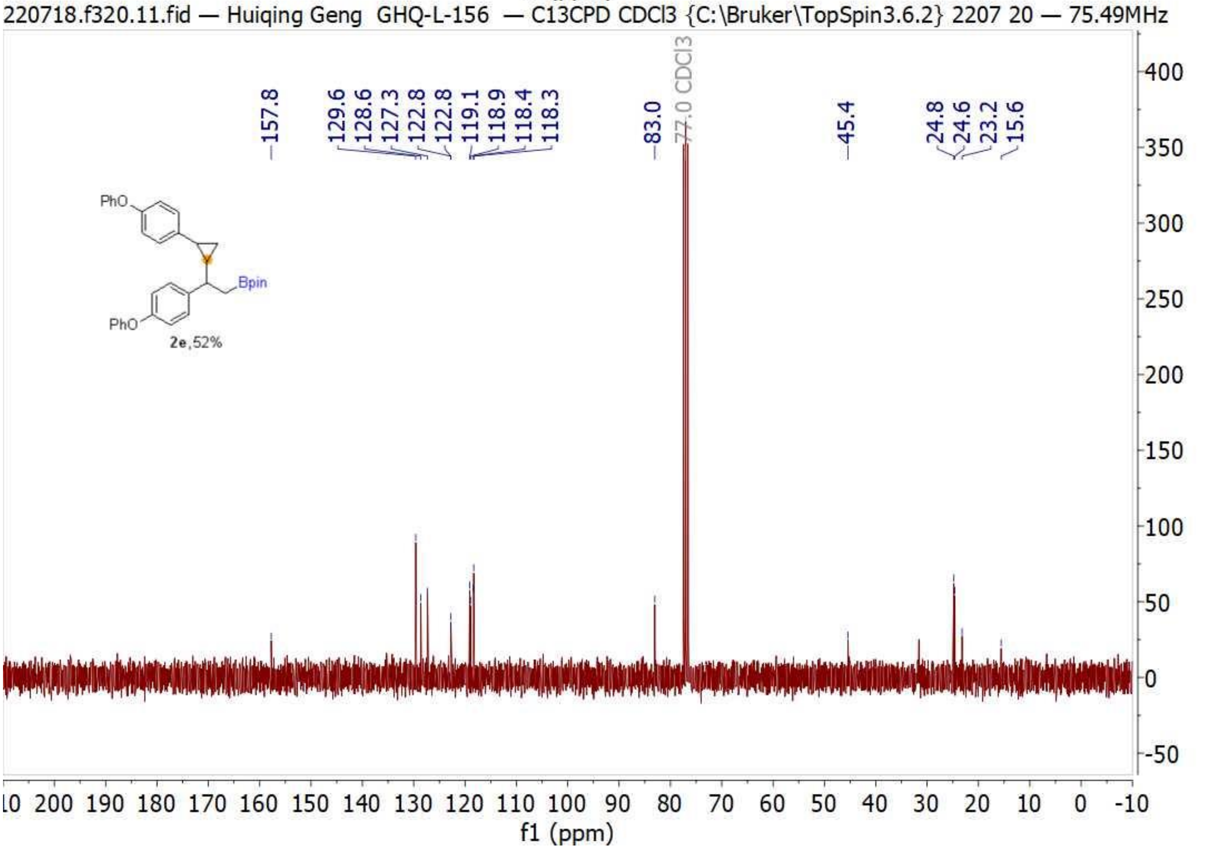
220718.f320.11.fid — Huiqing Geng GHQ-L-156 — C13CPD CDCl3 {C:\Bruker\TopSpin3.6.2} 2207 20 — 75.49MHz
157.8
129.6
128.6
127.3
122.8
122.8
119.1
118.9
118.4
118.3
83.0
77.0 CDCl3
45.4
24.8
24.6
23.2
15.6
PhO
Bpin
PhO
2e, 52%
f1 (ppm)

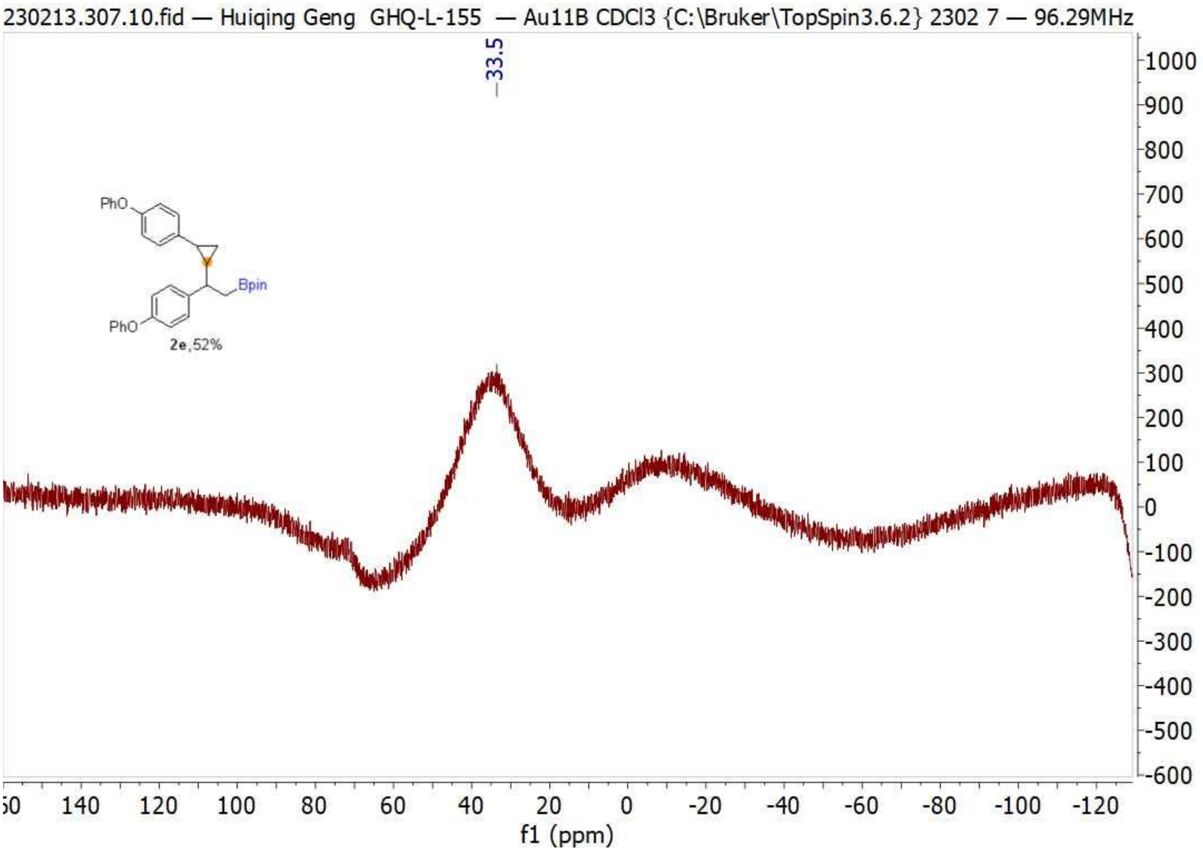
230213.307.10.fid — Huiqing Geng GHQ-L-155 — Au11B CDCl3 {C:\Bruker\TopSpin3.6.2} 2302 7 — 96.29MHz
33.5
PhO
Bpin
PhO
2e, 52%
f1 (ppm)

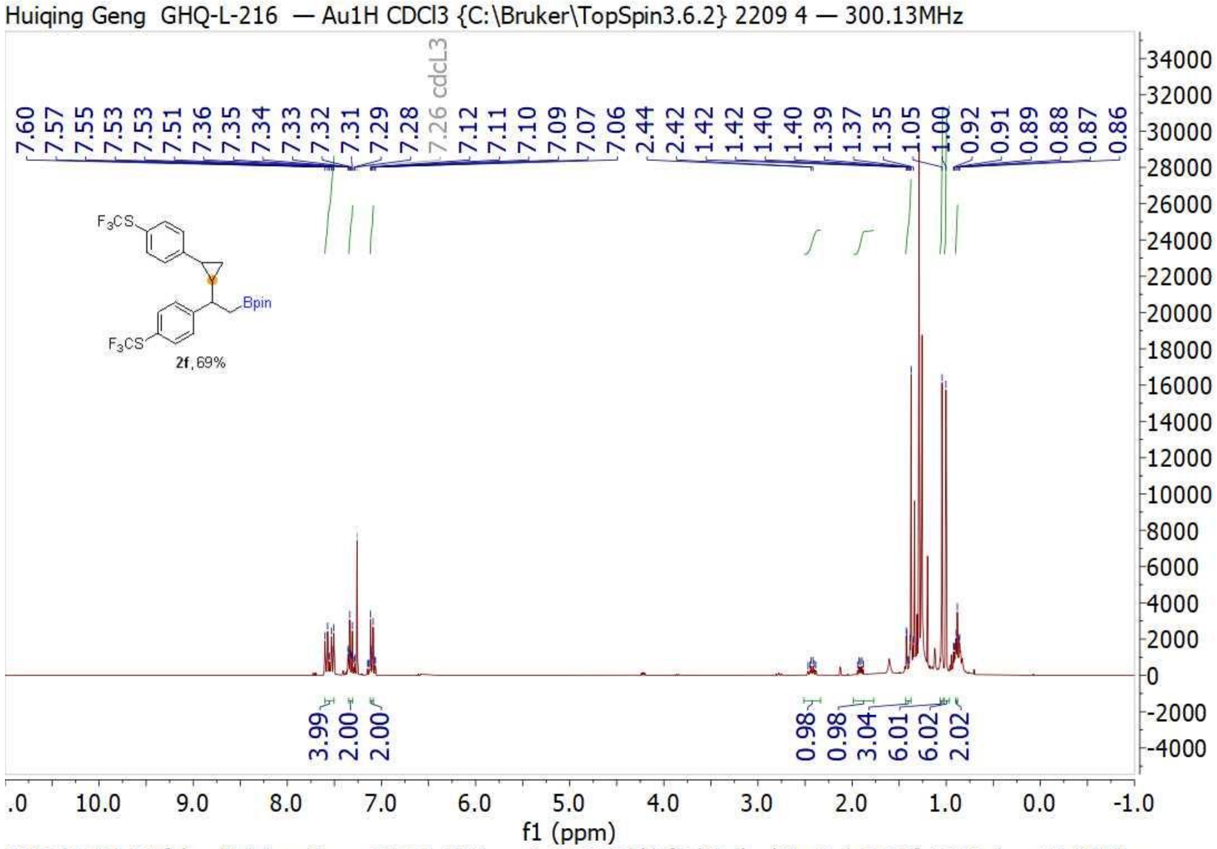
Huiqing Geng GHQ-L-216 — Au1H CDCl3 {C:\Bruker\TopSpin3.6.2} 2209 4 — 300.13MHz
7.60 7.57 7.55 7.53 7.53 7.51 7.36 7.35 7.34 7.33 7.32 7.31 7.29 7.28 7.26 cdcL3 7.12 7.11 7.10 7.09 7.07 7.06 2.44 2.42 1.42 1.42 1.40 1.40 1.39 1.37 1.35 1.05 1.00 0.92 0.91 0.89 0.88 0.87 0.86
F3CS
Bpin
2f, 69%
3.99 2.00 2.00 0.98 0.98 3.04 6.01 6.02 2.02
f1 (ppm)

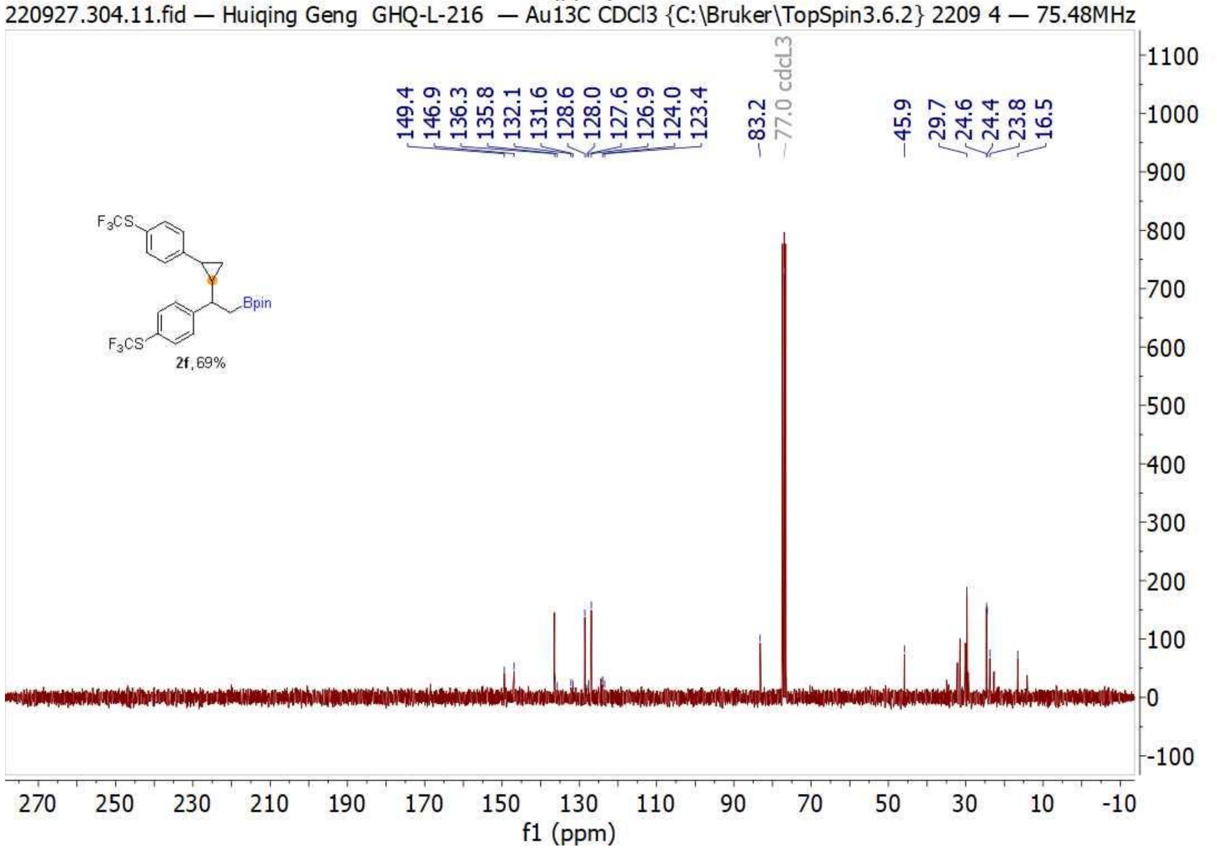
220927.304.11.fid — Huiqing Geng GHQ-L-216 — Au13C CDCl3 {C:\Bruker\TopSpin3.6.2} 2209 4 — 75.48MHz
149.4 146.9 136.3 135.8 132.1 131.6 128.6 128.0 127.6 126.9 124.0 123.4 83.2 77.0 cdcL3 45.9 29.7 24.6 24.4 23.8 16.5
F3CS
Bpin
2f, 69%
f1 (ppm)

Huiqing Geng GHQ-L-216 — Au11B CDCl3 {C:\Bruker\TopSpin3.6.2} 2209 4 — 96.29MHz

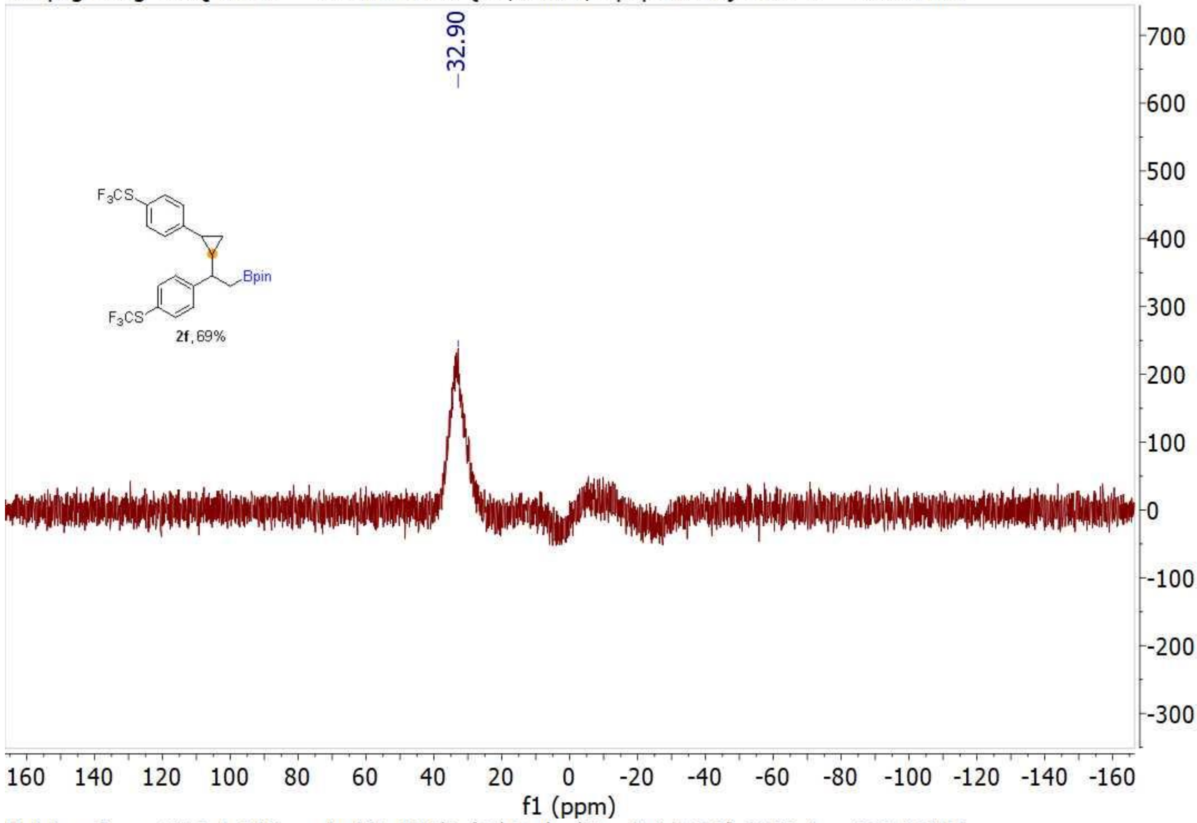

Huiqing Geng GHQ-L-216 — Au19F CDCl3 {C:\Bruker\TopSpin3.6.2} 2302 4 — 282.39MHz

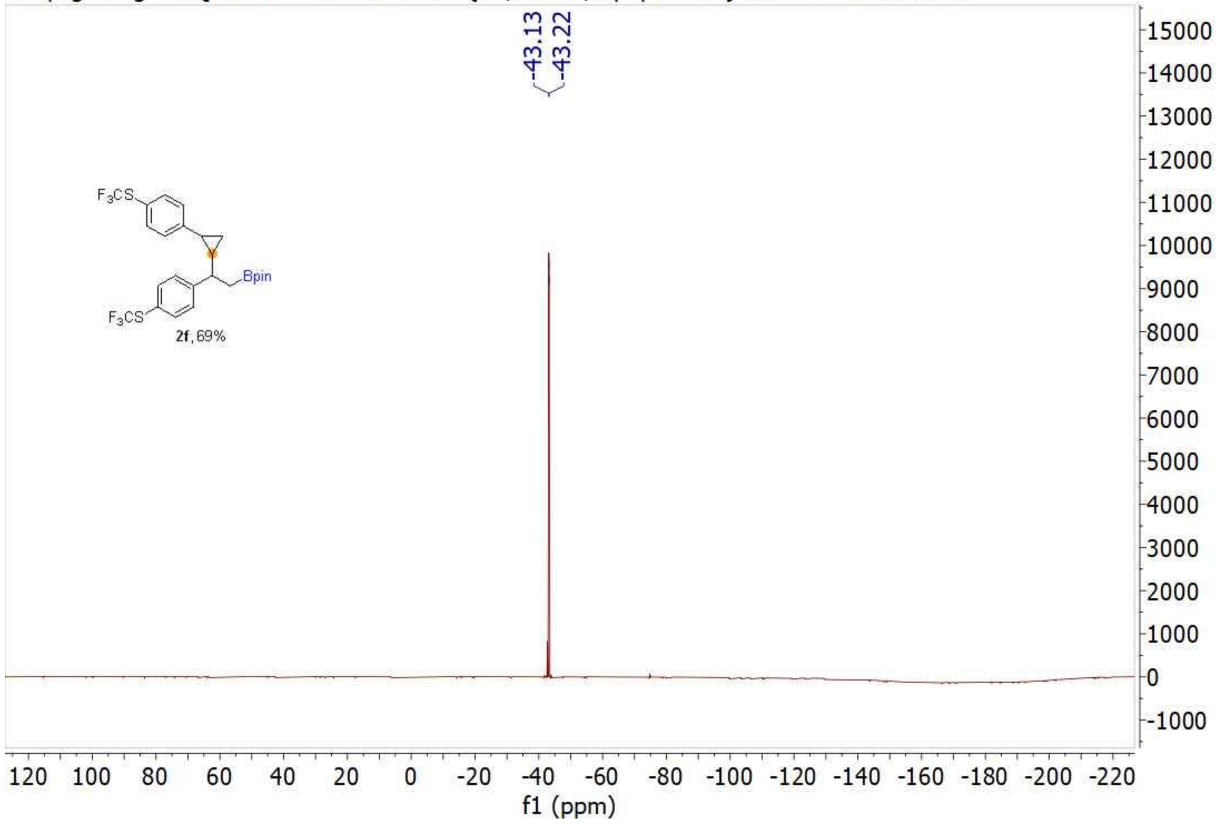

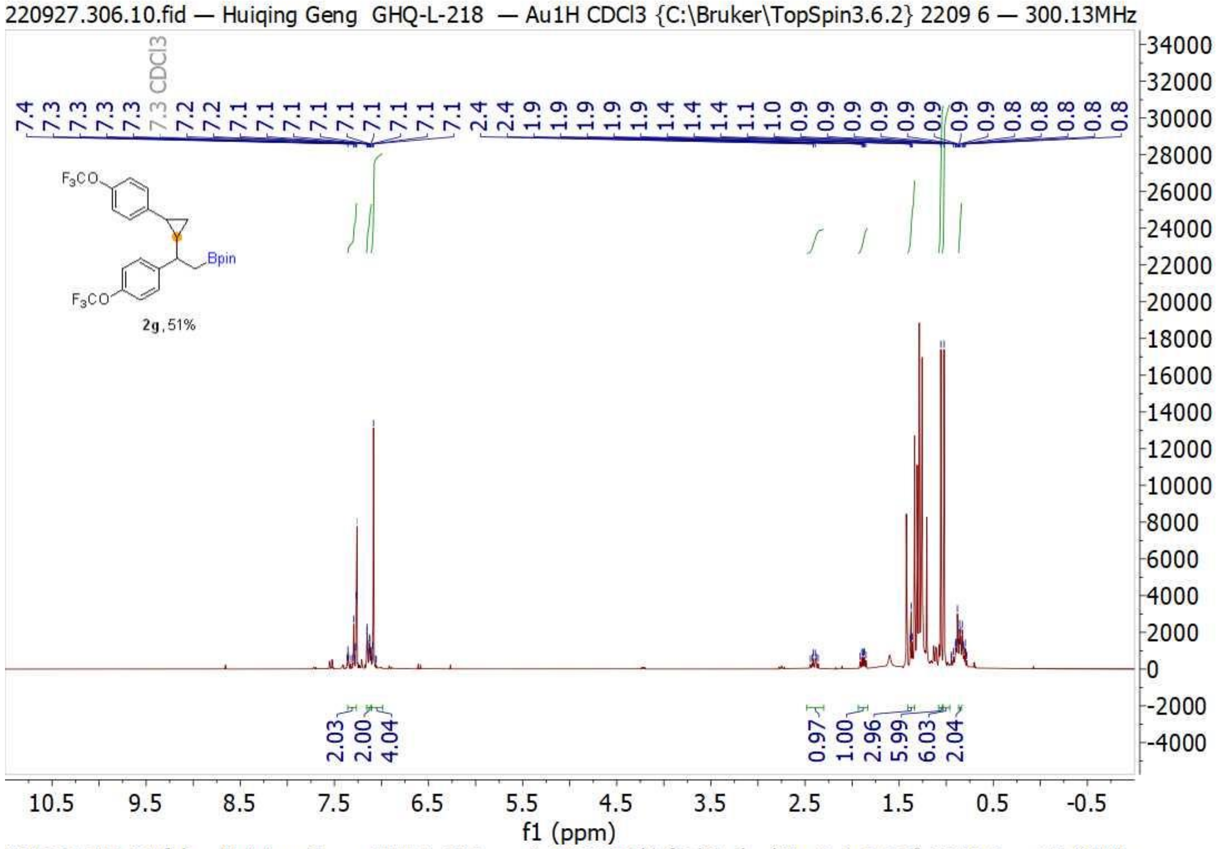
220927.306.10.fid — Huiqing Geng GHQ-L-218 — Au1H CDCl3 {C:\Bruker\TopSpin3.6.2} 2209 6 — 300.13MHz
7.3 CDCl3
F3CO
Bpin
2g, 51%
2.03 2.00 4.04
0.97 1.00 2.96 5.99 6.03 2.04
f1 (ppm)

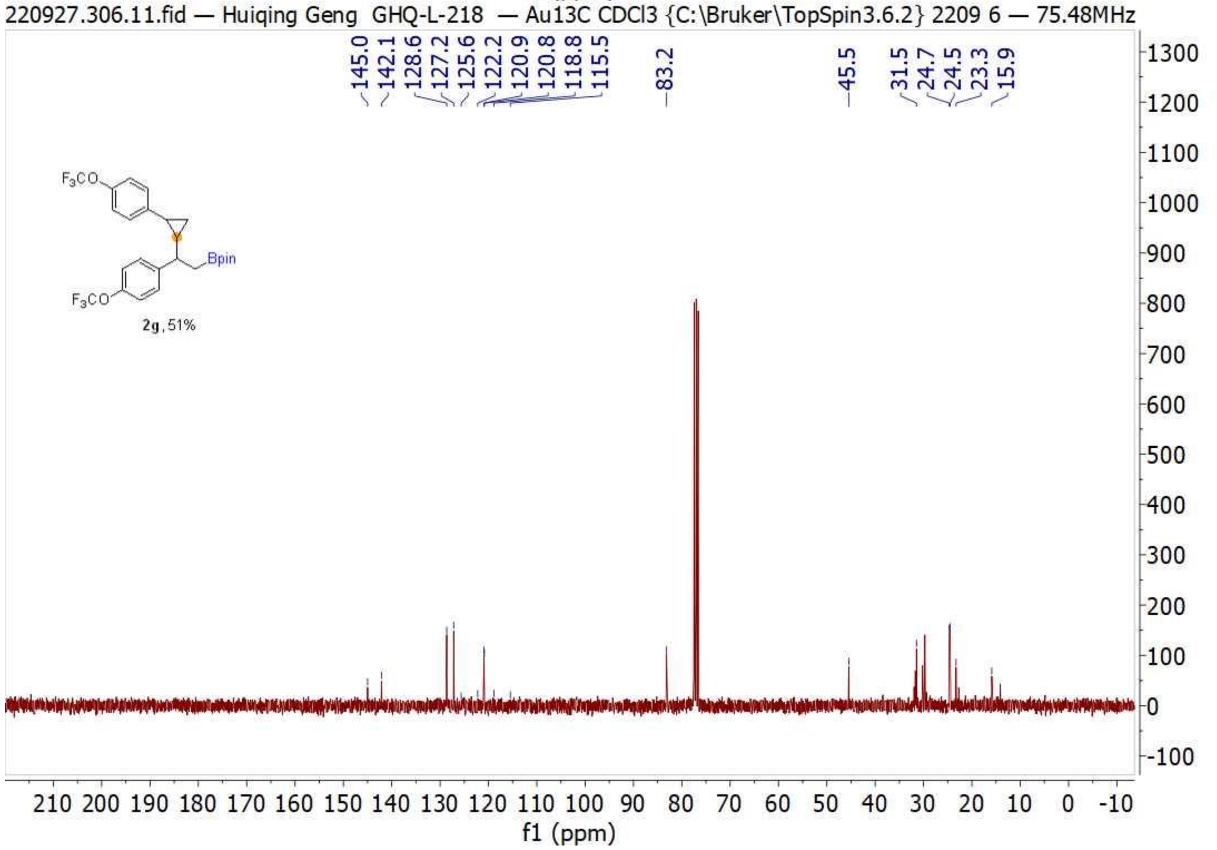
220927.306.11.fid — Huiqing Geng GHQ-L-218 — Au13C CDCl3 {C:\Bruker\TopSpin3.6.2} 2209 6 — 75.48MHz
145.0 142.1 128.6 127.2 125.6 122.2 120.9 120.8 118.8 115.5
83.2
45.5
31.5 24.7 24.5 23.3 15.9
F3CO
Bpin
2g, 51%
f1 (ppm)

Huiqing Geng GHQ-L-218 — Au11B CDCl3 {C:\Bruker\TopSpin3.6.2} 2209 6 — 96.29MHz

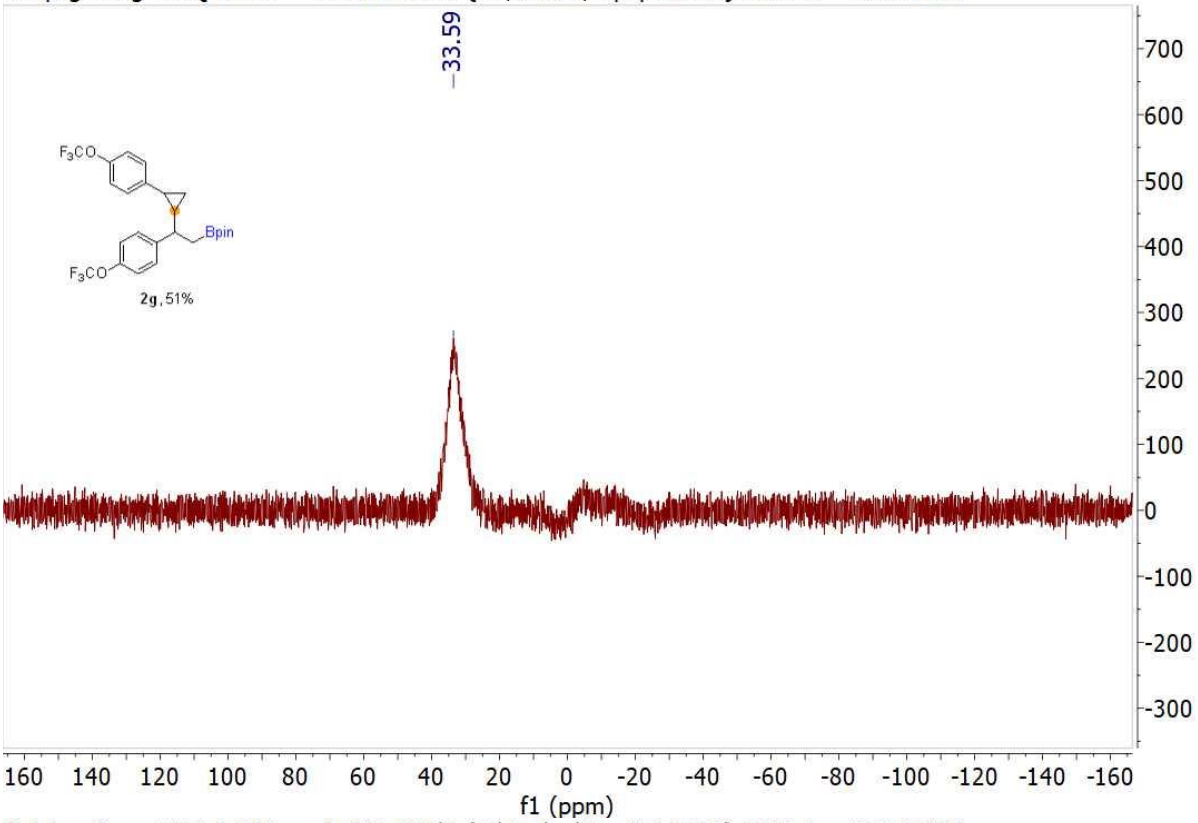

Huiqing Geng GHQ-L-218 — Au19F CDCl3 {C:\Bruker\TopSpin3.6.2} 2209 6 — 282.39MHz

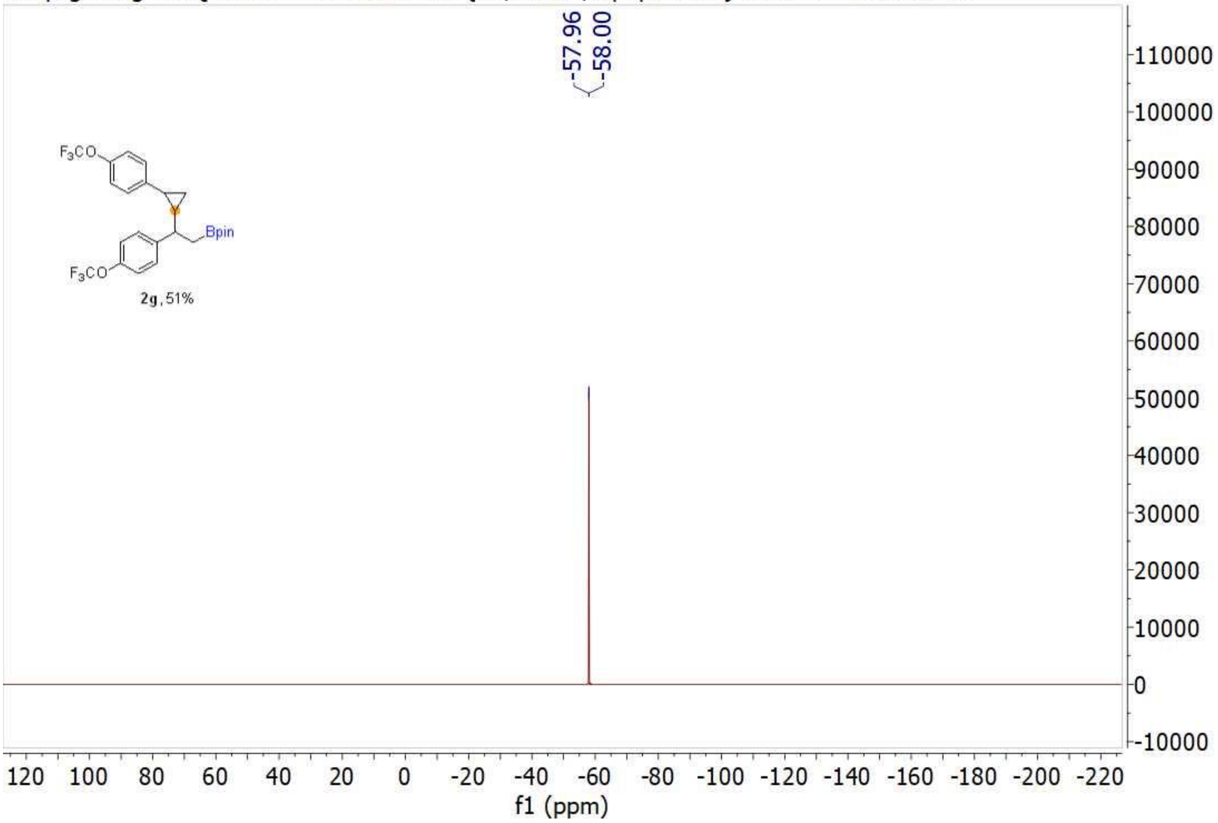

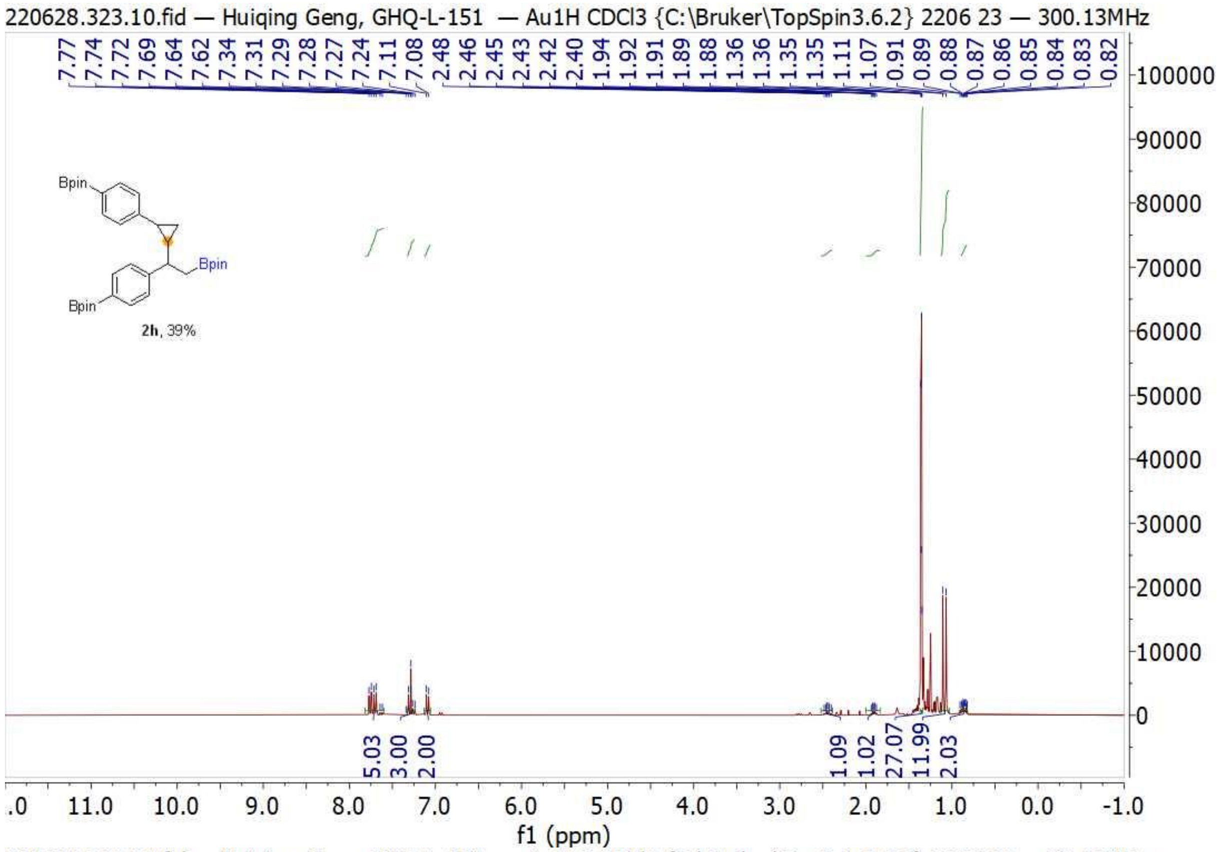
220628.323.10.fid — Huiqing Geng, GHQ-L-151 — Au1H CDCl3 {C:\Bruker\TopSpin3.6.2} 2206 23 — 300.13MHz
7.77 7.74 7.72 7.69 7.64 7.62 7.34 7.31 7.29 7.28 7.27 7.24 7.11 7.08 2.48 2.46 2.45 2.43 2.42 2.40 1.94 1.92 1.91 1.89 1.88 1.36 1.36 1.35 1.35 1.11 1.07 0.91 0.89 0.88 0.87 0.86 0.85 0.84 0.83 0.82
Bpin
Bpin
Bpin
2h, 39%
5.03 3.00 2.00 1.09 1.02 27.07 11.99 2.03
f1 (ppm)

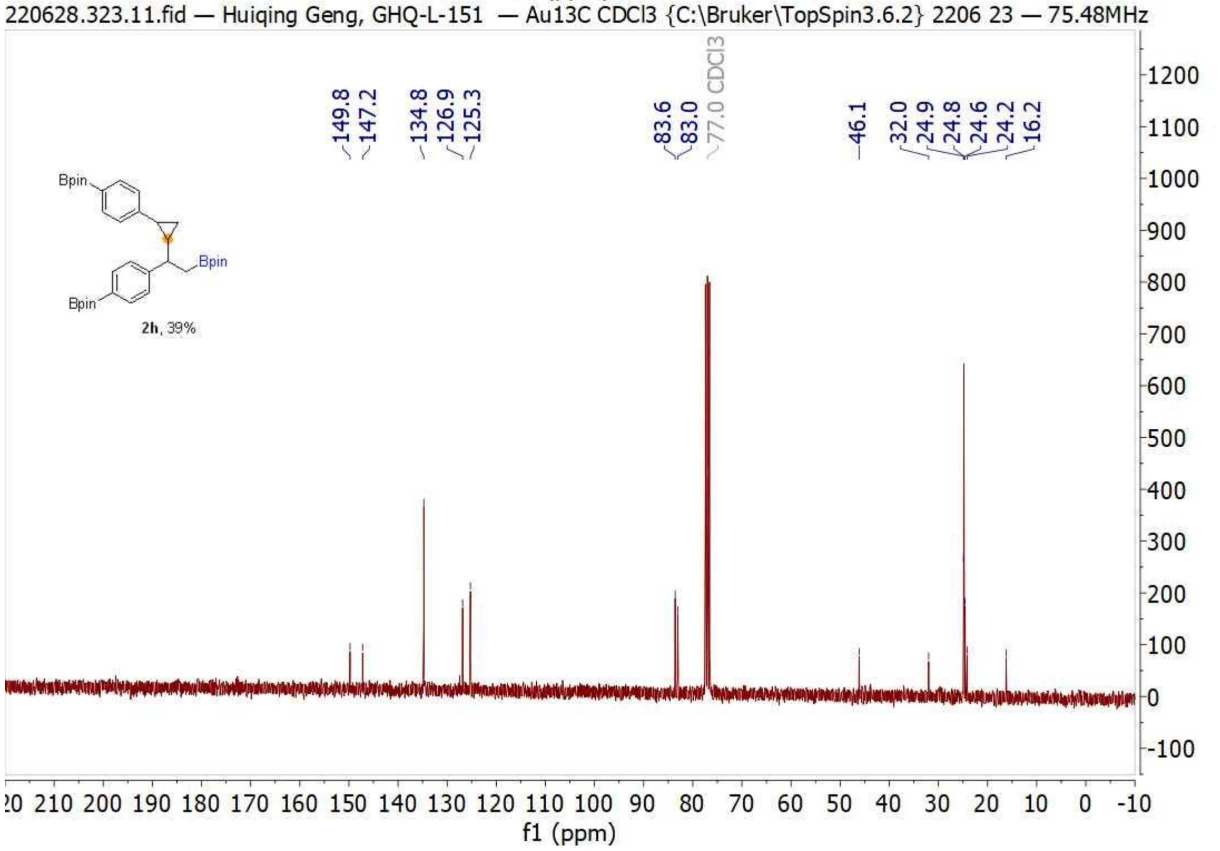
220628.323.11.fid — Huiqing Geng, GHQ-L-151 — Au13C CDCl3 {C:\Bruker\TopSpin3.6.2} 2206 23 — 75.48MHz
149.8 147.2 134.8 126.9 125.3 83.6 83.0 77.0 CDCl3 46.1 32.0 24.9 24.8 24.6 24.2 16.2
Bpin
Bpin
Bpin
2h, 39%
f1 (ppm)

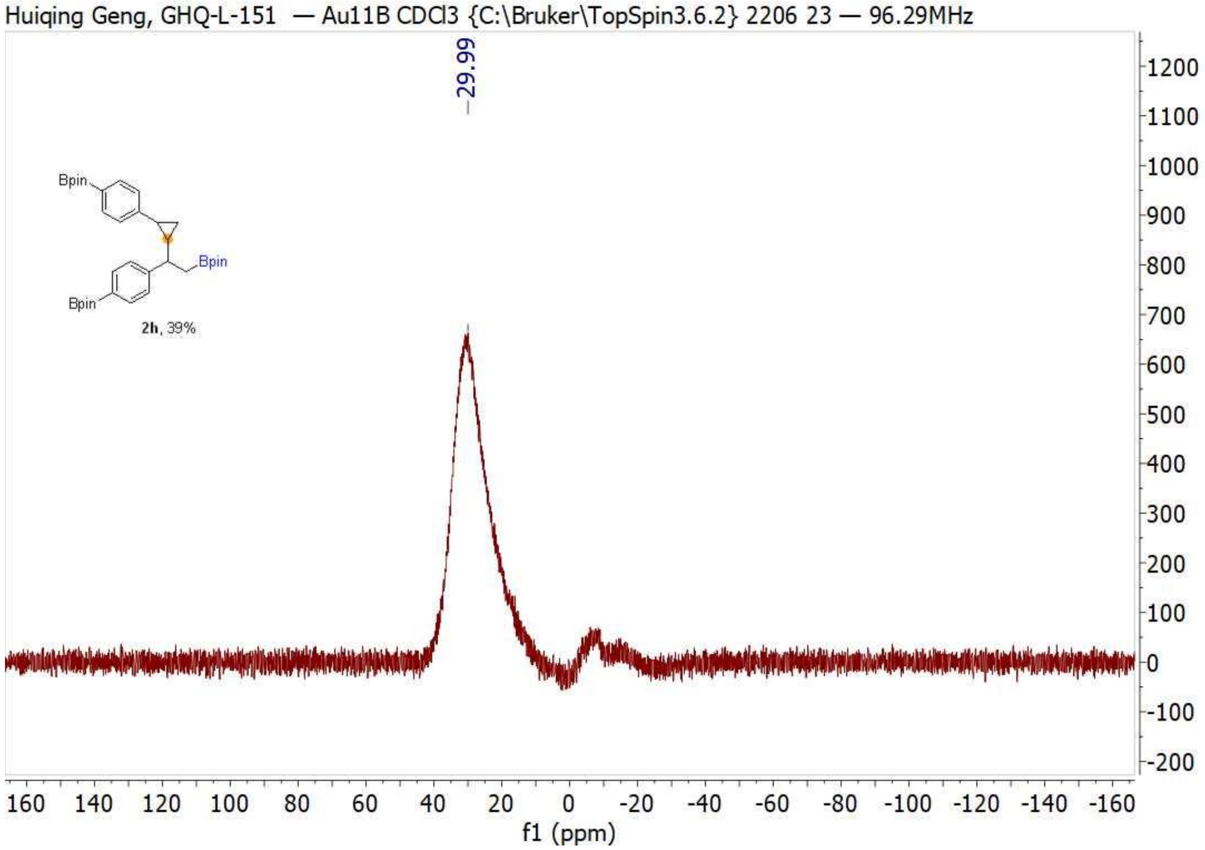
Huiqing Geng, GHQ-L-151 — Au11B CDCl3 {C:\Bruker\TopSpin3.6.2} 2206 23 — 96.29MHz
29.99
Bpin
Bpin
Bpin
2h, 39%
f1 (ppm)

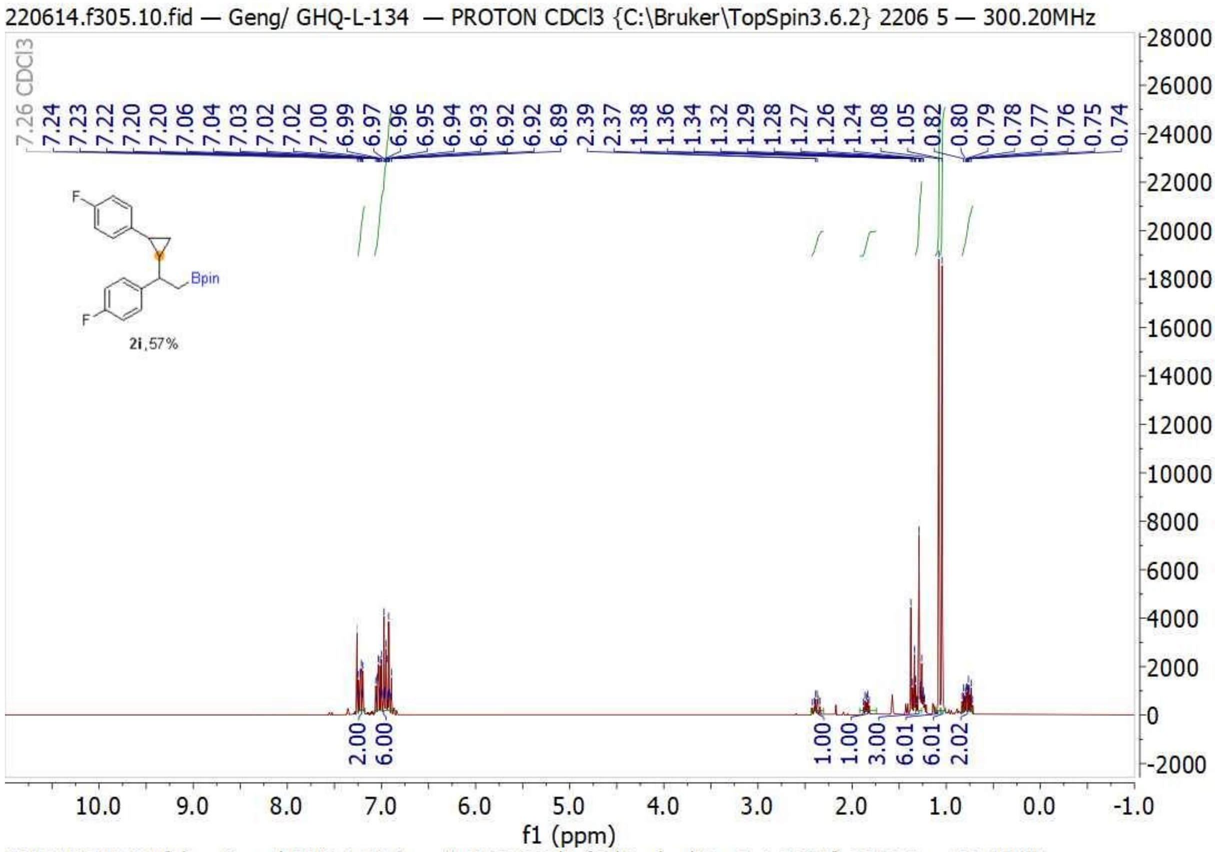
220614.f305.10.fid — Geng/ GHQ-L-134 — PROTON CDCl3 {C:\Bruker\TopSpin3.6.2} 2206 5 — 300.20MHz
7.26 CDCl3
7.24 7.23 7.22 7.20 7.20 7.06 7.04 7.03 7.02 7.02 7.00 6.99 6.97 6.96 6.95 6.94 6.93 6.92 6.92 6.89 2.39 2.37 1.38 1.36 1.34 1.32 1.29 1.28 1.27 1.26 1.24 1.08 1.05 0.82 0.80 0.79 0.78 0.77 0.76 0.75 0.74
Bpin
F
F
2i, 57%
2.00 6.00 1.00 1.00 3.00 6.01 6.01 2.02
f1 (ppm)

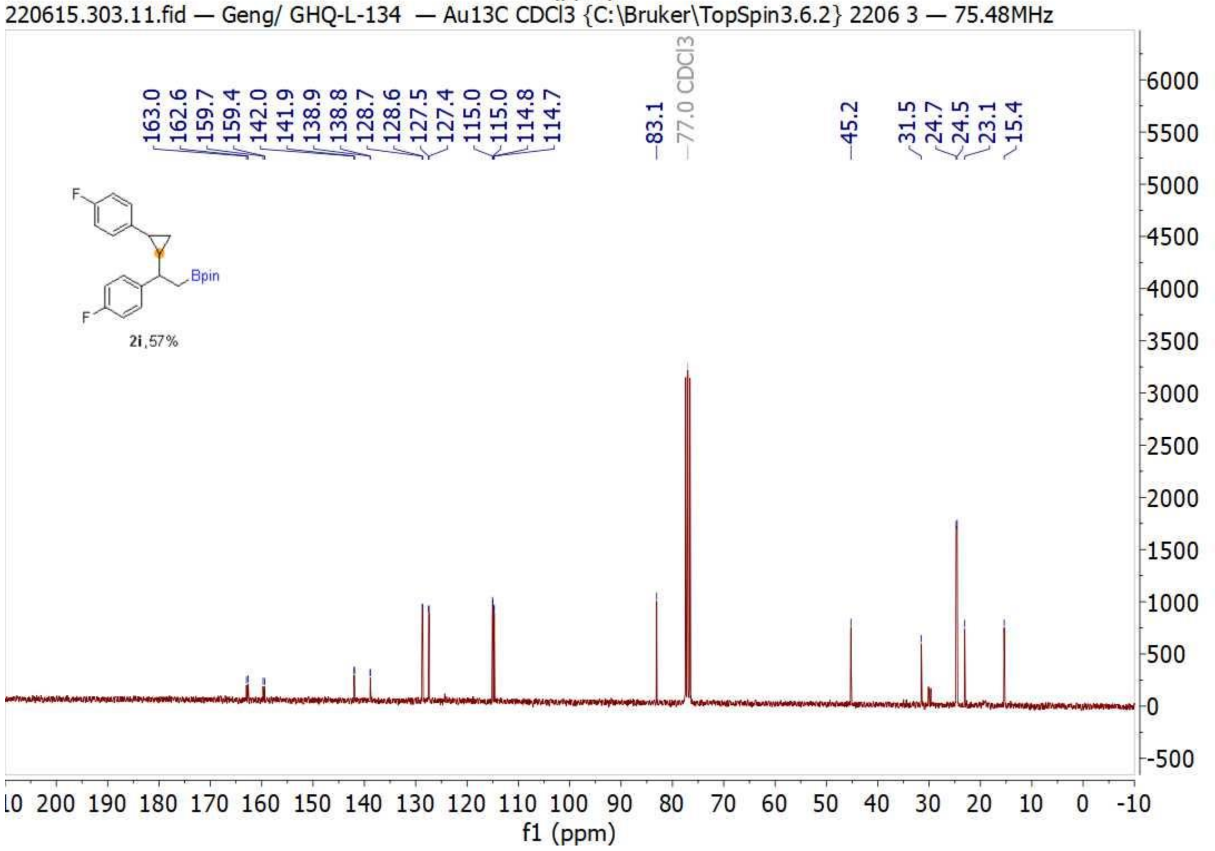
220615.303.11.fid — Geng/ GHQ-L-134 — Au13C CDCl3 {C:\Bruker\TopSpin3.6.2} 2206 3 — 75.48MHz
163.0 162.6 159.7 159.4 142.0 141.9 138.9 138.8 128.7 128.6 127.5 127.4 115.0 115.0 114.8 114.7
83.1
77.0 CDCl3
45.2
31.5 24.7 24.5 23.1 15.4
Bpin
F
F
2i, 57%
f1 (ppm)

Geng/ GHQ-L-134 — 11B(H-entk) CDCl3 {C:\Bruker\TopSpin3.6.2} 2206 5 — 96.32MHz

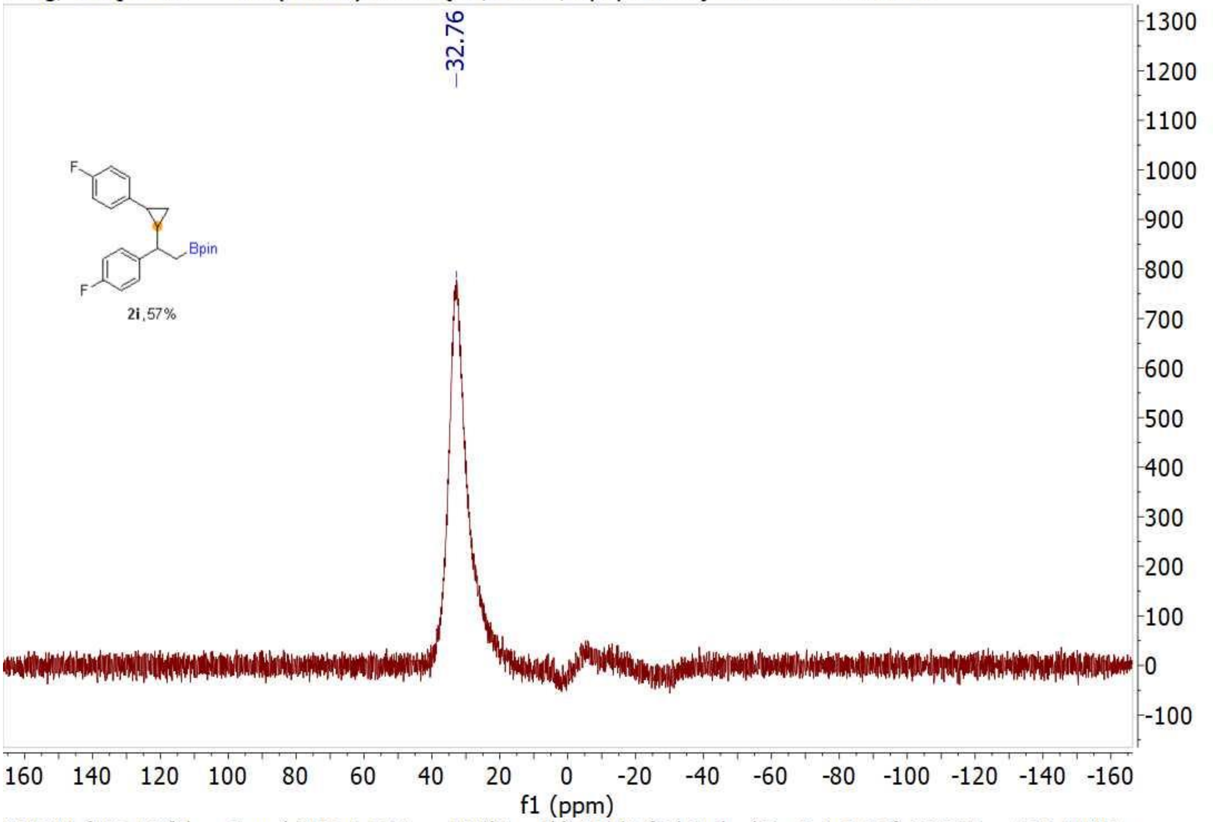

220614.f305.12.fid — Geng/ GHQ-L-134 — 19F(H-entk) CDCl3 {C:\Bruker\TopSpin3.6.2} 2206 5 — 282.44MHz

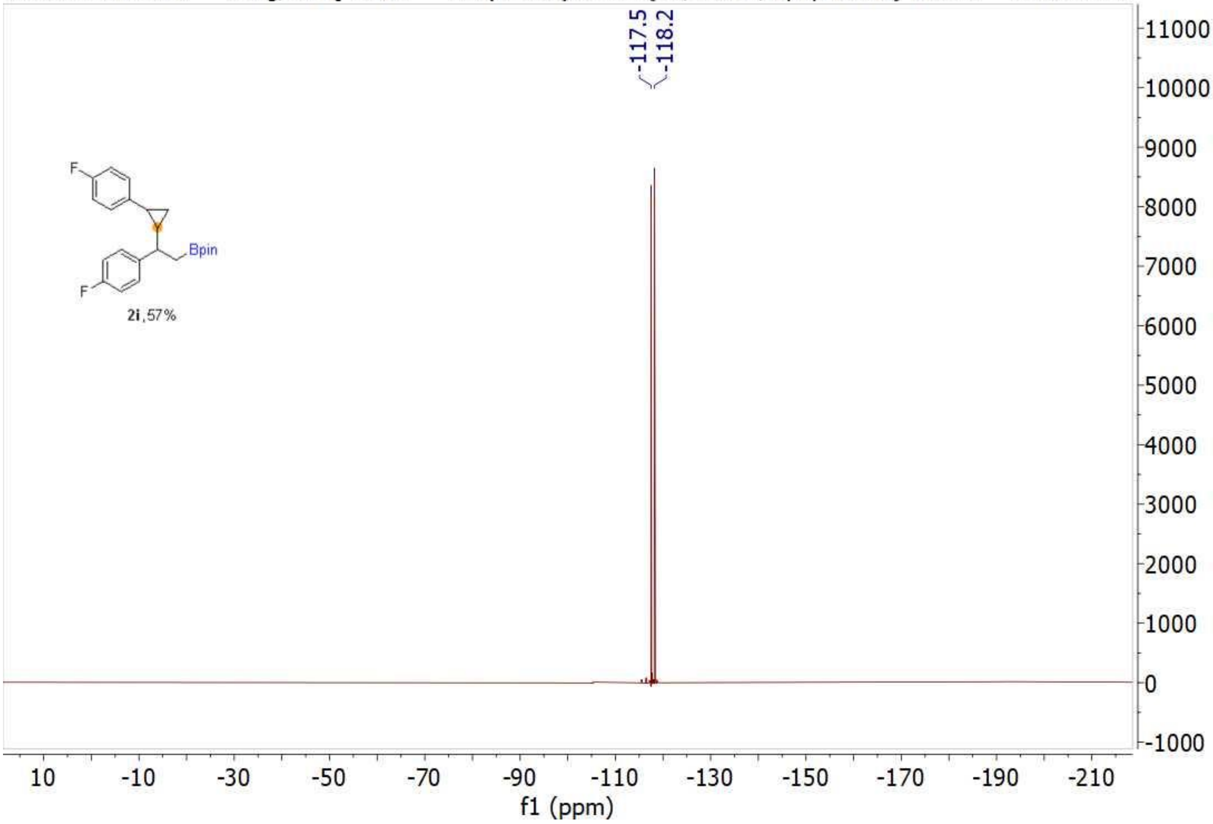

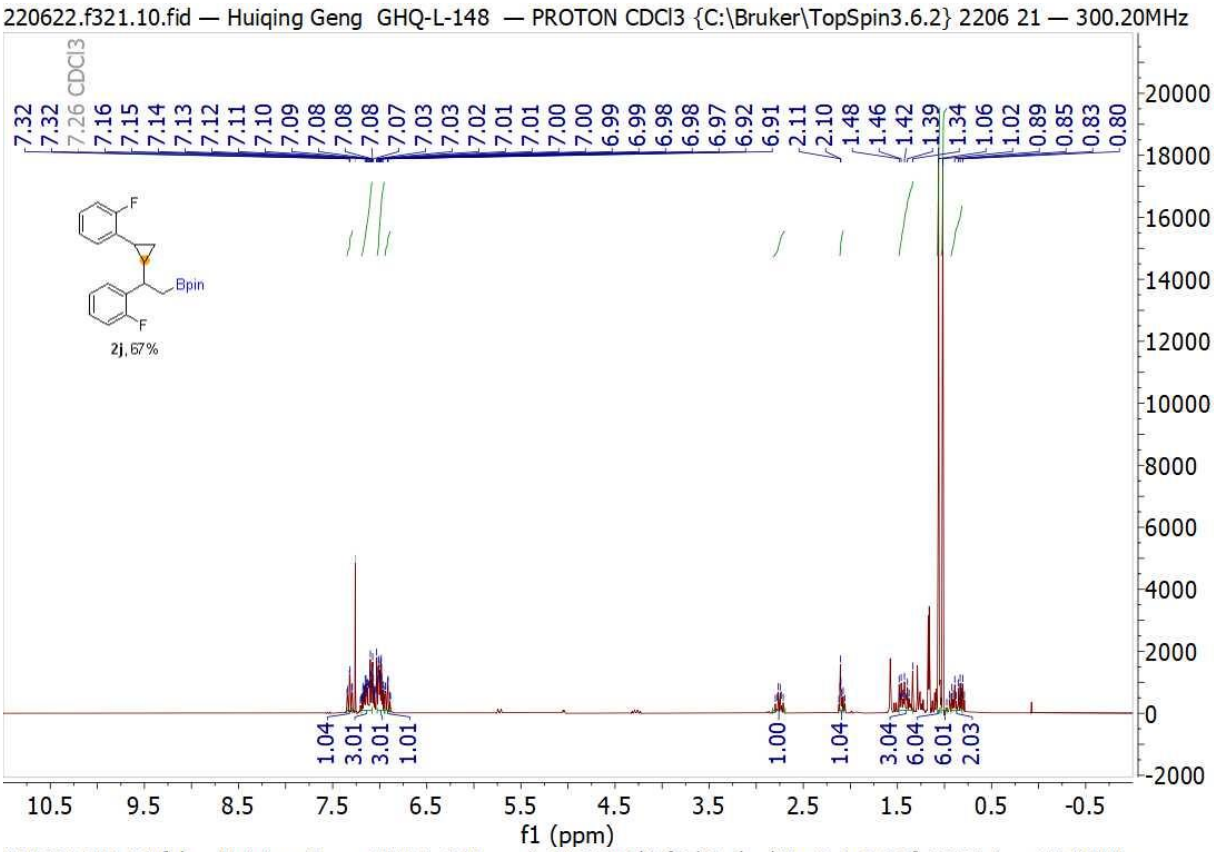
220622.f321.10.fid — Huiqing Geng GHQ-L-148 — PROTON CDCl3 {C:\Bruker\TopSpin3.6.2} 2206 21 — 300.20MHz
7.32 7.32 7.26 CDCl3 7.16 7.15 7.14 7.13 7.12 7.11 7.10 7.09 7.08 7.08 7.08 7.07 7.03 7.03 7.02 7.01 7.01 7.00 7.00 6.99 6.99 6.98 6.98 6.97 6.92 6.91 2.11 2.10 1.48 1.46 1.42 1.39 1.34 1.06 1.02 0.89 0.85 0.83 0.80
F
Bpin
F
2j, 67%
1.04 3.01 3.01 1.01 1.00 1.04 3.04 6.04 6.01 2.03
f1 (ppm)

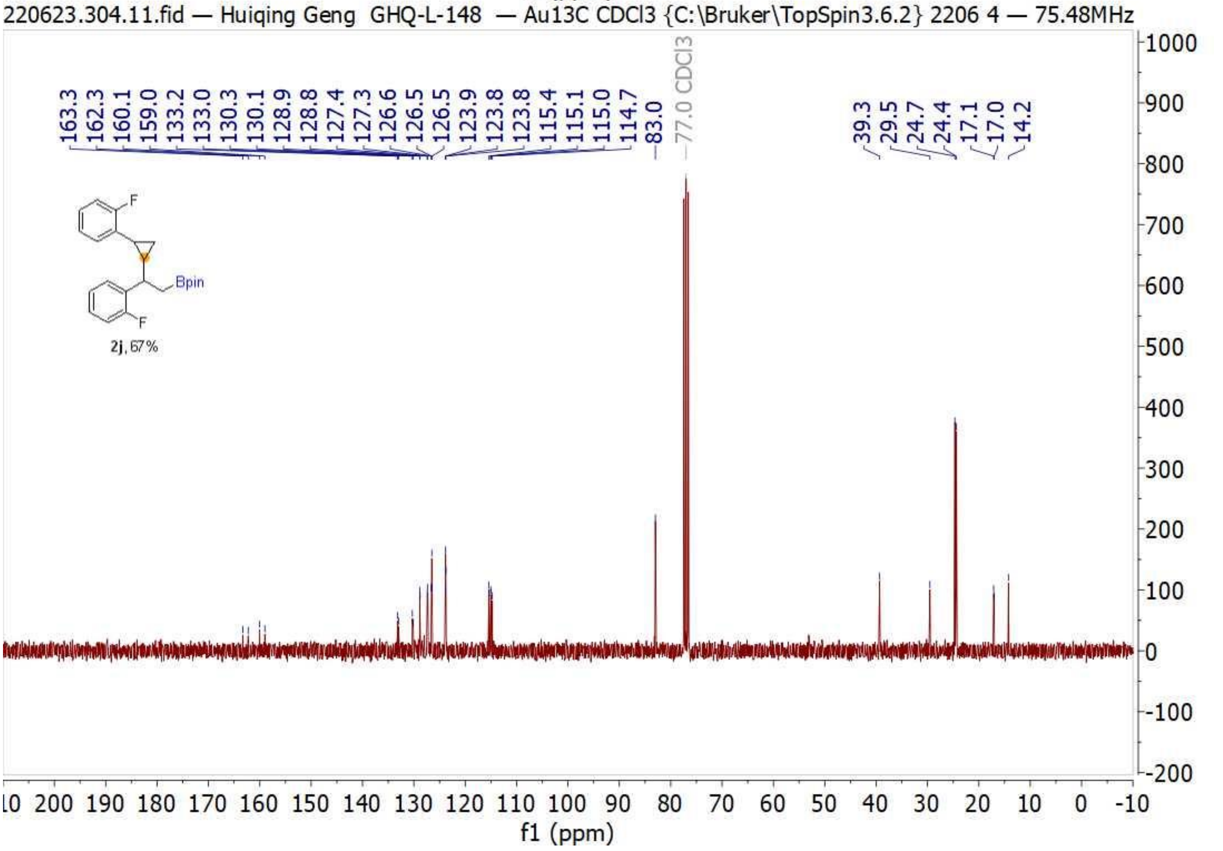
220623.304.11.fid — Huiqing Geng GHQ-L-148 — Au13C CDCl3 {C:\Bruker\TopSpin3.6.2} 2206 4 — 75.48MHz
163.3 162.3 160.1 159.0 133.2 133.0 130.3 130.1 128.9 128.8 127.4 127.3 126.6 126.5 126.5 123.9 123.8 123.8 115.4 115.1 115.0 114.7 83.0 77.0 CDCl3 39.3 29.5 24.7 24.4 17.1 17.0 14.2
F
Bpin
F
2j, 67%
f1 (ppm)

220622.f321.11.fid — Huiqing Geng GHQ-L-148 — 11B CDCl3 {C:\Bruker\TopSpin3.6.2} 2206 21 — 96.32MHz

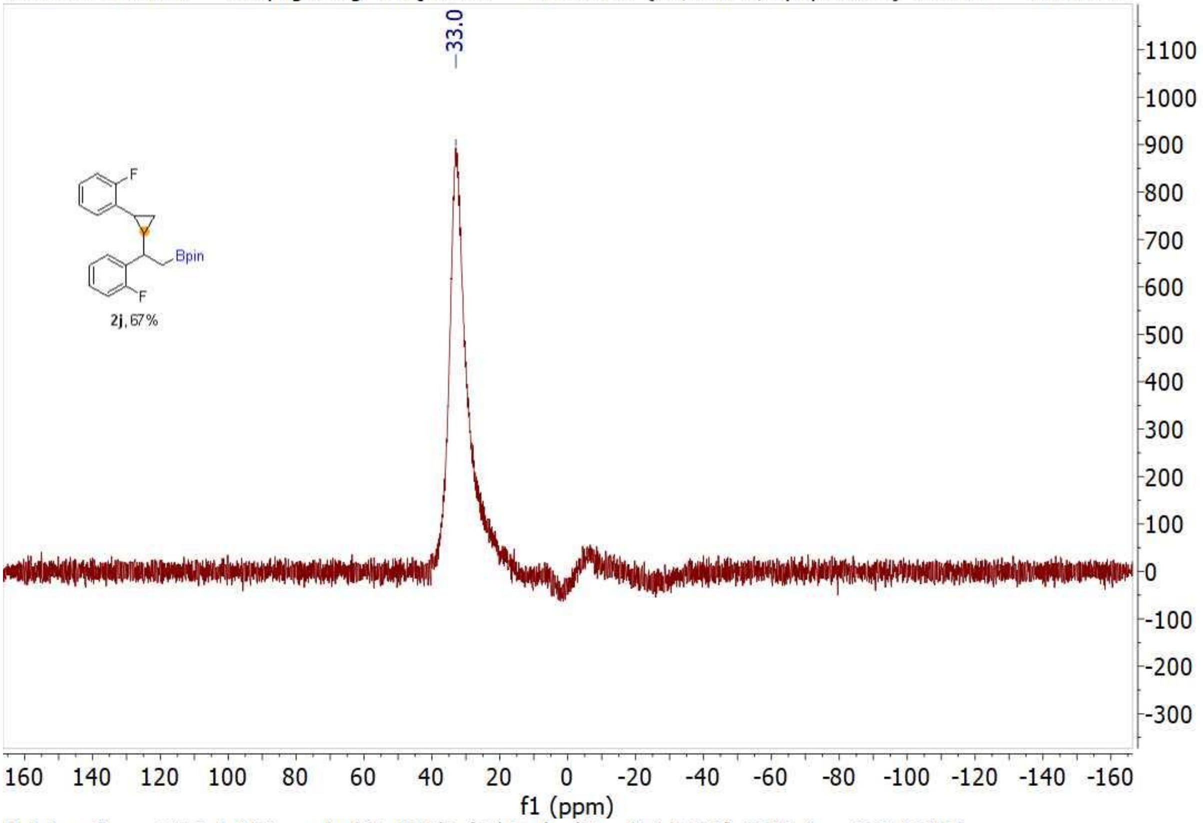

Huiqing Geng GHQ-L-148 — Au19F CDCl3 {C:\Bruker\TopSpin3.6.2} 2206 4 — 282.39MHz

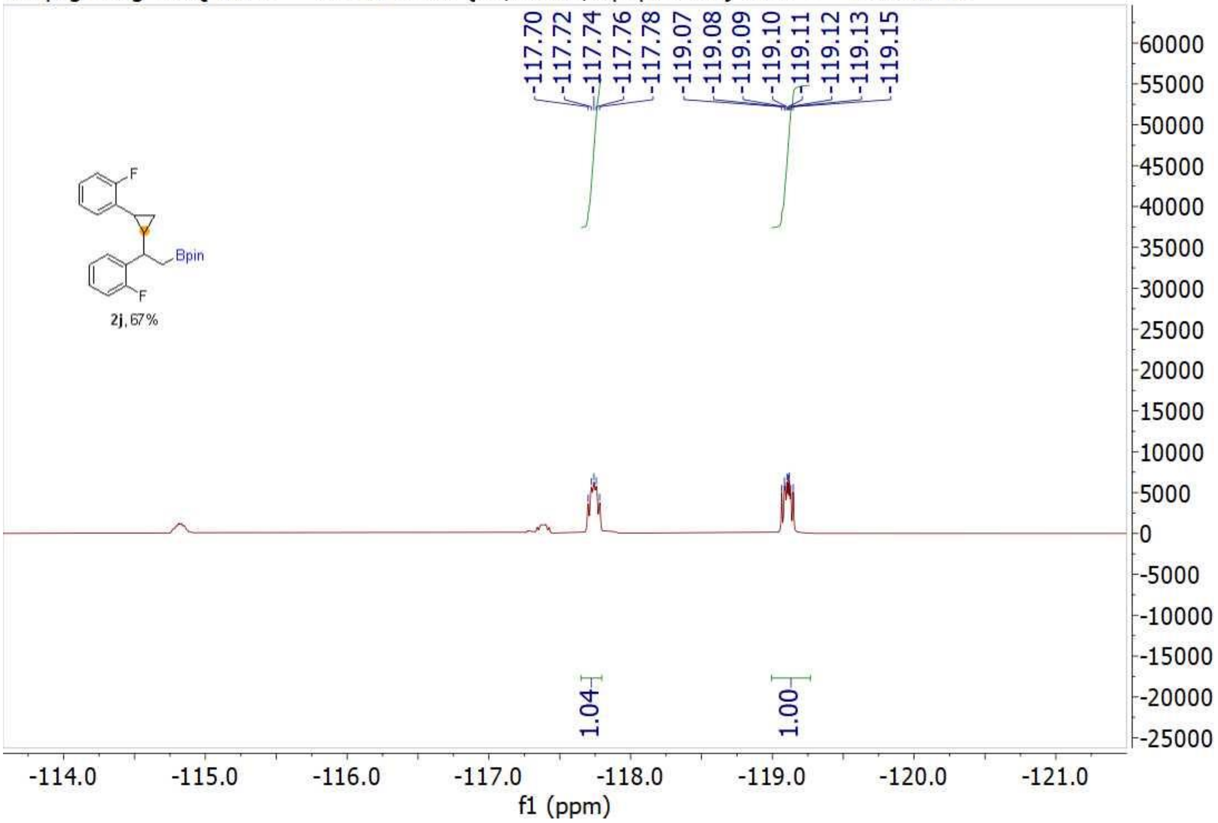

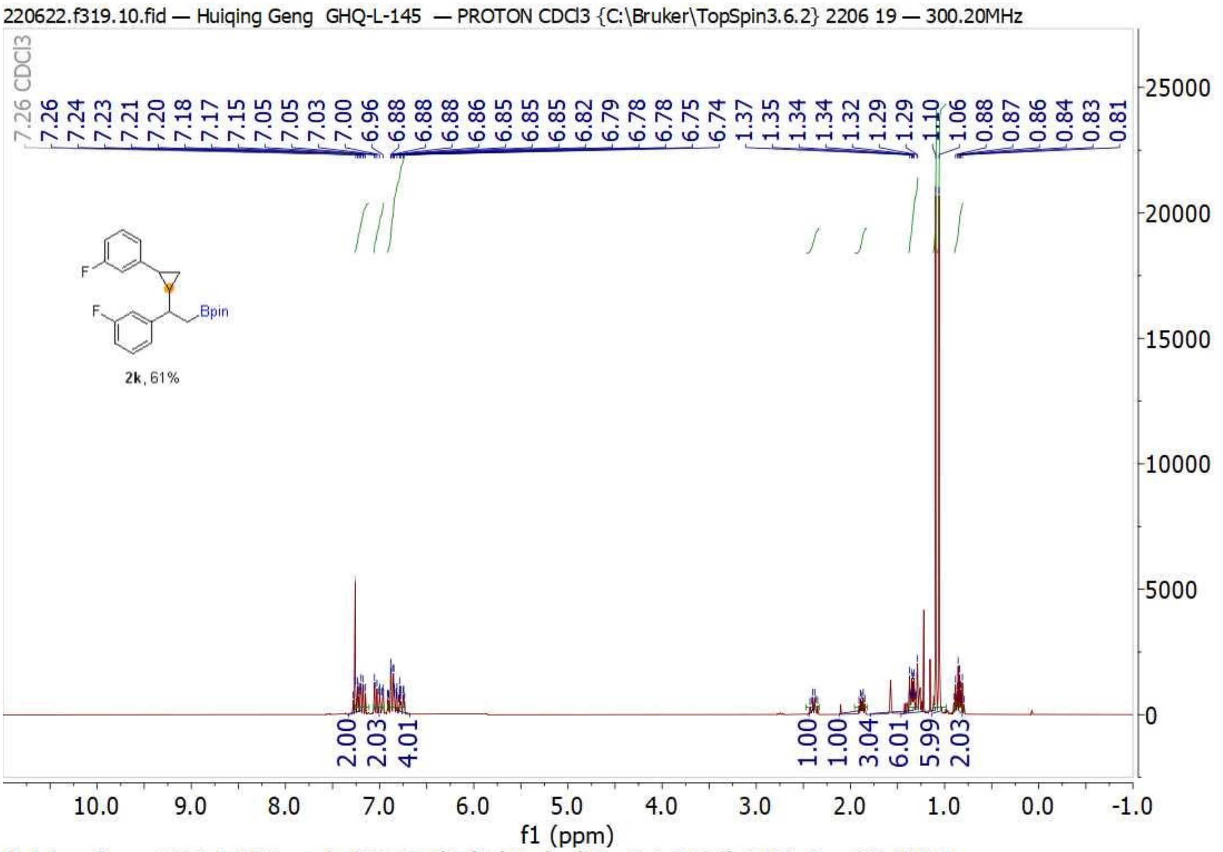
220622.f319.10.fid — Huiqing Geng GHQ-L-145 — PROTON CDCl3 {C:\Bruker\TopSpin3.6.2} 2206 19 — 300.20MHz
7.26 CDCl3
7.26 7.24 7.23 7.21 7.20 7.18 7.17 7.15 7.05 7.05 7.03 7.00 6.96 6.88 6.88 6.86 6.85 6.85 6.85 6.82 6.79 6.78 6.78 6.75 6.74 1.37 1.35 1.34 1.34 1.32 1.29 1.29 1.10 1.06 0.88 0.87 0.86 0.84 0.83 0.81
Bpin
2k, 61%
2.00 2.03 4.01 1.00 1.00 3.04 6.01 5.99 2.03
f1 (ppm)

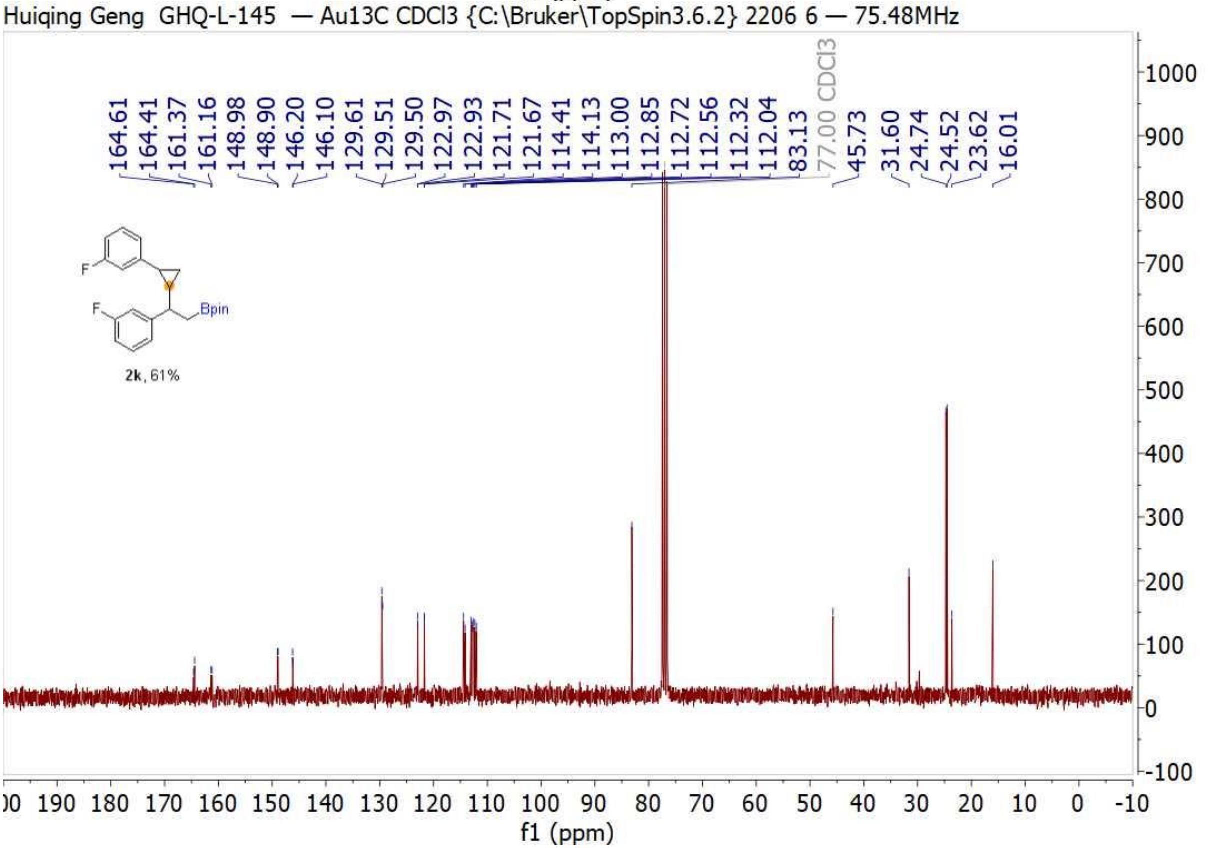
Huiqing Geng GHQ-L-145 — Au13C CDCl3 {C:\Bruker\TopSpin3.6.2} 2206 6 — 75.48MHz
164.61 164.41 161.37 161.16 148.98 148.90 146.20 146.10 129.61 129.51 129.50 122.97 122.93 121.71 121.67 114.41 114.13 113.00 112.85 112.72 112.56 112.32 112.04 83.13 77.00 CDCl3 45.73 31.60 24.74 24.52 23.62 16.01
Bpin
2k, 61%
f1 (ppm)

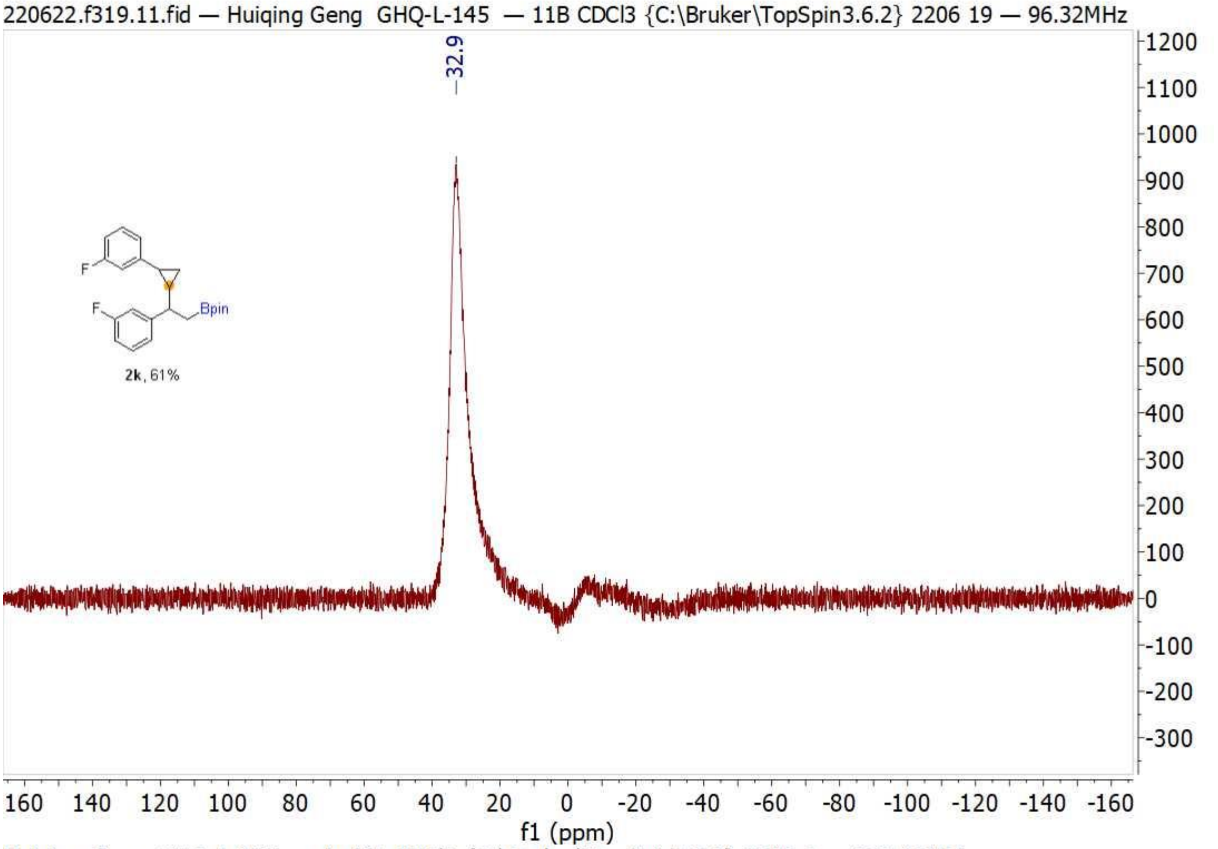
220622.f319.11.fid — Huiqing Geng GHQ-L-145 — 11B CDCl3 {C:\Bruker\TopSpin3.6.2} 2206 19 — 96.32MHz
32.9
Bpin
2k, 61%
f1 (ppm)

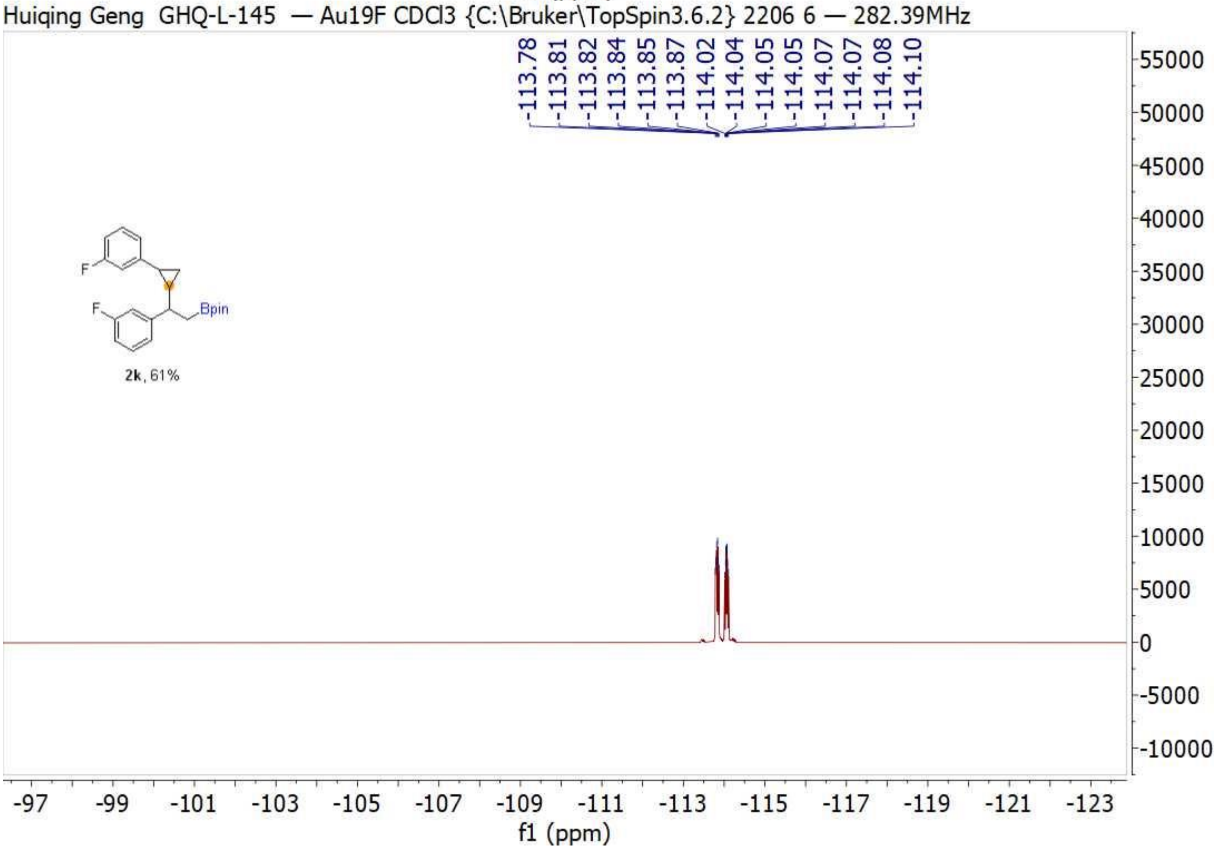
Huiqing Geng GHQ-L-145 — Au19F CDCl3 {C:\Bruker\TopSpin3.6.2} 2206 6 — 282.39MHz
-113.78
-113.81
-113.82
-113.84
-113.85
-113.87
-114.02
-114.04
-114.05
-114.05
-114.07
-114.07
-114.08
-114.10
Bpin
2k, 61%
f1 (ppm)

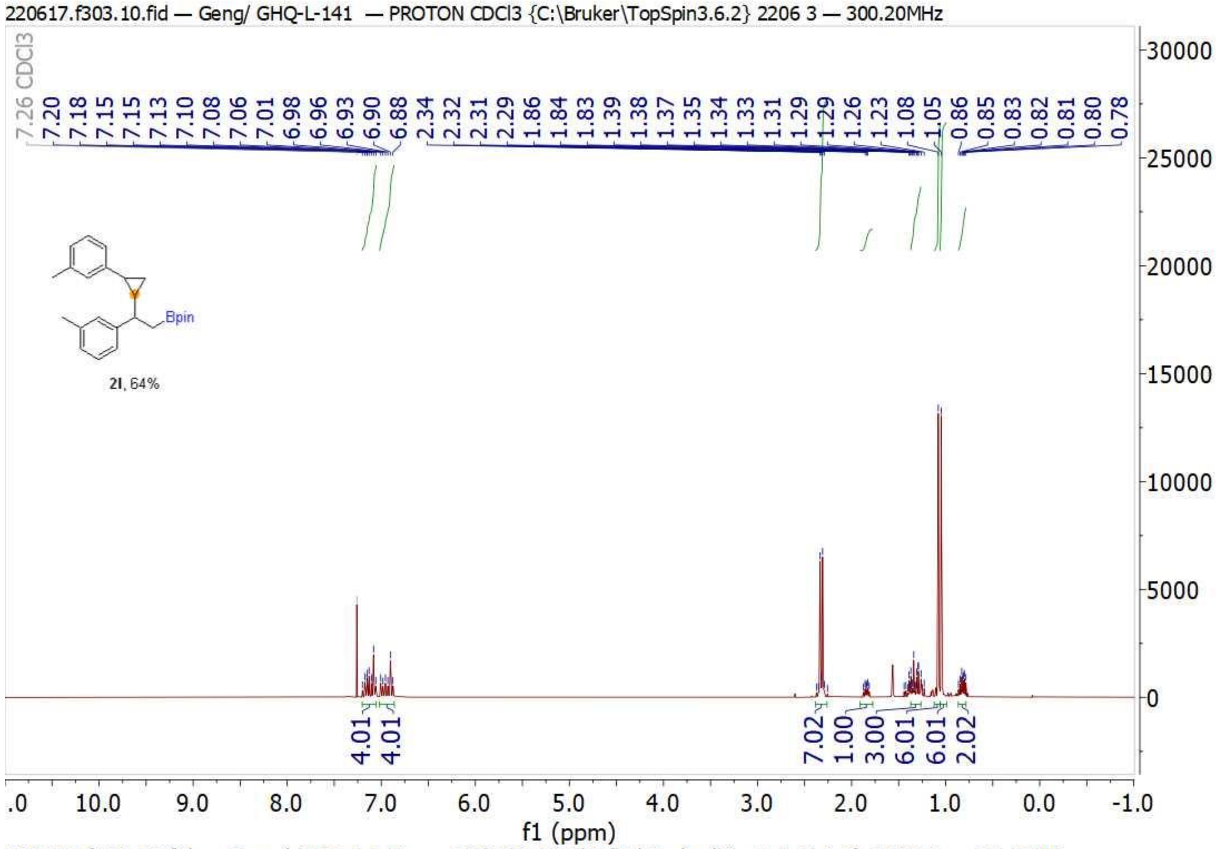
220617.f303.10.fid — Geng/ GHQ-L-141 — PROTON CDCl3 {C:\Bruker\TopSpin3.6.2} 2206 3 — 300.20MHz
7.26 CDCl3
7.20 7.18 7.15 7.15 7.13 7.10 7.08 7.06 7.01 6.98 6.96 6.93 6.90 6.88 2.34 2.32 2.31 2.29 1.86 1.84 1.83 1.39 1.38 1.37 1.35 1.34 1.33 1.31 1.29 1.29 1.26 1.23 1.08 1.05 0.86 0.85 0.83 0.82 0.81 0.80 0.78
Bpin
2l, 64%
4.01 4.01 7.02 1.00 3.00 6.01 6.01 2.02
f1 (ppm)

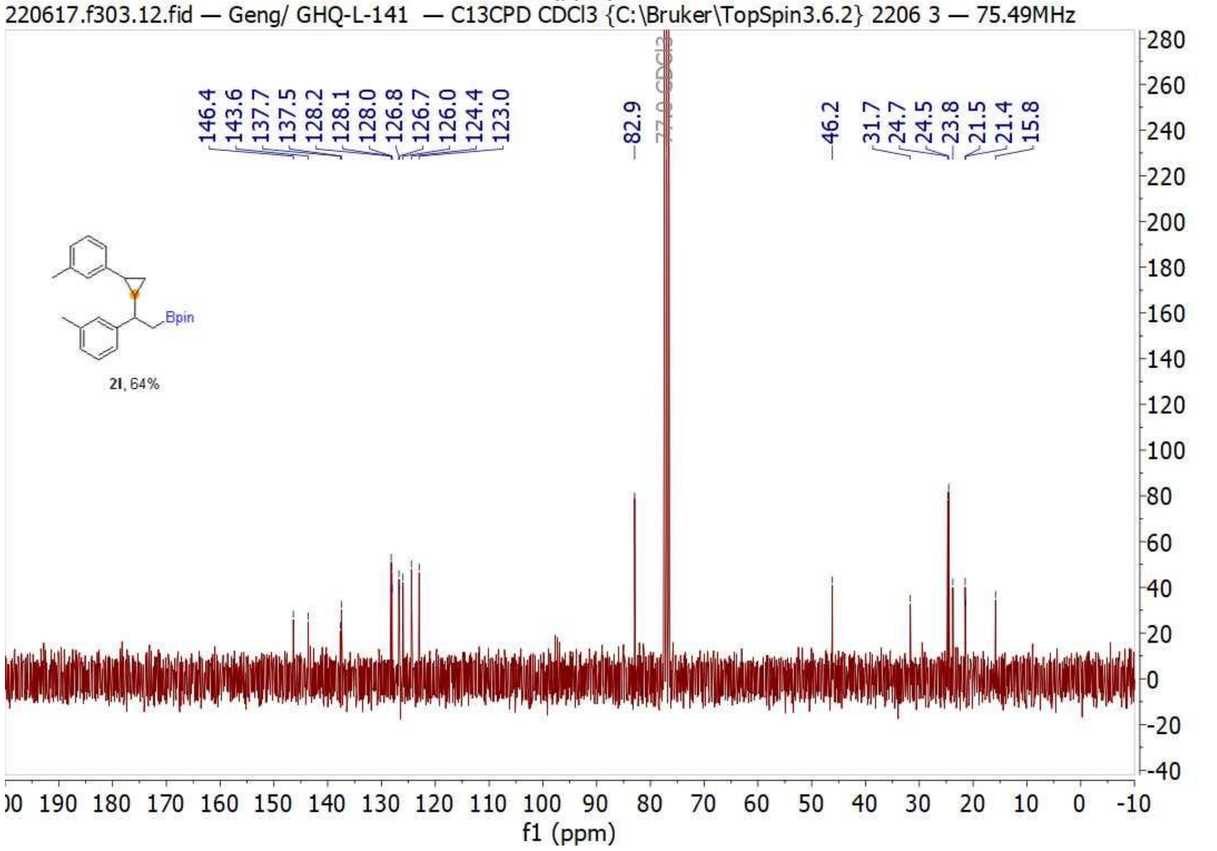
220617.f303.12.fid — Geng/ GHQ-L-141 — C13CPD CDCl3 {C:\Bruker\TopSpin3.6.2} 2206 3 — 75.49MHz
146.4 143.6 137.7 137.5 128.2 128.1 128.0 126.8 126.7 126.0 124.4 123.0
82.9
77.0 CDCl3
46.2
31.7 24.7 24.5 23.8 21.5 21.4 15.8
Bpin
2l, 64%
f1 (ppm)

220617.f303.11.fid — Geng/ GHQ-L-141 — 11B(H-entk) CDCl3 {C:\Bruker\TopSpin3.6.2} 2206 3 — 96.32MHz

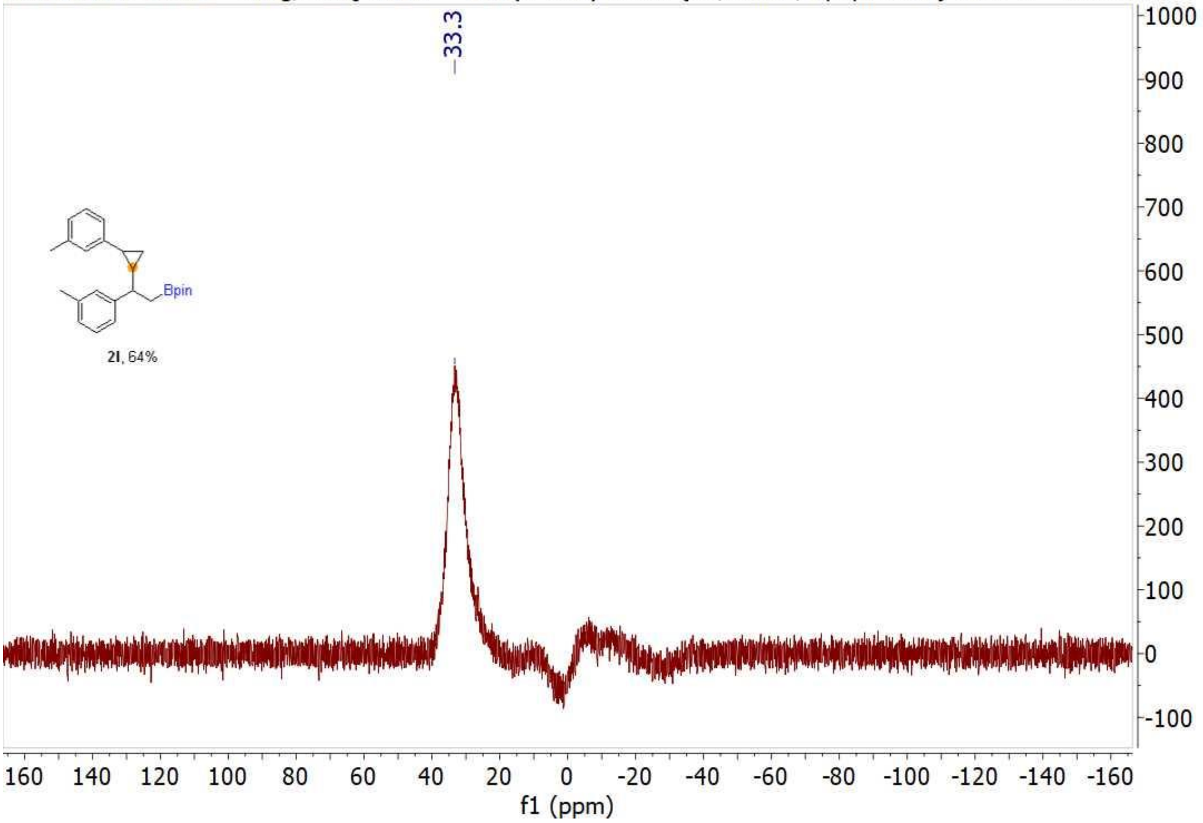

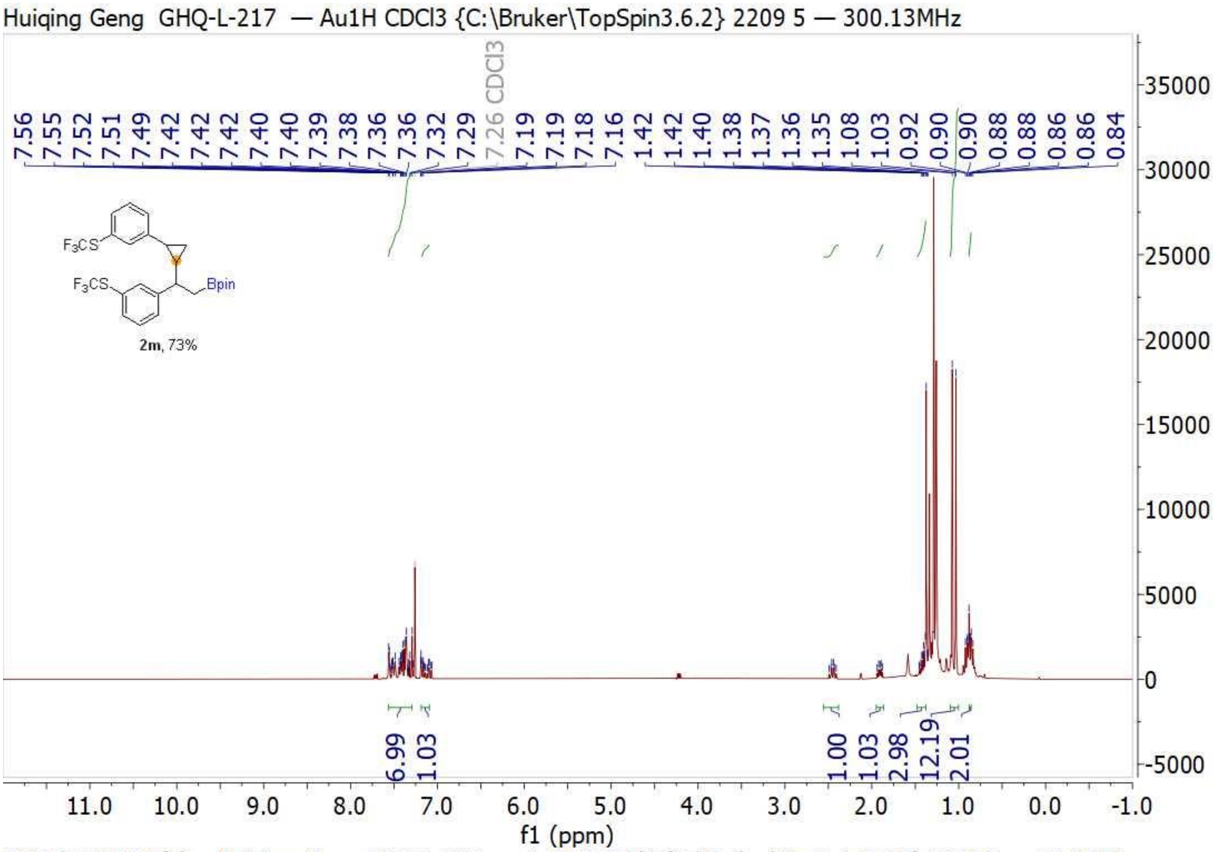
Huiqing Geng GHQ-L-217 — Au1H CDCl3 {C:\Bruker\TopSpin3.6.2} 2209 5 — 300.13MHz
7.56 7.55 7.52 7.51 7.49 7.42 7.42 7.42 7.40 7.40 7.39 7.38 7.36 7.36 7.32 7.29 7.26 CDCl3 7.19 7.19 7.18 7.16 1.42 1.42 1.40 1.38 1.37 1.36 1.35 1.08 1.03 0.92 0.90 0.90 0.88 0.88 0.86 0.86 0.84
F3CS
F3CS
Bpin
2m, 73%
6.99 1.03 1.00 1.03 2.98 12.19 2.01
f1 (ppm)

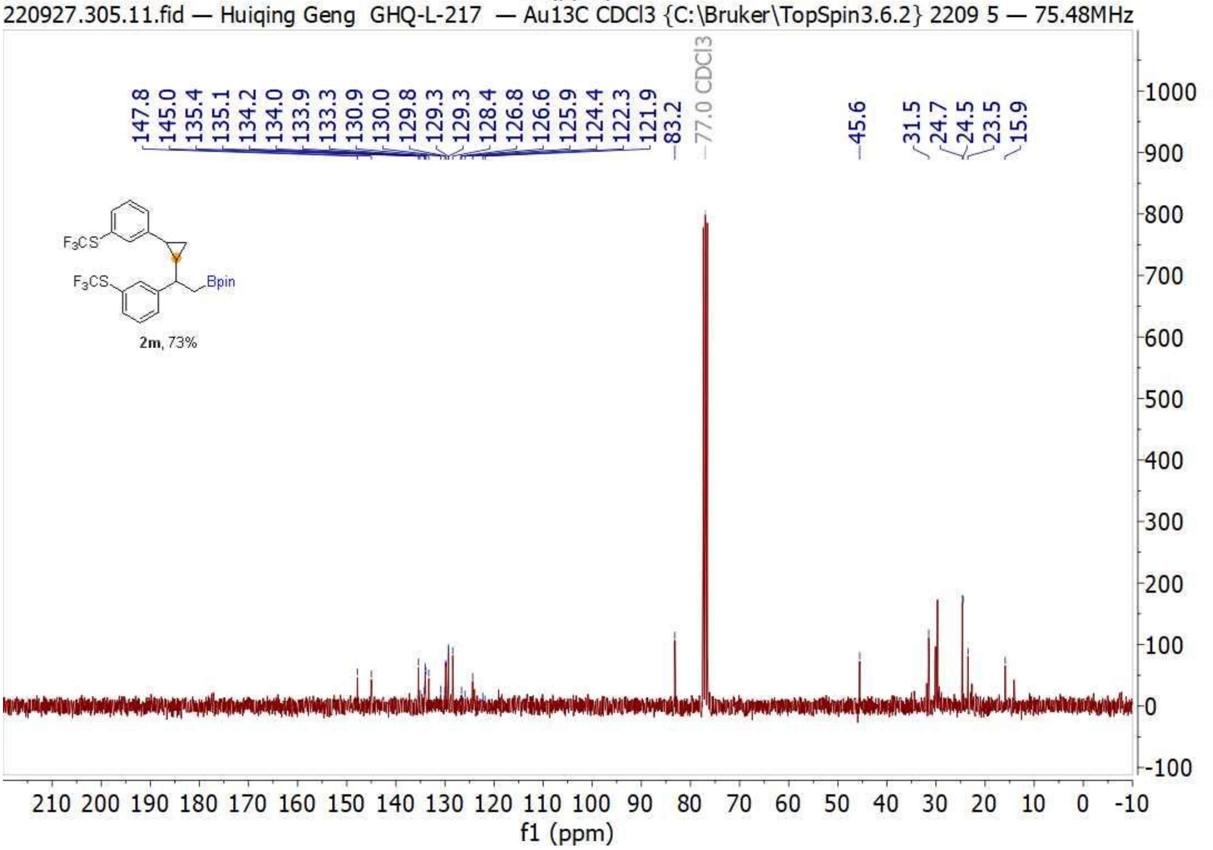
220927.305.11.fid — Huiqing Geng GHQ-L-217 — Au13C CDCl3 {C:\Bruker\TopSpin3.6.2} 2209 5 — 75.48MHz
147.8 145.0 135.4 135.1 134.2 134.0 133.9 133.3 130.9 130.0 129.8 129.3 129.3 128.4 126.8 126.6 125.9 124.4 122.3 121.9 83.2 77.0 CDCl3 45.6 31.5 24.7 24.5 23.5 15.9
F3CS
F3CS
Bpin
2m, 73%
f1 (ppm)

Huiqing Geng GHQ-L-217 — Au11B CDCl3 {C:\Bruker\TopSpin3.6.2} 2209 5 — 96.29MHz

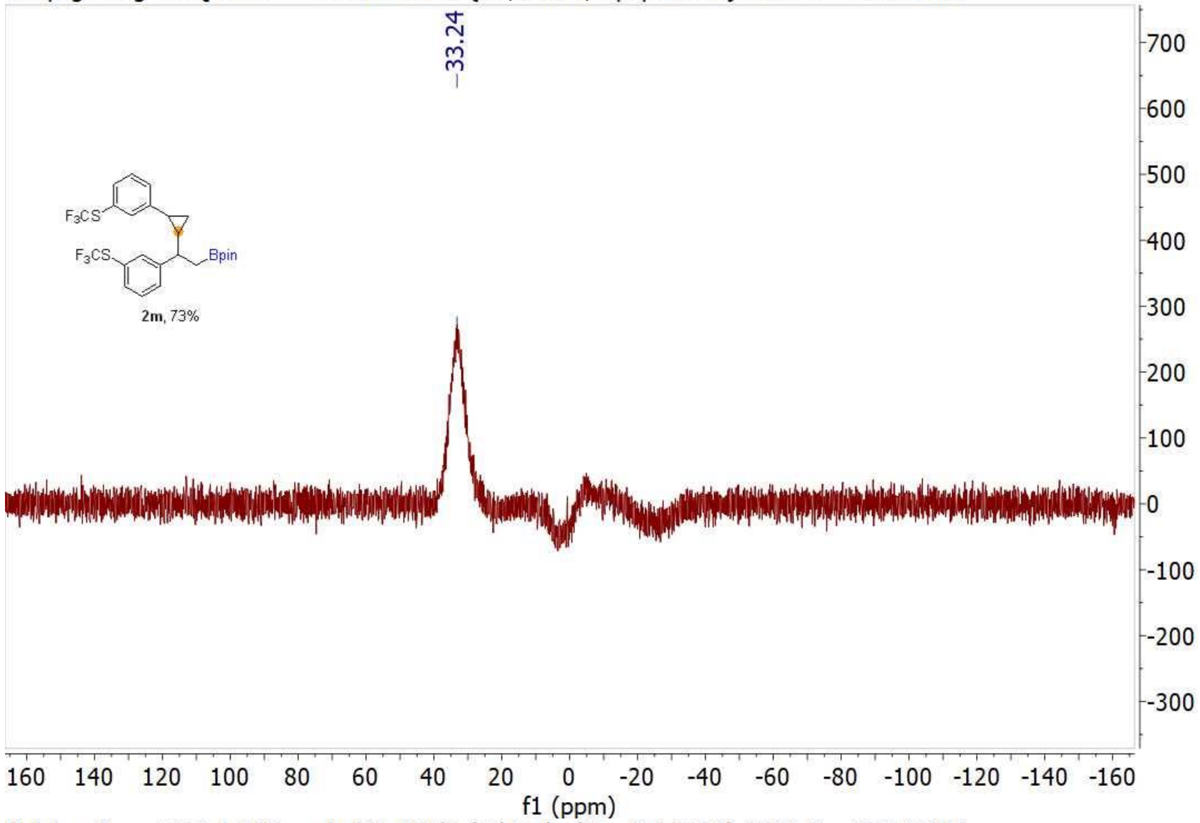

Huiqing Geng GHQ-L-217 — Au19F CDCl3 {C:\Bruker\TopSpin3.6.2} 2302 5 — 282.39MHz

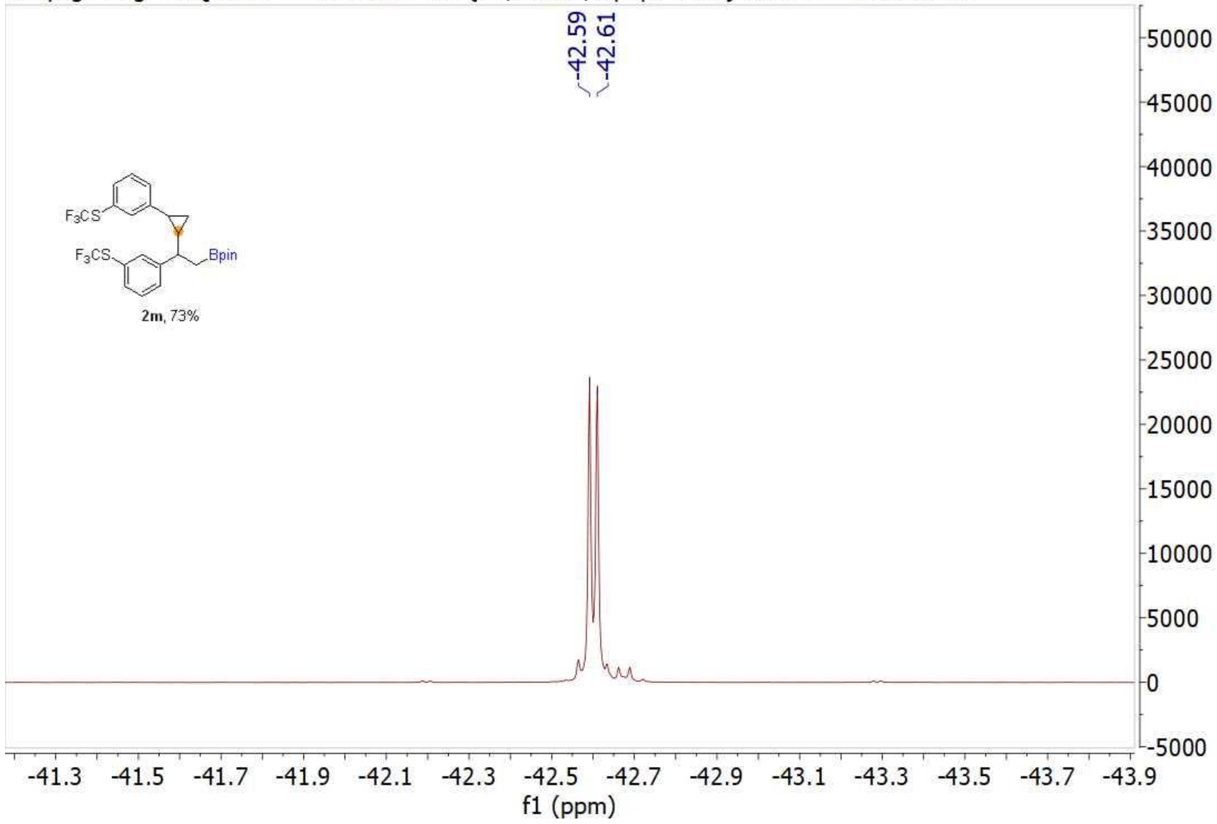

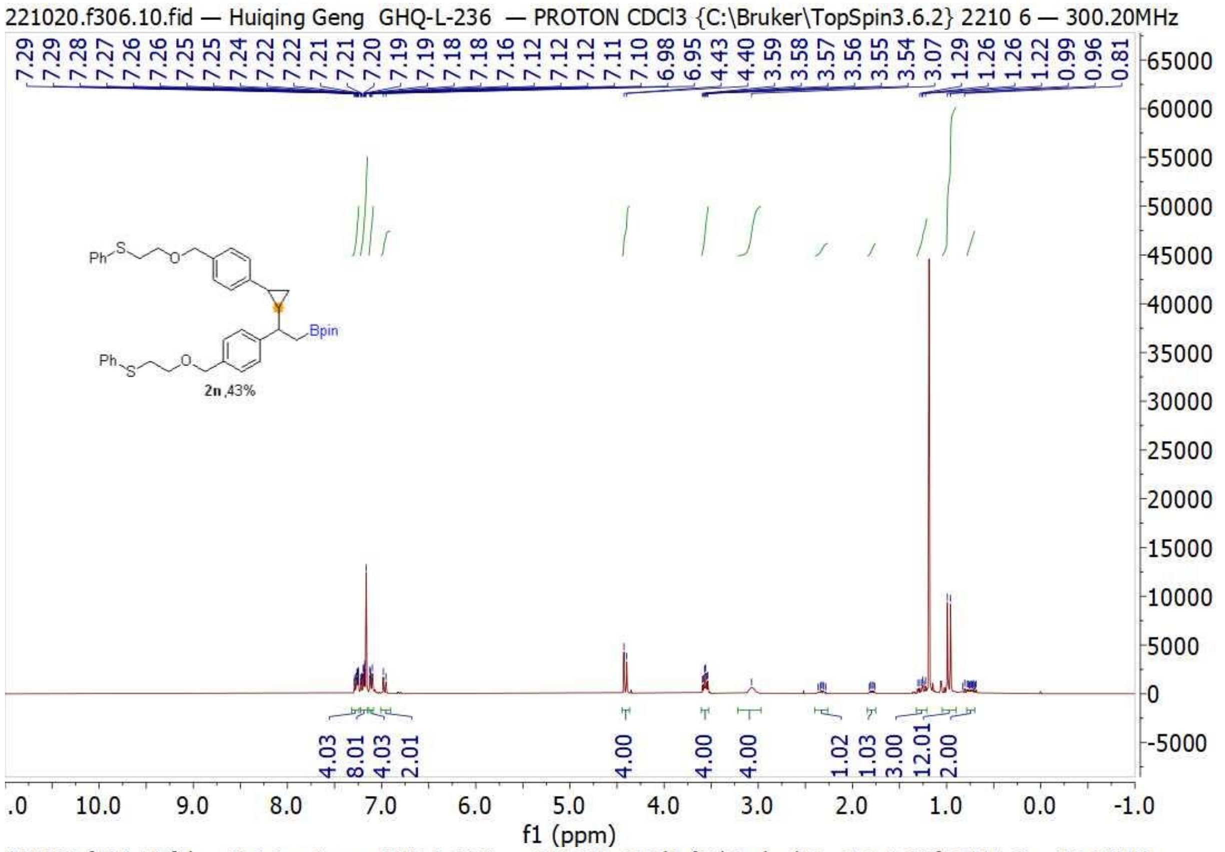

221020.f306.10.fid — Huiqing Geng GHQ-L-236 — PROTON CDCl3 {C:\Bruker\TopSpin3.6.2} 2210 6 — 300.20MHz
7.29 7.29 7.28 7.27 7.26 7.26 7.25 7.25 7.24 7.22 7.22 7.21 7.21 7.20 7.19 7.19 7.18 7.18 7.16 7.12 7.12 7.12 7.11 7.10 6.98 6.95 4.43 4.40 3.59 3.58 3.57 3.56 3.55 3.54 3.07 1.29 1.26 1.26 1.22 0.99 0.96 0.81
Ph S O
Bpin
Ph S O
2n,43%
4.03 8.01 4.03 2.01 4.00 4.00 4.00 1.02 1.03 3.00 12.01 2.00
f1 (ppm)

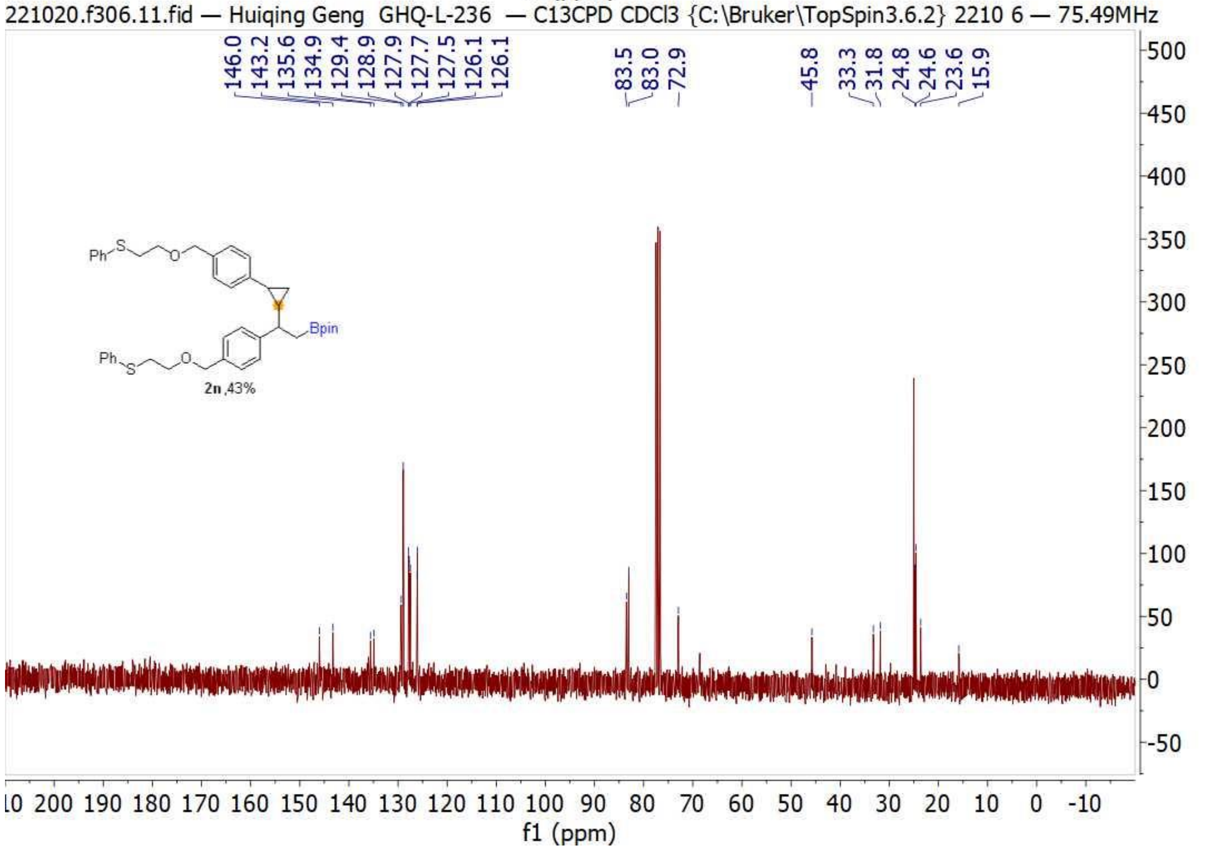

221020.f306.11.fid — Huiqing Geng GHQ-L-236 — C13CPD CDCl3 {C:\Bruker\TopSpin3.6.2} 2210 6 — 75.49MHz
146.0 143.2 135.6 134.9 129.4 128.9 127.9 127.7 127.5 126.1 126.1
83.5 83.0 72.9
45.8 33.3 31.8 24.8 24.6 23.6 15.9
Ph S O
Bpin
Ph S O
2n,43%
f1 (ppm)

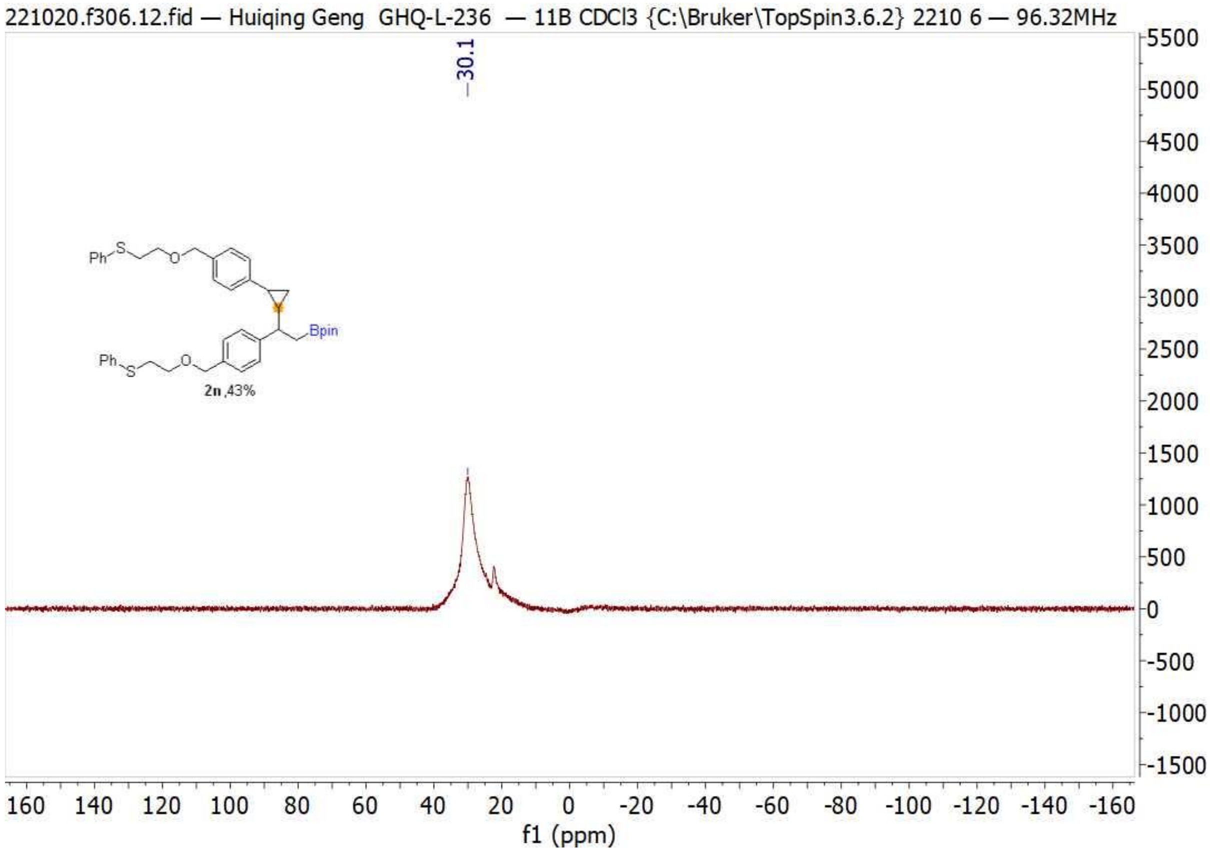

221020.f306.12.fid — Huiqing Geng GHQ-L-236 — 11B CDCl3 {C:\Bruker\TopSpin3.6.2} 2210 6 — 96.32MHz
30.1
Ph S O
Bpin
Ph S O
2n ,43%
5500
5000
4500
4000
3500
3000
2500
2000
1500
1000
500
0
-500
-1000
-1500
160 140 120 100 80 60 40 20 0 -20 -40 -60 -80 -100 -120 -140 -160
f1 (ppm)

221103.306.10.fid — Huiqing Geng GHQ-L-245-B — Au1H CDCl3 {C:\Bruker\TopSpin3.6.2} 2211 6 — 300.13MHz

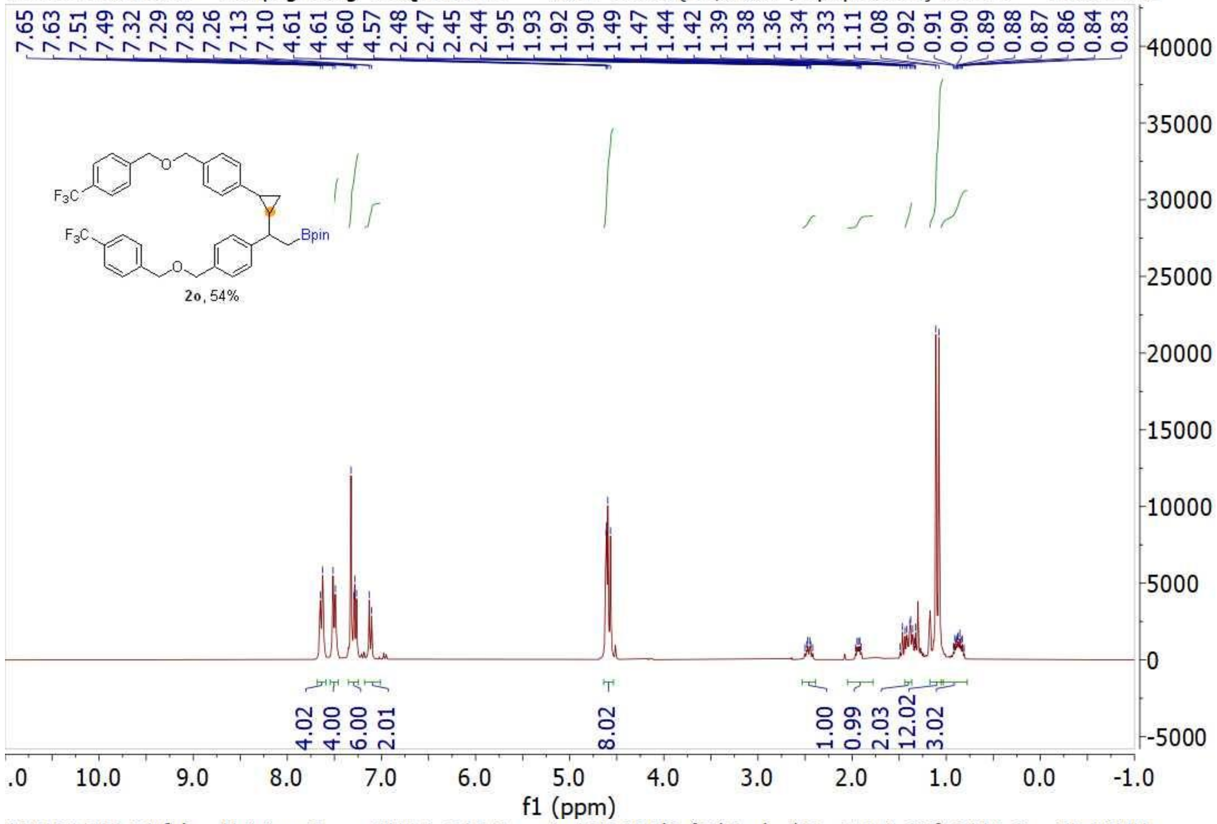

221103.306.11.fid — Huiqing Geng GHQ-L-245-B — Au13C CDCl3 {C:\Bruker\TopSpin3.6.2} 2211 6 — 75.48MHz

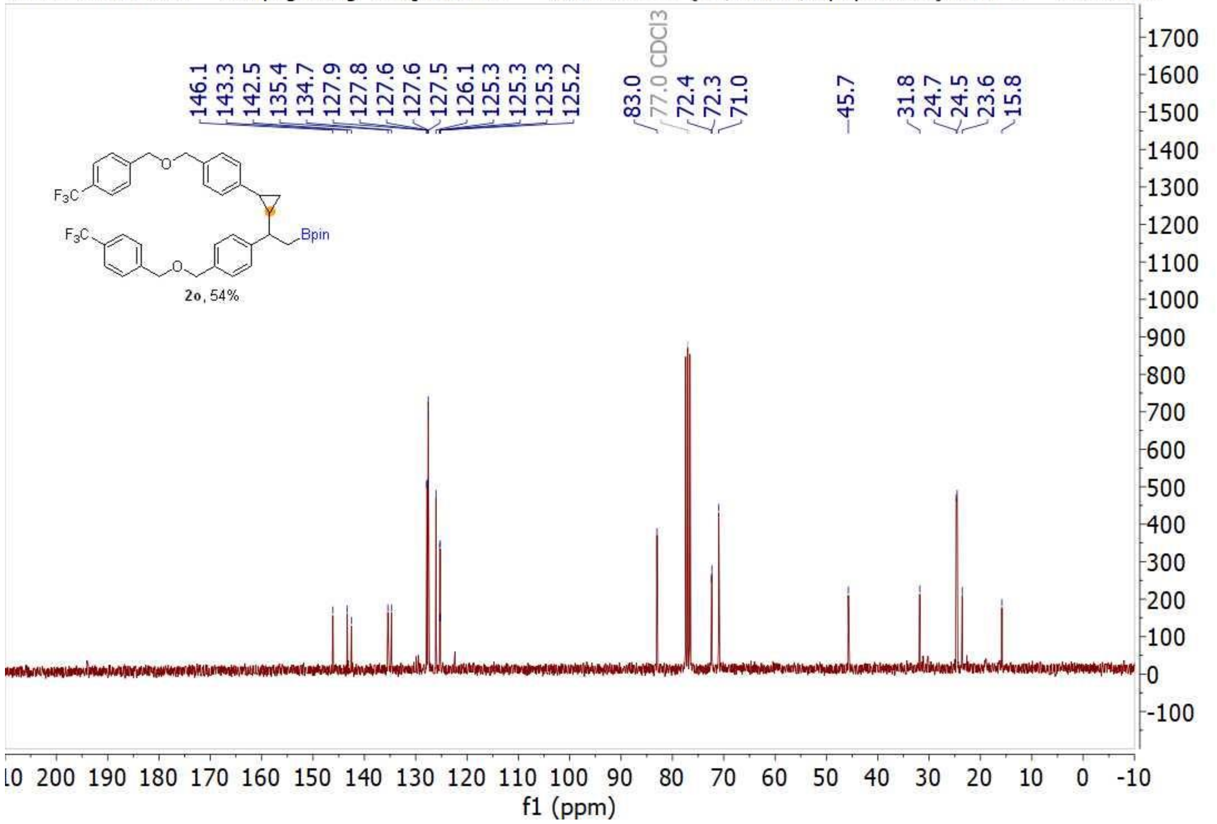

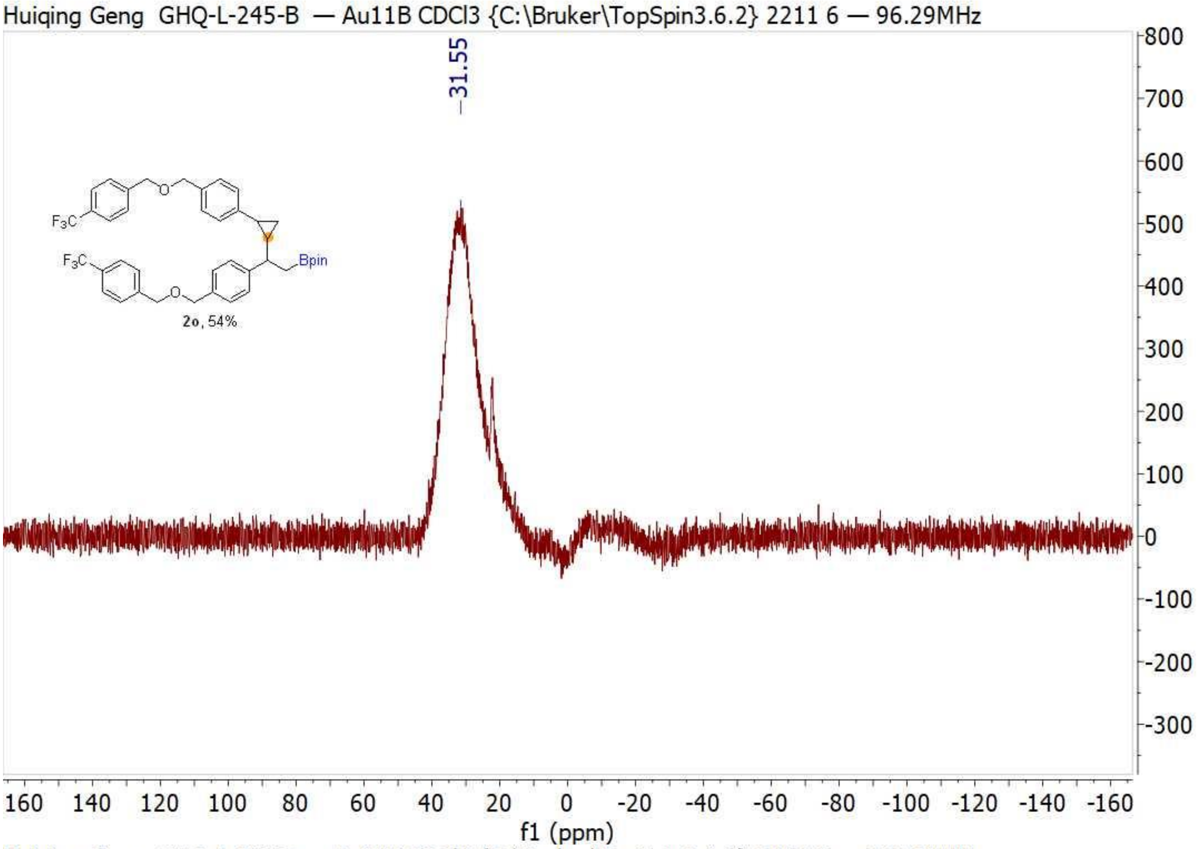
Huiqing Geng GHQ-L-245-B — Au11B CDCl3 {C:\Bruker\TopSpin3.6.2} 2211 6 — 96.29MHz
31.55
F3C
Bpin
2o, 54%
f1 (ppm)

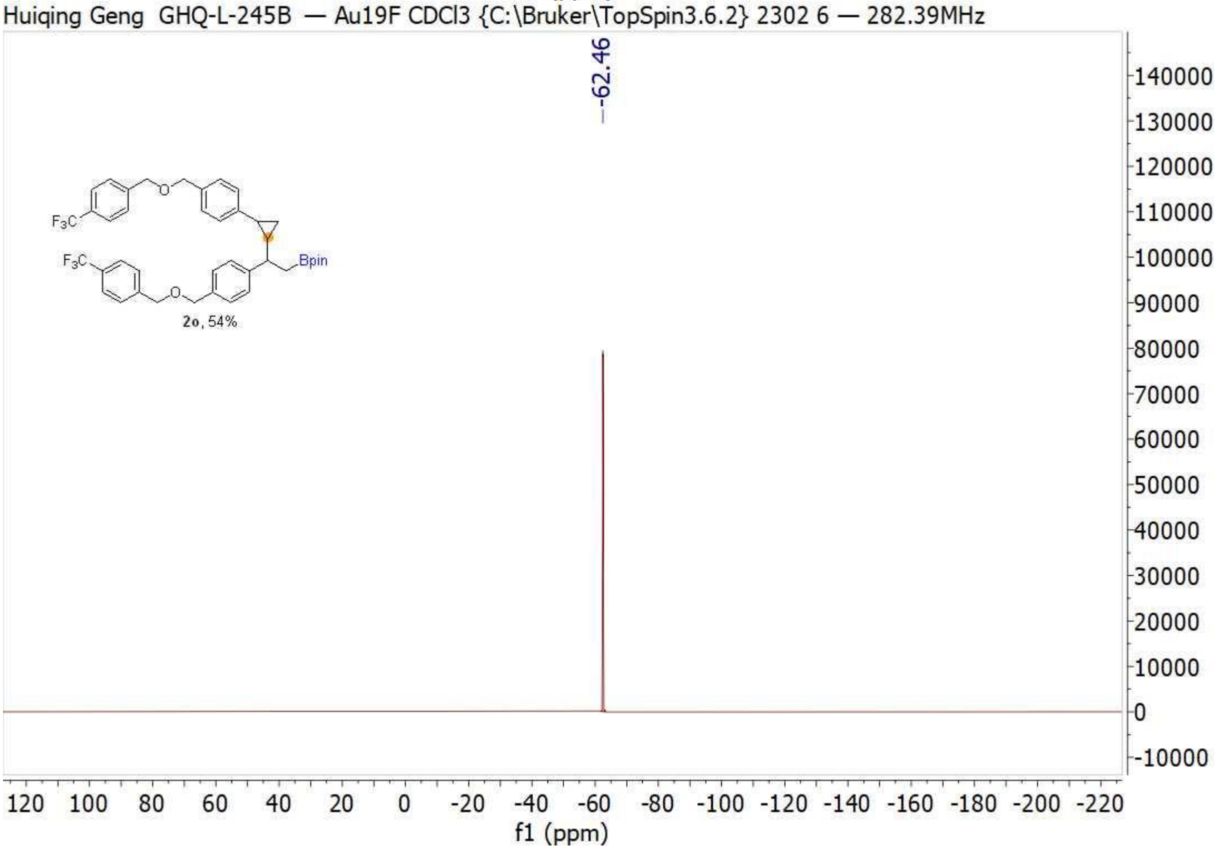
Huiqing Geng GHQ-L-245B — Au19F CDCl3 {C:\Bruker\TopSpin3.6.2} 2302 6 — 282.39MHz
-62.46
F3C
Bpin
2o, 54%
f1 (ppm)

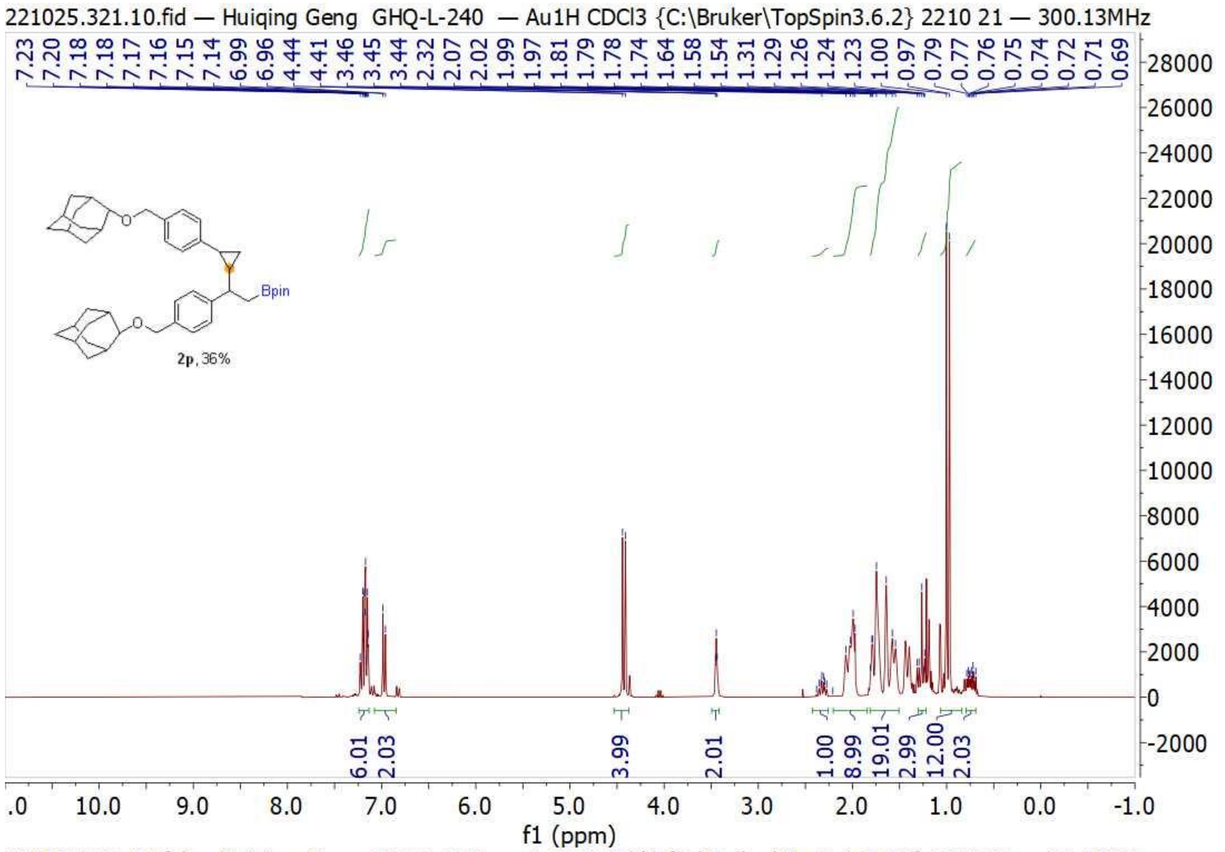
221025.321.10.fid — Huiqing Geng GHQ-L-240 — Au1H CDCl3 {C:\Bruker\TopSpin3.6.2} 2210 21 — 300.13MHz
7.23 7.20 7.18 7.18 7.17 7.16 7.15 7.14 6.99 6.96 4.44 4.41 3.46 3.45 3.44 2.32 2.07 2.02 1.99 1.97 1.81 1.79 1.78 1.74 1.64 1.58 1.54 1.31 1.29 1.26 1.24 1.23 1.00 0.97 0.79 0.77 0.76 0.75 0.74 0.72 0.71 0.69
Bpin
2p, 36%
6.01 2.03 3.99 2.01 1.00 8.99 19.01 2.99 12.00 2.03
f1 (ppm)

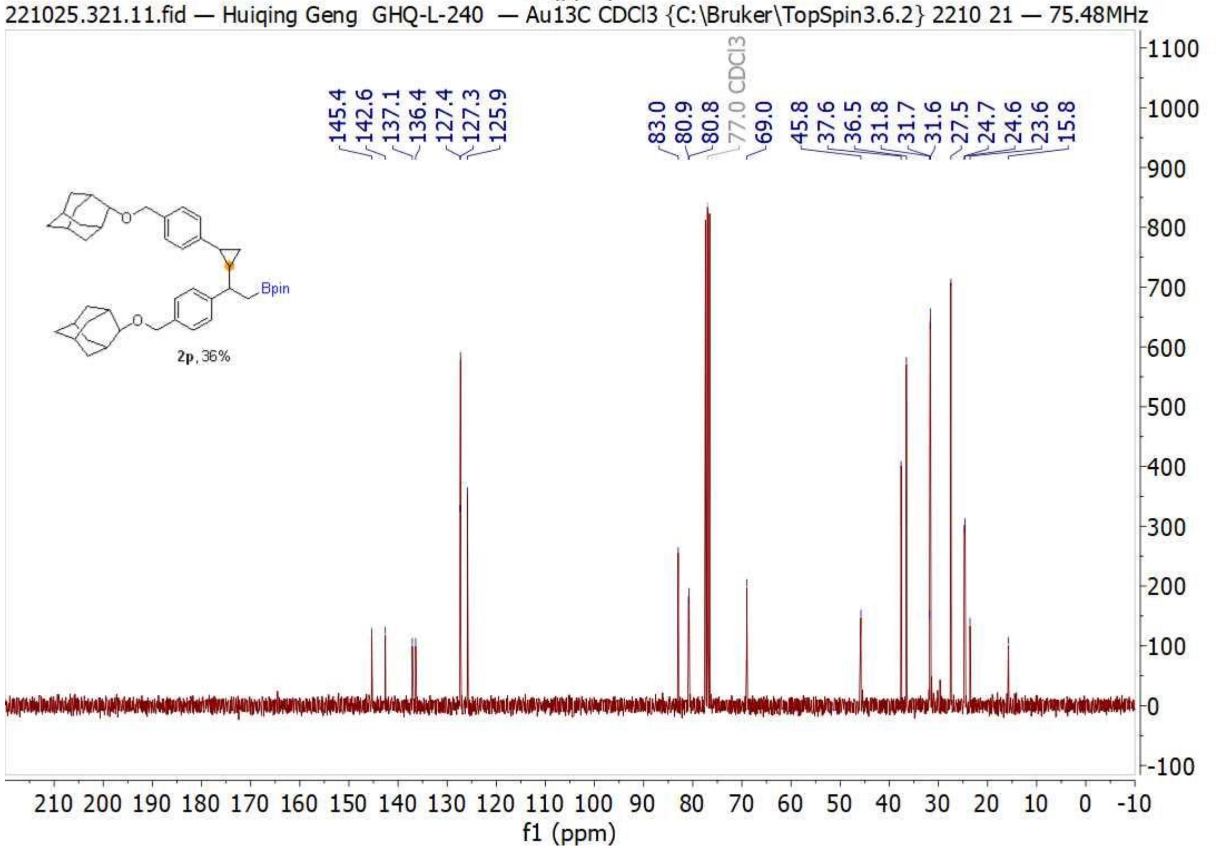
221025.321.11.fid — Huiqing Geng GHQ-L-240 — Au13C CDCl3 {C:\Bruker\TopSpin3.6.2} 2210 21 — 75.48MHz
145.4 142.6 137.1 136.4 127.4 127.3 125.9 83.0 80.9 80.8 77.0 CDCl3 69.0 45.8 37.6 36.5 31.8 31.7 31.6 27.5 24.7 24.6 23.6 15.8
Bpin
2p, 36%
f1 (ppm)

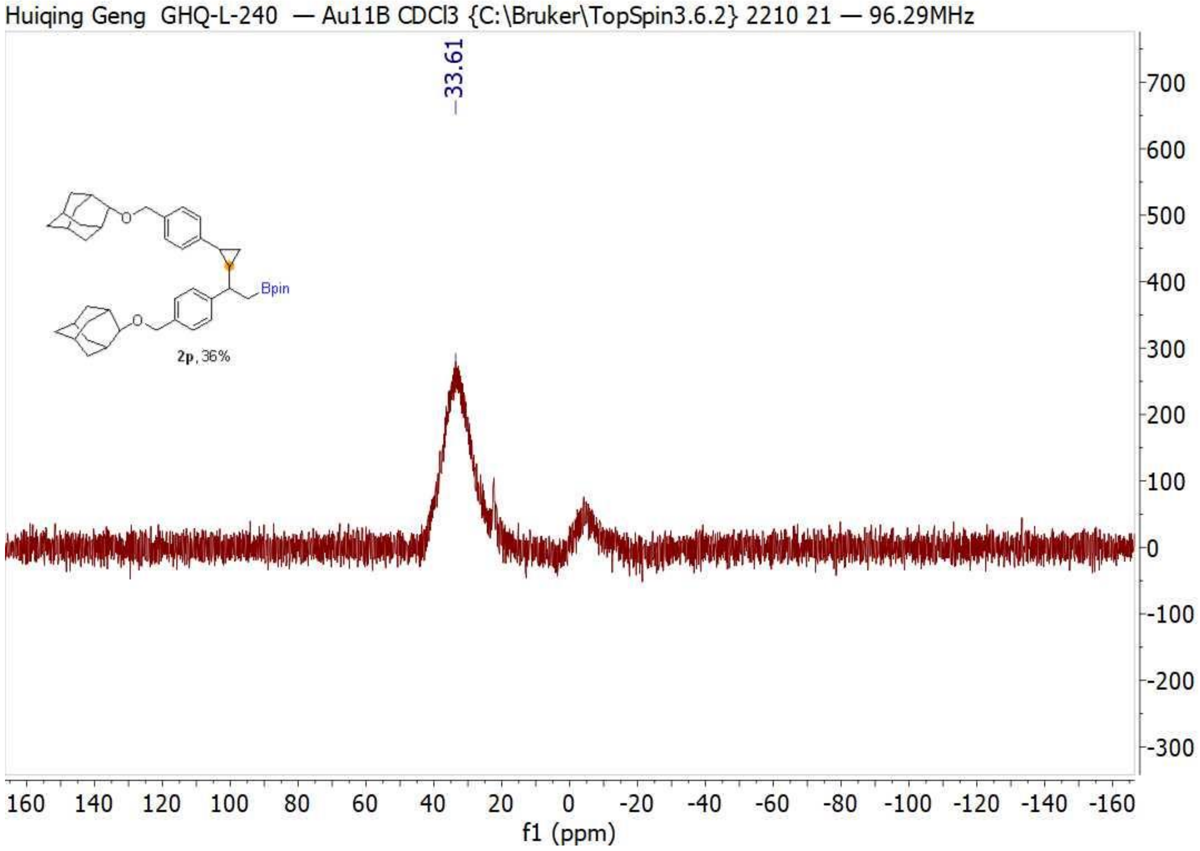
Huiqing Geng GHQ-L-240 — Au11B CDCl3 {C:\Bruker\TopSpin3.6.2} 2210 21 — 96.29MHz
33.61
Bpin
2p, 36%
700
600
500
400
300
200
100
0
-100
-200
-300
160 140 120 100 80 60 40 20 0 -20 -40 -60 -80 -100 -120 -140 -160
f1 (ppm)

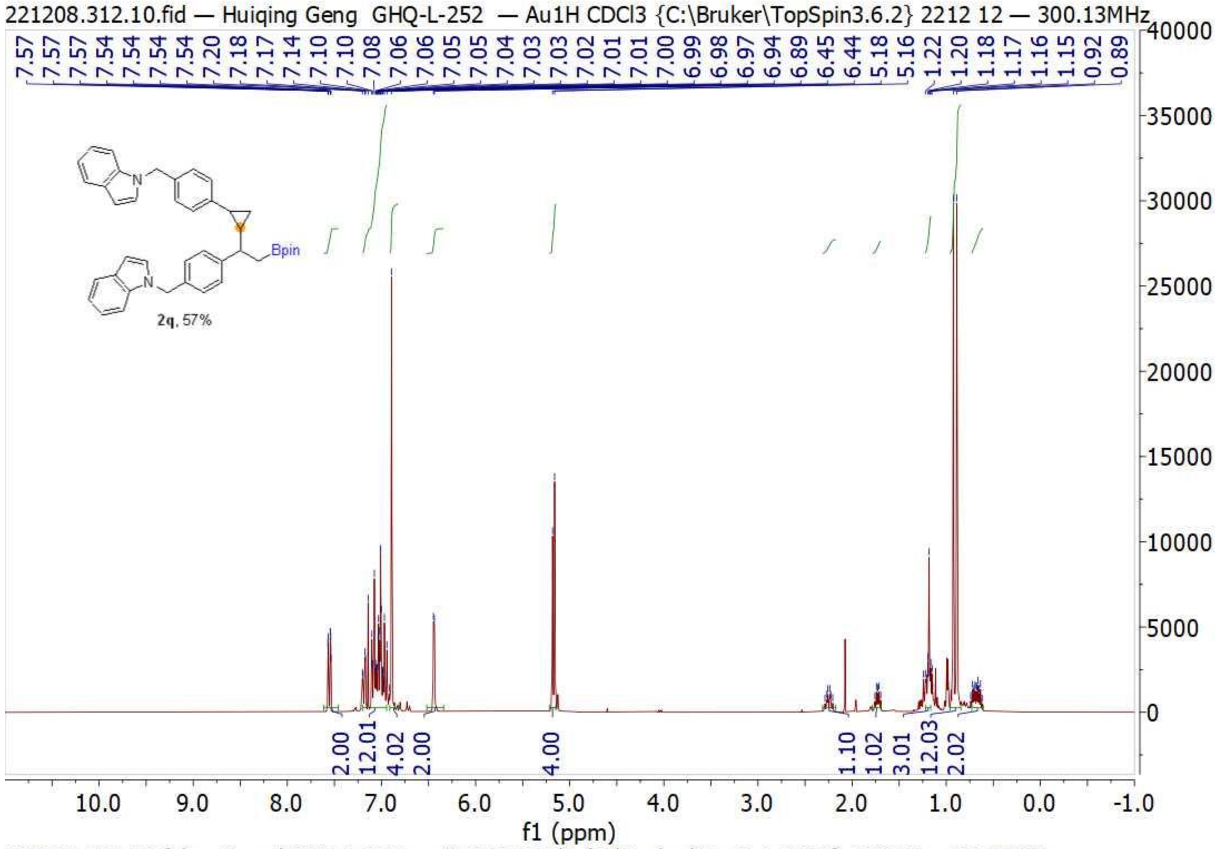
221208.312.10.fid — Huiqing Geng GHQ-L-252 — Au1H CDCl3 {C:\Bruker\TopSpin3.6.2} 2212 12 — 300.13MHz
7.57 7.57 7.57 7.54 7.54 7.54 7.54 7.20 7.18 7.17 7.14 7.10 7.10 7.08 7.06 7.06 7.05 7.05 7.04 7.03 7.03 7.02 7.01 7.01 7.00 6.99 6.98 6.97 6.94 6.89 6.45 6.44 5.18 5.16 1.22 1.20 1.18 1.17 1.16 1.15 0.92 0.89
Bpin
2q, 57%
2.00 12.01 4.02 2.00 4.00 1.10 1.02 3.01 12.03 2.02
f1 (ppm)
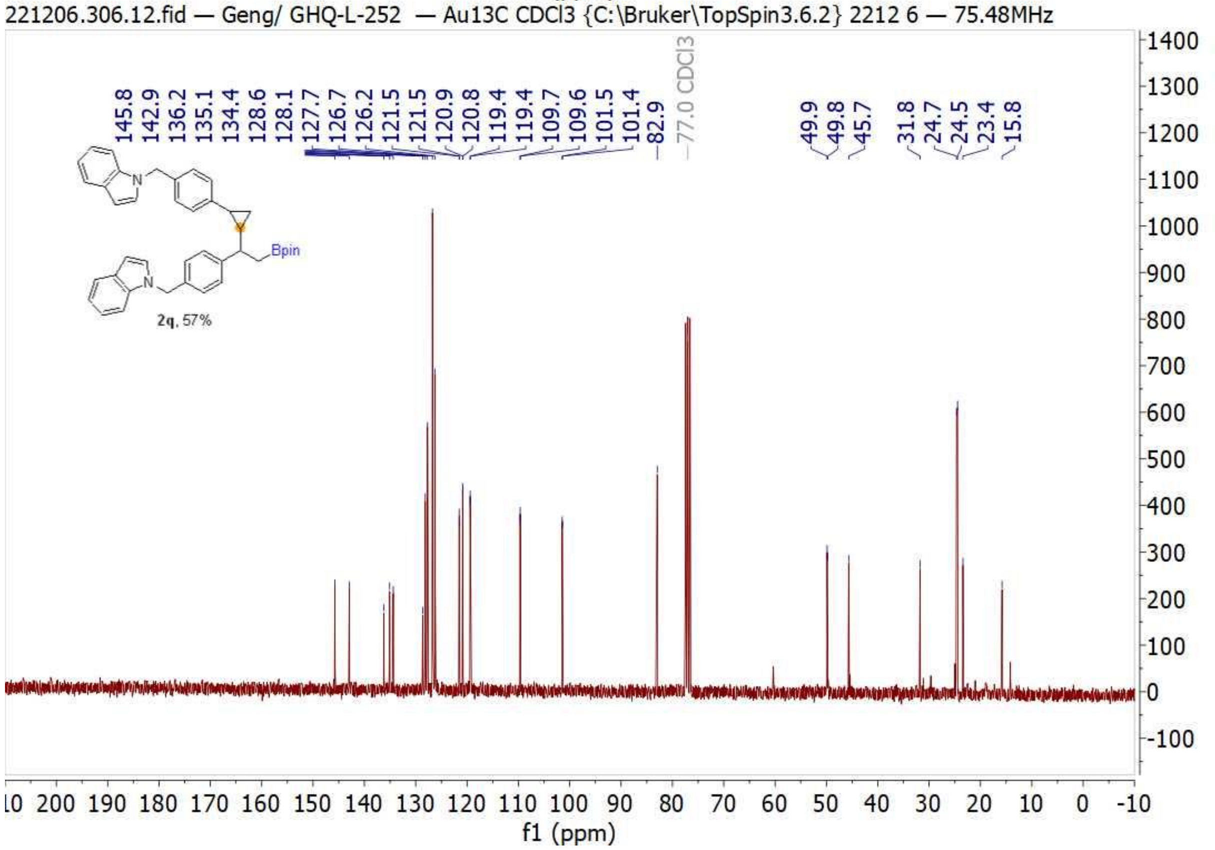
221206.306.12.fid — Geng/ GHQ-L-252 — Au13C CDCl3 {C:\Bruker\TopSpin3.6.2} 2212 6 — 75.48MHz
145.8 142.9 136.2 135.1 134.4 128.6 128.1 127.7 126.7 126.2 121.5 121.5 120.9 120.8 119.4 119.4 109.7 109.6 101.5 101.4 82.9 77.0 CDCl3 49.9 49.8 45.7 31.8 24.7 24.5 23.4 15.8
Bpin
2q, 57%
f1 (ppm)

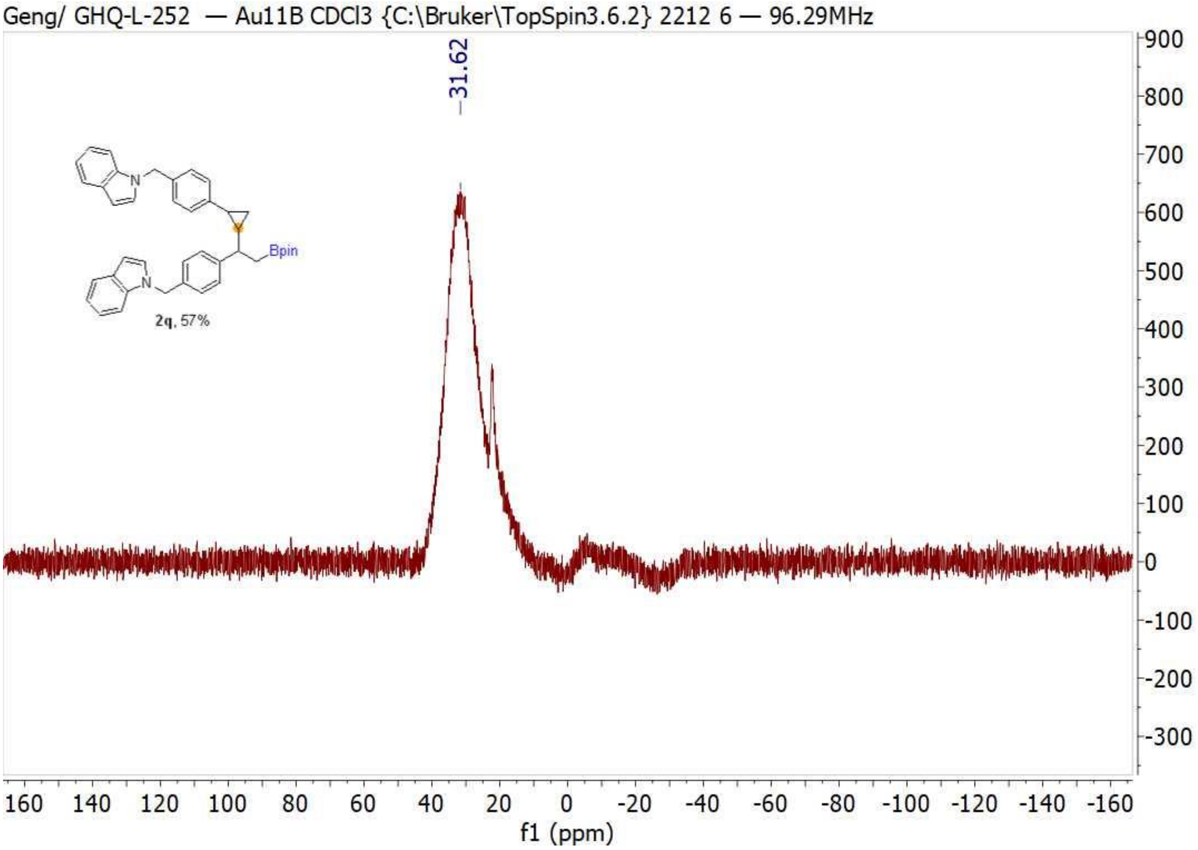
Geng/ GHQ-L-252 — Au11B CDCl3 {C:\Bruker\TopSpin3.6.2} 2212 6 — 96.29MHz
31.62
Bpin
2q, 57%
900
800
700
600
500
400
300
200
100
0
-100
-200
-300
160 140 120 100 80 60 40 20 0 -20 -40 -60 -80 -100 -120 -140 -160
f1 (ppm)

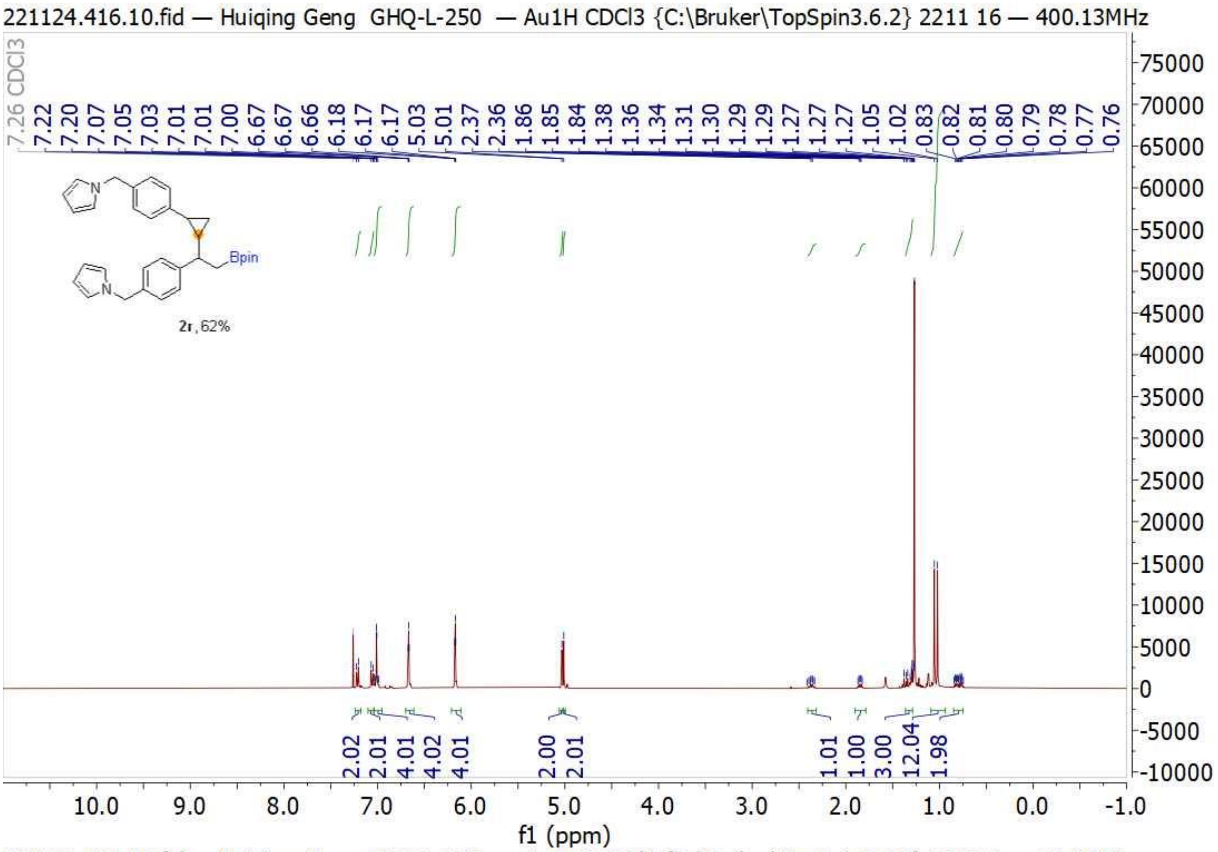
221124.416.10.fid — Huiqing Geng GHQ-L-250 — Au1H CDCl3 {C:\Bruker\TopSpin3.6.2} 2211 16 — 400.13MHz
7.26 CDCl3
7.22 7.20 7.07 7.05 7.03 7.01 7.01 7.00 6.67 6.67 6.66 6.18 6.17 6.17 5.03 5.01 2.37 2.36 1.86 1.85 1.84 1.38 1.36 1.34 1.31 1.30 1.29 1.29 1.27 1.27 1.27 1.05 1.02 0.83 0.82 0.81 0.80 0.79 0.78 0.77 0.76
Bpin
2r, 62%
2.02 2.01 4.01 4.02 4.01 2.00 2.01 1.01 1.00 3.00 12.04 1.98
f1 (ppm)

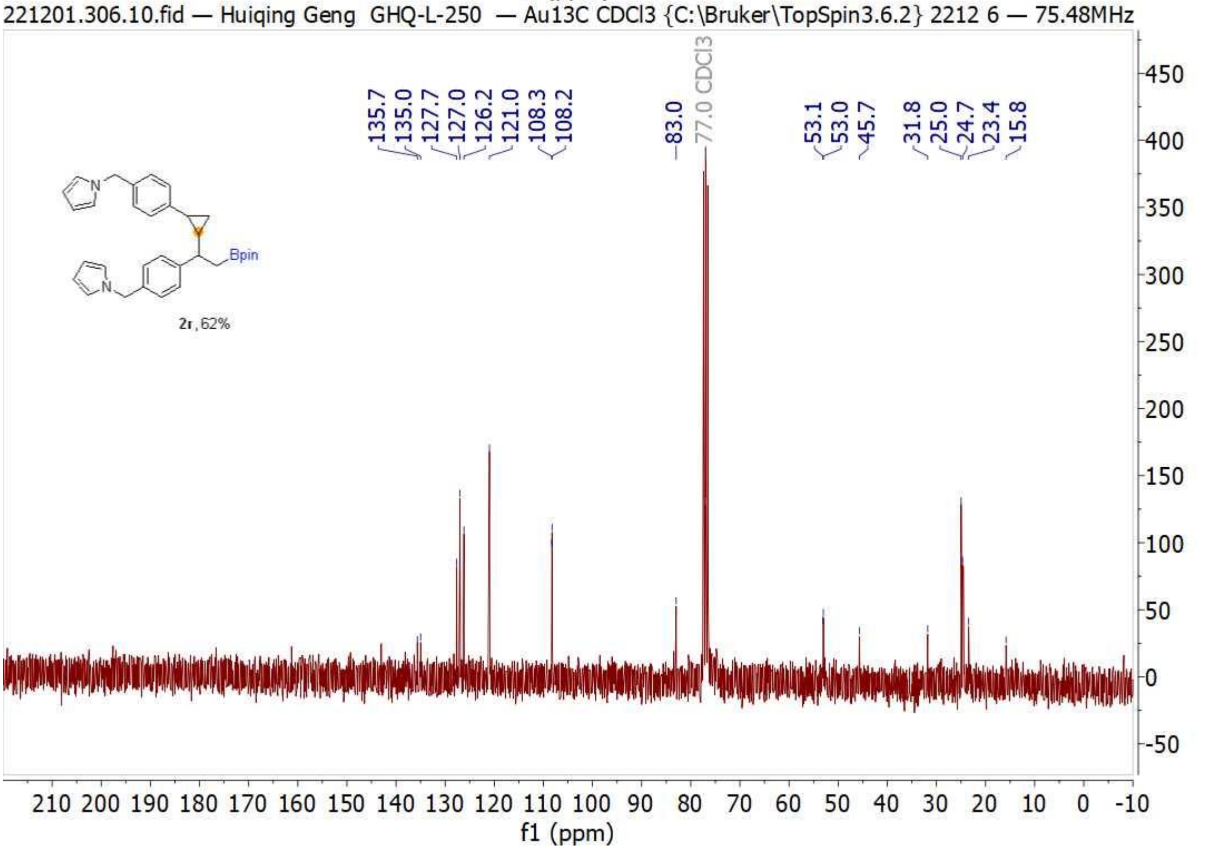
221201.306.10.fid — Huiqing Geng GHQ-L-250 — Au13C CDCl3 {C:\Bruker\TopSpin3.6.2} 2212 6 — 75.48MHz
135.7 135.0 127.7 127.0 126.2 121.0 108.3 108.2
83.0
77.0 CDCl3
53.1 53.0 45.7 31.8 25.0 24.7 23.4 15.8
Bpin
2r, 62%
f1 (ppm)

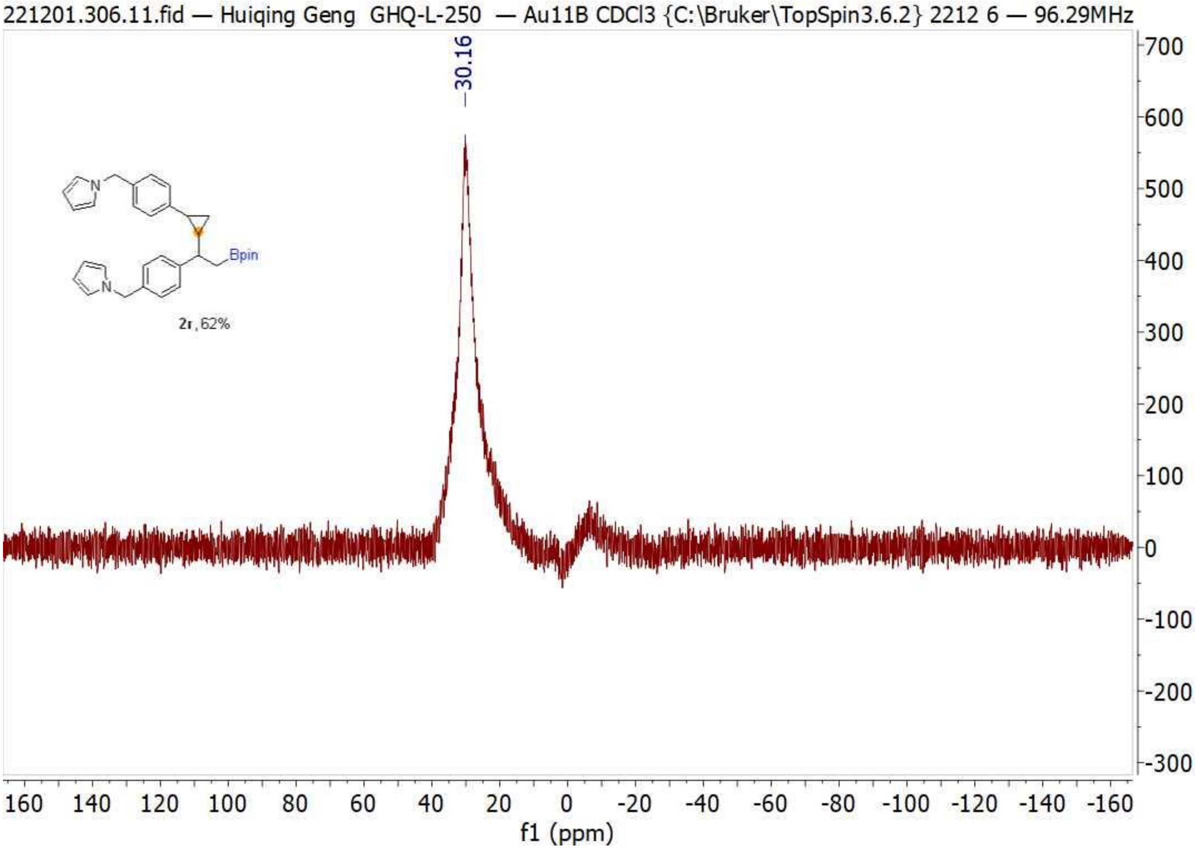
221201.306.11.fid — Huiqing Geng GHQ-L-250 — Au11B CDCl3 {C:\Bruker\TopSpin3.6.2} 2212 6 — 96.29MHz
30.16
Bpin
2r, 62%
700
600
500
400
300
200
100
0
-100
-200
-300
160 140 120 100 80 60 40 20 0 -20 -40 -60 -80 -100 -120 -140 -160
f1 (ppm)

220629.324.10.fid — Geng/ GHQ-L-152-2 — Au1H CDCl3 {C:\Bruker\TopSpin3.6.2} 2206 24 — 300.13MHz

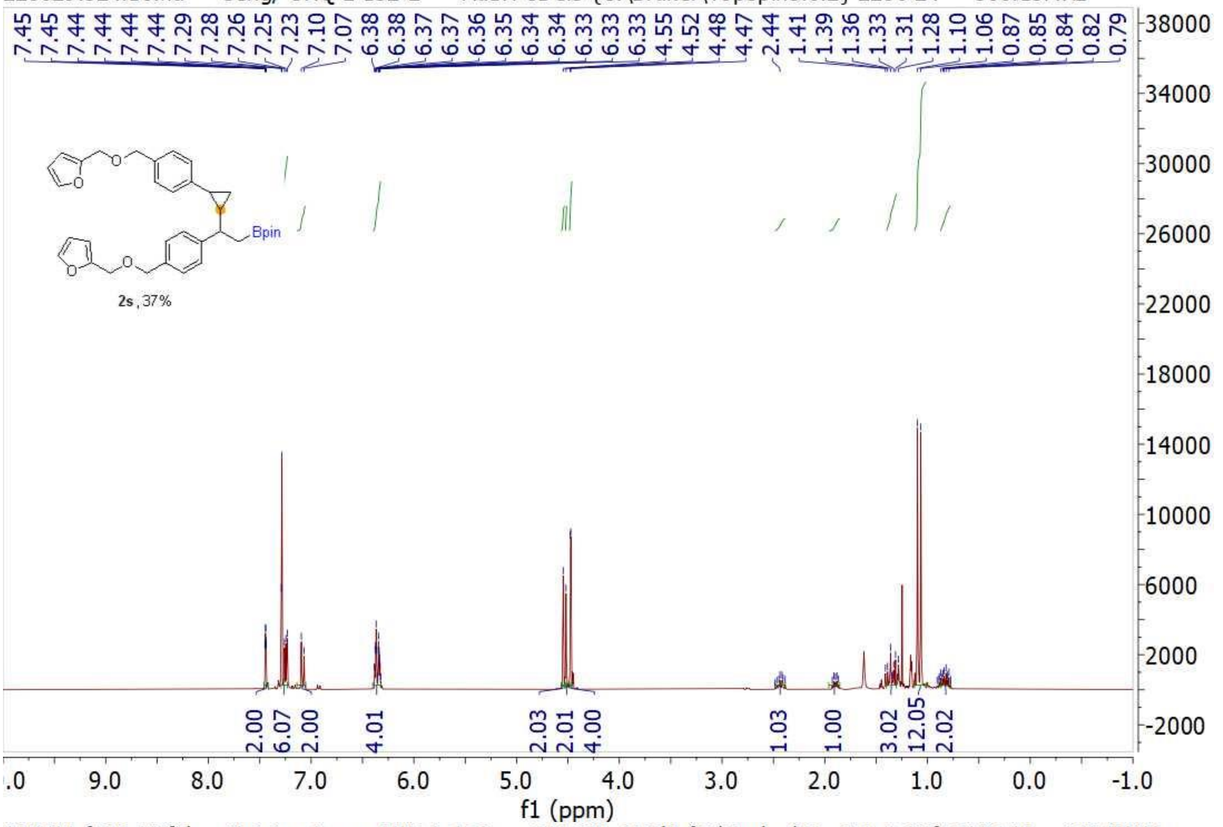

220711.f328.10.fid — Huiqing Geng GHQ-L-153 — C13CPD CDCl3 {C:\Bruker\TopSpin3.6.2} 2207 28 — 75.49MHz

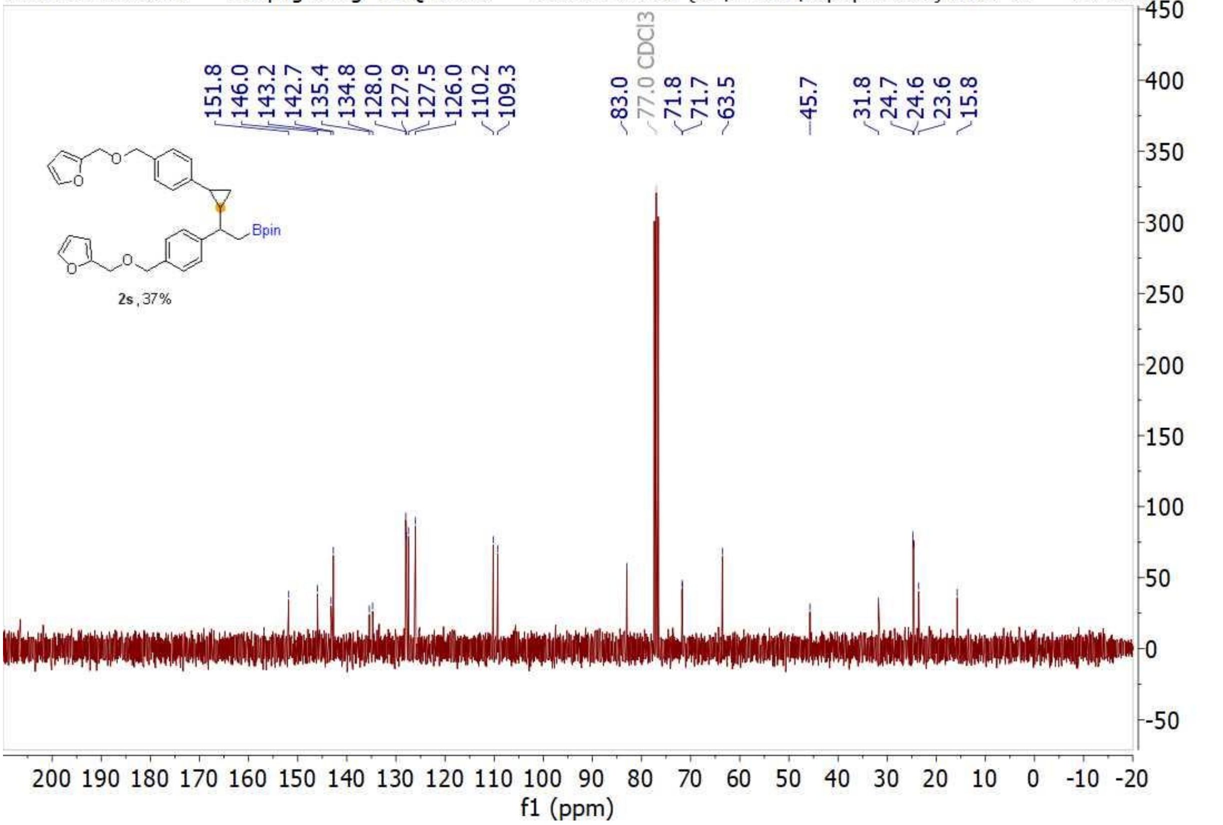

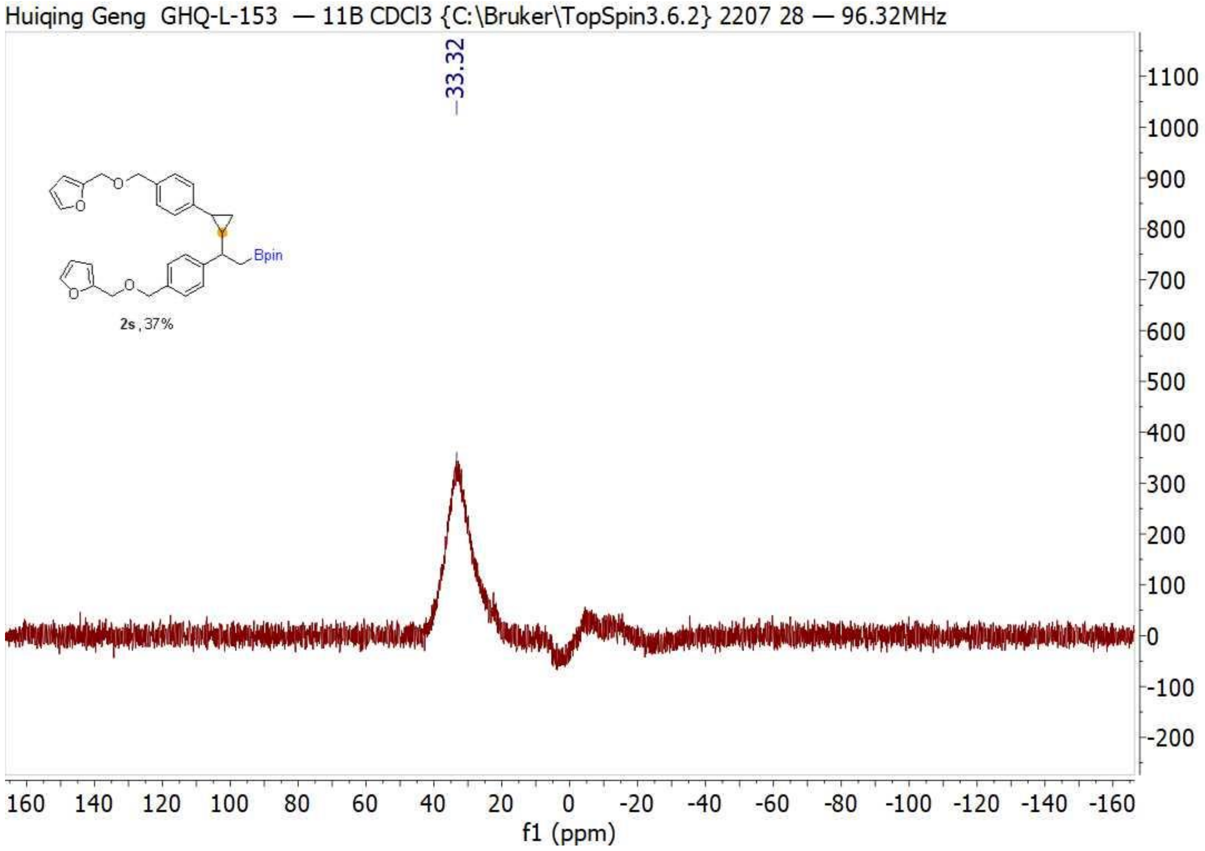
Huiqing Geng GHQ-L-153 — 11B CDCl3 {C:\Bruker\TopSpin3.6.2} 2207 28 — 96.32MHz
—33.32
Bpin
2s, 37%
f1 (ppm)

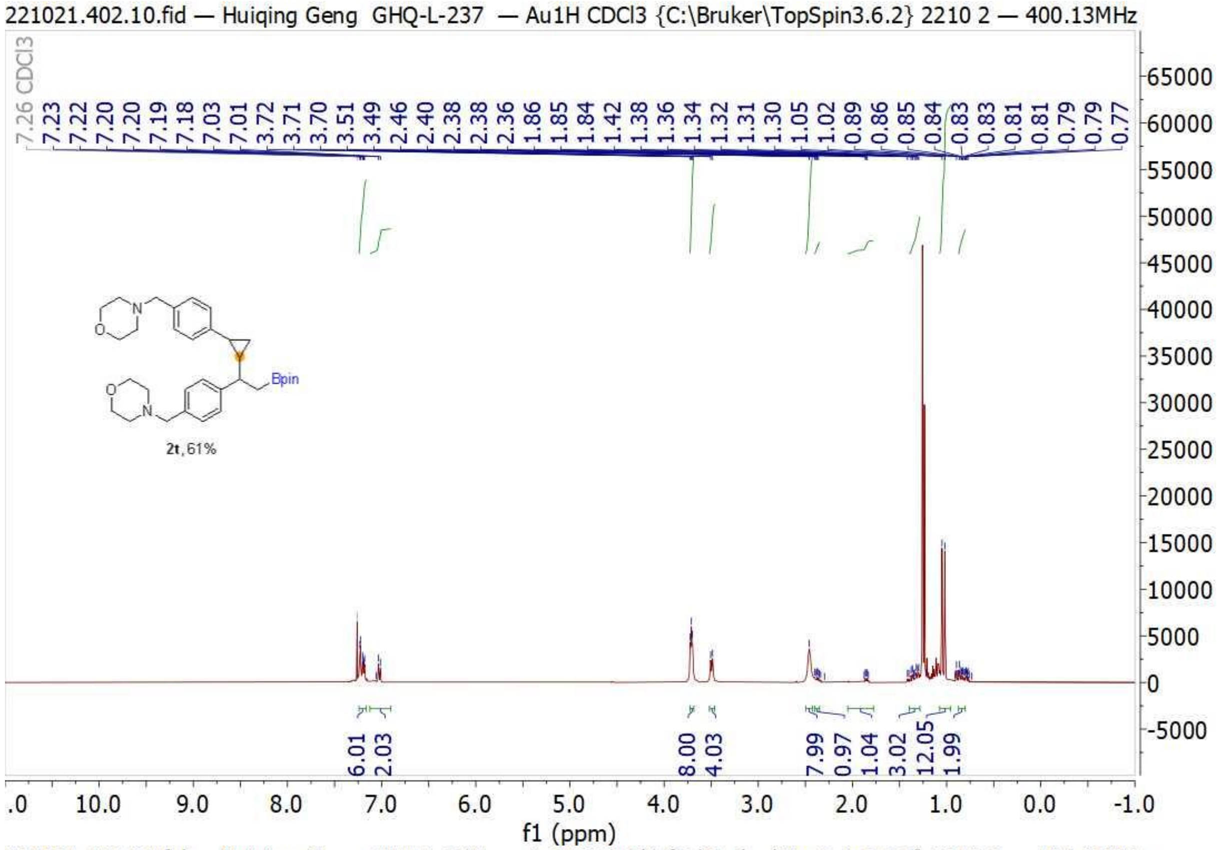
221021.402.10.fid — Huiqing Geng GHQ-L-237 — Au1H CDCl3 {C:\Bruker\TopSpin3.6.2} 2210 2 — 400.13MHz
7.26 CDCl3
7.23 7.22 7.20 7.20 7.19 7.18 7.03 7.01 3.72 3.71 3.70 3.51 3.49 2.46 2.40 2.38 2.38 2.36 1.86 1.85 1.84 1.42 1.38 1.36 1.34 1.32 1.31 1.30 1.05 1.02 0.89 0.86 0.85 0.84 0.83 0.83 0.81 0.81 0.79 0.79 0.77
Bpin
2t, 61%
6.01 2.03 8.00 4.03 7.99 0.97 1.04 3.02 12.05 1.99
f1 (ppm)

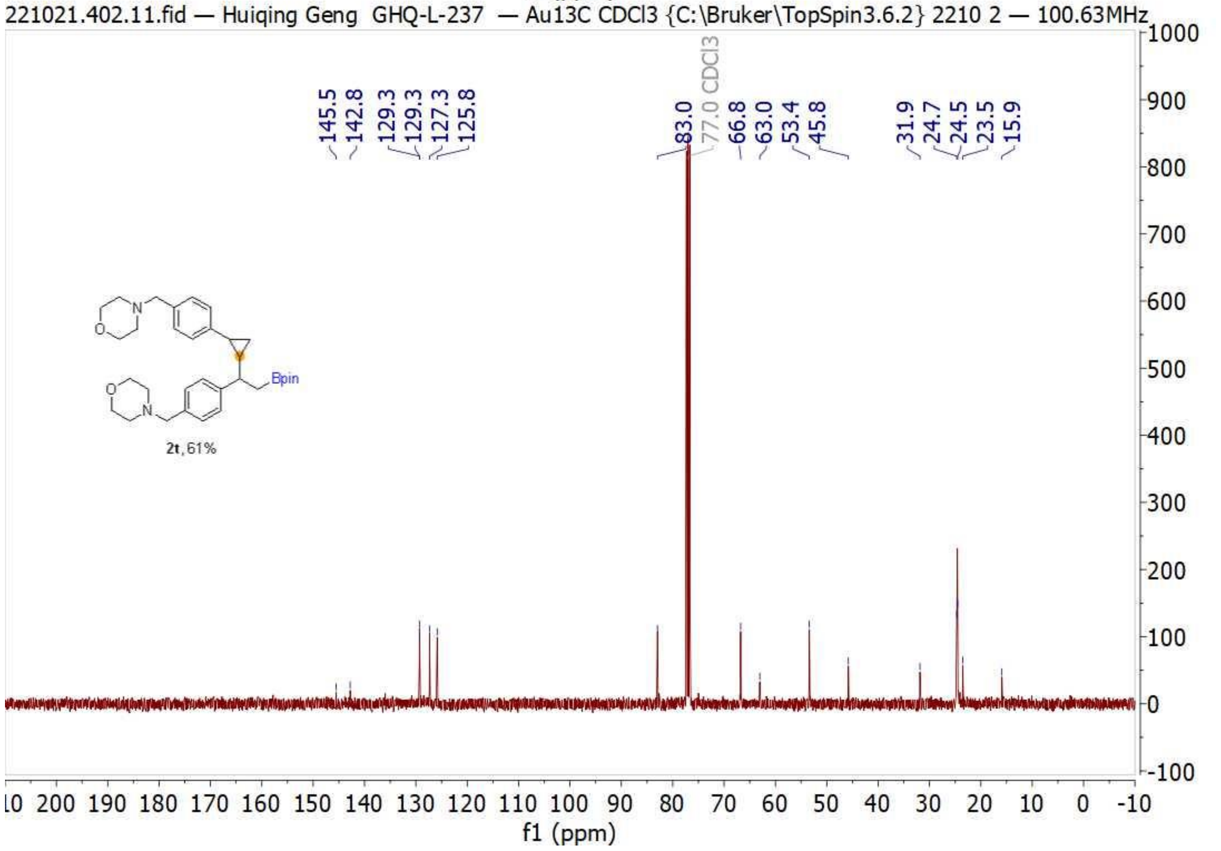
221021.402.11.fid — Huiqing Geng GHQ-L-237 — Au13C CDCl3 {C:\Bruker\TopSpin3.6.2} 2210 2 — 100.63MHz
77.0 CDCl3
145.5 142.8 129.3 129.3 127.3 125.8 83.0 66.8 63.0 53.4 45.8 31.9 24.7 24.5 23.5 15.9
Bpin
2t, 61%
f1 (ppm)

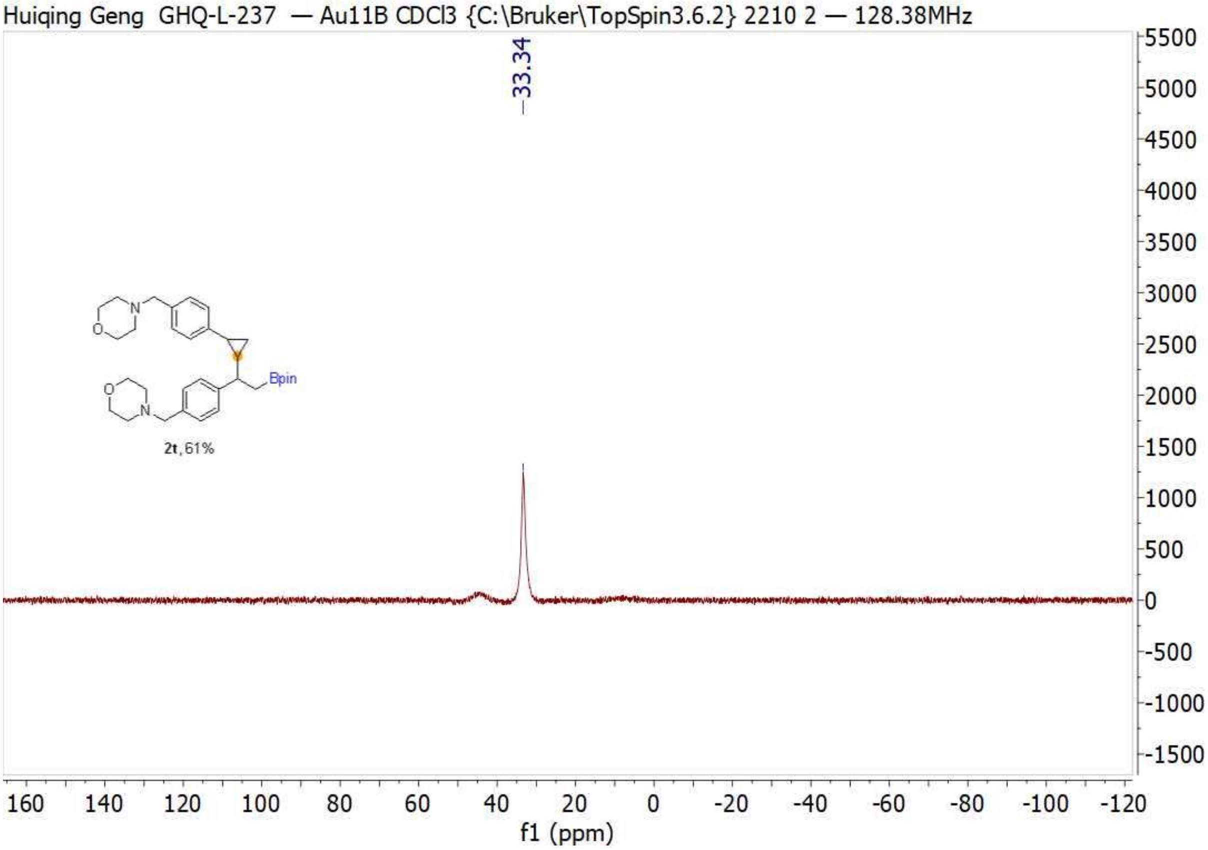
Huiqing Geng GHQ-L-237 — Au11B CDCl3 {C:\Bruker\TopSpin3.6.2} 2210 2 — 128.38MHz
33.34
Bpin
2t, 61%
f1 (ppm)

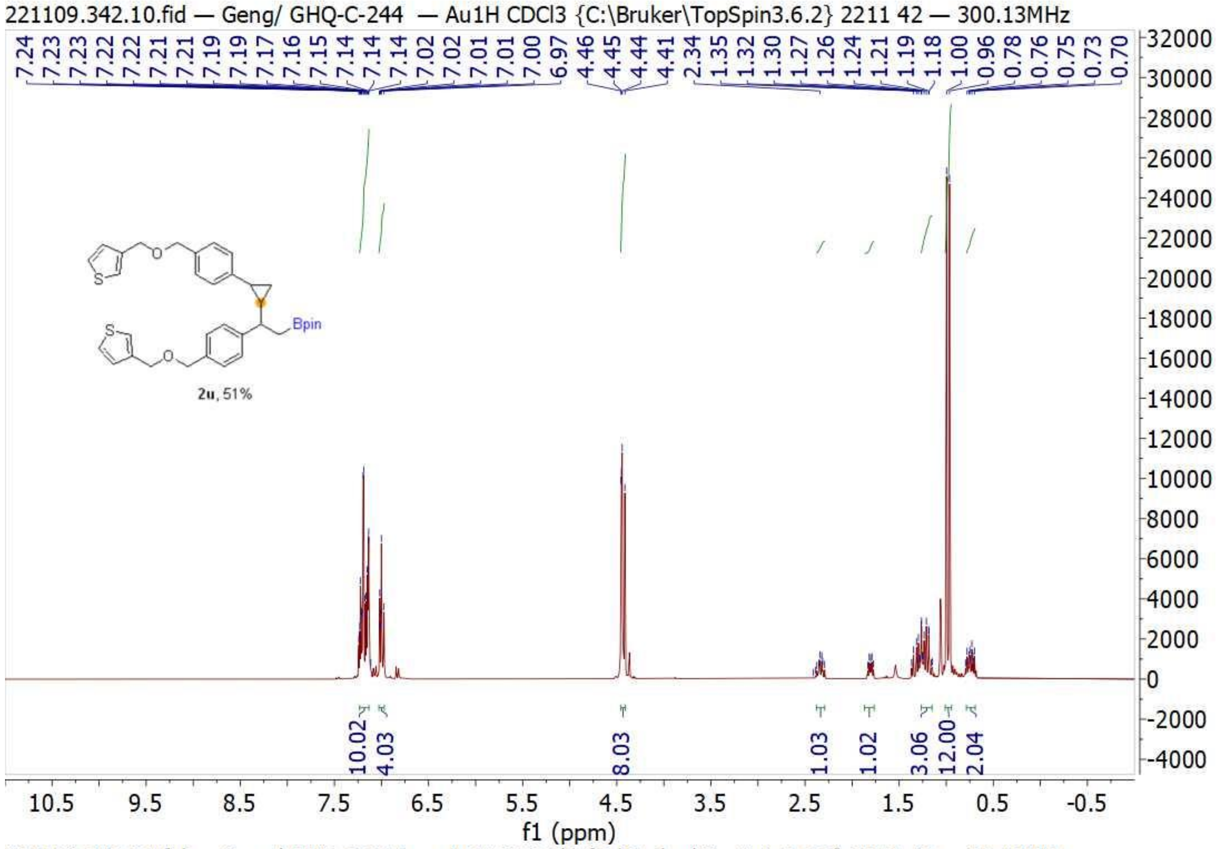
221109.342.10.fid — Geng/ GHQ-C-244 — Au1H CDCl3 {C:\Bruker\TopSpin3.6.2} 2211 42 — 300.13MHz
7.24 7.23 7.23 7.22 7.22 7.21 7.21 7.19 7.19 7.17 7.16 7.15 7.14 7.14 7.14 7.02 7.02 7.01 7.01 7.00 6.97 4.46 4.45 4.44 4.41 2.34 1.35 1.32 1.30 1.27 1.26 1.24 1.21 1.19 1.18 1.00 0.96 0.78 0.76 0.75 0.73 0.70
Bpin
2u, 51%
10.02 4.03 8.03 1.03 1.02 3.06 12.00 2.04
f1 (ppm)

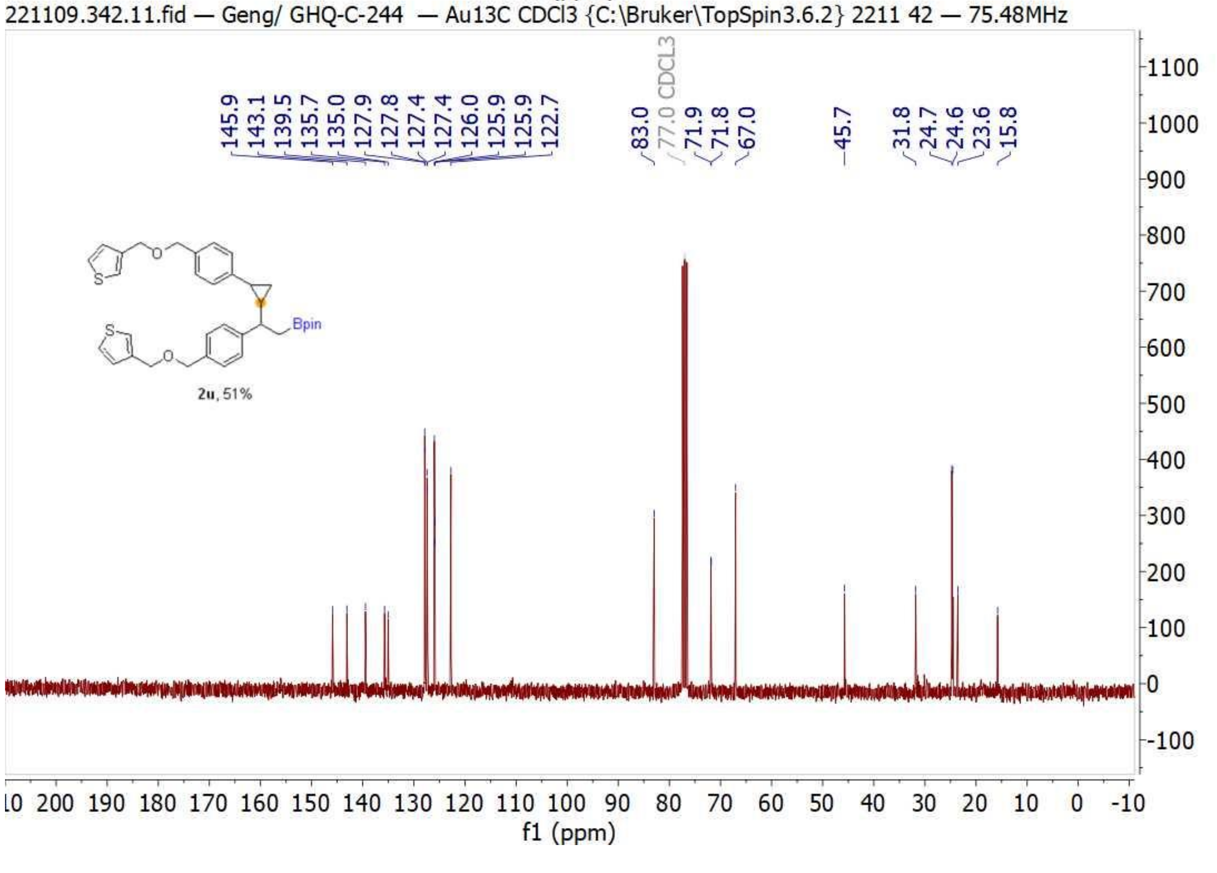
221109.342.11.fid — Geng/ GHQ-C-244 — Au13C CDCl3 {C:\Bruker\TopSpin3.6.2} 2211 42 — 75.48MHz
145.9 143.1 139.5 135.7 135.0 127.9 127.8 127.4 127.4 126.0 125.9 125.9 122.7 83.0 77.0 CDCL3 71.9 71.8 67.0 45.7 31.8 24.7 24.6 23.6 15.8
Bpin
2u, 51%
f1 (ppm)

Geng/ GHQ-C-244 — Au11B CDCl3 {C:\Bruker\TopSpin3.6.2} 2211 42 — 96.29MHz

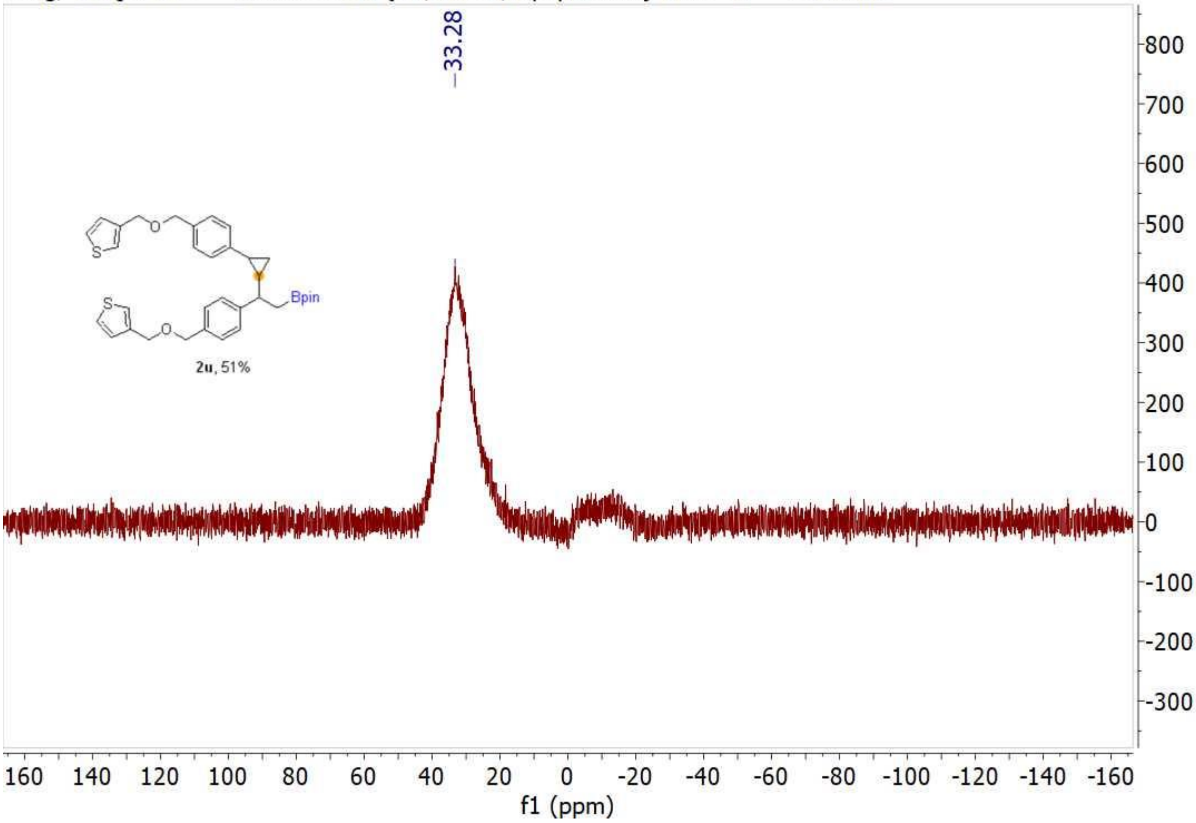

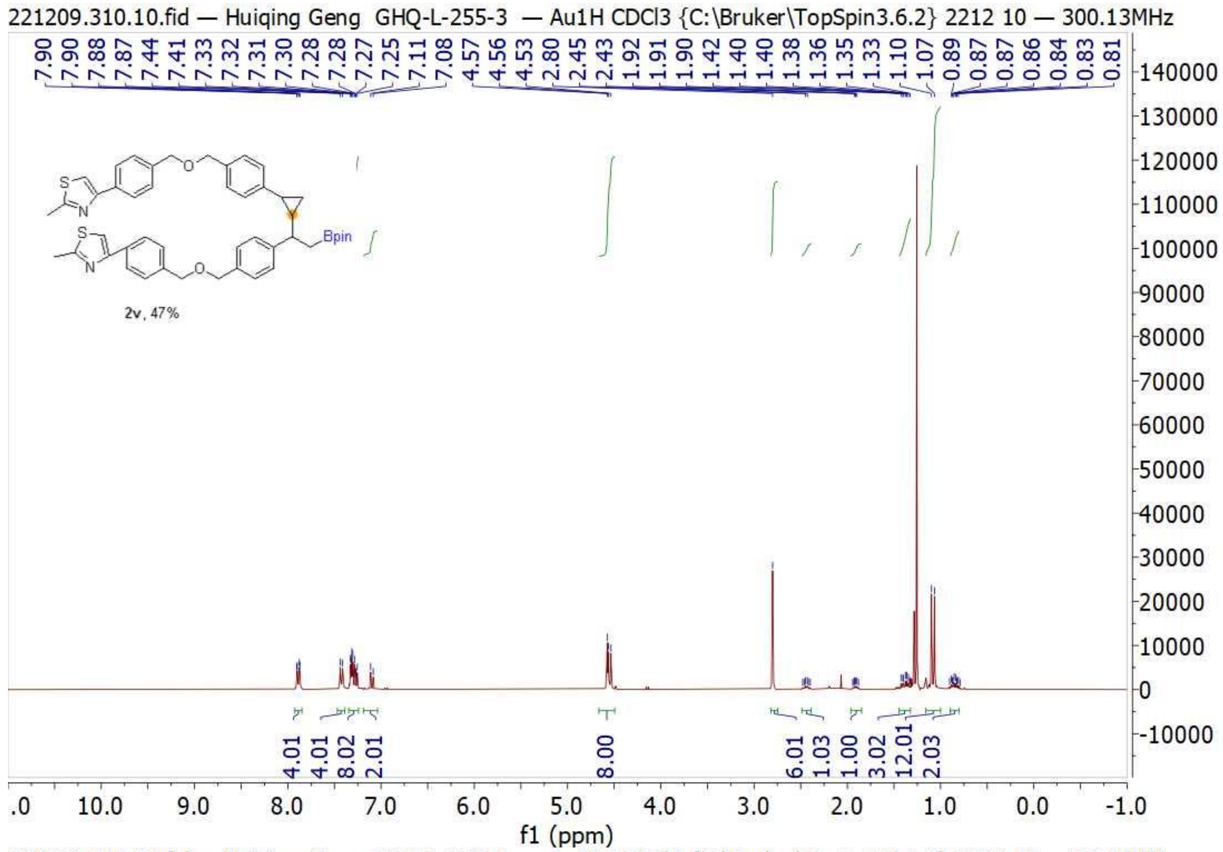
221209.310.10.fid — Huiqing Geng GHQ-L-255-3 — Au1H CDCl3 {C:\Bruker\TopSpin3.6.2} 2212 10 — 300.13MHz
7.90 7.90 7.88 7.87 7.44 7.41 7.33 7.32 7.31 7.30 7.28 7.28 7.27 7.25 7.11 7.08 4.57 4.56 4.53 2.80 2.45 2.43 1.92 1.91 1.90 1.42 1.40 1.40 1.38 1.36 1.35 1.33 1.10 1.07 0.89 0.87 0.87 0.86 0.84 0.83 0.81
Bpin
2v, 47%
4.01 4.01 8.02 2.01 8.00 6.01 1.03 1.00 3.02 12.01 2.03
f1 (ppm)

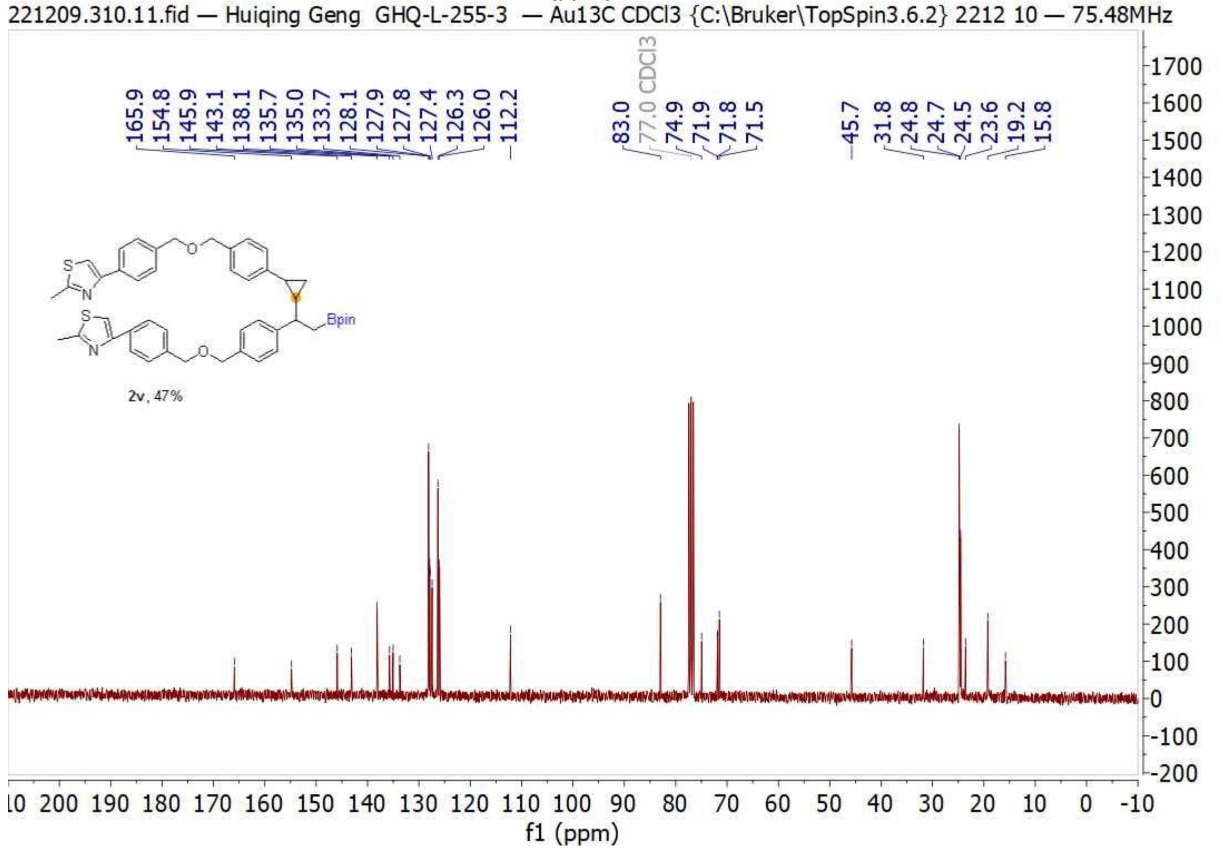
221209.310.11.fid — Huiqing Geng GHQ-L-255-3 — Au13C CDCl3 {C:\Bruker\TopSpin3.6.2} 2212 10 — 75.48MHz
165.9 154.8 145.9 143.1 138.1 135.7 135.0 133.7 128.1 127.9 127.8 127.4 126.3 126.0 112.2
83.0 77.0 CDCl3 74.9 71.9 71.8 71.5
45.7 31.8 24.8 24.7 24.5 23.6 19.2 15.8
Bpin
2v, 47%
f1 (ppm)

Huiqing Geng GHQ-L-255-3 — Au11B CDCl3 {C:\Bruker\TopSpin3.6.2} 2212 10 — 96.29MHz

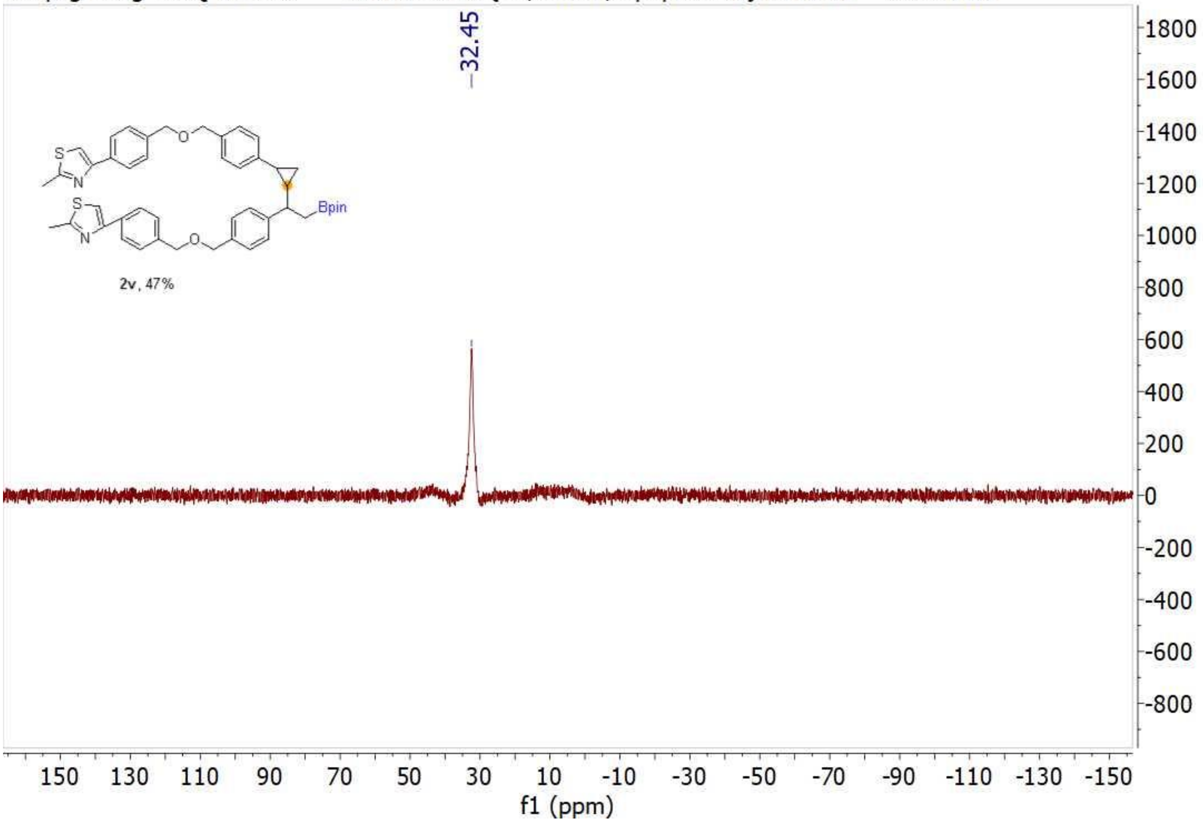

221209.312.10.fid — Huiqing Geng GHQ-L-256-3 — Au1H CDCl3 {C:\Bruker\TopSpin3.6.2} 2212 12 — 300.13MHz

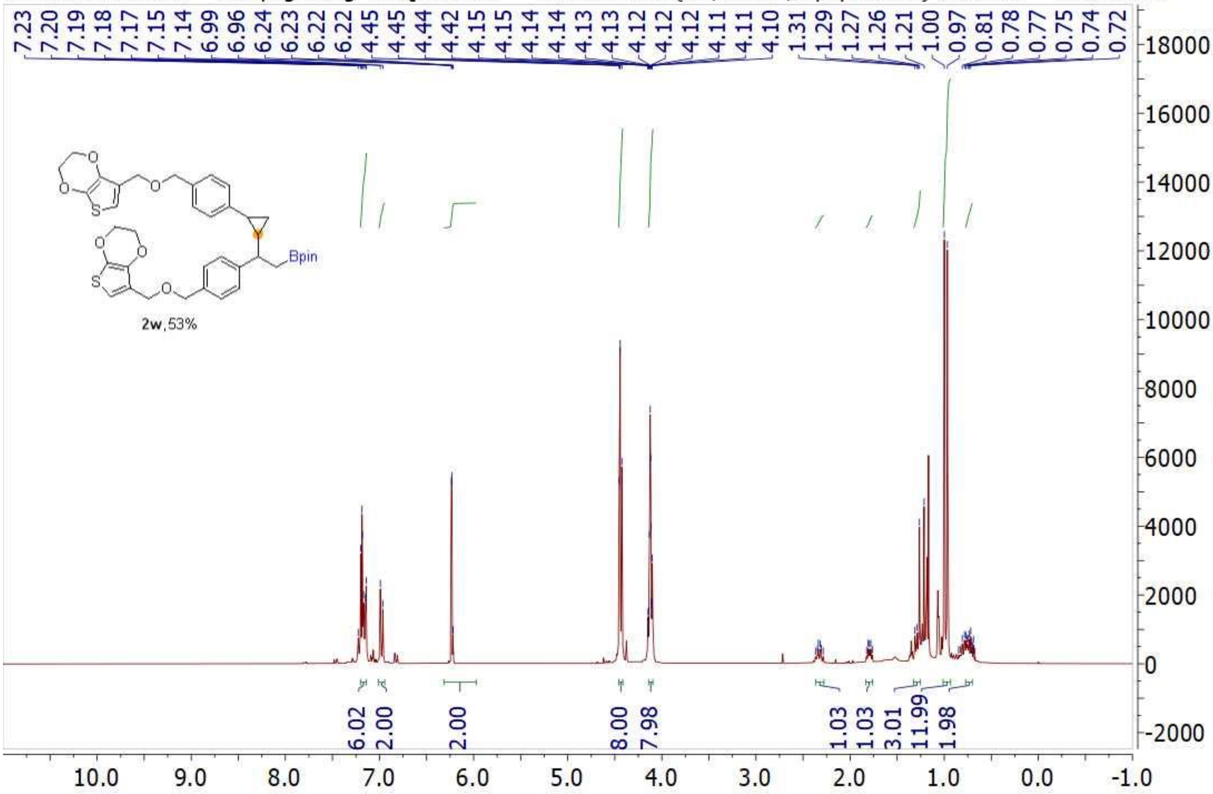

221209.311.10.fid — Huiqing Geng GHQ-L-256 — Au13C CDCl3 {C:\Bruker\TopSpin3.6.2} 2212 11 — 75.48MHz

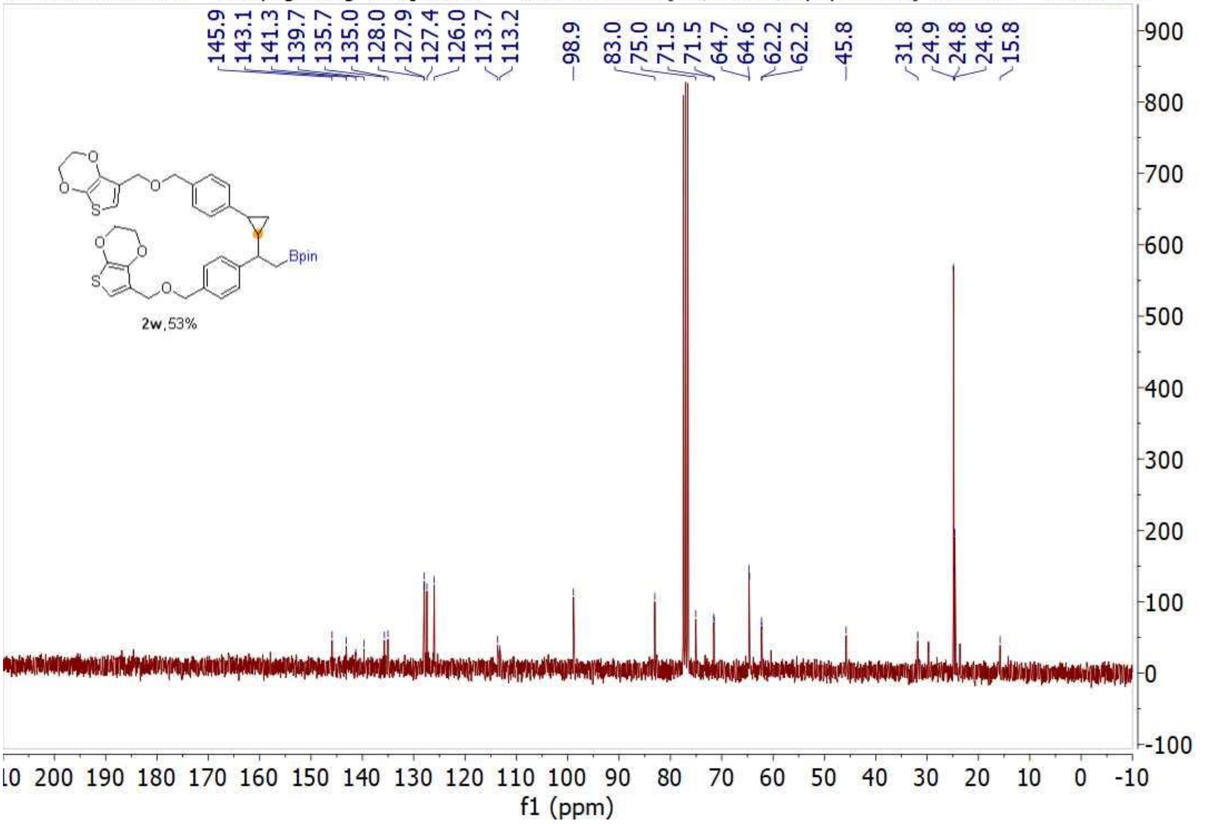

221209.311.11.fid — Huiqing Geng GHQ-L-256 — Au11B CDCl3 {C:\Bruker\TopSpin3.6.2} 2212 11 — 96.29MHz

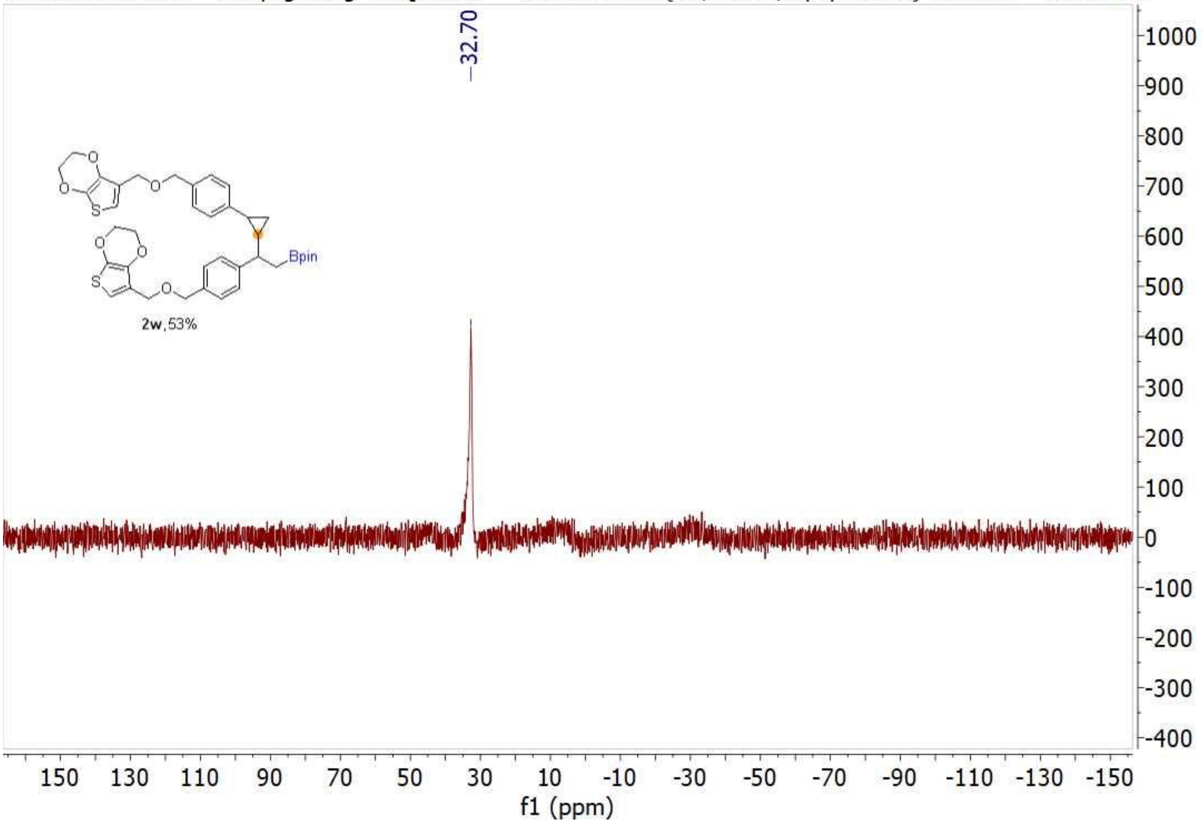

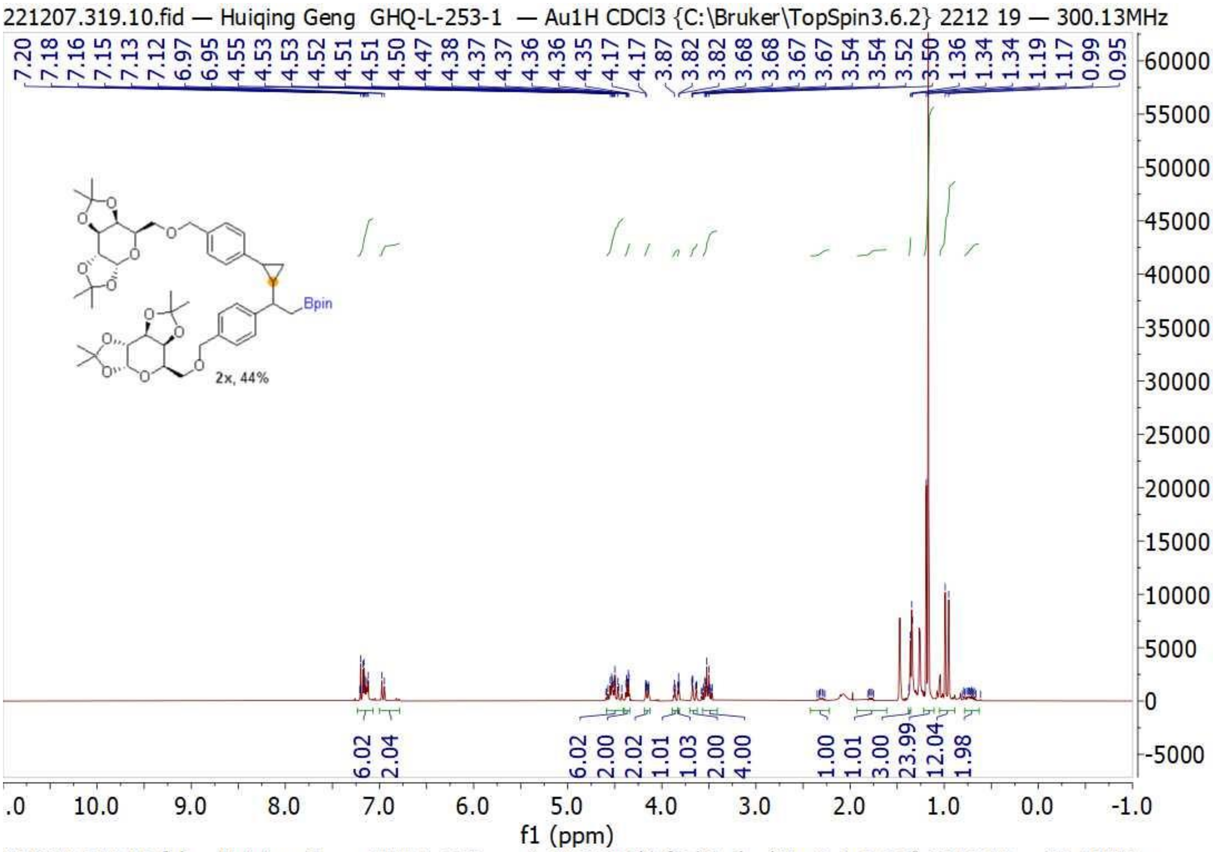
221207.319.10.fid — Huiqing Geng GHQ-L-253-1 — Au1H CDCl3 {C:\Bruker\TopSpin3.6.2} 2212 19 — 300.13MHz
Bpin
2x, 44%
f1 (ppm)

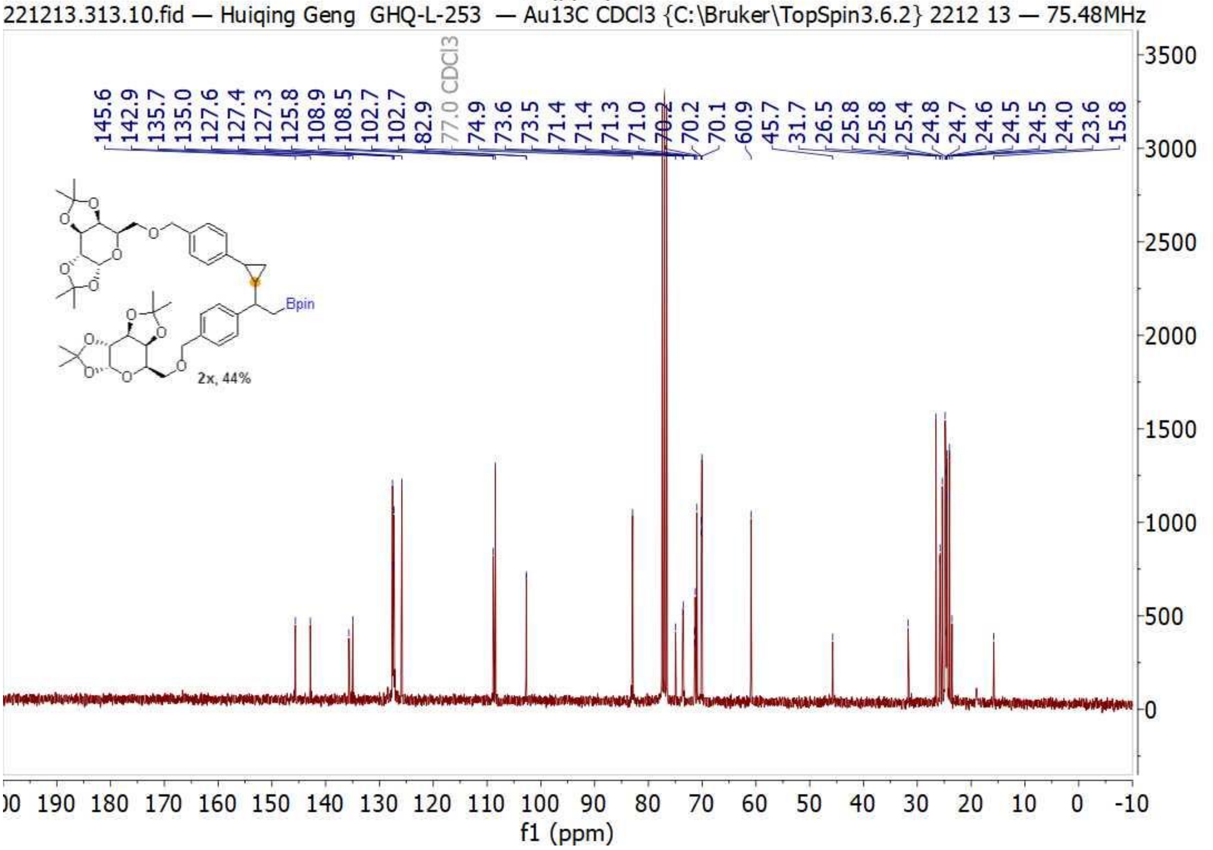
221213.313.10.fid — Huiqing Geng GHQ-L-253 — Au13C CDCl3 {C:\Bruker\TopSpin3.6.2} 2212 13 — 75.48MHz
CDCl3
Bpin
2x, 44%
f1 (ppm)

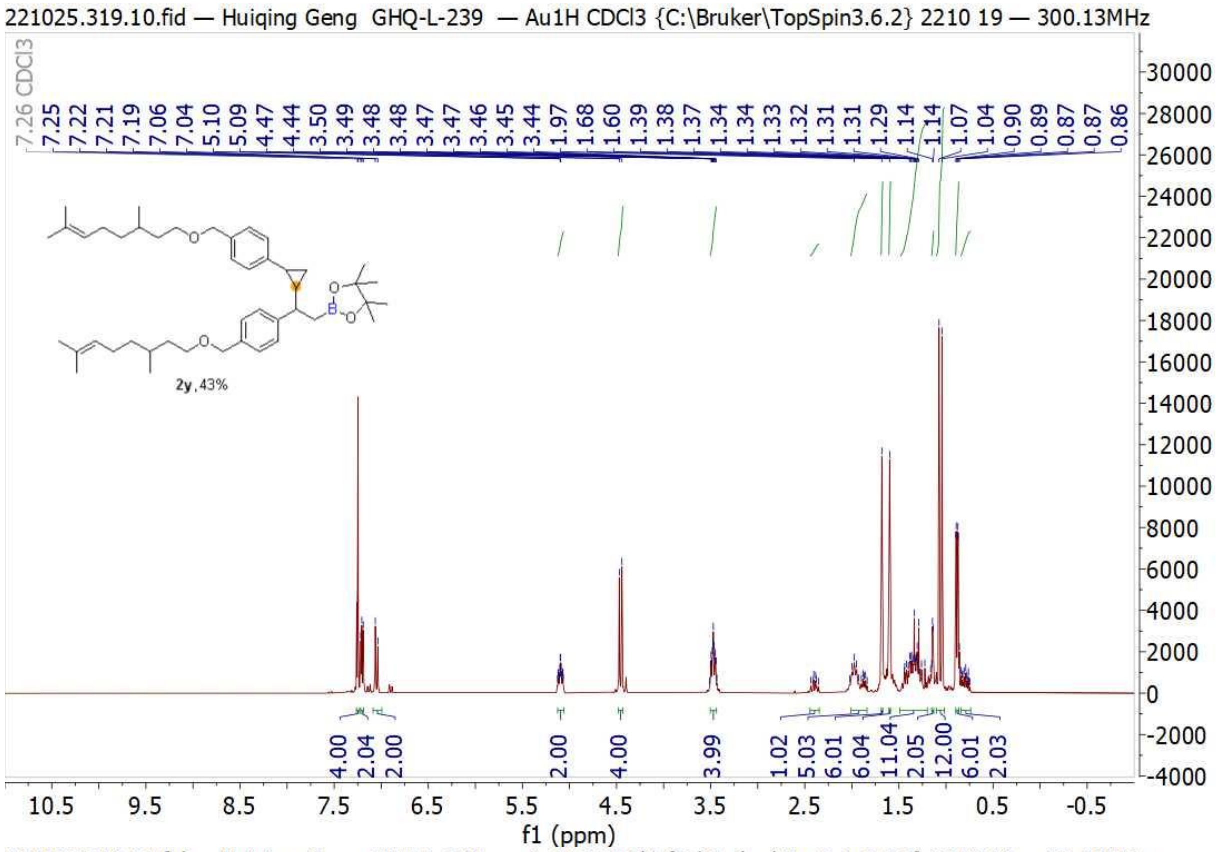
221025.319.10.fid — Huiqing Geng GHQ-L-239 — Au1H CDCl3 {C:\Bruker\TopSpin3.6.2} 2210 19 — 300.13MHz
7.26 CDCl3
7.25 7.22 7.21 7.19 7.06 7.04 5.10 5.09 4.47 4.44 3.50 3.49 3.48 3.48 3.47 3.47 3.46 3.45 3.44 1.97 1.68 1.60 1.39 1.38 1.37 1.34 1.34 1.33 1.32 1.31 1.31 1.29 1.14 1.14 1.07 1.04 0.90 0.89 0.87 0.87 0.86
2y, 43%
4.00 2.04 2.00 2.00 4.00 3.99 1.02 5.03 6.01 6.04 11.04 2.05 12.00 6.01 2.03
f1 (ppm)

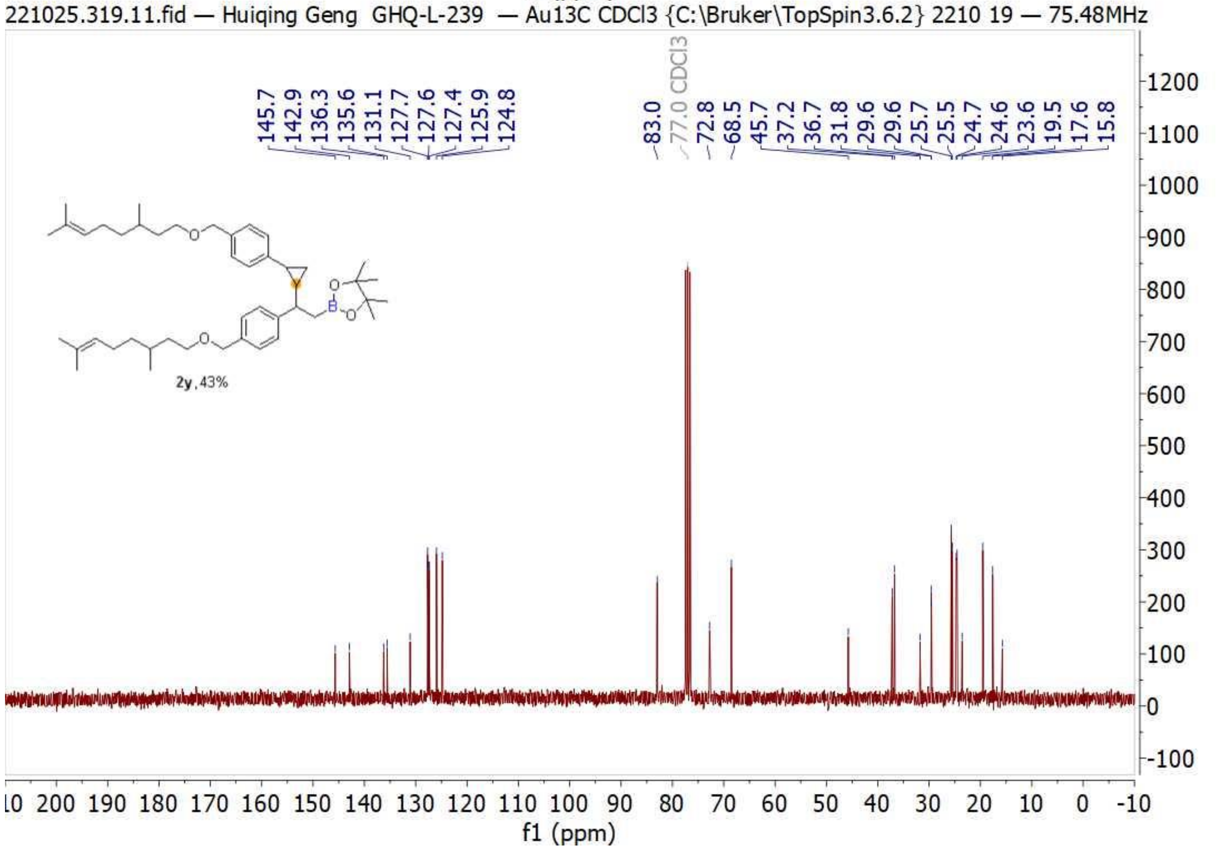
221025.319.11.fid — Huiqing Geng GHQ-L-239 — Au13C CDCl3 {C:\Bruker\TopSpin3.6.2} 2210 19 — 75.48MHz
145.7 142.9 136.3 135.6 131.1 127.7 127.6 127.4 125.9 124.8
83.0 77.0 CDCl3 72.8 68.5 45.7 37.2 36.7 31.8 29.6 29.6 25.7 25.5 24.7 24.6 23.6 19.5 17.6 15.8
2y, 43%
f1 (ppm)

Huiqing Geng GHQ-L-239 — Au11B CDCl3 {C:\Bruker\TopSpin3.6.2} 2210 19 — 96.29MHz

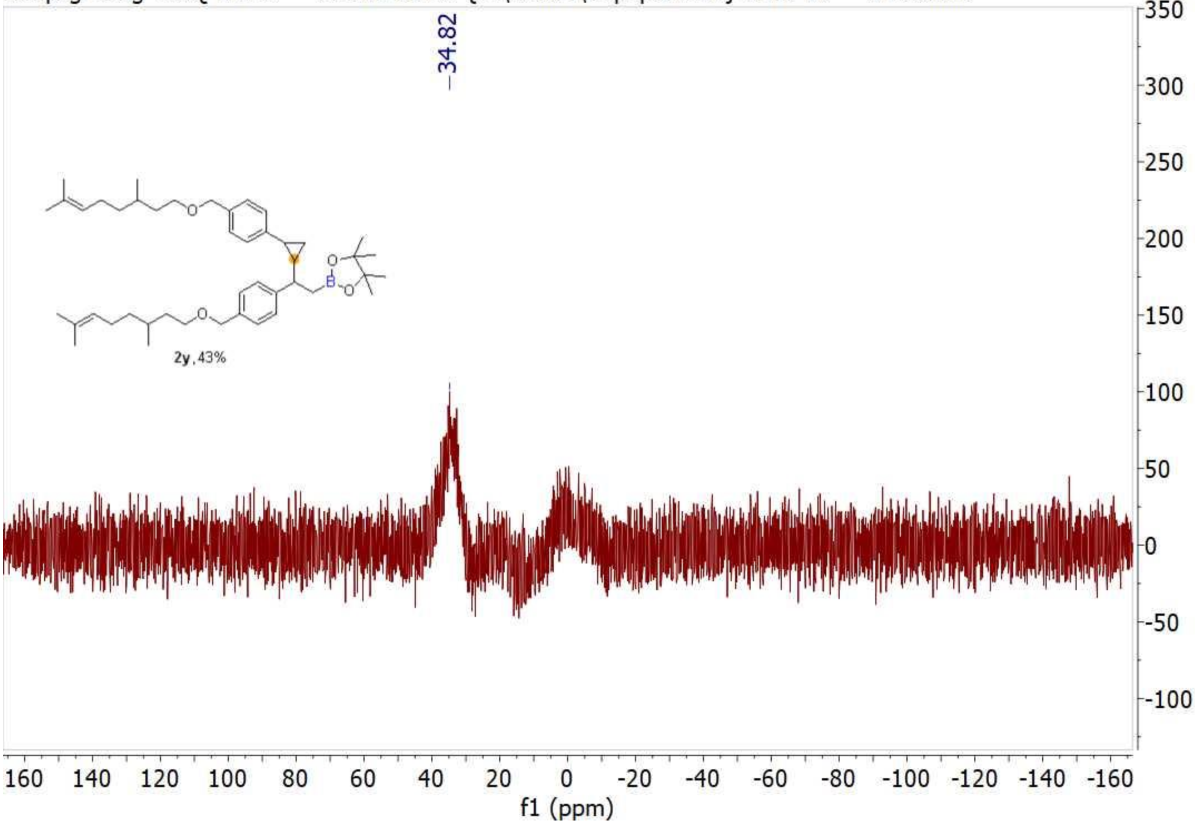

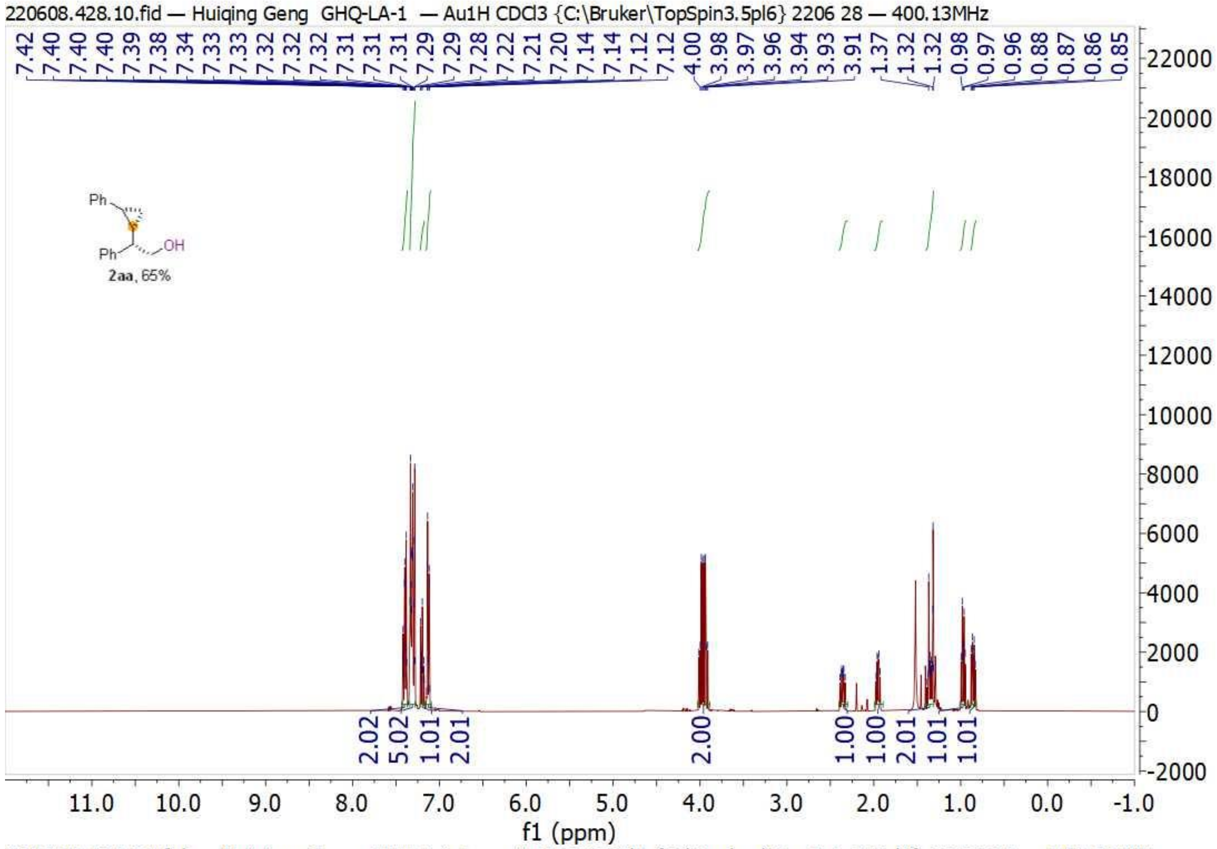
220608.428.10.fid — Huiqing Geng GHQ-LA-1 — Au1H CDCl3 {C:\Bruker\TopSpin3.5pl6} 2206 28 — 400.13MHz
7.42 7.40 7.40 7.40 7.39 7.38 7.34 7.33 7.33 7.32 7.32 7.32 7.31 7.31 7.31 7.29 7.29 7.28 7.22 7.21 7.20 7.14 7.14 7.12 7.12 4.00 3.98 3.97 3.96 3.94 3.93 3.91 1.37 1.32 1.32 0.98 0.97 0.96 0.88 0.87 0.86 0.85
Ph
Ph
OH
2aa, 65%
2.02 5.02 1.01 2.01 2.00 1.00 1.00 2.01 1.01 1.01
f1 (ppm)

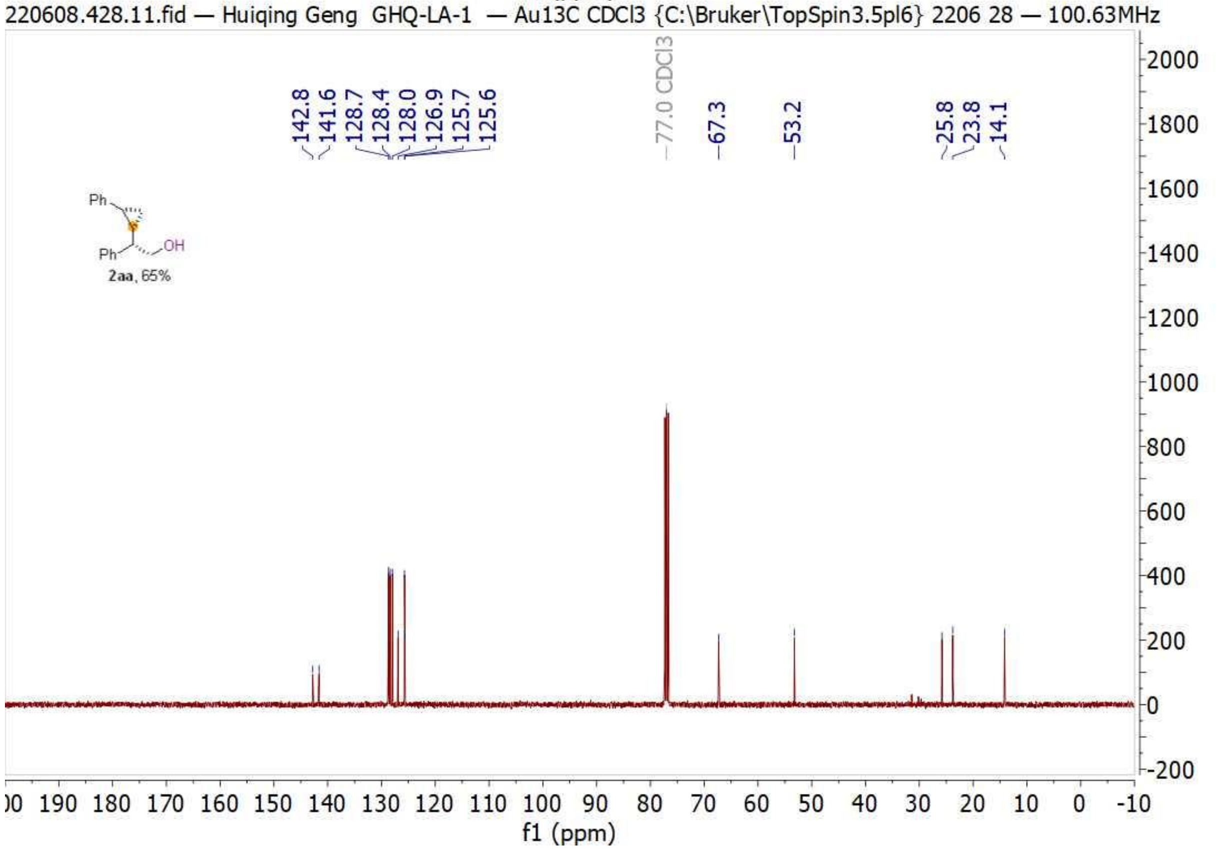
220608.428.11.fid — Huiqing Geng GHQ-LA-1 — Au13C CDCl3 {C:\Bruker\TopSpin3.5pl6} 2206 28 — 100.63MHz
142.8 141.6 128.7 128.4 128.0 126.9 125.7 125.6
77.0 CDCl3
67.3
53.2
25.8 23.8 14.1
Ph
Ph
OH
2aa, 65%
f1 (ppm)

221110.341.10.fid — Geng/ GHQ-LB-17 — Au1H CDCl3 {C:\Bruker\TopSpin3.6.2} 2211 41 — 300.13MHz

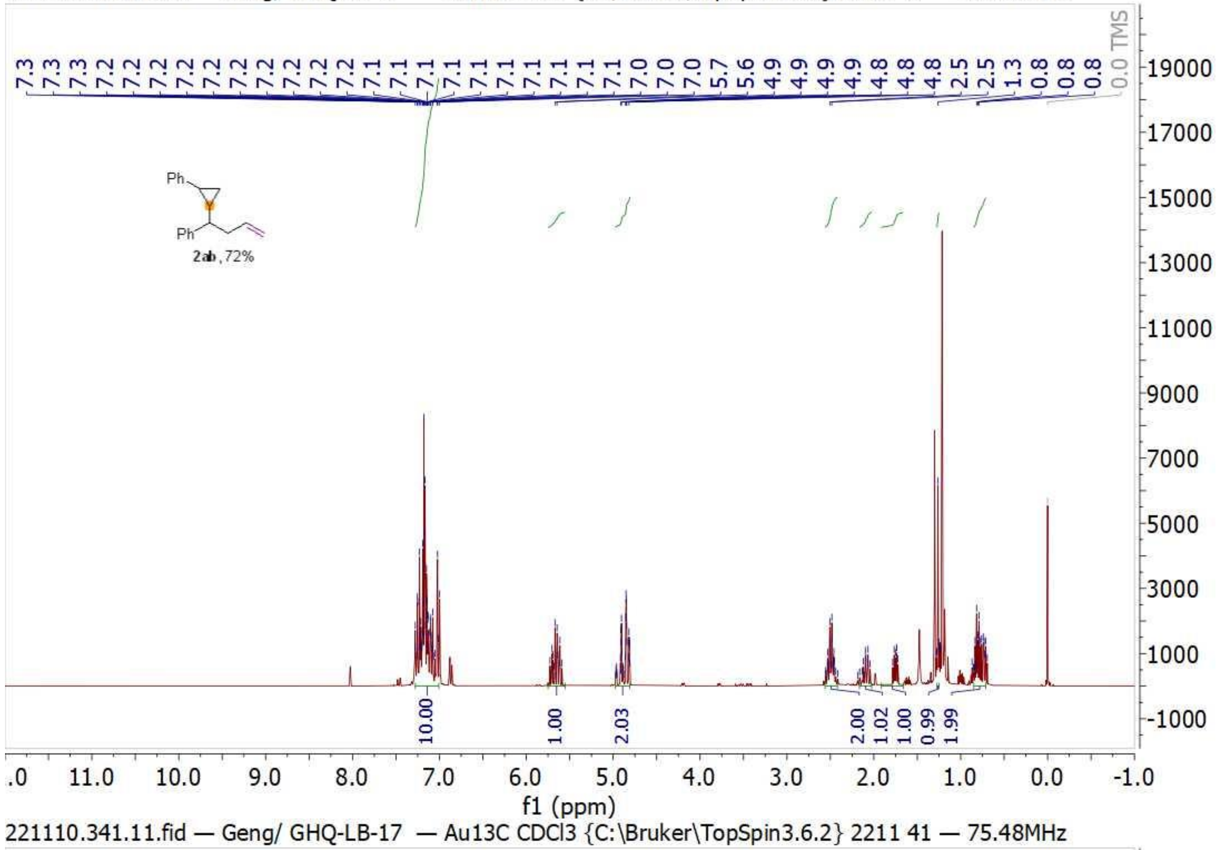

221110.341.11.fid — Geng/ GHQ-LB-17 — Au13C CDCl3 {C:\Bruker\TopSpin3.6.2} 2211 41 — 75.48MHz

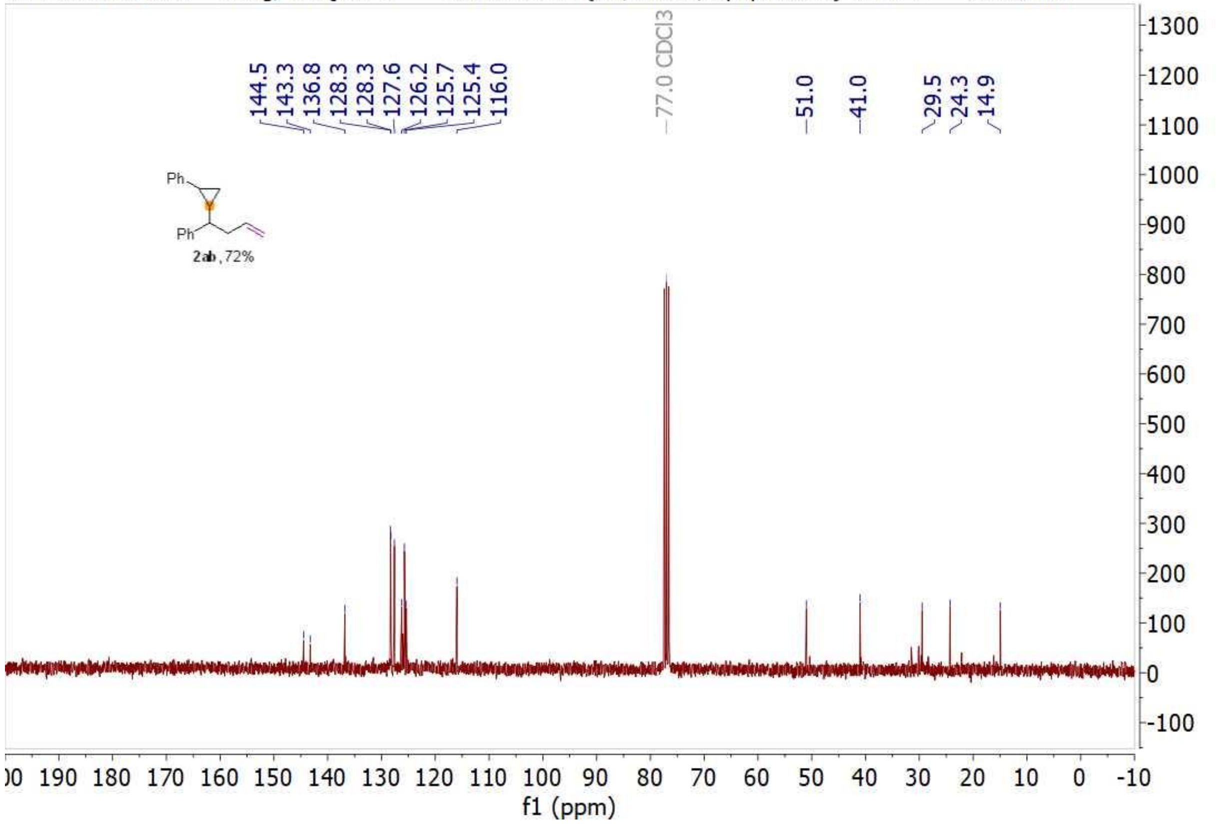

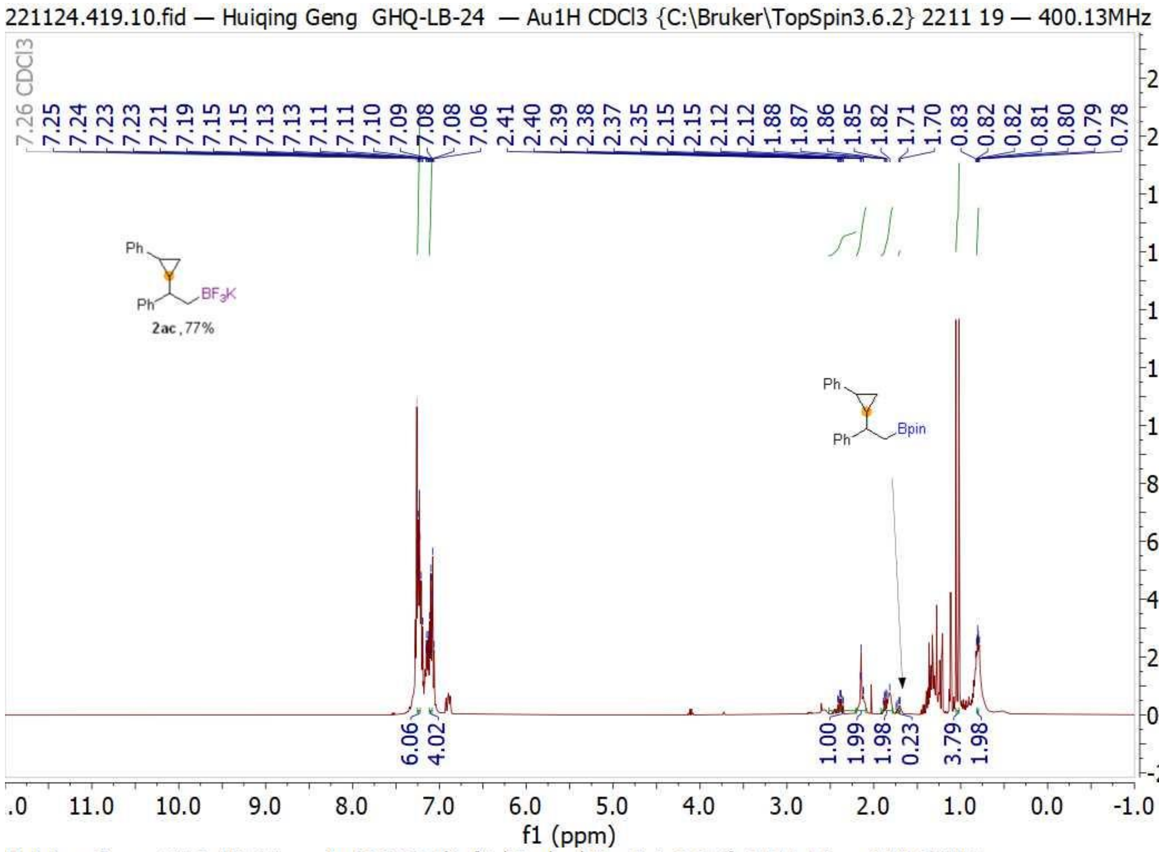
221124.419.10.fid — Huiqing Geng GHQ-LB-24 — Au1H CDCl3 {C:\Bruker\TopSpin3.6.2} 2211 19 — 400.13MHz
7.26 CDCl3
7.25 7.24 7.23 7.23 7.21 7.19 7.15 7.15 7.13 7.13 7.11 7.11 7.10 7.09 7.08 7.08 7.06 2.41 2.40 2.39 2.38 2.37 2.35 2.15 2.15 2.12 2.12 1.88 1.87 1.86 1.85 1.82 1.71 1.70 0.83 0.82 0.82 0.81 0.80 0.79 0.78
Ph
Ph
BF3K
2ac, 77%
Ph
Ph
Bpin
6.06 4.02 1.00 1.99 1.98 0.23 3.79 1.98
f1 (ppm)

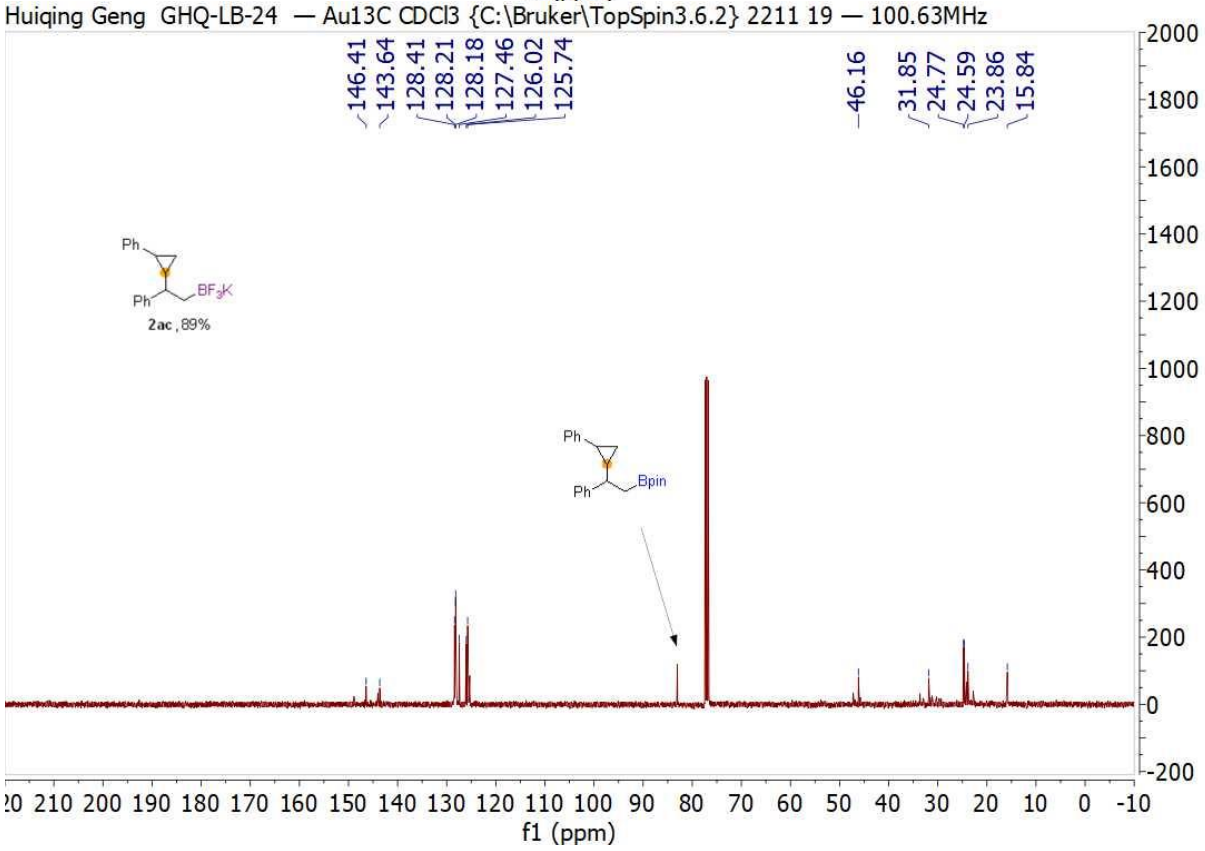
Huiqing Geng GHQ-LB-24 — Au13C CDCl3 {C:\Bruker\TopSpin3.6.2} 2211 19 — 100.63MHz
146.41 143.64 128.41 128.21 128.18 127.46 126.02 125.74 46.16 31.85 24.77 24.59 23.86 15.84
Ph
Ph
BF3K
2ac, 89%
Ph
Ph
Bpin
f1 (ppm)

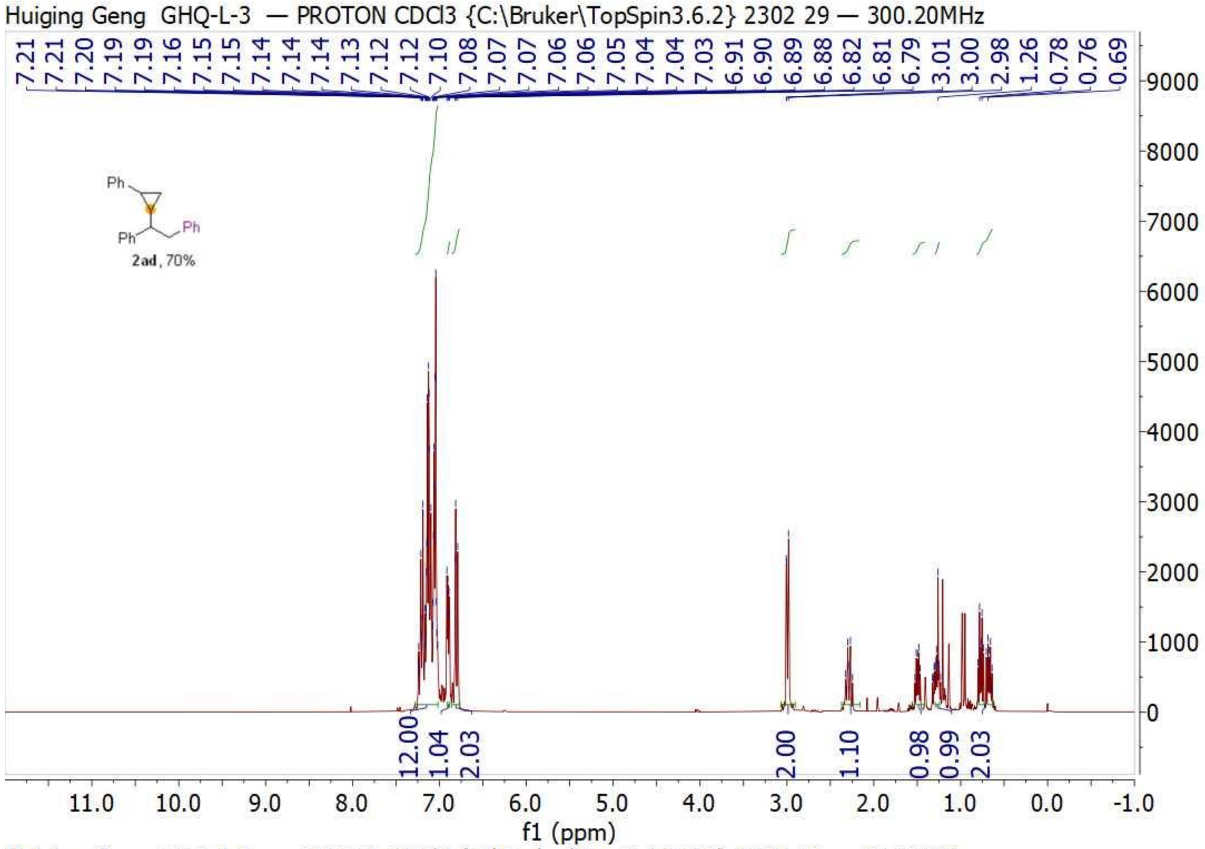
Huiging Geng GHQ-L-3 — PROTON CDCl3 {C:\Bruker\TopSpin3.6.2} 2302 29 — 300.20MHz
7.21 7.21 7.20 7.19 7.19 7.16 7.15 7.15 7.14 7.14 7.14 7.13 7.12 7.12 7.10 7.08 7.07 7.07 7.06 7.06 7.05 7.04 7.04 7.03 6.91 6.90 6.89 6.88 6.82 6.81 6.79 3.01 3.00 2.98 1.26 0.78 0.76 0.69
Ph
Ph
Ph
2ad, 70%
12.00 1.04 2.03 2.00 1.10 0.98 0.99 2.03
f1 (ppm)

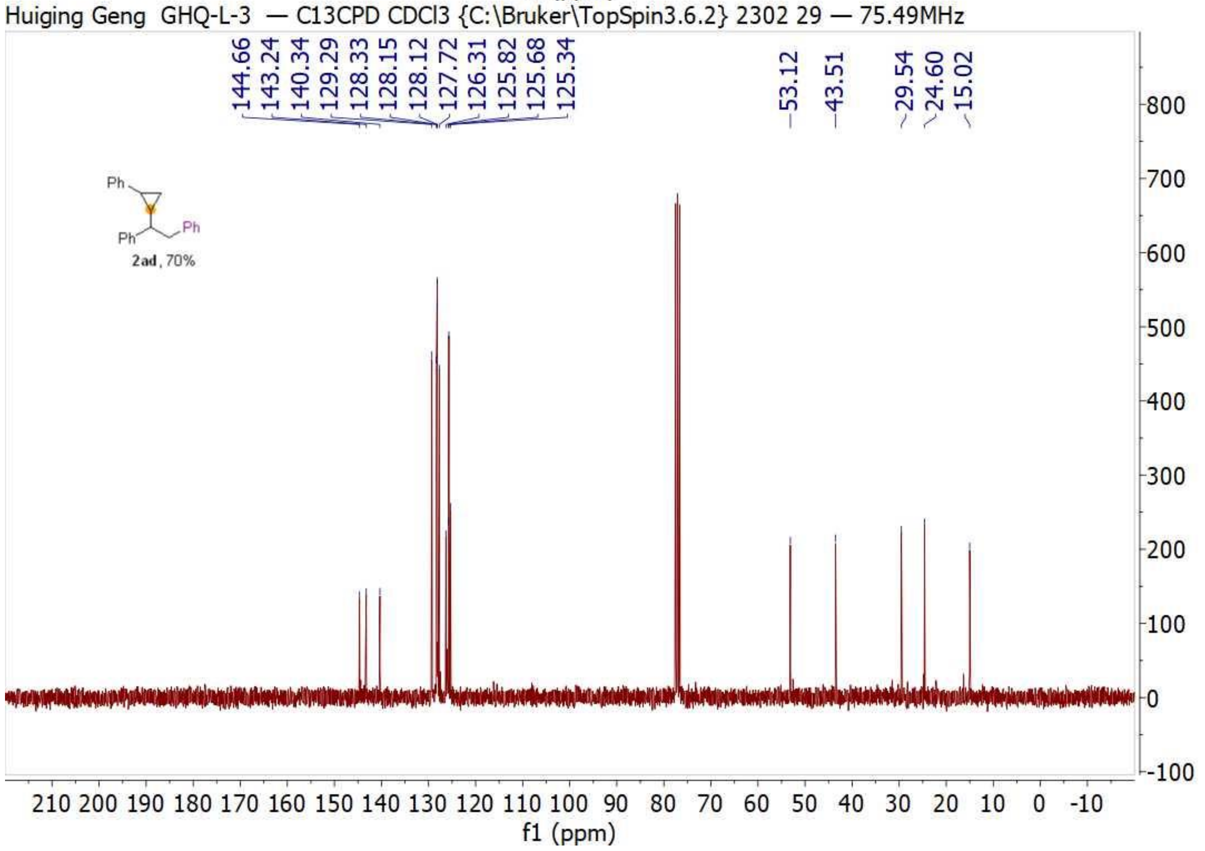
Huiging Geng GHQ-L-3 — C13CPD CDCl3 {C:\Bruker\TopSpin3.6.2} 2302 29 — 75.49MHz
144.66 143.24 140.34 129.29 128.33 128.15 128.12 127.72 126.31 125.82 125.68 125.34
53.12 43.51 29.54 24.60 15.02
Ph
Ph
Ph
2ad, 70%
f1 (ppm)

Geng/ GHQ-LB-14-C — Au1H CDCl3 {C:\Bruker\TopSpin3.6.2} 2211 48 — 300.13MHz

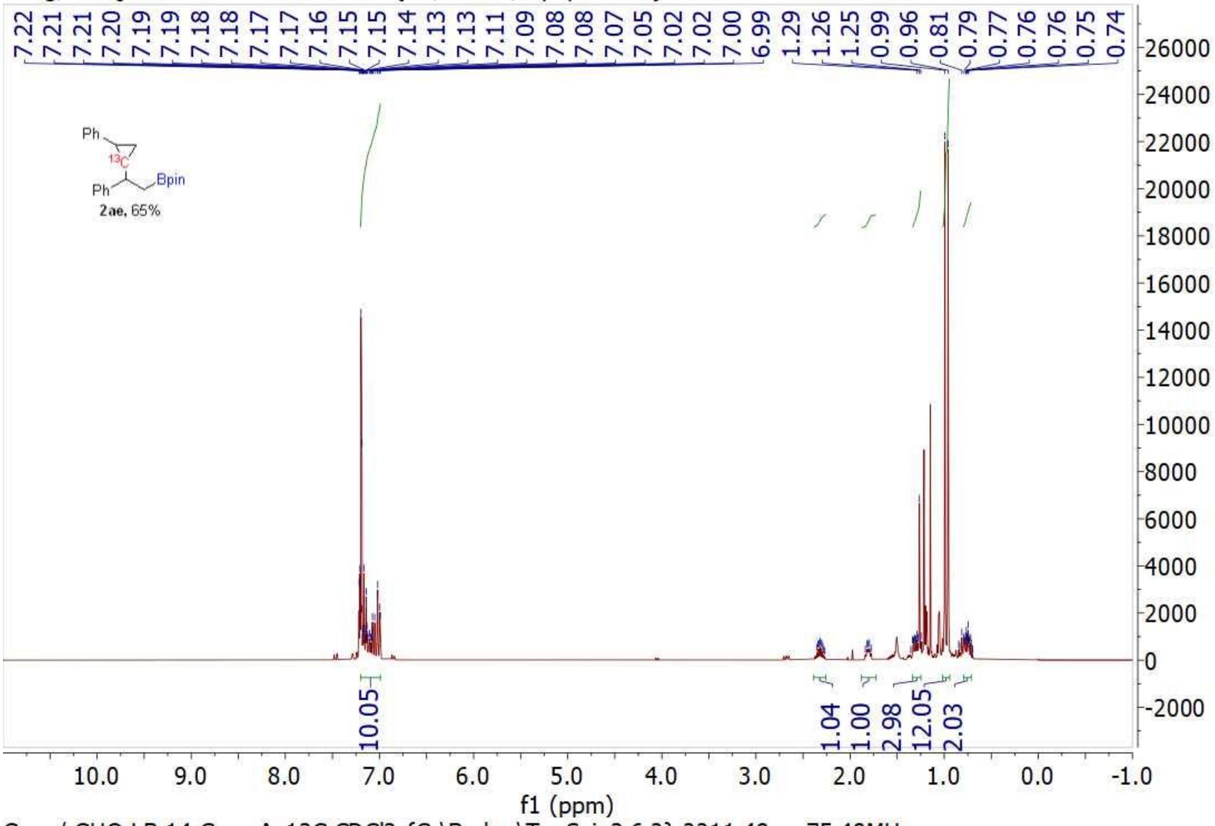

Geng/ GHQ-LB-14-C — Au13C CDCl3 {C:\Bruker\TopSpin3.6.2} 2211 48 — 75.48MHz

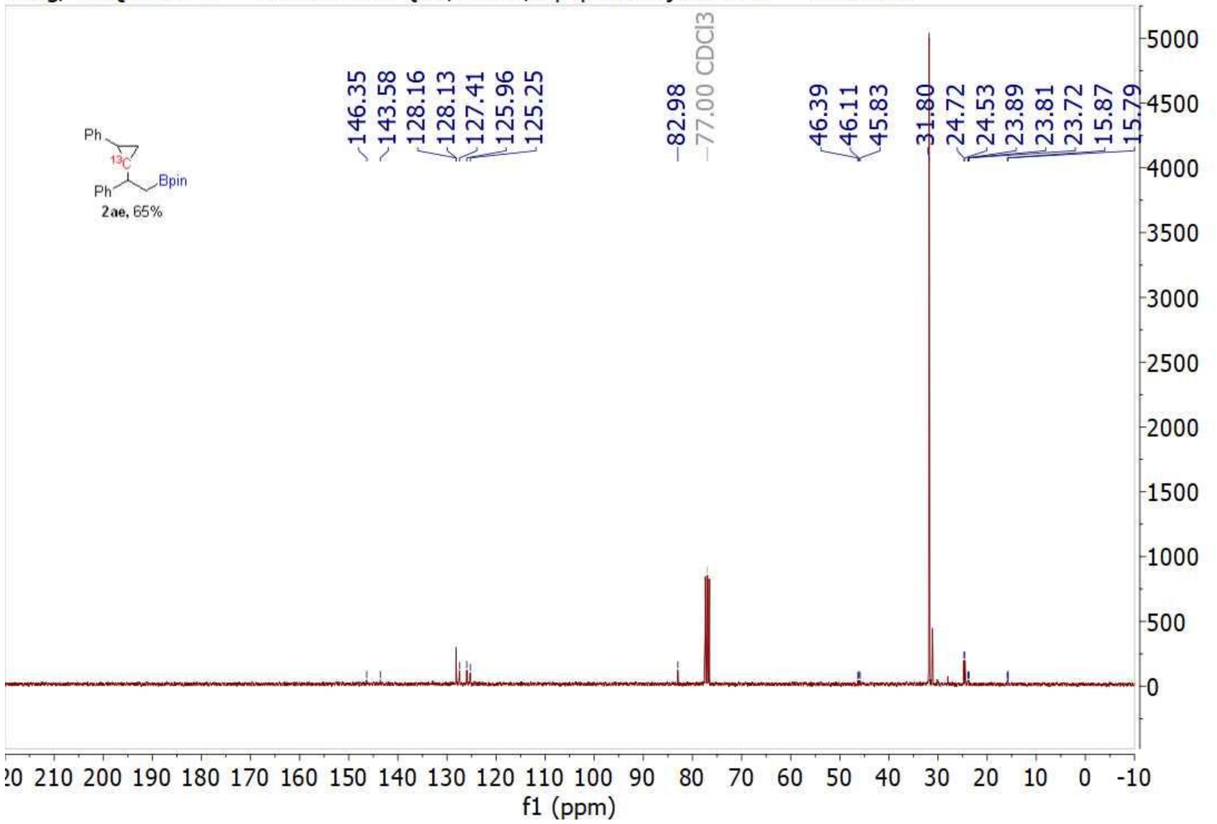

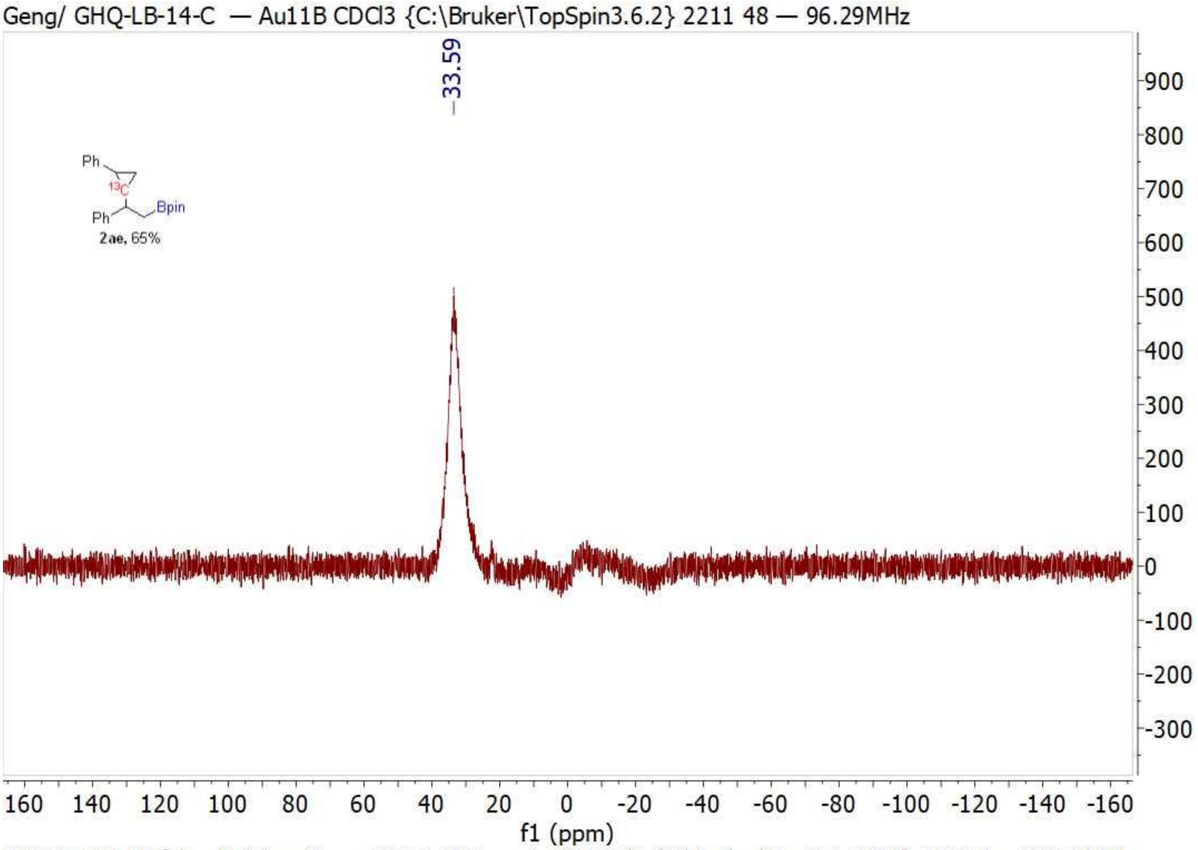
Geng/ GHQ-LB-14-C — Au11B CDCl3 {C:\Bruker\TopSpin3.6.2} 2211 48 — 96.29MHz
33.59
Ph
13C
Ph
Bpin
2ae, 65%
f1 (ppm)

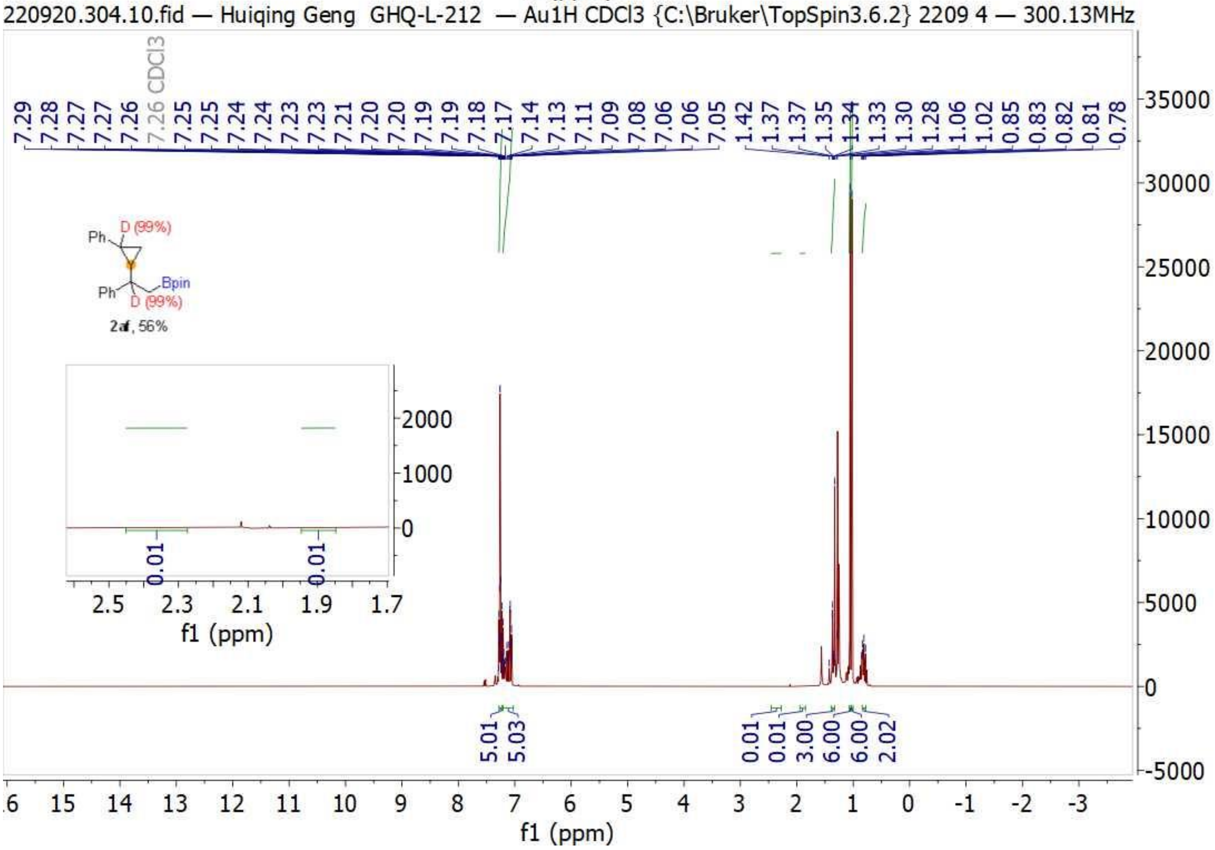
220920.304.10.fid — Huiqing Geng GHQ-L-212 — Au1H CDCl3 {C:\Bruker\TopSpin3.6.2} 2209 4 — 300.13MHz
7.29 7.28 7.27 7.27 7.26 7.26 CDCl3 7.25 7.25 7.24 7.24 7.23 7.23 7.21 7.20 7.20 7.19 7.19 7.18 7.17 7.14 7.13 7.11 7.09 7.08 7.06 7.06 7.05 1.42 1.37 1.35 1.35 1.34 1.33 1.30 1.28 1.06 1.02 0.85 0.83 0.82 0.81 0.78
Ph
D (99%)
Ph
Bpin
D (99%)
2af, 56%
0.01 0.01
5.01 5.03 0.01 0.01 3.00 6.00 6.00 2.02
f1 (ppm)

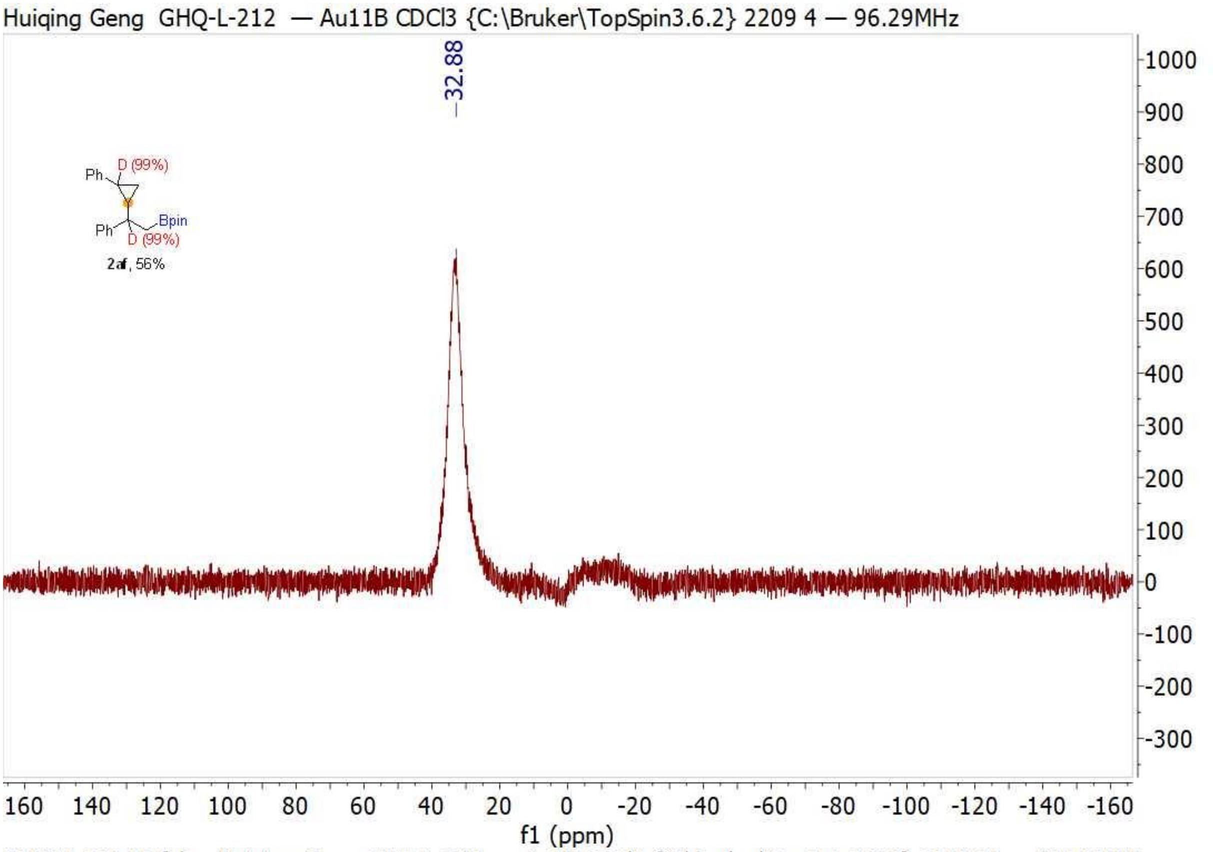
Huiqing Geng GHQ-L-212 — Au11B CDCl3 {C:\Bruker\TopSpin3.6.2} 2209 4 — 96.29MHz
32.88
Ph
D (99%)
Bpin
D (99%)
2af, 56%
f1 (ppm)

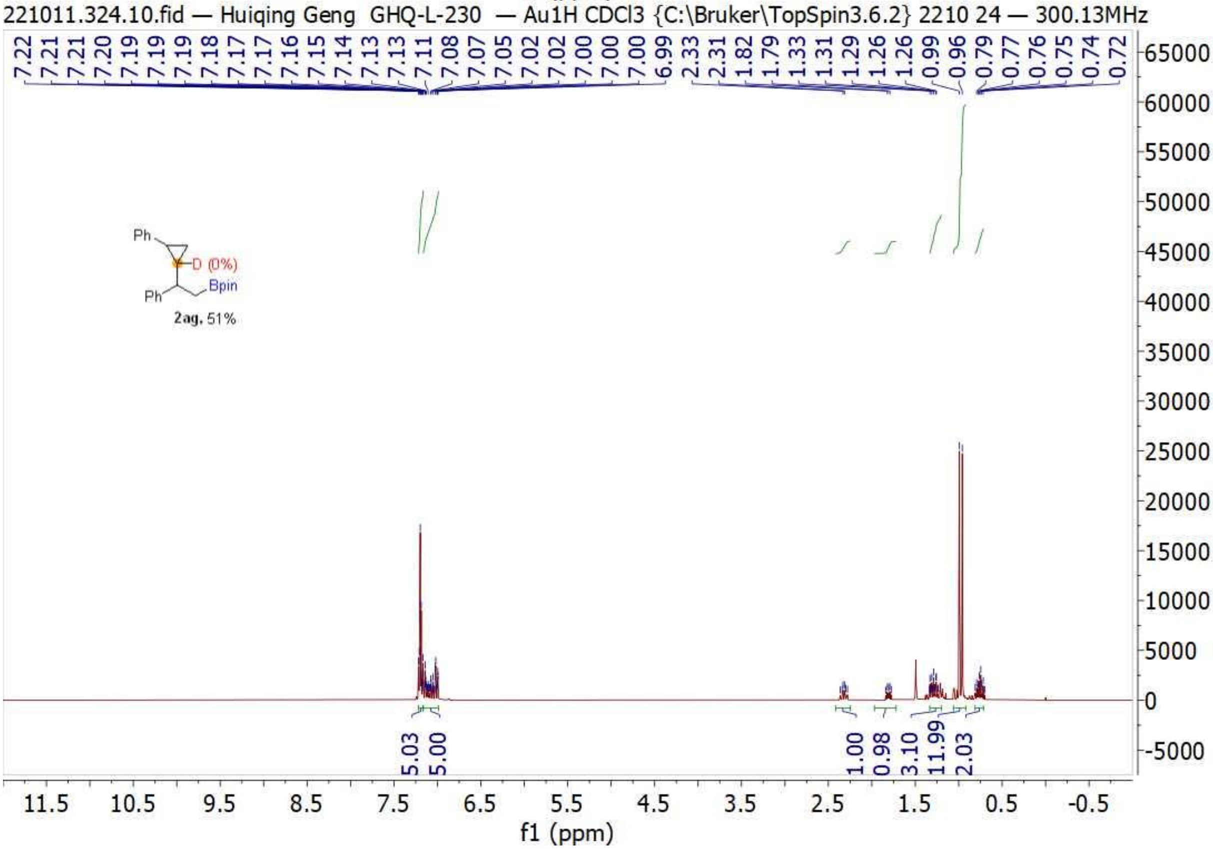
221011.324.10.fid — Huiqing Geng GHQ-L-230 — Au1H CDCl3 {C:\Bruker\TopSpin3.6.2} 2210 24 — 300.13MHz
7.22 7.21 7.21 7.20 7.19 7.19 7.19 7.18 7.17 7.17 7.16 7.15 7.14 7.13 7.13 7.11 7.08 7.07 7.05 7.02 7.02 7.00 7.00 7.00 6.99 2.33 2.31 1.82 1.79 1.33 1.31 1.29 1.26 1.26 0.99 0.96 0.79 0.77 0.76 0.75 0.74 0.72
Ph
D (0%)
Bpin
2ag, 51%
5.03 5.00 1.00 0.98 3.10 11.99 2.03
f1 (ppm)

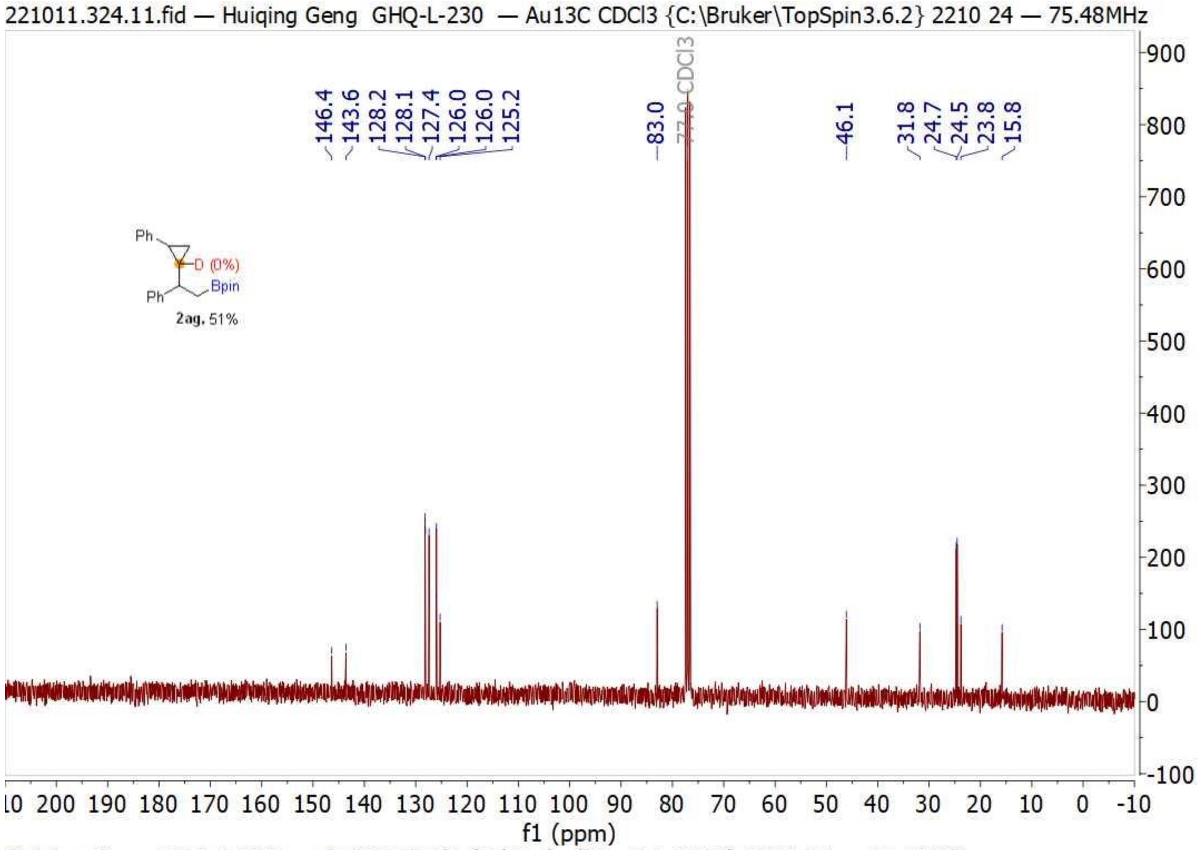
221011.324.11.fid — Huiqing Geng GHQ-L-230 — Au13C CDCl3 {C:\Bruker\TopSpin3.6.2} 2210 24 — 75.48MHz
146.4
143.6
128.2
128.1
127.4
126.0
126.0
125.2
83.0
77.0 CDCl3
46.1
31.8
24.7
24.5
23.8
15.8
Ph
D (0%)
Bpin
Ph
2ag, 51%
f1 (ppm)

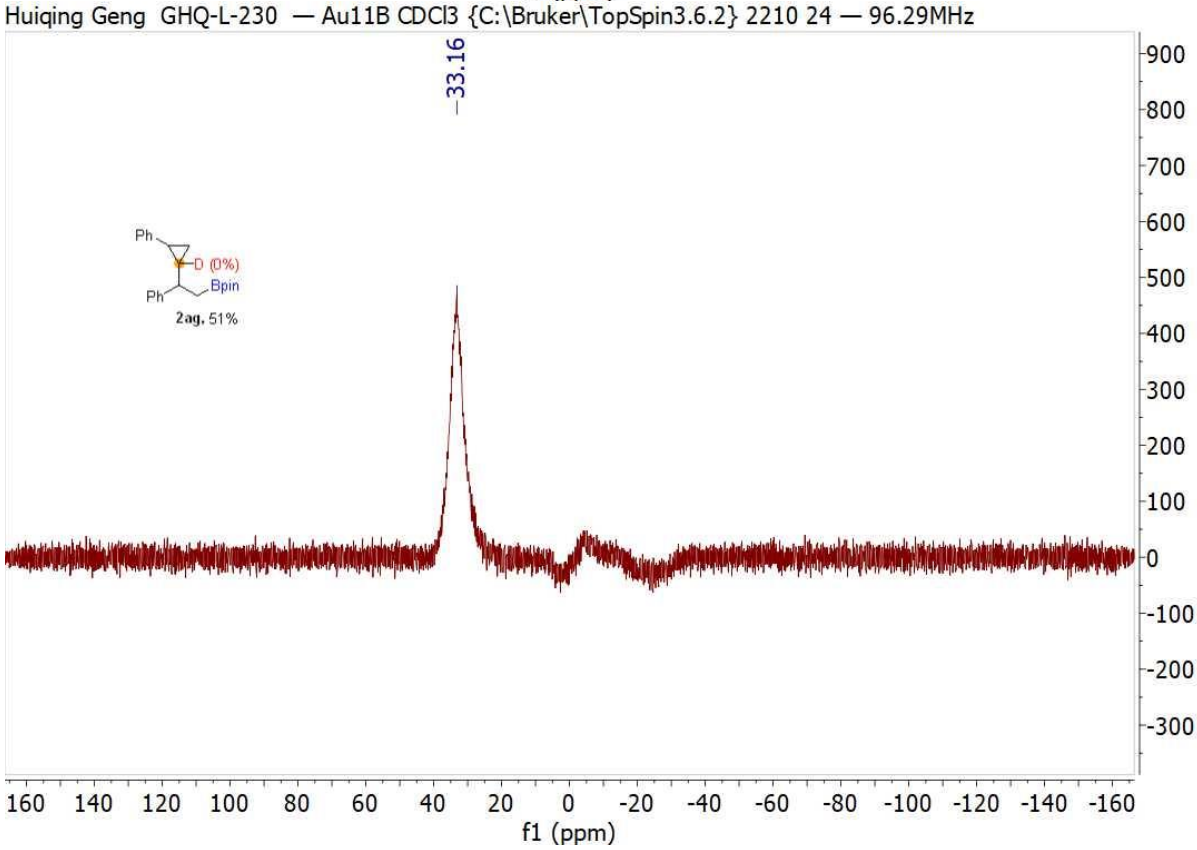
Huiqing Geng GHQ-L-230 — Au11B CDCl3 {C:\Bruker\TopSpin3.6.2} 2210 24 — 96.29MHz
33.16
Ph
D (0%)
Bpin
Ph
2ag, 51%
f1 (ppm)
